# Androgen-dependent alternative mRNA isoform expression in prostate cancer cells

**DOI:** 10.12688/f1000research.15604.1

**Published:** 2018-08-03

**Authors:** Jennifer Munkley, Teresa M. Maia, Nekane Ibarluzea, Karen E. Livermore, Daniel Vodak, Ingrid Ehrmann, Katherine James, Prabhakar Rajan, Nuno L. Barbosa-Morais, David J. Elliott

**Affiliations:** 1 Institute of Genetic Medicine, University of Newcastle, Newcastle upon Tyne, Newcastle, NE1 3BZ, UK; 2 Instituto de Medicina Molecular, Faculdade de Medicina, Universidade de Lisboa, Lisboa, 1649-028, Portugal; 3 VIB Proteomics Core, Albert Baertsoenkaai 3, Ghent, 9000, Belgium; 4 Biocruces Bizkaia Health Research Institute, Cruces University Hospital, Barakaldo, 48903, Spain; 5 Centre for Biomedical Research on Rare Diseases (CIBERER), ISCIII, Valencia, 46010, Spain; 6 Institute of Clinical Medicine, Faculty of Medicine, University of Oslo, Oslo, Norway; 7 Interdisciplinary Computing and Complex BioSystems Research Group, Newcastle University, Newcastle upon Tyne, NE4 5TG, UK; 8 Life and Earth Sciences, Natural History Museum, Cromwell Road, London, SW7 5BD, UK; 9 Barts Cancer Institute, Queen Mary University of London, John Vane Science Centre, London, EC1M 6BQ, UK

**Keywords:** Androgens, AR, prostate cancer, alternative splicing, alternative promoters, alternative 3' ends, transcription, mRNA isoforms

## Abstract

**Background:** Androgen steroid hormones are key drivers of prostate cancer. Previous work has shown that androgens can drive the expression of alternative mRNA isoforms as well as transcriptional changes in prostate cancer cells. Yet to what extent androgens control alternative mRNA isoforms and how these are expressed and differentially regulated in prostate tumours is unknown.

**Methods:** Here we have used RNA-Seq data to globally identify alternative mRNA isoform expression under androgen control in prostate cancer cells, and profiled the expression of these mRNA isoforms in clinical tissue.

**Results:** Our data indicate androgens primarily switch mRNA isoforms through alternative promoter selection. We detected 73 androgen regulated alternative transcription events, including utilisation of 56 androgen-dependent alternative promoters, 13 androgen-regulated alternative splicing events, and selection of 4 androgen-regulated alternative 3′ mRNA ends. 64 of these events are novel to this study, and 26 involve previously unannotated isoforms. We validated androgen dependent regulation of 17 alternative isoforms by quantitative PCR in an independent sample set. Some of the identified mRNA isoforms are in genes already implicated in prostate cancer (including *LIG4*, *FDFT1* and *RELAXIN*), or in genes important in other cancers (e.g. *NUP93* and *MAT2A*). Importantly, analysis of transcriptome data from 497 tumour samples in the TGCA prostate adenocarcinoma (PRAD) cohort identified 13 mRNA isoforms (including *TPD52*, *TACC2* and *NDUFV3*) that are differentially regulated in localised prostate cancer relative to normal tissue, and 3 ( *OSBPL1A*, *CLK3* and *TSC22D3*) which change significantly with Gleason grade and  tumour stage.

**Conclusions:** Our findings dramatically increase the number of known androgen regulated isoforms in prostate cancer, and indicate a highly complex response to androgens in prostate cancer cells that could be clinically important.

## Introduction

A single human gene can potentially yield a diverse array of alternative mRNA isoforms, thereby expanding both the repertoire of gene products and subsequently the number of alternative proteins produced. mRNAs with different exon combinations are transcribed from most (up to 90%) human genes, and can generate variants that differ in regulatory untranslated regions, or encode proteins with different sub-cellular localisations and functions ^1– 5^. Altered splicing patterns have been suggested as a new hallmark of cancer cells ^6– 8^, and in prostate cancer there is emerging evidence that expression of specific mRNA isoforms derived from cancer-relevant genes may contribute to disease progression ^9– 11^.

Androgen steroid hormones and the androgen receptor (AR) play a key role in the development and progression of prostate cancer, with alternative splicing enabling cancer cells to produce constitutively active ARs ^11– 13^. The AR belongs to the nuclear receptor superfamily of transcription factors, and is essential for prostate cancer cell survival, proliferation and invasion ^14– 16^. Classically, androgen binding promotes AR dimerization and its translocation to the nucleus, where it acts as either a transcriptional activator or a transcriptional repressor to dictate prostate specific gene expression patterns ^17– 23^. The major focus for prostate cancer therapeutics has been to reduce androgen levels through androgen deprivation therapy (ADT), either with inhibitors of androgen synthesis (for example, abiraterone) or with antagonists that prevent androgen binding to the AR (such as bicalutamide or enzalutamide) ^24^. Although ADT is usually initially effective, most patients ultimately develop lethal castrate resistant disease for which there are limited treatment options ^11, 12^.

Androgens and other steroid hormones have also been associated with alternative splicing. Recent RNA-sequencing-based analysis of the androgen response of prostate cancer cells grown *in vitro* and within patients following ADT identified a set of 700 genes whose transcription is regulated by the AR in prostate cancer cells ^25^. However, in addition to regulating transcriptional levels, steroid hormone receptors can control exon content of mRNA ^10, 26– 29^. In prostate cancer androgens can modulate the expression of mRNA isoforms via pre-mRNA processing and promoter selection ^9, 10, 18, 30^. The AR can recruit the RNA binding proteins Sam68 and p68 as cofactors to influence alternative splicing of specific genes, and studies using minigenes driven from steroid responsive promoters indicate that the AR can affect both the transcriptional activity and alternative splicing of a subset of target genes ^11, 31, 32^. Other steroid hormones also coordinate both transcription and splicing decisions ^29^. The thyroid hormone receptor (TR) is known to play a role in coordinating the regulation of transcription and alternative splicing ^27^, and the oestrogen receptor (ER) can both regulate alternative promoter selection and induce alternative splicing of specific gene sets that can influence breast cancer cell behaviour ^28, 33– 35^.

In previous work we used exon level microarray analysis to identify 7 androgen dependent changes in mRNA isoform expression ^10^. However, to what extent androgen-regulated mRNA isoforms are expressed in clinical prostate cancer is unclear. To address this, here we have used RNA-Sequencing data to globally profile alternative isoform expression in prostate cancer cells exposed to androgens, and correlated the results with transcriptomic data from clinical tissue. Our findings increase the number of known AR regulated mRNA isoforms by 10 fold and imply that pre-mRNA processing is an important mechanism through which androgens regulate gene expression in prostate cancer.

## Methods

### Cell culture

Cell culture was as described previously ^25, 36^. All cells were grown at 37°C in 5% CO _2_. LNCaP cells (CRL-1740, ATCC) were maintained in RPMI-1640 with L-Glutamine (PAA Laboratories, R15-802) supplemented with 10% Fetal Bovine Serum (FBS) (PAA Laboratories, A15-101). For androgen treatment of cells, medium was supplemented with 10% dextran charcoal stripped FBS (PAA Laboratories, A15-119) to produce a steroid-deplete medium. Following culture for 72 hours, 10 nM synthetic androgen analogue methyltrienolone (R1881) (Perkin-Elmer, NLP005005MG) was either added (Androgen +) or absent (Steroid deplete) for the times indicated.

### RNA-Seq analysis

RNA-seq transcript expression analysis of previously generated data ^25^ was performed according to the Tuxedo protocol ^37^. All reads were first mapped to human transcriptome/genome (build hg19) with TopHat ^38^/Bowtie ^39^, followed by per-sample transcript assembly with Cufflinks ^40^. The mapped data was processed with Cuffmerge, Cuffdiff and Cuffcompare, followed by extraction of significantly differentially expressed genes/isoforms; expression changes between cells grown with androgen and cells grown without androgens were assessed. Reference files for the human genome (UCSC build hg19) were downloaded from the Cufflinks pages: ( UCSC-hg19 package from June 2012 was used.). The software versions used for the analysis were: TopHat v1.4.1, SAM tools Version: 0.1.18 (r982:295), bowtie version 0.12.8 (64-bit) and cufflinks v1.3.0 (linked against Boost version 104000). The Tuxedo protocol ^37^ was carried out as follows: For steps 1–5, no parameters (except for paths to input/output files) were altered. In step 15, additional switches -s, -R, and -C were used when running cuffcompare. Steps 16–18 (extraction of significant results) were performed on the command line.

### RNA extraction, RT–PCR and real-time PCR

Cells were harvested and total RNA extracted using TRIzol (Invitrogen, 15596-026) according to manufacturer's instructions. RNA was treated with DNase 1 (Ambion, AM2222) and cDNA was generated by reverse transcription of 500ng of total RNA using the Superscript VILO cDNA synthesis kit (Invitrogen, 11754-050). Alternative events were analysed by either reverse transcriptase PCR or real-time PCR. Exon profiles were monitored and quantified using the Qiaxcel capillary electrophoresis system (Qiagen) and percentage inclusion was calculated as described previously ^10^. Real time PCR was performed in triplicate on cDNA using SYBR® Green PCR Master Mix (Invitrogen, 4309155) and the QuantStudio 7 Flex Real-Time PCR System (Thermo Fisher Scientific). Samples were normalised using the average of three reference genes, GAPDH, β -tubulin and actin. Ct values for each sample were calculated using SDS 2.4 software (Applied Biosystems) and relative mRNA expression was calculated using the 2-ΔΔCt method. All primer sequences are listed in Supplementary Table 1. Raw Ct values are given in Dataset 1 ^41^.

### Antibodies

The following commercial antibodies were used in the study: anti-RLN2 rabbit monoclonal (Abcam, ab183505 1:1000 dilution), anti-TACC2 rabbit polyclonal antibody (11407-1-AP, Proteintech 1:500 dilution), anti-NDUFV3 rabbit polyclonal antibody (13430-1-AP, Proteintech 1:500 dilution), anti-actin rabbit polyclonal (A2668, Sigma 1:2000 dilution), anti-α-Tubulin mouse monoclonal (Sigma, T5168 1:2000 dilution), normal rabbit IgG (711-035-152, Jackson labs 1:2000 dilution) and normal mouse IgG (715-036-150, Jackson labs 1:2000 dilution).

### Gene ontology analysis

Gene ontology (GO) analysis of RNA-Seq data was carried out as described previously ^42^. Enrichment of GO terms (with b500 annotations) was calculated using the goseq R package (version 1.18.0). Genes were considered significant at a p-value threshold of 0.05 after adjustment using the Benjamini-Hochberg false discovery rate.

### Bioinformatic analysis of patient transcriptome data

Available clinical and processed RNA-Seq data from The Cancer Genome Atlas (TCGA) prostate adenocarcinoma (PRAD) cohort, comprising 497 tumour samples from as many patients with different stages / Gleason grades and 52 matched samples taken from normal prostate tissue (were downloaded from the Broad Institute TCGA Genome Analysis Center (Firehose 16/01/28 run https://doi.org/10.7908/C11G0KM9 ^43^). Transcriptome data from the TCGA PRAD cohort were analysed for alternative isoform expression, with transcript models relying on TCGA GAF2.1, corresponding to the University of California, Santa Cruz (UCSC) genome annotation from June 2011 ( hg19 assembly). This annotation encompassed 42 of the 73 androgen-regulated alternative mRNA isoform pairs identified. These were studied using two types of analysis: 1) differential transcript expression between tumour and normal prostate tissue and 2) correlation between isoform expression in tumour samples and Gleason score or tumour stage.

Differential isoform and gene expression analysis was performed on estimated read counts using the *limma* software R package (version 3.7) following its RNA-Seq analysis workflow ^44^. This workflow was also used for differential isoform ratio analysis, relying on logit-transformed ratio (see below). An FDR-adjusted p-value of 0.05 for the moderated t-statistics was used as threshold for significance of differential expression. Individual isoform expression was estimated in TPM (transcripts per million mapped reads). The expression ratio, henceforth called PSI (percent spliced-in), of each annotated androgen-regulated isoform pair in each TCGA sample was calculated as the ratio between the expression of isoform 1 and the total expression of isoforms 1 and 2 combined, i.e. the sum of their expressions. For each isoform pair, ΔPSI is the difference of median PSI between the tumour and the normal groups of samples.

Two-tailed Spearman’s rank correlation tests were used to study the association between isoform expression and both Gleason score and tumour stage (these were used herein as numeric variables). An FDR-adjusted p-value of 0.05 was used as threshold for significance. Isoform expression differences between tumour and normal samples were considered equivalent to those detected in LNCaP cells under androgen stimulation when there was a statistically significant consistent change in the levels of the expected induced or repressed isoform (1 or 2), concomitant with no contradictory change in the PSI. Isoform “switches” were considered equivalent when there was a minimum (ΔPSI > 2.5%) and statistically significant consistent change in the PSI. Equivalent criteria were used to evaluate the equivalence between androgen-dependence and the associations with Gleason score and tumour stage.

## Statistical analysis

Statistical analyses were conducted using the GraphPad Prism software (version 5.04/d). PCR quantification of mRNA isoforms was assessed using the unpaired student’s t-test.

Data is presented as the mean of three independent samples ± standard error of the mean (SEM). Statistical significance is denoted as * p ≤ 0.05, ** p ≤ 0.01, *** p ≤ 0.001 and **** p ≤ 0.0001.

## Results

### Global identification of androgen-dependent mRNA isoform production in prostate cancer cells predicts a major role for alternative promoter utilisation

We analysed previously published RNAseq data from LNCaP cells ^25^ to globally profile how frequently androgens drive production of alternative mRNA isoforms in prostate cancer cells. This analysis identified a group of 73 androgen regulated alternative mRNA isoforms, which could be validated by visualisation on the UCSC Genome Browser ^45^ ( Table 1). 64 AR regulated mRNA isoforms were novel to this study. Experimental validation in an independent RNA sample set using RT-PCR confirmed 17/17 of these alternative events at the mRNA level ( Supplementary Figure 1). 73% of genes (53/73) with identified alternative androgen regulated mRNA isoforms also changed their overall expression levels in response to androgens ( Table 2). Some of the androgen regulated alternative events are in genes are already implicated in in either prostate cancer or other cancer types (summarised in Table 3). However, Gene Ontology analysis of these 73 genes did not identify any significantly enriched biological processes.

**Table 1.** Details of the 73 androgen regulated mRNA isoforms identified in prostate cancer cells.

		Isoform 1		Isoform 2					TCGA PRAD		
Gene	Event type	Position (hg19)	RefSeq	Position (hg19)	RefSeq	Change with androgens	PCR Validation	Predicted to change protein?	Isoform 1 ID	Isoform 2 ID	Comparable?
**LIG4**	Alternative promoter	chr13:108859792- 108870716	NM_001098268.1	chr13:108859792- 108867130	NM_002312.3	Induction of promoter 2	Yes (Qiaxel)	No (5' UTR)	uc001vqp.2	uc001vqn.2	Yes
**TACC2**	Alternative promoter	chr10:123748689- 124014060	NM_206862.3	chr10:123872554- 124014060	NM_001291879.1	Repression of promoter 1	Yes (Qiaxel)	Yes	uc001lfv.2	uc001lfx.2	Yes
**TPD52**	Alternative promoter	chr8:80947103- 81083894	NM_001287144.1	chr8:80947103- 80993066	NM_001025252.2	Induction of promoter 2	Yes (Qiaxel)	Yes	uc003ybs.1	uc003ybr.1	Yes
**NUP93**	Alternative promoter	chr16:56764017- 56878861	NM_014669.4	chr16:56815704- 56878861	NM_001242795.1	Induction of promoter 1	Yes (SYBR)	Yes	uc002eka.2	uc002ekb.2	Yes
**RLN1**	Alternative promoter	chr9:5334932- 5339873	NM_006911.3	chr9:5335270- 5339396	Not annotated	Repression of promoter 2	Yes (Qiaxel)	Yes (change from non- coding)	uc003zjb.1	Not annotated	No
**AP2S1**	Alternative promoter	chr19:47341415- 47354252	NM_001301078.1	chr19:47341415- 47353547	NM_001301076.1	Induction of promoter 2	Yes (SYBR)	Yes	uc002pft.1	Not annotated	No
**RLN2**	Alternative promoter	chr9:5299866- 5304611	NM_005059.3	chr9:5299890- 5304222	Not annotated	Induction of promoter 1	Yes (Qiaxel)	Yes (change from non- coding)	uc003ziz.1	Not annotated	No
**PIK3R1**	Alternative promoter	chr5:67511584- 67597649	NM_181523.2	chr5:67584252- 67597649	NM_181524.1	Repression of promoter 2	Yes (SYBR)	Yes	uc003jva.2	uc003jvc.2	Yes
**MAPRE2**	Alternative promoter	chr18:32556892- 32723432	NM_001143826.2	chr18:32621324- 32723432	NM_014268.3	Switch to promoter 2	Yes (Qiaxel)	Yes	uc010xcb.1	uc002kyf.2	Yes
**NDUFAF4**	Alternative promoter	chr6:97337187- 97345767	NM_014165.3	chr6:97337227- 97345368	Not annotated	Repression of promoter 2	Yes (Qiaxel)	Yes (change from non- coding)	uc003pov.2	Not annotated	No
**DCXR**	Alternative promoter	chr17:79993757- 79995573	NM_016286.3	chr17:79993765- 79995217	Not annotated	Repression of promoter 2	Yes (Qiaxel)	Yes	uc002kdg.2	Not annotated	No
**PEX10**	Alternative promoter	chr1:2336241- 2344010	NM_002617.3	Not annotated		Switch to promoter 2	Yes (Qiaxel)	Yes	uc001ajh.2	Not annotated	No
**SNAPC2**	Alternative promoter	chr19:7985194- 7988136	NM_003083.3	chr19:7985867- 7988136	NR_030717.1	Switch to promoter 2	Yes (SYBR)	Yes (change to non- coding)	uc002miw.1	uc002mix.1	Yes
ATP6V0D1	Alternative promoter	chr16:67471917- 67515089	NM_004691.4	chr16:67471931- 67475338	Not annotated	Repression of promoter 2		Yes	uc002ete.1	Not annotated	No
ARRDC1	Alternative promoter	chr9:140500092- 140509812	NM_001317968.1	chr9:140506874- 140509793	Not annotated	Induction of promoter 2		Yes (change to non- coding)	uc004cnp.1	Not annotated	No
DENND1A	Alternative promoter	chr9:126141933- 126692417	NM_020946.1	chr9:126143408- 126586780	Not annotated	Repression of promoter 2		Yes	uc004bnz.1	Not annotated	No
KLHL36	Alternative promoter	chr16:84682117- 84701292	NM_024731.3	chr16:84684274- 84701134	Not annotated	Induction of promoter 2		Yes	uc002fig.2	Not annotated	No
RAB3IL1	Alternative promoter	chr11:61664768- 61687741	NM_001271686.1	chr11:61664768- 61685081	NM_013401.3	Repression of promoter 2		Yes	uc001nsp.2	uc001nso.2	Yes
ACER3	Alternative promoter	chr11:76571917- 76737841	NM_018367.6	chr11:76631206- 76737818	Not annotated	Repression of promoter 2		Yes	uc009yum.1	Not annotated	No
OSBPL1A	Alternative promoter	chr18:21742011- 21977833	NM_080597.3	chr18:21742011- 21852196	NM_018030.4	Induction of promoter 2		Yes	uc002kve.2	uc002kvd.2	Yes
TRIM16	Alternative promoter	chr17:15531280- 15586193	NM_006470.3	chr17:15530970- 15555735	Not annotated	Induction of promoter 2		Yes	uc002gow.2	Not annotated	No
VSIG10L	Alternative promoter	chr19:51834795- 51845378	NM_001163922.1	chr19:51834795- 51843009	Not annotated	Induction of promoter 1		Yes	uc002pwf.2	Not annotated	No
SEPT5	Alternative promoter	chr22:19701987- 19710845	NM_002688.5	chr22:19705958- 19710845	NM_001009939.2	Repression of promoter 2		Yes	uc002zpv.1	uc002zpw.1	Yes
HMGCR	Alternative promoter	chr5:74632154- 74657926	NM_000859	chr5:74632993- 74657926	NM_000859.2	Repression of promoter 1		Yes	uc011cst.1	uc003kdp.2	Yes
RDH13	Alternative promoter	chr19:55555692- 55580914	NM_138412.3	chr19:55555692- 55574585	NM_001145971.1	Induction of promoter 1		Yes	uc002qip.2	uc010esr.1	Yes
GPRIN2	Alternative promoter	chr10:46993001- 47000677	Not annotated	chr10:46993546- 47000568	NM_014696.3	Repression of promoter 2		No (5' UTR)	Not annotated	uc001jec.2	No
CLK3	Alternative promoter	chr15:74900713- 74922542	NM_003992.4	chr15:74,908,246- 74,922,542	NM_003992	Repression of promoter 1		Yes	uc002ayg.3	uc002ayj.3	Yes
RNH1	Alternative promoter	chr11:494512- 507283	NM_203387.2	chr11:494512- 506821	NM_002939.3	Induction of promoter 1		No (5' UTR)	uc001lpp.1	uc001lpl.1	Yes
ZFAND6	Alternative promoter	chr15:80351910- 80430735	NM_001242911.1	chr15:80364903- 80430735	NM_001242916.1	Repression of promoter 2		No (5' UTR)	uc002bff.1	uc002bfh.1	Yes
CDIP1	Alternative promoter	chr16:4560677- 4588816	NM_013399.2	chr16:4560677- 4588471	NM_001199054.1	Repression of promoter 2		No (5' UTR)	uc002cwu.2	uc002cwv.2	Yes
YIF1B	Alternative promoter	chr19:38794200- 38806606	NM_001039672.2	chr19:38794200- 38806445	NM_001145461.1	Switch to promoter 2		Yes	uc002ohz.2	uc002ohx.2	Yes
LIMK2	Alternative promoter	chr22:31608250- 31676066	NM_005569.3	chr22:31644348- 31676066	NM_016733.2	Switch to promoter 2		Yes	uc003akh.2	uc003aki.2	Yes
TSC22D3	Alternative promoter	chrX:106956452- 106959711	NM_001015881.1	chrX:106956452- 106960291	NM_004089.3	Repression of promoter 1		Yes	uc004enf.2	uc004eng.2	Yes
ALDH1A3	Alternative promoter	chr15:101419897- 101456830	NM_000693.3	chr15:101438281- 101457072	Not annotated	Repression of promoter 1		Yes	uc002bwn.3	Not annotated	No
TRABD	Alternative promoter	chr22:50624341- 50638028	NM_001320485.1	chr22:50628979- 50638028	NM_001320487.1	Switch to promoter 2		No (5' UTR)	uc003bjq.1	uc003bjs.1	Yes
LIMCH1	Alternative promoter	chr4:41361624- 41702061	NM_001289124.1	chr4:41362648- 41702061	NM_001289122.2	Repression of promoter 2		Yes	uc003gvu.3	Not annotated	No
GMFB	Alternative promoter	chr14:54941209- 54955744	NM_004124.2	chr14:54941314- 54955637	Not annotated	Induction of promoter 2		Yes (change to non- coding)	uc010tqz.1	Not annotated	No
MLST8	Alternative promoter	chr16:2255178- 2259418	NM_022372.4	chr16:2255732- 2259418	NM_001199174.1	Switch to promoter 1		No (5' UTR)	uc010uvy.1	uc002cpf.2	Yes
TLE3	Alternative promoter	chr15:70340130- 70390256	NM_020908.2	chr15:70340130- 70387124	NM_001282982.1	Induction of promoter 2		Yes	uc002asn.2	uc002ask.2	Yes
UBA1	Alternative promoter	chrX:47050199- 47074527	NM_153280.2	chrX:47053201- 47074527	NM_003334.3	Repression of promoter 1		No (5' UTR)	uc004dhj.3	uc004dhk.3	Yes
TNRC6B	Alternative promoter	chr22:40440821- 40731812	NM_001024843.1	chr22:40573929- 40731812	NM_001162501.1	Repression of promoter 2		Yes	uc003aym.2	uc011aor.1	Yes
FDFT1	Alternative promoter	chr8:11660120- 11696818	NM_004462.4	chr8:11665926- 11696818	NM_001287750.1	Repression of promoter 2		Yes	uc003wui.2	uc010lsb.2	Yes
GREB1	Alternative promoter	chr2:11674242- 11782912	NM_014668.3	chr2:11680080- 11728355	NM_148903.2	Induction of promoter 2		Yes	uc002rbo.1	uc002rbl.2	Yes
NCAPD3	Alternative promoter	chr11:134022337- 134094426	NM_015261.2	chr11:134022772- 134093593	Not annotated	Induction of promoter 2		Yes	uc001qhd.1	Not annotated	No
SLC36A4	Alternative promoter	chr11:92877337- 92931141	NM_152313.3	chr11:92877337- 92930621	NM_001286139.1	Induction of promoter 2		Yes	uc001pdn.2	Not annotated	No
KLC2	Alternative promoter	chr11:66024765- 66035331	NM_001134775.1	chr11:66025174- 66035331	NM_022822.2	Repression of promoter 1		No (5' UTR)	uc010rov.1	uc001ohb.2	Yes
RAP1GAP	Alternative promoter	chr1:21922708- 21978348	NM_001145658.1	chr1:21922533- 21946950	Not annotated	Repression of promoter 1		Yes	uc001bez.1	Not annotated	No
TMEM79	Alternative promoter	chr1:156252704- 156262234	NR_026678.1	chr1:156254070- 156262234	NM_032323.2	Repression of promoter 1		No (5' UTR)	uc001fod.2	uc010phi.1	Yes
NR4A1	Alternative promoter	chr12:52416616- 52453291	NM_001202233.1	chr12:52445186- 52453291	NM_173157.2	Induction of promoter 2		Yes	uc010sno.1	uc001rzr.2	Yes
ZNF32	Alternative promoter	chr10:44139307- 44144326	NM_001324166.1	chr10:44139307- 44144326	NM_001324167.1	Repression of promoter 2		No (5' UTR)	uc001jbc.2	uc001jbb.2	Yes
C1QTNF3	Alternative promoter	chr5:34017963- 34043371	NM_181435.5	chr5:34018571- 34035881	Not annotated	Induction of promoter 1		Yes	uc003jio.2	Not annotated	No
UBE2D3	Alternative promoter	chr4:103715540- 103748710	NM_181887.2	chr4:103715540- 103749105	NM_181886.3	Switch to promoter 2		No (5' UTR)	uc003hwk.2	uc011cet.1	Yes
KRT8	Alternative promoter	chr12:53290971- 53343650	NM_001256293.1	chr12:53,290,971- 53,298,868	NM_002273	Repression of promoter 1		No (5' UTR)	uc009zml.1	uc001sbd.2	Yes
ELOVL1	Alternative promoter	chr1:43829068- 43833745	NM_022821.3	chr1:43829093- 43832057	Not annotated	Induction of promoter 2		Yes (change to non- coding)	uc001cjb.2	Not annotated	No
RCAN1	Alternative promoter	chr21:35888740- 35987441	NM_004414.6	chr21:35888740- 35899308	NM_203418.2	Induction of promoter 2		Yes	uc002yue.2	uc002yub.2	Yes
SORBS3	Alternative promoter	chr8:22409251- 22433008	NM_005775.4	chr8:22422332- 22433100	Not annotated	Induction of promoter 2		Yes	uc003xbv.2	Not annotated	No
**MAT2A**	Alternative 3' end	chr2:85766101- 85772403	NM_005911.5	chr2:85,766,101- 85,770,775	NM_005911	Repression of isoform 2	Yes (Qiaxel)	Yes	uc002spr.2	uc010ysr.1	Yes
**CNNM2**	Alternative 3' end	chr10:104678075- 104687375	NM_199077.2	chr10:104678075- 104838344	NM_017649.4	Induction of isoform 1	Yes (SYBR)	Yes	uc001kwl.2	uc001kwm.2	Yes
TMEM125	Alternative 3' end	chr1:43735698- 43736343	Not annotated	chr1:43735665- 43739673	NM_144626.2	Induction of isoform 1		Yes (change to non- coding)	Not annotated	uc001cir.2	No
CBWD2	Alternative 3' end	chr2:114195268- 114253781	NM_172003.3	chr2:114195169- 114199073	Not annotated	Induction of isoform 2		Yes	uc002tju.2	Not annotated	No
NDUFV3	Alternative exon	chr21:44313378- 44329773	NM_021075.3	chr21:44313378- 44329773	NM_001001503.1	Switch to isoform 2 (exon excluded)		Yes	uc002zcm.2	uc002zcn.2	Yes
ZNF678	Alternative exon	chr1:227751220- 227850164	NM_178549.3	Not annotated		Switch to isoform 2 (exon excluded)		Yes (change to non- coding)	uc009xet.1	Not annotated	No
ZNF121	Alternative exon	chr19:9676404- 9695209	NM_001308269.1	chr19:9676404- 9695209	NM_001008727.3	Switch to isoform 2 (exon excluded)		Yes	uc010xkq.1	uc010xkp.1	Yes
SPATC1L	Alternative exon	chr21:47581062- 47604373	NM_032261.4	Not annotated		Induction of isoform 2 (exon included)		Yes	uc002zii.2	Not annotated	No
MOCOS	Alternative exon	chr18:33767480- 33848685	NM_017947.2	Not annotated		Switch to isoform 2 (exon excluded)		Yes	uc002kzq.3	Not annotated	No
RBM45	Alternative exon	chr2:178977151- 178994382	NM_152945.3	Not annotated		Switch to isoform 2 (exon included)		Yes	uc002ulv.2	Not annotated	No
MIPEP	Alternative exon	chr13:24304328- 24463587	NM_005932.3	Not annotated		Repression of isoform 2 (exon excluded)		Yes	uc001uox.3	Not annotated	No
BBS4	Alternative exon	chr15:72978520- 73030817	NM_001320665.1	Not annotated		Induction of isoform 2 (exon included)		Yes	uc002avb.2	Not annotated	No
FAM195A	Alternative exon	chr16:691804- 698474	NM_138418.3	chr16:691804- 698474	NR_138607.1	Switch to isoform 1 (exon exluded)		Yes (change from non- coding)	uc002cic.1	uc002cie.2	Yes
LINC01133	Alternative exon	chr1:159931008- 159948851	ENST00000443364.6	chr1:159931014- 159948876	NR_038849.1	Induction of isoform 1 (exon excluded)		Both non-coding	Not annotated	uc001fuu.2	No
SS18	Alternative exon	chr18:23596217- 23670611	NM_001007559.2	chr18:23596217- 23670611	NM_005637.3	Switch to isoform 2 (exon excluded)		Yes	uc002kvm.2	uc002kvn.2	Yes
RHOC	Alternative exon	chr1:113243897- 113249757	ENST00000369638.6	chr1:113243947- 113249742	ENST00000369636.6	Switch to isoform 2 (exon excluded)		No (5' UTR)	uc009wgk.1	uc001ecr.1	Yes
ZNF226	Retained intron	chr19:44669215- 44681838	NM_001319088.1	chr19:44669249- 44679582	NM_015919.3	Switch to isoform 1 (intron included)		Yes	uc002oyo.2	uc002oyn.2	Yes

**Table 2.** Quantitative changes in gene expression in response to androgens for the 73 genes with AR regulated alternative mRNA isoforms.

LNCaP RNA-Seq (+/- androgens for 24 hours)			Reciprocal RNA-Seq (also change in 7 patients following ADT)		
No change	Upregulated	Downregulated	No change	Upregulated	Downregulated
RLN2	LIG4	NUP93	LIG4	TPD52	None
DENND1A	TACC2	PIK3R1	TACC2	AP2S1	
RAB3IL1	RLN1	MAPRE2	NUP93	DCXR	
OSBPL1A	AP2S1	NDUFAF4	RLN1	PEX10	
TRIM16	DCXR	ACER3	RLN2	HMGCR	
Sep-05	PEX10	GPRIN2	PIK3R1	ALDH1A3	
RDH13	SNAPC2	TLE3	MAPRE2	FDFT1	
ZFAND6	ATP6V0D1	TNRC6B	NDUFAF4	GREB1	
CDIP1	ARRDC1	SORBS3	SNAPC2	NCAPD3	
LIMK2	KLHL36	ZNF121	ATP6V0D1	RAP1GAP	
TSC22D3	VSIG10L	LINC01133	ARRDC1	TMEM79	
GMFB	HMGCR		DENND1A	KRT8	
MLST8	CLK3		KLHL36	ELOVL1	
znf32	RNH1		RAB3IL1	TMEM125	
C1QTNF3	YIF1B		ACER3		
UBE2D3	PAK1IP1		OSBPL1A		
MAT2A	ALDH1A3		TRIM16		
CBWD2	TRABD		VSIG10L		
ZNF678	LIMCH1		SEPT5		
MOCOS	UBA1		RDH13		
	FDFT1		GPRIN2		
	GREB1		CLK3		
	NCAPD3		RNH1		
	SLC36A4		ZFAND6		
	KLC2		CDIP1		
	RAP1GAP		YIF1B		
	TMEM79		LIMK2		
	NR4A1		TSC22D3		
	KRT8		TRABD		
	ELOVL1		LIMCH1		
	RCAN1		GMFB		
	CNNM2		MLST8		
	TMEM125		TLE3		
	NDUFV3		UBA1		
	SPATC1L		TNRC6B		
	RBM45		SLC36A4		
	MIPEP		KLC2		
	BBS4		NR4A1		
	FAM195A		znf32		
	SS18		C1QTNF3		
	RHOC		UBE2D3		
	ZNF226		RCAN1		
	TPD52		SORBS3		
			MAT2A		
			CNNM2		
			CBWD2		
			NDUFV3		
			ZNF678		
			ZNF121		
			SPATC1L		
			MOCOS		
			RBM45		
			MIPEP		
			BBS4		
			FAM195A		
			LINC01133		
			SS18		
			RHOC		
			ICAM3		
			ZNF226		

**Table 3.** Alternative events in genes previously linked to cancer.

Gene name	Function	Clinical importance and roles in other cancer types	Clinical importance and roles in prostate cancer
***TACC2*** Transforming Acidic Coiled- Coil Containing Protein 2	centrosome- and microtubule-interacting protein	Growth and prognosis of breast cancer ^56^	castration-resistant growth of prostate cancer ^57^
***LIG4***	DNA ligase with role in DNA repair	Prognostic marker in nasopharyngeal cancer ^58^ Upregulated in colorectal cancer with role in wnt signalling ^59^	Predictor of poor prognosis ^60^
***RLN1 and RLN2*** (Relaxin1 and 2)	Endocrine hormones (part of insulin gene superfamily)	Breast cancer invasiveness ^61, 62^ metastasis of human osteosarcoma ^63^ Thyroid cancer oncogenesis ^64, 65^	Well characterised role in the development and progression of prostate cancer ^5, 50– 55^.
***TPD52*** (Tumor Protein D52)	Role in proliferation and exo- and endocytic pathways	Well characterised role in numerous cancer types ^46, 66– 69^	Known AR target, overexpressed and amplified in prostate cancer ^70^ Oncogene in prostate cancer ^71^ Neuroendocrine transdifferentiation of prostate cancer ^72^ Isoform produced by alternative promoter known as PrLZ and already linked to prostate cancer ^47– 49, 73, 74^
***FDFT1*** (Farnesyl-Diphosphate Farnesyltransferase 1)	squalene synthase	Role in lung cancer metastasis ^75^	Linked to prostate cancer risk and aggressiveness ^76^
***TLE3*** (Transducin Like Enhancer Of Split 3)	Negative regulator of Wnt/β- catenin signaling	Predictive marker for response to therapy in ovarian and breast cancer ^77, 78^ Represses colon cancer proliferation ^79^	Upregulated in prostate tumours ^80^ and linked to wnt signalling in castrate resistant disease ^81^
***CNNM2*** (Cyclin & CBS Domain Divalent Metal Cation Transport Mediator 2)	Magnesium transporter	Proposed oncogenic role via increasing magnesium uptake ^82^	Unknown
***NUP93***	Nucleoporin protein – role in apoptosis	Driver mutation linked to breast cancer ^83^	Unknown
***MAT2A*** Methionine adenosyltransferase II	Biosynthesis of S-adenosylmethionine, the principal biological methyl donor and precursor of polyamines and glutathione.	Upregulated in liver and colon cancer, potential drug target ^84, 85^ Tumour suppressor in kidney carcinogenesis ^86^ Role in other cancer types ^87^	Upregulated in prostate cancer and linked to cell migration via miR-34a and miR- 34b ^87, 88^
***PIK3R1***	PI3K regulatory subunit	Underexpressed in breast cancer ^89^ High mutation frequency in endometrial cancer ^90^	Controlled by androgens and repressed in prostate cancer cells ^21^
***SNAPC2*** (Small Nuclear RNA Activating Complex Polypeptide 2)	Subunit of the snRNA- activating protein complex. Necessary for RNA polymerase II and III dependent small-nuclear RNA gene transcription	Epigenetic silencing is prognostic in glioblastoma ^91^	Unknown
***ZNF678*** (Zinc Finger Protein 678)	Potential role in transcriptional regulation	Unknown	Unknown
***NDUFV3*** (NADH:Ubiquinone Oxidoreductase Subunit V3)	Subunit of part of the mitochondrial respiratory chain	Unknown	Androgen regulated alternative splice isoform previously identified by our exon array study ^10^
***OSBPL1A*** (Oxysterol Binding Protein Like 1A)	Intracellular lipid receptor	Alternative promoter use in colorectal cancer ^92^	Unknown
***RDH13*** (Retinol Dehydrogenase 13)	Role in retinoic acid production and protection against oxidative stress	Unknown	Unknown
***ZNF121*** (Zinc Finger Protein 121)	Potential role in transcriptional regulation	Interacts with MYC. Upregulated in breast cancer ^93^	Unknown
***SLC36A4.1*** (Solute Carrier Family 36 Member 4)	amino acid transporter	Unknown	Unknown
***RCAN1*** (Regulator of Calcineurin 1)	Inhibits calcineurin- dependent signaling pathways	Inhibits NF-κB and suppresses lymphoma growth in mice ^94^. Role in cancer cell migration ^95^	Unknown
***DCXR*** (Dicarbonyl & l-xylulose reductase)	Role in the uronate cycle of glucose metabolism	Low expression indicates poor prognosis for hepatocellular carcinoma ^96^. Role in cell adhesion ^97, 98^	Upregulated and potential biomarker in prostate cancer ^99^
***NDUFAF4*** (NADH:Ubiquinone Oxidoreductase Complex Assembly Factor 4)	Role in the mitochondrial respiratory chain	Unknown	Unknown
***MAPRE2*** (Microtubule Associated Protein RP/EB Family Member 2)	Microtubule-associated protein that is necessary for spindle symmetry during mitosis	Role in the invasion of pancreatic cancer cells ^100^	Unknown
***PEX10*** (Peroxisomal Biogenesis Factor 10)	Involved in import of peroxisomal matrix proteins	Unknown	Unknown
***AP2S1*** (Adaptor Related Protein Complex 2 Sigma 1 Subunit)	Function in protein transport across membranes	Unknown	Unknown
***LINC01133*** (long non-coding RNA)	Long non-coding RNA	Poor prognosis in colorectal cancer ^101^ Upregulated and linked to poor prognosis in lung cancer ^102^	Unknown
***ZNF226*** (Zinc Finger Protein 226)	Potential role in transcriptional regulation	Unknown	Unknown
***CDIP1*** (Cell death inducing p53 target 1)	p53 apoptotic effector Regulates TNF-alpha- mediated apoptosis	sensitivity to TNFα- induced apoptosis in cancer cells ^103^	Unknown

**Figure 1.**
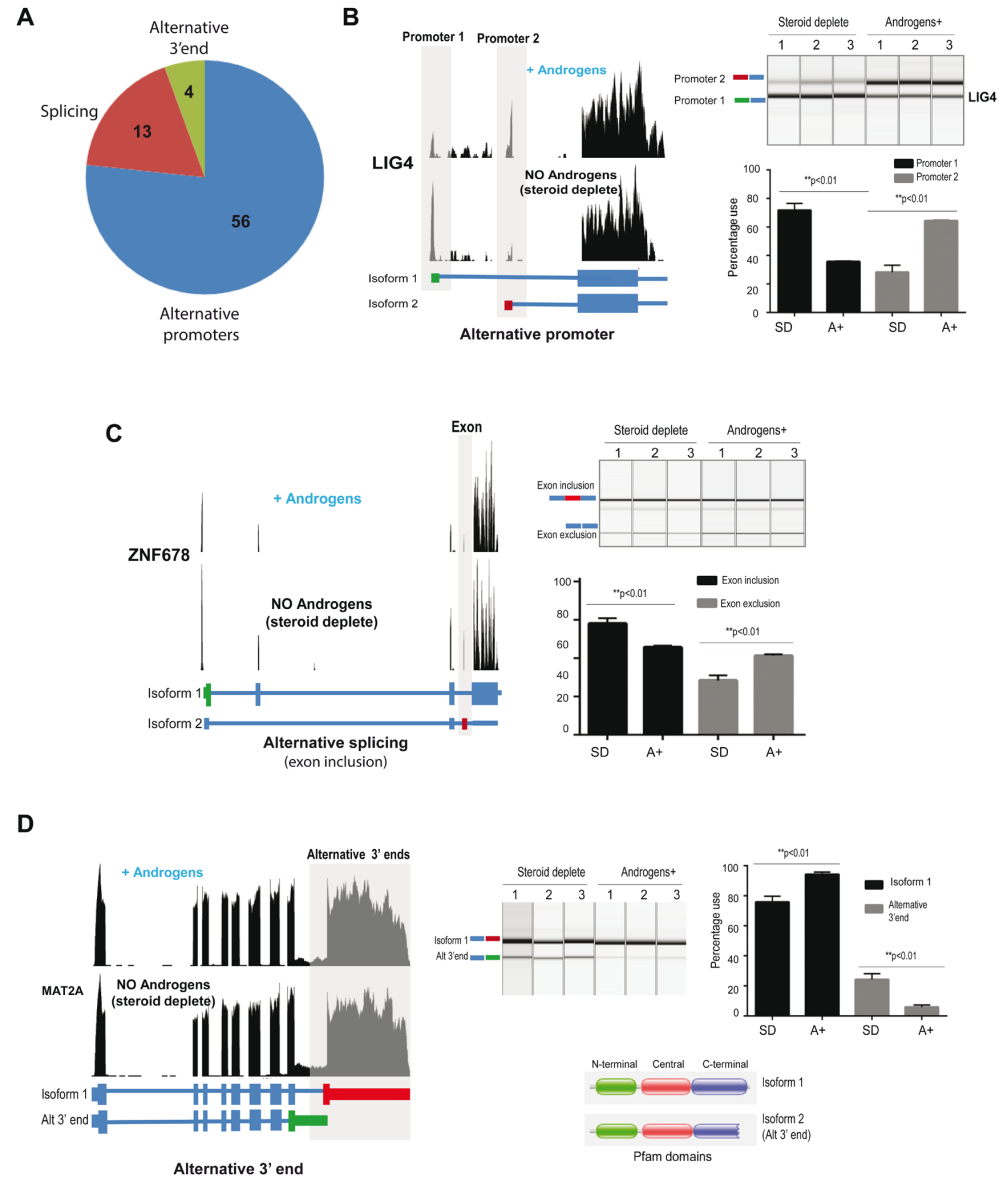
Global identification of androgen-dependent mRNA isoform production in prostate cancer cells predicts a major role for alternative promoter utilisation. (A) Analysis of RNAseq data from LNCaP cells grown with (A+) or without androgens (R1881) (steroid deplete, SD) for 24 hours identified 73 androgen regulated alternative mRNA isoforms. The 73 alternative events were generated via androgen-regulated utilisation of 56 alternative promoters, 4 alternative 3' ends and 13 alternative splicing events. (B) Androgens drive a promoter switch in the *LIG4* gene, which produces an mRNA isoform with an alternative 5’UTR. Visualisation of our LNCaP cell RNA-seq reads for the *LIG4* gene on the UCSC genome browser identified a switch from promoter 1 to alternative promoter 2 in cells grown in the presence of androgens. Promoter 2 is predicted to produce a different 5’UTR without influencing the protein sequence (left panel). Quantitative PCR using primers specific to each promoter indicate that in response to androgens there is repression of promoter 1 and induction of promoter 2 (right panel). (C) Androgens drive alternative splicing of the *ZNF678* gene. Visualisation of our LNCaP cell RNA-seq reads for the *ZNF678* gene on the UCSC genome browser identified a switch to inclusion of a cassette exon in the presence of androgens. Inclusion of the alternative cassette exon in the *ZNF678* gene is predicted to induce a switch to an alternative non-coding mRNA isoform (left panel). Quantitative PCR using primers in flanking exons confirmed increased inclusion of the alternative exon in LNCaP cells exposed to androgens (right panel). (D) Androgens promote selection of an alternative 3’ end for the *MAT2A* gene. Visualisation of our LNCaP cell RNA-seq reads for the *MAT2A* gene on the UCSC genome browser indicates a switch to reduced usage of an alternative 3’ end in the presence of androgens (left panel). Quantitative PCR using primers specific to each isoform confirmed down-regulation of an alternative 3’ end (p<0.01). Alternative 3’ ends for the *MAT2A* gene are predicted to produce proteins with different amino acid sequences and to influence a known Pfam domain (right panel).

The 73 identified mRNA isoforms were generated via androgen-regulated utilisation of 56 alternative promoters, 4 alternative 3′ ends and 13 alternative splicing events ( Figure 1A). Of the 56 androgen regulated alternative promoters that were identified, 23 alternative promoters were induced by androgens (including *LIG4*, Figure 1B), 26 promoters were repressed by androgens, and for 7 genes there was a switch in usage from one promoter to another ( Table 1). The alternative splicing events that were under androgen control included 12 alternative exons and one androgen-regulated intron retention ( Table 1). 10 of these are novel to this study, including exclusion of an alternative exon in *ZNF678* ( Figure 1C). Of the alternative exons, six genes contained switches in previously unannotated protein-coding exons in response to androgen-exposure. We also identified four androgen regulated alternative mRNA 3' end isoform switches, including a switch in the 3’ end of the mRNA transcript for the *MAT2A* gene ( Figure 1D).

### Androgen regulated events control the production of alternative protein isoforms, non-coding RNAs and alternative 5' UTRs

**Figure 2.**
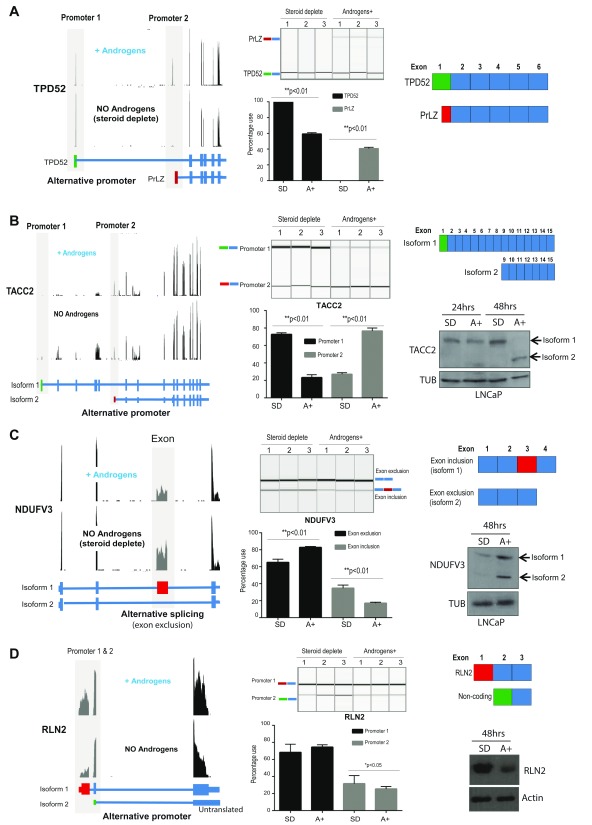
Androgen regulated mRNA isoform switches control alternative protein isoforms and non-coding RNAs. ( **A**) Androgens induce an alternative promoter in the oncogene *TPD52* that produces an isoform called PrLZ. Visualisation of our LNCaP cell RNA-seq reads for the *TPD52* gene on the UCSC genome browser identified a switch from promoter 1 to alternative promoter 2 in cells grown in the presence of androgens. Promoter 2 is known to produce an alternative protein isoform of TPD52 known as PrLZ (left panel). Quantitative PCR using primers specific to each promoter indicate an induction of the PrLZ isoform in response to androgens (middle panel). PrLZ has an alternative N-terminal amino acid sequence which results in an alternative protein isoform and disrupts a known Pfam domain (right panel). ( **B**) Androgens induce an alternative promoter in the TACC2 gene that produces a novel alternative protein isoform. Visualisation of our LNCaP cell RNA-seq reads for the *TACC2* gene on the UCSC genome browser identified a switch from promoter 1 to alternative promoter 2 in cells grown in the presence of androgens. Promoter 2 is predicted to produce an alternative shorter protein isoform of TACC2 (isoform 2) (left panel). Quantitative PCR using primers specific to each promoter indicate a switch from isoform 1 to isoform 2 in response to androgens (middle panel). Detection of TACC2 protein in LNCaP by western blotting (cells were grown with or without androgens for 24 or 48 hours). Tubulin was used as a loading control. Exposure to androgens for 48 hours induces expression of the alternative TACC2 protein isoform (right panel). ( **C**) Androgens drive alternative splicing of the *NDUFV3* gene. Visualisation of our LNCaP cell RNA-seq reads for the *NDUFV3* gene on the UCSC genome browser identified a switch to exclusion of a cassette exon in the presence of androgens (left panel). Quantitative PCR using primers in flanking exons confirmed less inclusion of the alternative exon in LNCaP cells exposed to androgens (middle panel). Exclusion of the alternative cassette exon is predicted to produce an alternative protein isoform. Detection of NDUFV3 protein in LNCaP cells using western blotting (right panel). ( **D**) Androgens suppress an alternative promoter in the *RLN2* gene, which produces a shorter non-coding mRNA isoform. Visualisation of our LNCaP cell RNA-seq reads for the *RLN2* gene on the UCSC genome browser identified a switch from promoter 1 to alternative promoter 2 in cells grown in the presence of androgens. Promoter 2 is predicted to produce an untranslated non-coding mRNA isoform (left panel). Quantitative PCR using primers specific to each promoter indicated a significant switch in promoter usage in response to androgens (middle panel). Detection of RLN2 protein in LNCaP by western blotting (cells were grown with or without androgens for 48 hours). Actin was used as a loading control. As seen previously ^55^, androgens suppress RLN2 protein levels.

48/73 (66%) of the androgen regulated alternative events detected in response to androgen stimulation are predicted to change the amino acid sequence of the resulting protein ( Table 1). Some of these are already known to have a well characterised role in prostate cancer progression, including an alternative promoter in the oncogene *TPD52* that produces a protein isoform called PrLZ ( Figure 2A) ^46– 49^. Others are not so well characterised. Using western blotting we could detect a novel shorter protein isoform corresponding to androgen-driven selection of an alternative promoter in the *TACC2* gene ( Figure 2B); and exclusion of a cassette exon in the *NDUFV3* gene, which we show also produces a novel shorter protein isoform ( Figure 2C). We also detected a switch in the 3' end of the mRNA transcript for the *MAT2A* gene, which is predicted to produce a protein isoform with a shorter C-terminal domain ( Figure 1D); and induction of an alternative 3' isoform of *CNNM2,* which is predicted to be missing a conserved CBS domain ( Table 1 and Supplementary Figure 1).

11 of the remaining identified androgen-regulated alternative events change the expression of mRNAs from coding to non-coding or untranslated (not predicted to produce a protein) ( Table 1). These included promoter switches for the *RLN1* and *RLN2* genes which encode peptide hormones that may be important in prostate cancer ^5, 50– 55^. Androgens drive a promoter switch in both *RLN1* and *RLN2* to produce predicted non-coding or untranslated mRNA isoforms, reducing expression of protein-coding *RLN1* and *RLN2* mRNA isoforms. To test whether prostate cancer cells turn off gene expression by switching between utilisation of promoters that generate coding and noncoding mRNAs, we analysed RLN2 protein levels. Consistent with our hypothesis and a previous study ^55^, RLN2 protein production was negatively regulated by androgens in parallel to the switch to the non-coding mRNA isoform ( Figure 2D).

14 of the identified androgen-dependent mRNA isoforms lead to/result in coding mRNAs with altered 5’ untranslated regions (5′ UTR) with no impact on the coding sequence. These include a promoter switch in the *LIG4* gene ( Figure 1B).

### Differential expression of androgen-dependent mRNA isoforms in prostate adenocarcinoma versus normal tissue

To investigate potential links between androgen-dependent mRNA isoforms and tumourigenesis, we analysed the expression of 41 androgen-regulated mRNA isoform pairs in clinical prostate adenocarcinoma and normal prostate tissues. This analysis utilised transcriptomic data from 497 tumour samples and 52 normal samples in the PRAD TCGA cohort ^104^. The remaining isoform pairs identified within our dataset have not been previously annotated by UCSC, therefore it was not possible to include them in our comparison. A description of the cohort used is summarised in Table 4.

**Table 4.** Description of the TCGA PRAD cohort.

Features	Total Cases
Cohort	497 patients
Tumour	497
Normal	52 (w/tumour matched sample available)
Gleason grade	
6	50
7	287
8	67
9	140
10	4
Tumour stage	
T2a	14
T2b	10
T2c	192
T3a	173
T3b	140
T4	12
Gleason grade (alternative gleason grade grouping)	
1 (primary + secondary score ≤ 6)	50
2 (3 + 4)	171
3 (4 + 3)	123
4 (4 + 4)	93
5 (primary + secondary score ≥ 9)	111

All tumours were hormone naive (not subject to ADT) at the time of sample collection

**Figure 3.**
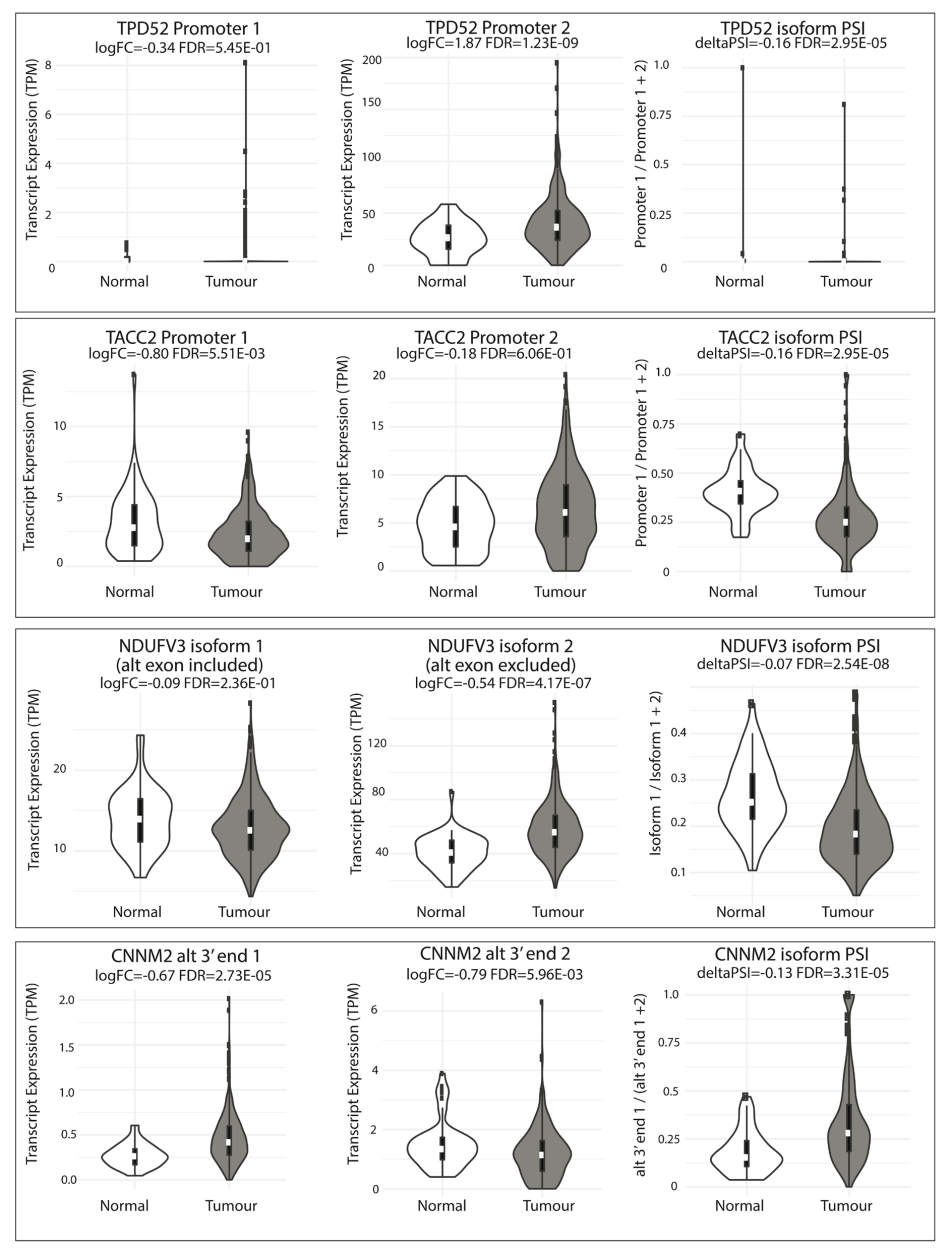
Differential expression of androgen dependent mRNA isoforms in prostate cancer versus normal tissue within the PRAD TCGA cohort for *TPD52*, *TACC2*, *NDUFV3* and *CNNM2*. Violin-boxplots of expression in transcripts per million mapped reads (TPM) of Isoforms 1 (left panel) and 2 (central panel), and of their expression ratio in PSI (right panel) in normal and tumour samples. The mean log2 fold-change (logFC) in expression between tumour and normal samples and the associated FDR-adjusted p-value for the moderated t-statistic of differential expression are shown for both isoforms (left and central panels). The mean difference in PSI (deltaPSI) between tumour and normal samples and the associated FDR-adjusted p-value for the Mann-Whitney U test of differential splicing are shown (right panel).

33 of the 42 mRNA isoform pairs exhibited significant differences in the expression of at least one of the isoforms, or in the isoform expression ratio between tumour and normal tissues ( Table 5). 13 of those tumour-specific alterations mimicked the effect of androgen stimulation in LNCaP cells: the changes were in form of alternative promoters for *TACC2*, *TPD52*, *NUP93*, *PIK3R1*, *RDH13*, *ZFAND6*, *CDIP1*, *YIF1B*, *LIMK2*, and *FDFT1*; an alternative 3´ end in *CNNM2*; and alternative exons in *NDUFV3* and *SS18* ( Figure 3, Table 5 & Supplementary Figure 2). Two of the alternative promoters ( *ZFAND6* and *CDIP1*) are predicted to introduce a change in the 5′UTR, whereas all the others are predicted to alter the resulting protein isoform. A number of mRNA isoforms that were androgen responsive in LNCaP cells showed tumour specific alterations opposite to the effect of androgen stimulation. These were *LIG4*, *MAPRE2*, *OSBPL1A*, *SEPT5*, *NR4A1*, and *RCAN1 (*all predicted to alter the resulting protein isoform except *LIG4*). For the remaining 14 mRNA isoform pairs, the data was inconclusive according to the consistency conditions listed in the methods section ( Table 5).

**Table 5.** Summarised results of the differential expression analysis of androgen-regulated isoforms between tumour and normal tissue samples in the TCGA PRAD cohort.

			Isoform 1			Isoform 2			PSI			
Gene	Event type	Change with androgens (LNCap)	log2FC	Av.Expr. (TPM)	FDR	log2FC	Av.Expr. (TPM)	FDR	Delta PSI	Av. PSI	FDR	Consistency of change in tumours
LIG4	Alternative promoter	Induction of promoter 2	**-0.81**	**1.77**	**4.31E-02**	**-1.53**	**1.28**	**4.48E-05**	**0.06**	**0.597300667**	**9.85E-02**	Opposite
TACC2	Alternative promoter	Repression of promoter 1	**-0.80**	**2.42**	**5.51E-03**	0.18	6.22	6.06E-01	**-0.16**	**0.284239843**	**2.95E-05**	**Consistent**
TPD52	Alternative promoter	Induction of promoter 2	-0.34	0.17	5.45E-01	**1.87**	**39.20**	**1.23E-09**	0.00	0.011365308	8.11E-06	**Consistent**
NUP93	Alternative promoter	Induction of promoter 1	**0.25**	**25.52**	**6.45E-04**	0.31	7.20	6.08E-01	0.01	0.828738669	7.52E-01	**Consistent**
RLN1	Alternative promoter	Repression of promoter 2	-0.45	133.50	4.97E-01	--	--	--	--	--	--	Not assessed
AP2S1	Alternative promoter	Induction of promoter 2	0.48	191.44	2.24E-05	--	--	--	--	--	--	Not assessed
RLN2	Alternative promoter	Induction of promoter 1	0.48	5.07	2.41E-01	--	--	--	--	--	--	Not assessed
PIK3R1	Alternative promoter	Repression of promoter 2	**-1.79**	**7.15**	**3.26E-12**	**-1.79**	**1.26**	**8.20E-06**	-0.02	0.820282185	7.52E-01	**Consistent**
MAPRE2	Alternative promoter	Switch to promoter 2	1.17	1.52	1.22E-01	-0.34	0.07	1.96E-01	**0.09**	**0.730349729**	**4.67E-02**	Opposite
NDUFAF4	Alternative promoter	Repression of promoter 2	0.55	0.06	5.86E-02	--	--	--	--	--	--	Not assessed
DCXR	Alternative promoter	Repression of promoter 2	0.68	623.07	2.05E-05	--	--	--	--	--	--	Not assessed
PEX10	Alternative promoter	Switch to promoter 2	0.92	75.55	7.84E-06	--	--	--	--	--	--	Not assessed
SNAPC2	Alternative promoter	Switch to promoter 2	0.38	5.42	1.23E-01	**0.22**	**37.58**	**3.20E-02**	-0.01	0.130583106	8.29E-01	Inconclusive
ATP6V0D1	Alternative promoter	Repression of promoter 2	-0.12	109.86	1.42E-01	--	--	--	--	--	--	Not assessed
ARRDC1	Alternative promoter	Induction of promoter 2	0.46	12.78	2.34E-05	--	--	--	--	--	--	Not assessed
DENND1A	Alternative promoter	Repression of promoter 2	0.04	7.09	9.11E-01	--	--	--	--	--	--	Not assessed
KLHL36	Alternative promoter	Induction of promoter 2	-0.38	10.58	4.61E-06	--	--	--	--	--	--	Not assessed
RAB3IL1	Alternative promoter	Repression of promoter 2	0.34	0.28	5.07E-01	0.05	4.68	6.91E-01	0.01	0.062673984	4.28E-01	Inconclusive
ACER3	Alternative promoter	Repression of promoter 2	0.13	6.32	8.52E-01	--	--	--	--	--	--	Not assessed
OSBPL1A	Alternative promoter	Induction of promoter 2	0.14	4.11	5.75E-01	**-1.06**	**3.56**	**3.44E-09**	**0.17**	**0.522207286**	**1.03E-08**	Opposite
TRIM16	Alternative promoter	Induction of promoter 2	-0.65	6.87	1.03E-14	--	--	--	--	--	--	Not assessed
VSIG10L	Alternative promoter	Induction of promoter 1	-1.01	1.91	5.49E-04	--	--	--	--	--	--	Not assessed
SEPT5	Alternative promoter	Repression of promoter 2	**0.80**	**11.47**	**1.79E-09**	**1.09**	**3.86**	**1.82E-06**	-0.03	0.749615358	1.90E-01	Opposite
HMGCR	Alternative promoter	Repression of promoter 1	-0.86	0.59	1.07E-01	**-0.55**	**17.41**	**1.09E-02**	0.00	0.029105295	9.62E-01	Inconclusive
RDH13	Alternative promoter	Induction of promoter 1	**1.67**	**2.10**	**1.31E-08**	**0.72**	**0.05**	**5.88E-03**	0.00	0.962155441	9.33E-02	**Consistent**
GPRIN2	Alternative promoter	Repression of promoter 2	--	--	--	-0.48	3.31	3.98E-02	--	--	--	Not assessed
CLK3	Alternative promoter	Repression of promoter 1	0.10	31.34	1.07E-01	--	0.04	--	0.00	0.998537929	6.18E-01	Inconclusive
RNH1	Alternative promoter	Induction of promoter 1	-0.16	4.38	7.95E-01	-0.19	6.56	5.74E-01	0.00	0.375368151	7.52E-01	Inconclusive
ZFAND6	Alternative promoter	Repression of promoter 2	-0.10	37.63	6.33E-01	**-1.51**	**2.29**	**5.59E-03**	**0.03**	**0.935657481**	**3.73E-02**	**Consistent**
CDIP1	Alternative promoter	Repression of promoter 2	0.77	0.35	1.16E-01	**-1.83**	**3.70**	**2.77E-11**	**0.06**	**0.142411928**	**1.46E-03**	**Consistent**
YIF1B	Alternative promoter	Switch to promoter 2	0.50	2.52	3.18E-01	**2.83**	**3.08**	**1.60E-04**	**-0.32**	**0.497841217**	**1.64E-02**	**Consistent**
LIMK2	Alternative promoter	Switch to promoter 2	**-0.90**	**6.80**	**1.50E-03**	**0.58**	**10.99**	**1.10E-05**	**-0.19**	**0.382613244**	**2.85E-06**	**Consistent**
TSC22D3	Alternative promoter	Repression of promoter 1	--	35.48	--	**-1.08**	**173.59**	**8.13E-15**	0.01	0.203019277	2.97E-01	Inconclusive
ALDH1A3	Alternative promoter	Repression of promoter 1	0.71	279.09	7.51E-03	--	--	--	--	--	--	Not assessed
TRABD	Alternative promoter	Switch to promoter 2	**1.57**	**21.80**	**3.42E-02**	0.87	0.54	1.18E-01	0.00	0.958501941	5.17E-01	Inconclusive
LIMCH1	Alternative promoter	Repression of promoter 2	--	0.01	--	--	--	--	--	--	--	Not assessed
GMFB	Alternative promoter	Induction of promoter 2	-0.11	11.91	7.54E-01	--	--	--	--	--	--	Not assessed
MLST8	Alternative promoter	Switch to promoter 1	**0.87**	**0.19**	**9.88E-04**	**1.51**	**4.90**	**9.60E-03**	0.02	0.121241399	5.81E-01	Inconclusive
TLE3	Alternative promoter	Induction of promoter 2	0.10	0.10	8.70E-01	-0.20	5.14	4.28E-01	0.00	0.02562604	6.14E-01	Inconclusive
UBA1	Alternative promoter	Repression of promoter 1	0.21	23.51	1.39E-01	0.01	131.71	9.46E-01	0.01	0.190009964	2.99E-01	Inconclusive
TNRC6B	Alternative promoter	Repression of promoter 2	**0.18**	**2.27**	**3.34E-02**	-0.43	0.03	4.15E-01	0.00	0.988593061	3.56E-02	Inconclusive
FDFT1	Alternative promoter	Repression of promoter 2	**-0.57**	**94.14**	**1.13E-07**	**-1.07**	**1.05**	**5.62E-12**	0.00	0.986642757	2.13E-02	**Consistent**
GREB1	Alternative promoter	Induction of promoter 2	**1.45**	**1.01**	**6.45E-04**	0.28	1.48	3.21E-01	**0.14**	**0.378280864**	**3.40E-02**	Inconclusive
NCAPD3	Alternative promoter	Induction of promoter 2	0.16	75.75	6.55E-01	--	--	--	--	--	--	Not assessed
SLC36A4	Alternative promoter	Induction of promoter 2	-0.91	2.15	1.60E-03	--	--	--	--	--	--	Not assessed
KLC2	Alternative promoter	Repression of promoter 1	0.47	0.27	4.16E-01	-0.76	3.64	8.12E-02	0.00	0.1048405	4.53E-01	Inconclusive
RAP1GAP	Alternative promoter	Repression of promoter 1	1.94	3.42	3.45E-08	--	--	--	--	--	--	Not assessed
TMEM79	Alternative promoter	Repression of promoter 1	0.21	3.77	7.91E-01	**-1.40**	**1.67**	**2.05E-05**	0.19	0.399443544	5.07E-02	Inconclusive
NR4A1	Alternative promoter	Induction of promoter 2	-0.40	1.86	2.34E-01	**-0.74**	**5.81**	**7.87E-03**	0.06	0.292753045	2.53E-01	Opposite
ZNF32	Alternative promoter	Repression of promoter 2	0.03	67.26	7.14E-01	0.03	4.12	7.14E-01	0.00	0.942446541	1.00E+00	Inconclusive
C1QTNF3	Alternative promoter	Induction of promoter 1	-0.30	3.41	4.67E-01	--	--	--	--	--	--	Not assessed
UBE2D3	Alternative promoter	Switch to promoter 2	**-0.50**	**8.00**	**5.09E-04**	-0.13	0.32	8.18E-01	-0.01	0.953413055	5.49E-01	Inconclusive
KRT8	Alternative promoter	Repression of promoter 1	-0.08	2.08	8.55E-01	**0.48**	**697.27**	**1.26E-05**	0.00	0.003455479	9.85E-02	Inconclusive
ELOVL1	Alternative promoter	Induction of promoter 2	-0.10	100.07	1.38E-01	--	--	--	--	--	--	Not assessed
RCAN1	Alternative promoter	Induction of promoter 2	-0.31	1.39	4.66E-01	**-1.40**	**6.90**	**4.40E-07**	**0.09**	**0.2372612**	**1.64E-02**	Opposite
SORBS3	Alternative promoter	Induction of promoter 2	0.21	6.33	6.20E-01	--	--	--	--	--	--	Not assessed
MAT2A	Alternative 3' end	Repression of isoform 2	-0.36	102.47	6.63E-02	0.27	13.41	2.87E-01	**-0.03**	**0.888519015**	**5.32E-03**	Inconclusive
CNNM2	Alternative 3' end	Induction of isoform 1	**0.67**	**0.44**	**2.73E-05**	**-0.79**	**1.22**	**5.96E-03**	**0.13**	**0.331082656**	**3.31E-05**	**Consistent**
TMEM125	Alternative 3' end	Induction of isoform 1	--	--	--	0.45	40.70	9.40E-04	--	--	--	Not assessed
CBWD2	Alternative 3' end	Induction of isoform 2	0.00	16.56	9.88E-01	--	--	--	--	--	--	Not assessed
NDUFV3	Alternative exon	Switch to isoform 2 (exon excluded)	-0.09	12.98	2.36E-01	**0.54**	**56.19**	**4.17E-07**	**-0.07**	**0.201011**	**2.54E-08**	**Consistent**
ZNF678	Alternative exon	Switch to isoform 2 (exon excluded)	0.32	0.97	2.23E-01	--	--	--	--	--	--	Not assessed
ZNF121	Alternative exon	Switch to isoform 2 (exon excluded)	**0.90**	**0.08**	**5.97E-03**	0.02	3.09	9.28E-01	0.00	0.037899858	9.85E-02	Inconclusive
SPATC1L	Alternative exon	Induction of isoform 2 (exon included)	0.35	36.98	4.71E-02	--	--	--	--	--	--	Not assessed
MOCOS	Alternative exon	Switch to isoform 2 (exon excluded)	-0.82	2.24	1.14E-09	--	--	--	--	--	--	Not assessed
RBM45	Alternative exon	Switch to isoform 2 (exon included)	0.25	7.85	9.96E-07	--	--	--	--	--	--	Not assessed
MIPEP	Alternative exon	Repression of isoform 2 (exon excluded)	0.87	49.00	9.53E-04	--	--	--	--	--	--	Not assessed
BBS4	Alternative exon	Induction of isoform 2 (exon included)	0.02	21.63	9.71E-01	--	--	--	--	--	--	Not assessed
FAM195A	Alternative exon	Switch to isoform 1 (exon exluded)	**0.87**	**43.81**	**4.03E-08**	**0.99**	**5.57**	**1.01E-08**	-0.01	0.884563881	2.50E-01	Inconclusive
LINC01133	Alternative exon	Induction of isoform 1 (exon excluded)	--	--	--	-1.58	2.77	1.39E-08	0.00	--	--	Not assessed
SS18	Alternative exon	Switch to isoform 2 (exon excluded)	-1.47	3.70	1.97E-02	-0.14	33.31	1.18E-02	**-0.07**	**0.087763421**	**2.88E-02**	**Consistent**
RHOC	Alternative exon	Switch to isoform 2 (exon excluded)	**0.62**	**1.48**	**3.71E-06**	0.13	153.20	1.96E-01	0.00	0.009830219	1.46E-03	Inconclusive
ZNF226	Retained intron	Switch to isoform 1 (intron included)	-0.13	2.48	5.37E-01	-0.08	13.49	7.40E-01	-0.01	0.184522223	8.77E-01	Inconclusive

### Changes in androgen-dependent mRNA isoform expression during tumour progression

**Figure 4.**
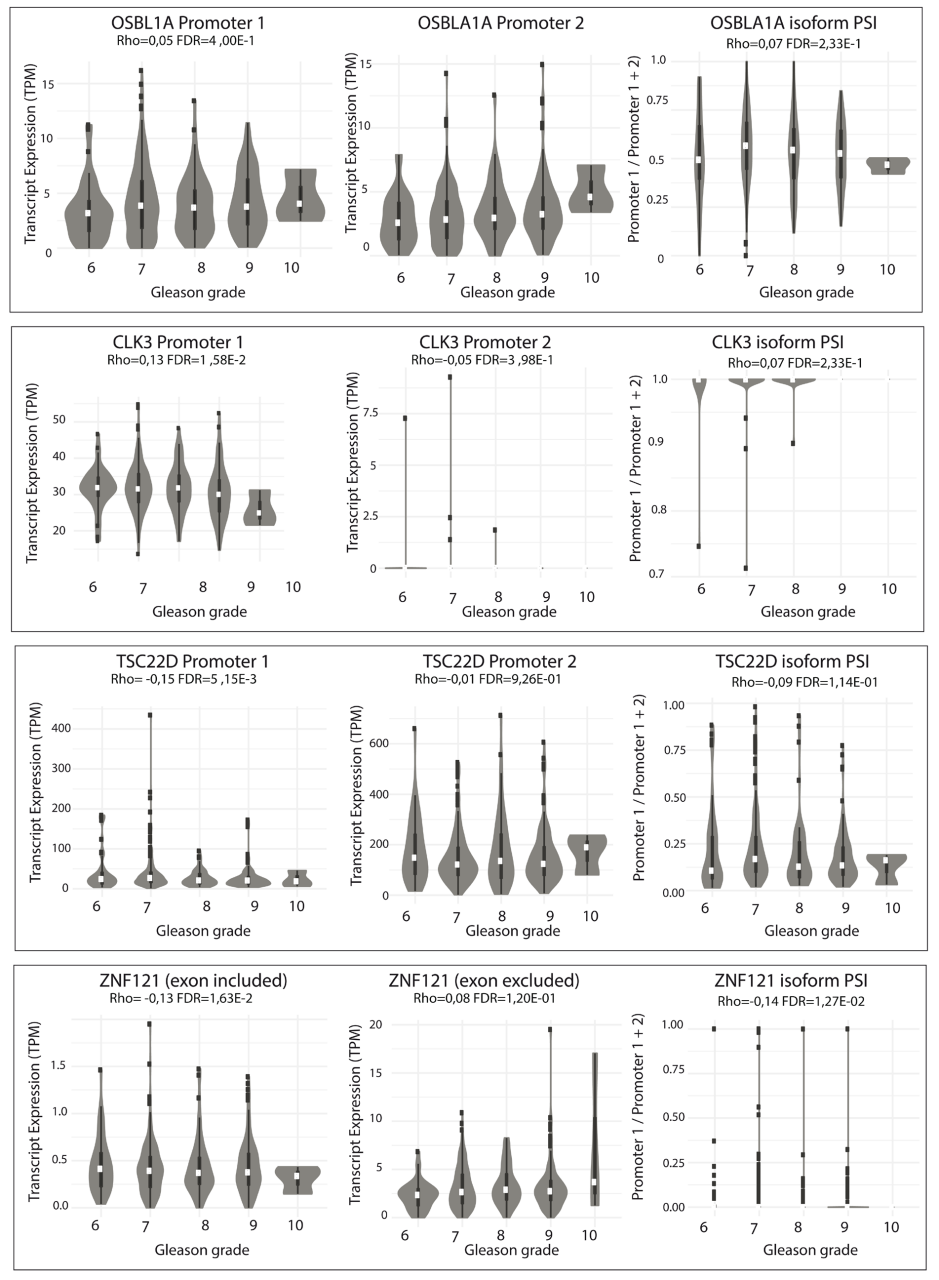
Differential alternative mRNA isoform expression in the TGCA PRAD cohort across different Gleason grades for *OSBPL1A*, *CLK3*, *TSC22D* and *ZNF121*. Violin-boxplots of expression in transcripts per million mapped reads (TPM) of Isoforms 1 (left panel) and 2 (central panel), and of their expression ratio (right panel) by Gleason grade. Their respective Spearman’s correlation coefficient (Rho) with grade and associated FDR-adjusted p-value are shown.

**Figure 5.**
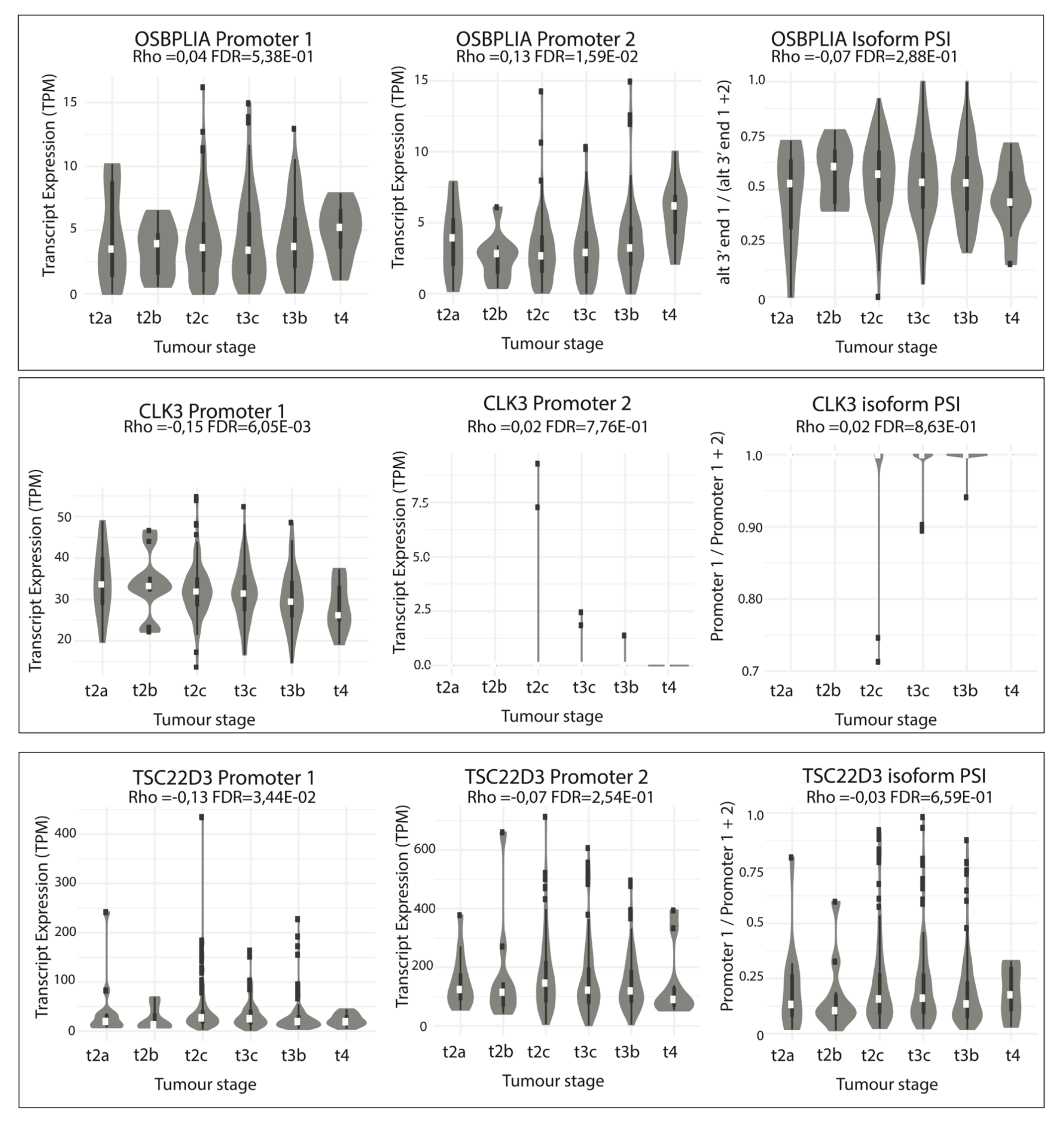
Differential alternative mRNA isoform expression in the TGCA PRAD cohort across different tumour stages for *OSBPL1A*, *CLK3* and *TSC22D3*. Violin-boxplots of expression in transcripts per million mapped reads (TPM) of Isoforms 1 (left panel) and 2 (central panel), and of their expression ratio (right panel) by tumour stage. Their respective Spearman’s correlation coefficient (Rho) with stage and associated FDR-adjusted p-value are shown.

We next investigated whether the identified androgen-dependent mRNA isoforms are differentially expressed during prostate cancer progression by correlating the expression levels of each isoform with Gleason scores and prostate tumour grades within the PRAD TCGA cohort ( Figure 4 & Figure 5, Table 6 & Table 7 and Supplementary Figure 3 & Supplementary Figure 4). For 6 of the alternative mRNA isoforms responsive to androgens (made from alternative promoters in *LIG4, OSBPL1A, CLK3, TSC22D3 & ZNF32* and utilising an alternative exon in *ZNF121*), the expression changed significantly with Gleason score and showed specific alterations consistent with the effect of androgen stimulation. Conversely, 9 alternative isoforms (which were androgen responsive in LNCaP cells) showed tumour specific alterations opposite to the effect of androgen stimulation (including an alternative promoters in *NUP93* and the alternative 3´end of *MAT2A)*. 3 androgen regulated mRNA isoforms ( *OSBPL1A*, *CLK3* and *TSC22D3*) change significantly with both Gleason grade and tumour stage.

**Table 6.** Summarised results of the correlation analysis of androgen-regulated isoforms expression with Gleason score in the TCGA PRAD cohort.

			Isoform 1		Isoform 2		PSI		
Gene	Event type	Change with androgens (LNCap)	Rho	FDR	Rho	FDR	Rho	FDR	Consistency of change with Gleason
LIG4	Alternative promoter	Induction of promoter 2	-0.07	1.92E-01	0.09	1.07E-01	**-0.18**	**4.21E-04**	Consistent -
TACC2	Alternative promoter	Repression of promoter 1	-0.08	1.55E-01	0.01	9.26E-01	-0.08	1.88E-01	Inconclusive
TPD52	Alternative promoter	Induction of promoter 2	0.00	9.51E-01	0.02	7.73E-01	0.00	9.46E-01	Inconclusive
NUP93	Alternative promoter	Induction of promoter 1	**-0.18**	**7.92E-04**	-0.07	1.81E-01	0.04	4.75E-01	Opposite
RLN1	Alternative promoter	Repression of promoter 2	-0.16	1.98E-03	--	--	--	--	Not assessed
AP2S1	Alternative promoter	Induction of promoter 2	-0.01	8.72E-01	--	--	--	--	Not assessed
RLN2	Alternative promoter	Induction of promoter 1	-0.10	6.03E-02	--	--	--	--	Not assessed
PIK3R1	Alternative promoter	Repression of promoter 2	-0.07	2.51E-01	0.09	1.20E-01	-0.17	1.29E-03	Inconclusive
MAPRE2	Alternative promoter	Switch to promoter 2	-0.07	1.92E-01	-0.06	2.73E-01	0.06	3.23E-01	Inconclusive
NDUFAF4	Alternative promoter	Repression of promoter 2	0.00	9.79E-01	--	--	--	--	Not assessed
DCXR	Alternative promoter	Repression of promoter 2	-0.29	4.07E-09	--	--	--	--	Not assessed
PEX10	Alternative promoter	Switch to promoter 2	0.08	1.50E-01	--	--	--	--	Not assessed
SNAPC2	Alternative promoter	Switch to promoter 2	**0.15**	**5.48E-03**	**-0.18**	**3.55E-04**	**0.21**	**5.13E-05**	Opposite
ATP6V0D1	Alternative promoter	Repression of promoter 2	-0.11	3.43E-02	--	--	--	--	Not assessed
ARRDC1	Alternative promoter	Induction of promoter 2	0.12	2.00E-02	--	--	--	--	Not assessed
DENND1A	Alternative promoter	Repression of promoter 2	-0.02	8.10E-01	--	--	--	--	Not assessed
KLHL36	Alternative promoter	Induction of promoter 2	-0.13	1.67E-02	--	--	--	--	Not assessed
RAB3IL1	Alternative promoter	Repression of promoter 2	0.06	3.17E-01	**0.32**	**9.13E-12**	-0.02	7.15E-01	Opposite
ACER3	Alternative promoter	Repression of promoter 2	0.16	3.79E-03	--	--	--	--	Not assessed
OSBPL1A	Alternative promoter	Induction of promoter 2	0.05	4.00E-01	**0.13**	**1.58E-02**	-0.07	2.33E-01	**Consistent**
TRIM16	Alternative promoter	Induction of promoter 2	0.10	6.06E-02	--	--	--	--	Not assessed
VSIG10L	Alternative promoter	Induction of promoter 1	-0.16	1.98E-03	--	--	--	--	Not assessed
SEPT5	Alternative promoter	Repression of promoter 2	0.17	1.12E-03	**0.12**	**1.93E-02**	-0.04	4.91E-01	Opposite
HMGCR	Alternative promoter	Repression of promoter 1	0.03	6.56E-01	-0.05	4.54E-01	0.07	2.33E-01	Inconclusive
RDH13	Alternative promoter	Induction of promoter 1	0.03	7.01E-01	0.08	1.20E-01	-0.10	1.00E-01	Inconclusive
GPRIN2	Alternative promoter	Repression of promoter 2	--	--	-0.01	8.93E-01	--	--	Not assessed
CLK3	Alternative promoter	Repression of promoter 1	**-0.13**	**1.58E-02**	-0.05	3.98E-01	0.07	2.33E-01	**Consistent**
RNH1	Alternative promoter	Induction of promoter 1	0.05	4.41E-01	0.07	1.83E-01	-0.01	9.23E-01	Inconclusive
ZFAND6	Alternative promoter	Repression of promoter 2	0.07	1.87E-01	0.05	3.82E-01	-0.03	6.36E-01	Inconclusive
CDIP1	Alternative promoter	Repression of promoter 2	0.02	8.10E-01	0.03	6.81E-01	-0.01	9.23E-01	Inconclusive
YIF1B	Alternative promoter	Switch to promoter 2	0.02	8.10E-01	-0.04	5.42E-01	0.05	4.39E-01	Inconclusive
LIMK2	Alternative promoter	Switch to promoter 2	-0.02	8.10E-01	-0.03	6.30E-01	0.00	9.49E-01	Inconclusive
TSC22D3	Alternative promoter	Repression of promoter 1	**-0.15**	**5.15E-03**	-0.01	9.26E-01	-0.09	1.14E-01	**Consistent**
ALDH1A3	Alternative promoter	Repression of promoter 1	-0.12	2.00E-02	--	--	--	--	Not assessed
TRABD	Alternative promoter	Switch to promoter 2	0.14	8.04E-03	-0.04	5.43E-01	0.05	4.39E-01	Inconclusive
LIMCH1	Alternative promoter	Repression of promoter 2	0.05	4.34E-01	--	--	--	--	Not assessed
GMFB	Alternative promoter	Induction of promoter 2	0.08	1.55E-01	--	--	--	--	Not assessed
MLST8	Alternative promoter	Switch to promoter 1	0.19	5.32E-04	0.19	2.05E-04	0.07	2.14E-01	Inconclusive
TLE3	Alternative promoter	Induction of promoter 2	0.05	4.28E-01	-0.10	7.19E-02	0.07	2.33E-01	Inconclusive
UBA1	Alternative promoter	Repression of promoter 1	0.09	8.99E-02	0.03	5.95E-01	0.01	8.68E-01	Inconclusive
TNRC6B	Alternative promoter	Repression of promoter 2	-0.05	4.00E-01	-0.09	1.19E-01	0.09	1.11E-01	Inconclusive
FDFT1	Alternative promoter	Repression of promoter 2	-0.02	7.41E-01	0.07	2.07E-01	-0.07	2.14E-01	Inconclusive
GREB1	Alternative promoter	Induction of promoter 2	-0.05	4.41E-01	**-0.14**	**5.45E-03**	0.04	4.60E-01	Opposite
NCAPD3	Alternative promoter	Induction of promoter 2	-0.23	3.61E-06	--	--	--	--	Not assessed
SLC36A4	Alternative promoter	Induction of promoter 2	0.12	1.88E-02	--	--	--	--	Not assessed
KLC2	Alternative promoter	Repression of promoter 1	-0.02	8.10E-01	0.13	1.58E-02	-0.04	4.60E-01	Inconclusive
RAP1GAP	Alternative promoter	Repression of promoter 1	0.01	8.79E-01	--	--	--	--	Not assessed
TMEM79	Alternative promoter	Repression of promoter 1	-0.04	4.70E-01	0.15	3.46E-03	-0.09	1.11E-01	Inconclusive
NR4A1	Alternative promoter	Induction of promoter 2	0.10	5.44E-02	0.00	9.79E-01	0.10	7.40E-02	Inconclusive
ZNF32	Alternative promoter	Repression of promoter 2	**-0.22**	**1.32E-05**	**-0.22**	**1.11E-05**	-0.09	1.31E-01	Consistent -
C1QTNF3	Alternative promoter	Induction of promoter 1	0.08	1.58E-01	--	--	--	--	Not assessed
UBE2D3	Alternative promoter	Switch to promoter 2	0.18	7.24E-04	0.08	1.27E-01	-0.02	7.15E-01	Inconclusive
KRT8	Alternative promoter	Repression of promoter 1	-0.05	3.81E-01	-0.16	2.07E-03	0.01	8.68E-01	Inconclusive
ELOVL1	Alternative promoter	Induction of promoter 2	0.18	7.24E-04	--	--	--	--	Not assessed
RCAN1	Alternative promoter	Induction of promoter 2	0.10	5.13E-02	-0.01	8.70E-01	0.12	3.69E-02	Inconclusive
SORBS3	Alternative promoter	Induction of promoter 2	0.12	2.21E-02	--	--	--	--	Not assessed
MAT2A	Alternative 3' end	Repression of isoform 2	0.04	5.39E-01	**0.27**	**3.68E-08**	**-0.33**	**8.82E-13**	Opposite
CNNM2	Alternative 3' end	Induction of isoform 1	-0.06	3.30E-01	0.03	5.87E-01	-0.08	2.04E-01	Inconclusive
TMEM125	Alternative 3' end	Induction of isoform 1	--	--	-0.19	2.05E-04	--	--	Not assessed
CBWD2	Alternative 3' end	Induction of isoform 2	0.13	1.37E-02	--	--	--	--	Not assessed
NDUFV3	Alternative exon	Switch to isoform 2 (exon excluded)	**0.14**	**8.04E-03**	-0.07	2.48E-01	**0.13**	**2.23E-02**	Opposite
ZNF678	Alternative exon	Switch to isoform 2 (exon excluded)	-0.07	1.87E-01	--	--	--	--	Not assessed
ZNF121	Alternative exon	Switch to isoform 2 (exon excluded)	**-0.13**	**1.63E-02**	0.08	1.20E-01	**-0.14**	**1.27E-02**	**Consistent**
SPATC1L	Alternative exon	Induction of isoform 2 (exon included)	-0.13	1.58E-02	--	--	--	--	Not assessed
MOCOS	Alternative exon	Switch to isoform 2 (exon excluded)	-0.01	8.72E-01	--	--	--	--	Not assessed
RBM45	Alternative exon	Switch to isoform 2 (exon included)	0.12	2.45E-02	--	--	--	--	Not assessed
MIPEP	Alternative exon	Repression of isoform 2 (exon excluded)	-0.14	9.92E-03	--	--	--	--	Not assessed
BBS4	Alternative exon	Induction of isoform 2 (exon included)	-0.08	1.87E-01	--	--	--	--	Not assessed
FAM195A	Alternative exon	Switch to isoform 1 (exon exluded)	0.04	5.43E-01	0.14	5.35E-03	**-0.18**	**4.65E-04**	Opposite
LINC01133	Alternative exon	Induction of isoform 1 (exon excluded)	--	--	-0.02	7.51E-01	--	--	Not assessed
SS18	Alternative exon	Switch to isoform 2 (exon excluded)	0.04	4.86E-01	-0.06	2.51E-01	0.07	2.33E-01	Inconclusive
RHOC	Alternative exon	Switch to isoform 2 (exon excluded)	**0.29**	**4.07E-09**	0.15	4.24E-03	**0.21**	**3.63E-05**	Opposite
ZNF226	Retained intron	Switch to isoform 1 (intron included)	0.01	8.67E-01	-0.10	7.49E-02	0.11	6.74E-02	Inconclusive

**Table 7.** Summarised results of the correlation analysis of androgen-regulated isoforms expression with tumour stage in the TCGA PRAD cohort (related to Figure 4 and Supplementary Figure 5).

			Isoform 1		Isoform 2		PSI		
Gene	Event type	Change with androgens (LNCap)	Rho	FDR	Rho	FDR	Rho	FDR	Consistency of change with stage
LIG4	Alternative promoter	Induction of promoter 2	-0.04	6.05E-01	0.02	6.82E-01	-0.09	1.82E-01	Inconclusive
TACC2	Alternative promoter	Repression of promoter 1	-0.08	1.74E-01	-0.05	4.47E-01	-0.04	5.65E-01	Inconclusive
TPD52	Alternative promoter	Induction of promoter 2	-0.02	7.85E-01	-0.02	6.82E-01	-0.02	7.95E-01	Inconclusive
NUP93	Alternative promoter	Induction of promoter 1	**-0.12**	**3.95E-02**	0.03	6.65E-01	-0.05	4.43E-01	Opposite
RLN1	Alternative promoter	Repression of promoter 2	-0.22	1.82E-05	--	--	--	--	Not assessed
AP2S1	Alternative promoter	Induction of promoter 2	-0.04	5.51E-01	--	--	--	--	Not assessed
RLN2	Alternative promoter	Induction of promoter 1	-0.16	5.68E-03	--	--	--	--	Not assessed
PIK3R1	Alternative promoter	Repression of promoter 2	-0.02	7.92E-01	0.11	5.92E-02	**-0.14**	**3.27E-02**	Opposite -
MAPRE2	Alternative promoter	Switch to promoter 2	-0.02	7.56E-01	-0.02	6.82E-01	0.03	1.00E+00	Inconclusive
NDUFAF4	Alternative promoter	Repression of promoter 2	0.08	1.89E-01	--	--	--	--	Not assessed
DCXR	Alternative promoter	Repression of promoter 2	-0.30	6.32E-10	--	--	--	--	Not assessed
PEX10	Alternative promoter	Switch to promoter 2	0.10	9.95E-02	--	--	--	--	Not assessed
SNAPC2	Alternative promoter	Switch to promoter 2	**0.13**	**2.87E-02**	**-0.23**	**5.57E-06**	**0.20**	**2.40E-04**	Opposite
ATP6V0D1	Alternative promoter	Repression of promoter 2	-0.11	5.43E-02	--	--	--	--	Not assessed
ARRDC1	Alternative promoter	Induction of promoter 2	0.08	2.06E-01	--	--	--	--	Not assessed
DENND1A	Alternative promoter	Repression of promoter 2	-0.01	8.49E-01	--	--	--	--	Not assessed
KLHL36	Alternative promoter	Induction of promoter 2	-0.10	1.04E-01	--	--	--	--	Not assessed
RAB3IL1	Alternative promoter	Repression of promoter 2	0.08	1.71E-01	**0.33**	**4.58E-12**	0.00	9.75E-01	Opposite
ACER3	Alternative promoter	Repression of promoter 2	0.16	4.77E-03	--	--	--	--	Not assessed
OSBPL1A	Alternative promoter	Induction of promoter 2	0.04	5.38E-01	**0.13**	**1.59E-02**	-0.07	2.88E-01	**Consistent**
TRIM16	Alternative promoter	Induction of promoter 2	0.06	3.95E-01	--	--	--	--	Not assessed
VSIG10L	Alternative promoter	Induction of promoter 1	-0.12	5.43E-02	--	--	--	--	Not assessed
SEPT5	Alternative promoter	Repression of promoter 2	0.11	7.96E-02	0.07	2.54E-01	-0.01	8.89E-01	Inconclusive
HMGCR	Alternative promoter	Repression of promoter 1	0.00	9.91E-01	-0.04	5.77E-01	0.04	6.25E-01	Inconclusive
RDH13	Alternative promoter	Induction of promoter 1	-0.03	7.33E-01	0.10	7.19E-02	-0.12	9.32E-02	Inconclusive
GPRIN2	Alternative promoter	Repression of promoter 2	--	--	0.03	6.48E-01	--	--	Not assessed
CLK3	Alternative promoter	Repression of promoter 1	**-0.15**	**6.05E-03**	0.02	7.76E-01	0.02	8.63E-01	**Consistent**
RNH1	Alternative promoter	Induction of promoter 1	-0.02	7.92E-01	0.10	6.12E-02	-0.08	2.28E-01	Inconclusive
ZFAND6	Alternative promoter	Repression of promoter 2	0.03	6.50E-01	0.04	5.78E-01	-0.04	6.05E-01	Inconclusive
CDIP1	Alternative promoter	Repression of promoter 2	0.10	1.04E-01	0.02	7.82E-01	0.06	3.78E-01	Inconclusive
YIF1B	Alternative promoter	Switch to promoter 2	-0.01	8.87E-01	-0.10	6.71E-02	0.06	3.97E-01	Inconclusive
LIMK2	Alternative promoter	Switch to promoter 2	0.00	9.67E-01	-0.05	4.72E-01	0.00	9.75E-01	Inconclusive
TSC22D3	Alternative promoter	Repression of promoter 1	**-0.13**	**3.44E-02**	-0.07	2.54E-01	-0.03	6.59E-01	**Consistent**
ALDH1A3	Alternative promoter	Repression of promoter 1	-0.18	7.69E-04	--	--	--	--	Not assessed
TRABD	Alternative promoter	Switch to promoter 2	0.06	3.95E-01	-0.03	6.48E-01	0.03	7.83E-01	Inconclusive
LIMCH1	Alternative promoter	Repression of promoter 2	0.02	7.85E-01	--	--	--	--	Not assessed
GMFB	Alternative promoter	Induction of promoter 2	0.07	2.57E-01	--	--	--	--	Not assessed
MLST8	Alternative promoter	Switch to promoter 1	0.10	8.19E-02	0.15	6.14E-03	0.02	7.83E-01	Inconclusive
TLE3	Alternative promoter	Induction of promoter 2	0.03	6.38E-01	**-0.11**	**3.84E-02**	0.04	5.65E-01	Opposite
UBA1	Alternative promoter	Repression of promoter 1	0.12	5.43E-02	0.00	9.72E-01	0.06	3.99E-01	Inconclusive
TNRC6B	Alternative promoter	Repression of promoter 2	-0.04	6.31E-01	-0.03	6.48E-01	0.02	7.83E-01	Inconclusive
FDFT1	Alternative promoter	Repression of promoter 2	-0.05	4.82E-01	0.04	5.46E-01	-0.08	2.28E-01	Inconclusive
GREB1	Alternative promoter	Induction of promoter 2	-0.11	7.48E-02	**-0.18**	**7.01E-04**	0.01	8.96E-01	Opposite
NCAPD3	Alternative promoter	Induction of promoter 2	-0.23	1.82E-05	--	--	--	--	Not assessed
SLC36A4	Alternative promoter	Induction of promoter 2	0.07	2.59E-01	--	--	--	--	Not assessed
KLC2	Alternative promoter	Repression of promoter 1	-0.03	6.33E-01	0.13	1.81E-02	-0.08	2.78E-01	Inconclusive
RAP1GAP	Alternative promoter	Repression of promoter 1	0.02	7.85E-01	--	--	--	--	Not assessed
TMEM79	Alternative promoter	Repression of promoter 1	-0.08	1.71E-01	0.16	1.97E-03	-0.10	1.20E-01	Inconclusive
NR4A1	Alternative promoter	Induction of promoter 2	0.01	8.49E-01	-0.06	3.69E-01	0.08	2.62E-01	Inconclusive
ZNF32	Alternative promoter	Repression of promoter 2	-0.15	6.70E-03	0.02	7.34E-01	-0.08	2.33E-01	Inconclusive
C1QTNF3	Alternative promoter	Induction of promoter 1	0.03	6.74E-01	--	--	--	--	Not assessed
UBE2D3	Alternative promoter	Switch to promoter 2	0.20	2.96E-04	0.07	2.17E-01	-0.02	7.83E-01	Inconclusive
KRT8	Alternative promoter	Repression of promoter 1	-0.04	6.05E-01	-0.24	2.72E-06	0.04	6.05E-01	Inconclusive
ELOVL1	Alternative promoter	Induction of promoter 2	0.13	2.87E-02	--	--	--	--	Not assessed
RCAN1	Alternative promoter	Induction of promoter 2	0.09	1.26E-01	-0.01	8.69E-01	0.10	1.20E-01	Inconclusive
SORBS3	Alternative promoter	Induction of promoter 2	0.11	7.96E-02	--	--	--	--	Not assessed
MAT2A	Alternative 3' end	Repression of isoform 2	0.01	9.35E-01	**0.18**	**7.83E-04**	**-0.21**	**8.42E-05**	Opposite
CNNM2	Alternative 3' end	Induction of isoform 1	0.05	3.95E-01	0.05	4.47E-01	-0.04	6.05E-01	Inconclusive
TMEM125	Alternative 3' end	Induction of isoform 1	--	--	-0.16	2.80E-03	--	--	Not assessed
CBWD2	Alternative 3' end	Induction of isoform 2	0.08	1.74E-01	--	--	--	--	Not assessed
NDUFV3	Alternative exon	Switch to isoform 2 (exon excluded)	0.11	7.48E-02	-0.05	4.72E-01	0.11	1.00E-01	Inconclusive
ZNF678	Alternative exon	Switch to isoform 2 (exon excluded)	-0.02	7.43E-01	--	--	--	--	Not assessed
ZNF121	Alternative exon	Switch to isoform 2 (exon excluded)	-0.08	1.80E-01	0.03	6.48E-01	-0.09	1.82E-01	Inconclusive
SPATC1L	Alternative exon	Induction of isoform 2 (exon included)	-0.10	9.95E-02	--	--	--	--	Not assessed
MOCOS	Alternative exon	Switch to isoform 2 (exon excluded)	0.03	6.33E-01	--	--	--	--	Not assessed
RBM45	Alternative exon	Switch to isoform 2 (exon included)	0.08	1.71E-01	--	--	--	--	Not assessed
MIPEP	Alternative exon	Repression of isoform 2 (exon excluded)	-0.16	4.48E-03	--	--	--	--	Not assessed
BBS4	Alternative exon	Induction of isoform 2 (exon included)	-0.06	3.85E-01	--	--	--	--	Not assessed
FAM195A	Alternative exon	Switch to isoform 1 (exon exluded)	0.06	3.37E-01	0.10	6.85E-02	-0.10	1.20E-01	Inconclusive
LINC01133	Alternative exon	Induction of isoform 1 (exon excluded)	--	--	0.00	9.72E-01	--	--	Not assessed
SS18	Alternative exon	Switch to isoform 2 (exon excluded)	0.04	5.68E-01	-0.04	5.46E-01	0.06	3.97E-01	Inconclusive
RHOC	Alternative exon	Switch to isoform 2 (exon excluded)	0.15	6.05E-03	0.11	3.84E-02	0.11	1.00E-01	Inconclusive
ZNF226	Retained intron	Switch to isoform 1 (intron included)	-0.03	6.64E-01	-0.09	1.23E-01	0.07	3.35E-01	Inconclusive

Real-time PCR raw Ct values Click here for additional data file. Copyright: © 2018 Munkley J et al. 2018 Data associated with the article are available under the terms of the Creative Commons Zero "No rights reserved" data waiver (CC0 1.0 Public domain dedication).

Raw unedited western blot images Click here for additional data file. Copyright: © 2018 Munkley J et al. 2018 Data associated with the article are available under the terms of the Creative Commons Zero "No rights reserved" data waiver (CC0 1.0 Public domain dedication).

## Discussion

The main function of the androgen receptor (AR) is as a DNA binding transcription factor that regulates gene expression. Here we show the AR can couple hormone induced gene transcription to alternative mRNA isoform expression in prostate cancer. In response to androgens, the AR can induce the use of alternative promoters, induce the expression of alternatively spliced mRNA isoforms, regulate the expression of non-coding mRNA transcripts, and promote the transcription of mRNA isoforms encoding different protein isoforms. Importantly, we also find that some of these alternative mRNA isoforms are differentially regulated in prostate cancer versus normal tissue and also significantly change expression during tumour progression. Our data suggest that most androgen regulated alternative mRNA isoforms are generated through alternative promoter selection rather than through internal alternative exon splicing mechanisms. This suggests expression of alternative isoforms of specific genes can be a consequence of RNA polymerase being recruited to different promoters in response to androgen stimulation. Alternative promoter usage has been observed for many genes and is believed to play a significant role in the control of gene expression ^4, 105, 106^. Alternative promoter use can also generate mRNA isoforms with distinct functional activities from the same gene, sometimes having opposing functions ^11^.

Androgen exposure further drives a smaller number of alternative splicing events suggesting that the AR could contribute to altered patterns of splicing in prostate cancer cells. Tumour progression is believed to be associated with a coordinated change in splicing patterns which is regulated by several factors including signalling molecules ^7^. We also identified 4 AR regulated alternative mRNA 3′ end isoform switches. This is the first time that regulation of 3′ mRNA end processing has been shown to be controlled by androgens. The selection of alternative 3′ ends can produce mRNA isoforms differing in the length of their 3′ UTRs (which can lead to the inclusion or exclusion of regulatory elements and influence gene expression), or in their C-terminal coding region (which can contribute to proteome diversity) ^107– 114^. Defective 3′ mRNA processing of numerous genes has been linked to an oncogenic phenotype ^115– 119^, and the 3′ mRNA end profiles of samples from multiple cancer types significantly differ from those of healthy tissue samples ^115, 119– 121^.

Based on the findings presented in this study, we propose that activated AR has the ability to coordinate both transcriptional activity and mRNA isoform decisions through the recruitment of co-regulators to specific promoters. The genomic action of the AR is influenced by a large number of collaborating transcription factors ^122– 124^. Specifically, Sam68 and p68 have been shown to modulate AR dependent alternative splicing of specific genes and are significantly overexpressed in prostate cancer ^31, 32^. In future work it will be important to define the role of specific AR co-regulators in AR mediated isoform selection.

Some of the androgen dependent mRNA isoforms identified here are predicted to yield protein isoforms that may be clinically important, or to switch off protein production via generation of noncoding mRNA isoforms. Although the functional significance of the alternative mRNA isoforms identified in this study is yet largely unexplored, as is their role in the cellular response to androgens, the presented results emphasize the importance of analysing gene regulation and function at the mRNA isoform level.

## Data availability

The data referenced by this article are under copyright with the following copyright statement: Copyright: © 2018 Munkley J et al.

Data associated with the article are available under the terms of the Creative Commons Zero "No rights reserved" data waiver (CC0 1.0 Public domain dedication). 

The RNASeq data from LNCaP cells has been published previously https://doi.org/10.1016/j.ebiom.2016.04.018 ^25^

The RNAseq custom tracks are available in Supplementary File 1. To view these files please load them onto the UCSC website using the ‘My data’ tab and ‘custom tracks’. Then ‘Paste URLs or data’. The data is aligned to Feb 2009 (GRCh37/hg19).

Prostate adenocarcinoma cohort RNA-Seq data was downloaded from the Broad Institute TCGA Genome Analysis Center: Firehose 16/01/28 run https://doi.org/10.7908/C11G0KM9 ^43^

Dataset 1: Real-time PCR raw Ct values 10.5256/f1000research.15604.d212873 ^41^

Dataset 2: Raw unedited western blot images 10.5256/f1000research.15604.d212874 ^125^

## Supplementary material

**Supplementary Table 1:** Details of primer sequences used.

**Supplementary File 1:** RNA-Seq reads custom tracks for visualisation on UCSC genome browser

**Supplementary Figure 1:** PCR validation of 17 androgen regulated alternative events.

**Supplementary Figure 2:** Differential alternative mRNA isoform expression in theTGCA PRAD cohort. Normal vs. tumour (unpaired samples) analysis. Violin-boxplots of expression in transcripts per million mapped reads (TPM) of Isoforms 1 (left panel) and 2 (central panel), and of their expression ratio in PSI (right panel) in normal and tumour samples. The mean log2 fold-change (logFC) in expression between tumour and normal samples and the associated FDR-adjusted p-value for the moderated t-statistic of differential expression are shown for both isoforms (left and central panels). The mean difference in PSI (deltaPSI) between tumour and normal samples and the associated FDR-adjusted p-value for the Mann-Whitney U test of differential splicing are shown (right panel).

**Supplementary Figure 3:** Differential alternative mRNA isoform expression in the TGCA PRAD cohort across different Gleason grades. Violin-boxplots of expression in transcripts per million mapped reads (TPM) of Isoforms 1 (left panel) and 2 (central panel), and of their expression ratio (right panel) by Gleason grade. Their respective Spearman’s correlation coefficient (Rho) with grade and associated FDR-adjusted p-value are shown.

**Supplementary Figure 4:** Differential alternative mRNA isoform expression in the TGCA PRAD cohort across different tumour stages. Violin-boxplots of expression in transcripts per million mapped reads (TPM) of Isoforms 1 (left panel) and 2 (central panel), and of their expression ratio (right panel) by tumour stage. Their respective Spearman’s correlation coefficient (Rho) with stage and associated FDR-adjusted p-value are shown.
