# Supplementary material for: Androgen-dependent alternative mRNA isoform expression in prostate cancer cells
Source: F1000Res. 2018 Aug 3;7:1189. [Version 1] doi: 10.12688/f1000research.15604.1 (PMC6143958; doi:10.12688/f1000research.15604.1)
Supplement: Supplementary file 4 [file f1000research-7-17022-s0003.tgz › 4d6e39a8-f02d-48b7-8865-4827c3baa490.docx]

**Supplementary File 1: RNA-Sequencing custom tracks**

SD1-3: LNCaP cells grown without androgens (steroid deplete)

A1-3: LNCaP cells grown with androgens(10nM R1881 for 24 hours)

track type=bigWig name="Munkley_A1" description="Munkley_A1" bigDataUrl=http://folk.uio.no/danielvo/Jenny_bwig/Munkley_run_2_2013-11-20_A1.bwig visibility=full

track type=bigWig name="Munkley_A2" description="Munkley_A2" bigDataUrl=http://folk.uio.no/danielvo/Jenny_bwig/Munkley_run_2_2013-11-20_A2.bwig visibility=full

track type=bigWig name="Munkley_A3" description="Munkley_A3" bigDataUrl=http://folk.uio.no/danielvo/Jenny_bwig/Munkley_run_2_2013-11-20_A3.bwig visibility=full

track type=bigWig name="Munkley_SD1" description="Munkley_SD1" bigDataUrl=http://folk.uio.no/danielvo/Jenny_bwig/Munkley_run_2_2013-11-20_SD1.bwig visibility=full

track type=bigWig name="Munkley_SD2" description="Munkley_SD2" bigDataUrl=http://folk.uio.no/danielvo/Jenny_bwig/Munkley_run_2_2013-11-20_SD2.bwig visibility=full

track type=bigWig name="Munkley_SD3" description="Munkley_SD3" bigDataUrl=http://folk.uio.no/danielvo/Jenny_bwig/Munkley_run_2_2013-11-20_SD3.bwig visibility=full

To view these files please load them onto the UCSC website using the ‘My data’ tab and ‘custom tracks’. Then ‘Paste URLs or data’. The data is aligned to Feb 2009 (GRCh37/hg19).
