# Supplementary material for: Androgen-dependent alternative mRNA isoform expression in prostate cancer cells
Source: F1000Res. 2018 Aug 3;7:1189. [Version 1] doi: 10.12688/f1000research.15604.1 (PMC6143958; doi:10.12688/f1000research.15604.1)
Supplement: Supplementary file 5 [file f1000research-7-17022-s0004.tgz › a024a36c-de79-42fd-81f8-3e8493c59515.pdf]

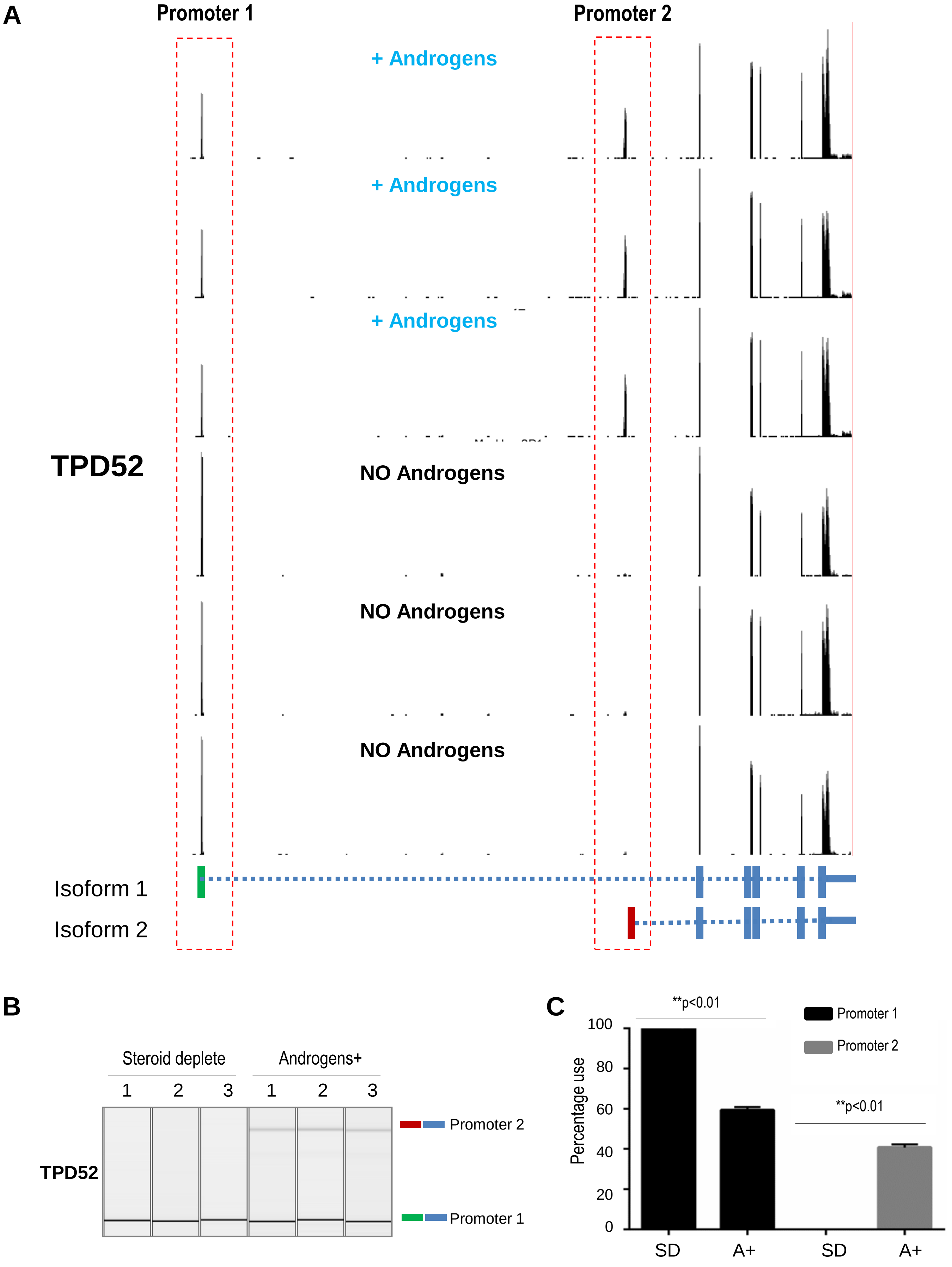

A
Promoter 1
Promoter 2
+ Androgens
+ Androgens
+ Androgens
TPD52
NO Androgens
NO Androgens
NO Androgens
Isoform 1
Isoform 2
B
Steroid deplete
Androgens+
1 2 3 1 2 3
TPD52
Promoter 2
Promoter 1
C
**p<0.01
Promoter 1
Promoter 2
Percentage use
100 80 60 40 20 0
**p<0.01
SD A+ SD A+

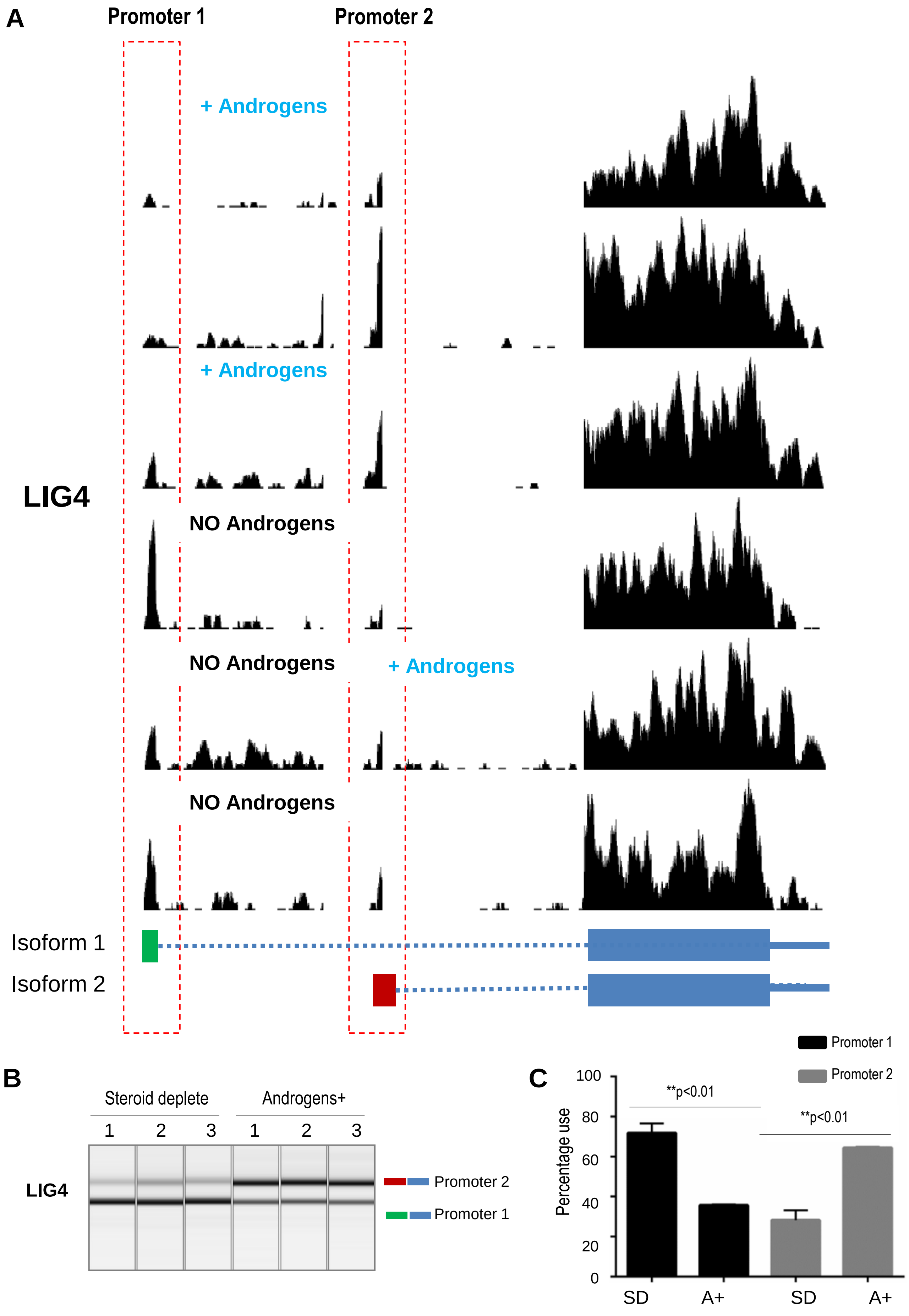

A
Promoter 1
Promoter 2
+ Androgens
+ Androgens
LIG4
NO Androgens
NO Androgens
+ Androgens
NO Androgens
Isoform 1
Isoform 2
B
Steroid deplete
Androgens+
1 2 3 1 2 3
LIG4
Promoter 2
Promoter 1
C
Promoter 1
Promoter 2
Percentage use
100
80
60
40
20
0
**p<0.01
**p<0.01
SD
A+
SD
A+

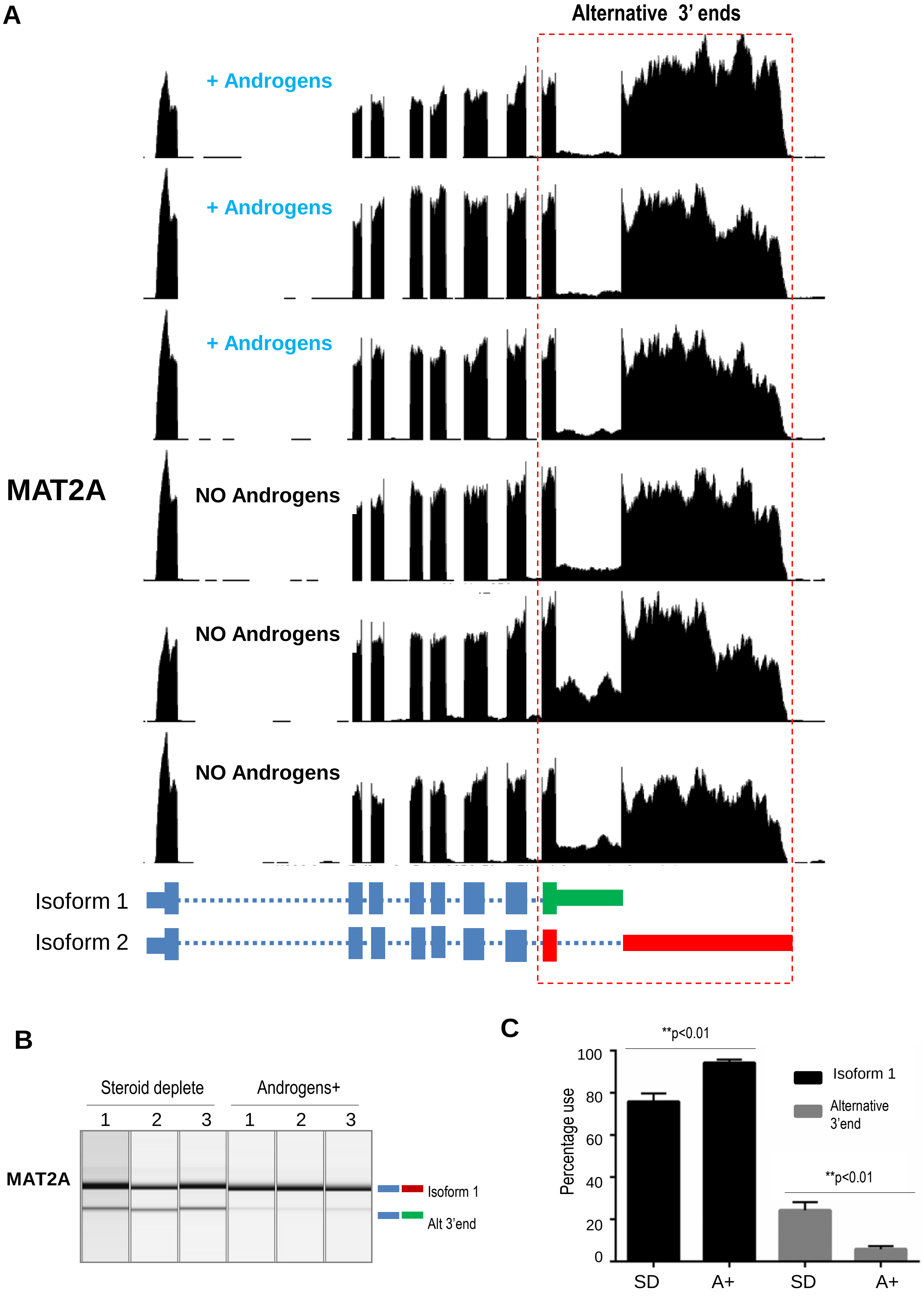

A
Alternative 3' ends
+ Androgens
+ Androgens
+ Androgens
MAT2A
NO Androgens
NO Androgens
NO Androgens
Isoform 1
Isoform 2
B
Steroid deplete
Androgens+
1
2
3
1
2
3
MAT2A
Isoform 1
Alt 3'end
C
**p<0.01
100
80
60
40
20
0
Percentage use
Isoform 1
Alternative 3'end
**p<0.01
SD
A+
SD
A+

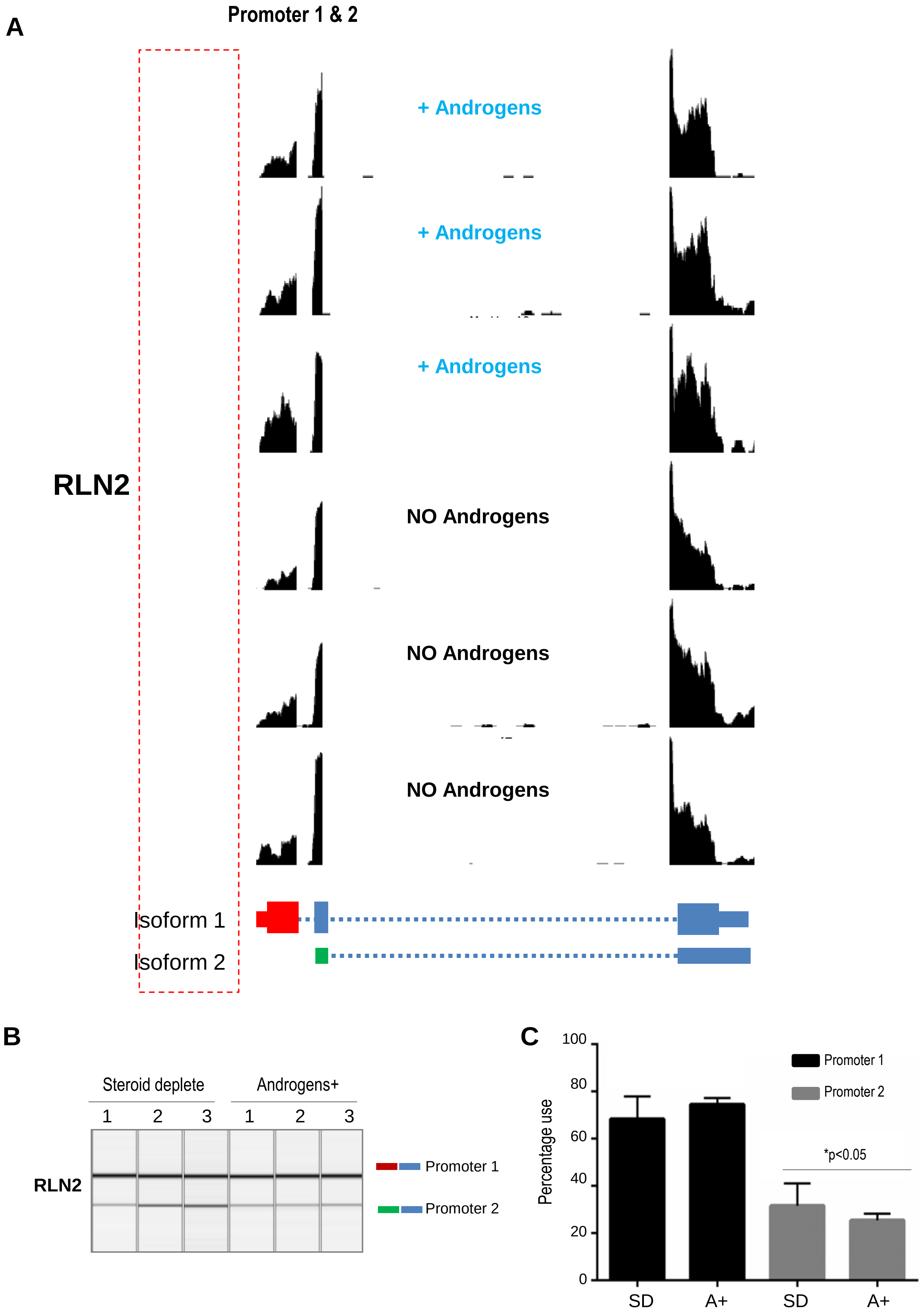
A
Promoter 1 & 2
+ Androgens
+ Androgens
+ Androgens
RLN2
NO Androgens
NO Androgens
NO Androgens
Isoform 1
Isoform 2
B
Steroid deplete
Androgens+
1 2 3 1 2 3
RLN2
Promoter 1
Promoter 2
C
Percentage use
100
80
60
40
20
0
Promoter 1
Promoter 2
*p<0.05
SD
A+
SD
A+

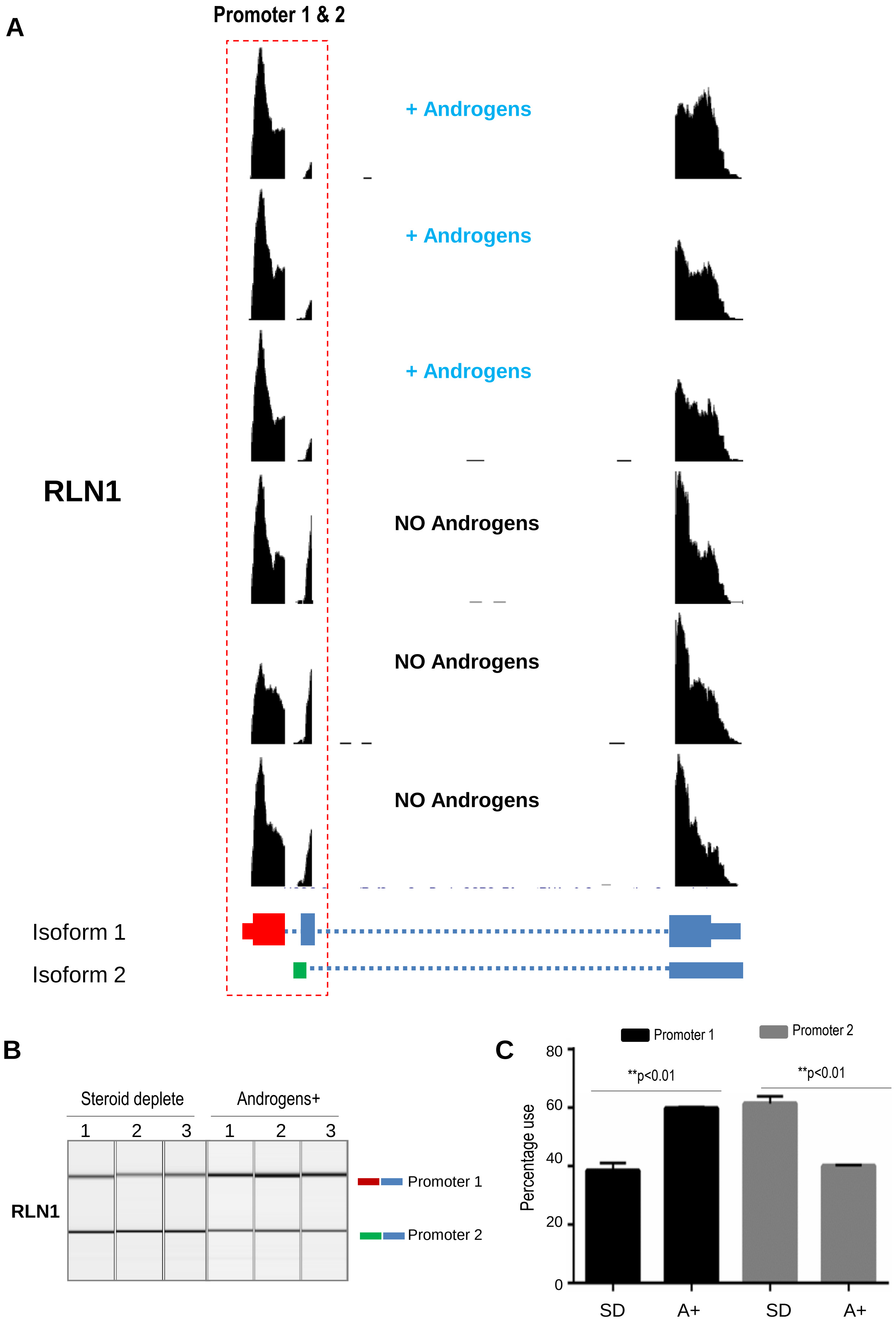

A
Promoter 1 & 2
+ Androgens
+ Androgens
+ Androgens
NO Androgens
NO Androgens
NO Androgens
RLN1
Isoform 1
Isoform 2
B
Steroid deplete
Androgens+
1 2 3 1 2 3
RLN1
Promoter 1
Promoter 2
C
Promoter 1
Promoter 2
**p<0.01
**p<0.01
Percentage use
0
20
40
60
80
SD
A+
SD
A+

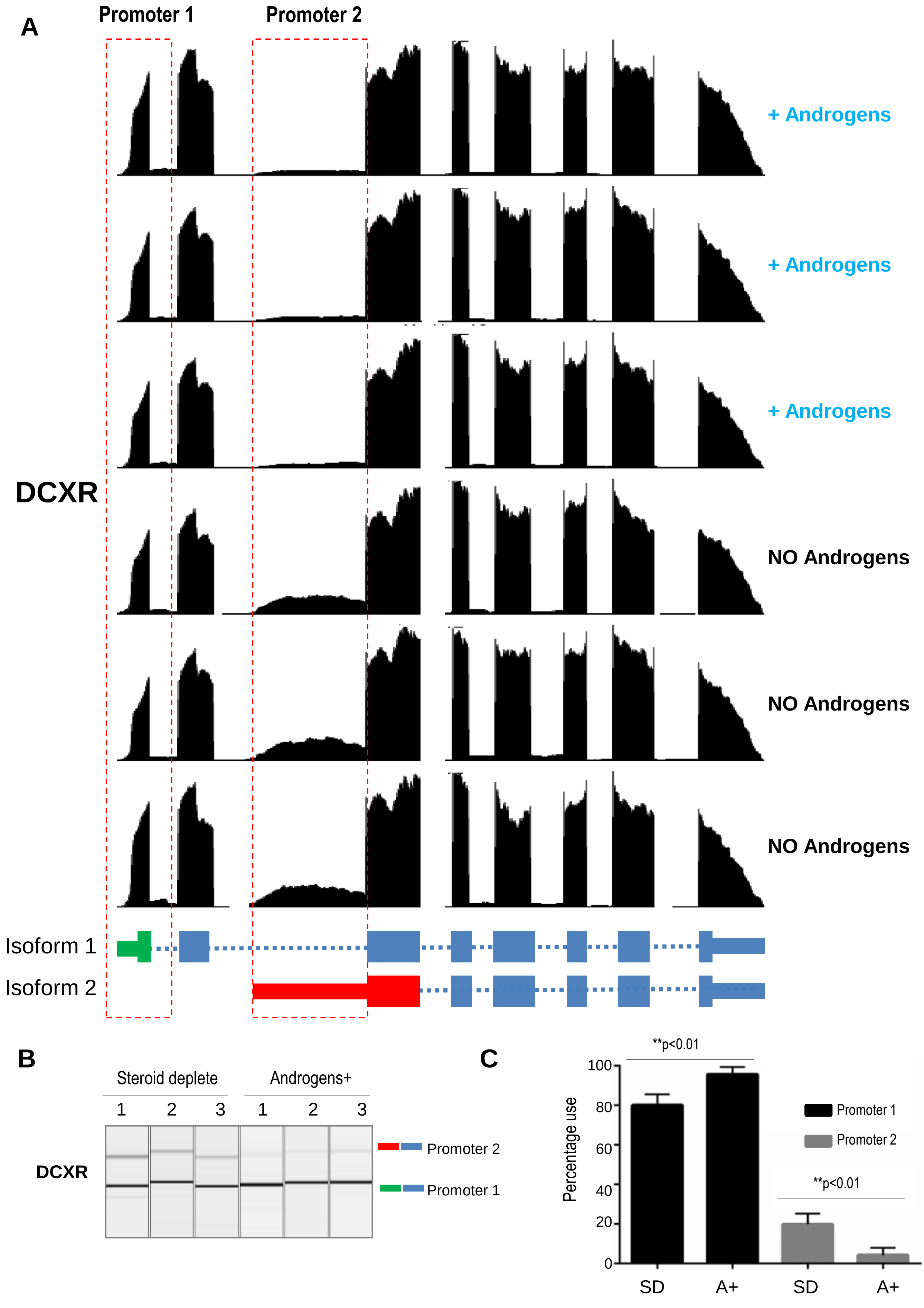
A
Promoter 1
Promoter 2
+ Androgens
+ Androgens
+ Androgens
DCXR
NO Androgens
NO Androgens
NO Androgens
Isoform 1
Isoform 2
B
Steroid deplete
Androgens+
1
2
3
1
2
3
DCXR
Promoter 2
Promoter 1
C
**p<0.01
100
80
60
40
20
0
Percentage use
Promoter 1
Promoter 2
**p<0.01
SD
A+
SD
A+

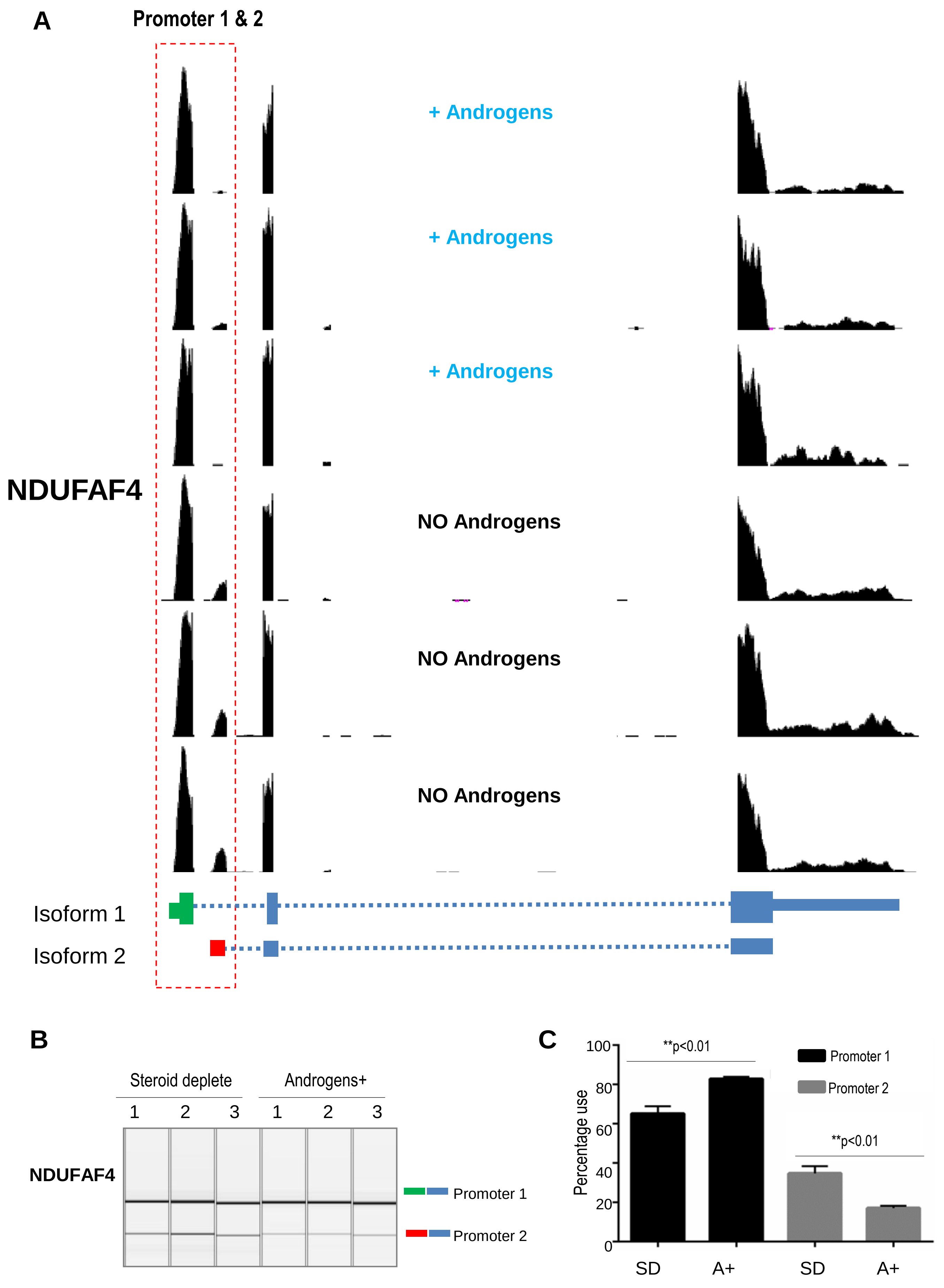

A
Promoter 1 & 2
+ Androgens
+ Androgens
+ Androgens
NDUFAF4
NO Androgens
NO Androgens
NO Androgens
Isoform 1
Isoform 2
B
Steroid deplete
Androgens+
1 2 3 1 2 3
NDUFAF4
Promoter 1
Promoter 2
C
**p<0.01
Promoter 1
Promoter 2
**p<0.01
Percentage use
0 20 40 60 80 100
SD A+ SD A+

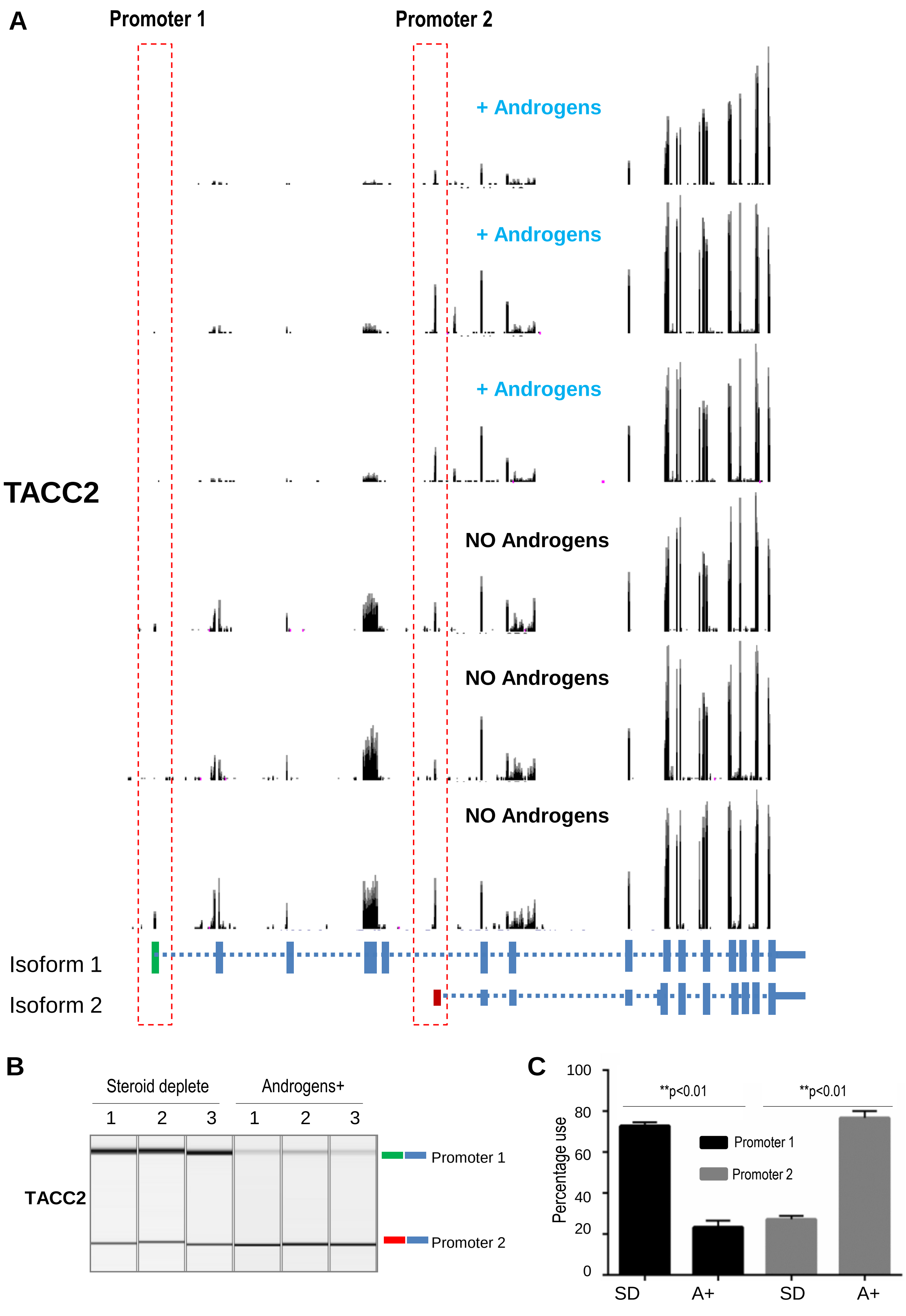

A
Promoter 1
Promoter 2
TACC2
+ Androgens
+ Androgens
+ Androgens
NO Androgens
NO Androgens
NO Androgens
Isoform 1
Isoform 2
B
Steroid deplete
Androgens+
1 2 3 1 2 3
TACC2
Promoter 1
Promoter 2
C
Percentage use
100
80
60
40
20
0
**p<0.01
**p<0.01
Promoter 1
Promoter 2
SD
A+
SD
A+

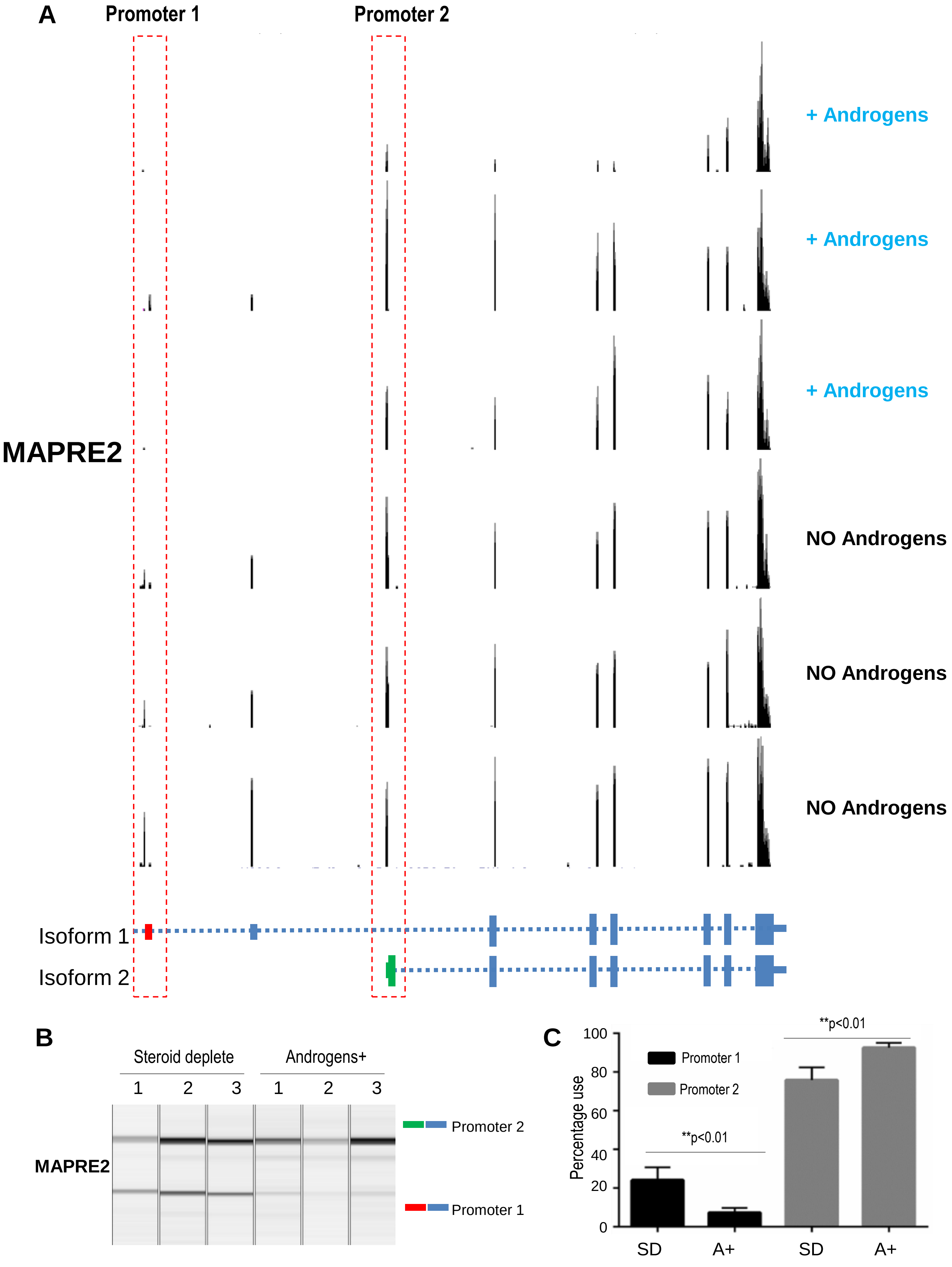
A
Promoter 1
Promoter 2
+ Androgens
+ Androgens
+ Androgens
MAPRE2
NO Androgens
NO Androgens
NO Androgens
Isoform 1
Isoform 2
B
Steroid deplete
Androgens+
1
2
3
1
2
3
MAPRE2
Promoter 2
Promoter 1
C
100
80
60
40
20
0
Percentage use
Promoter 1
Promoter 2
**p<0.01
**p<0.01
SD
A+
SD
A+

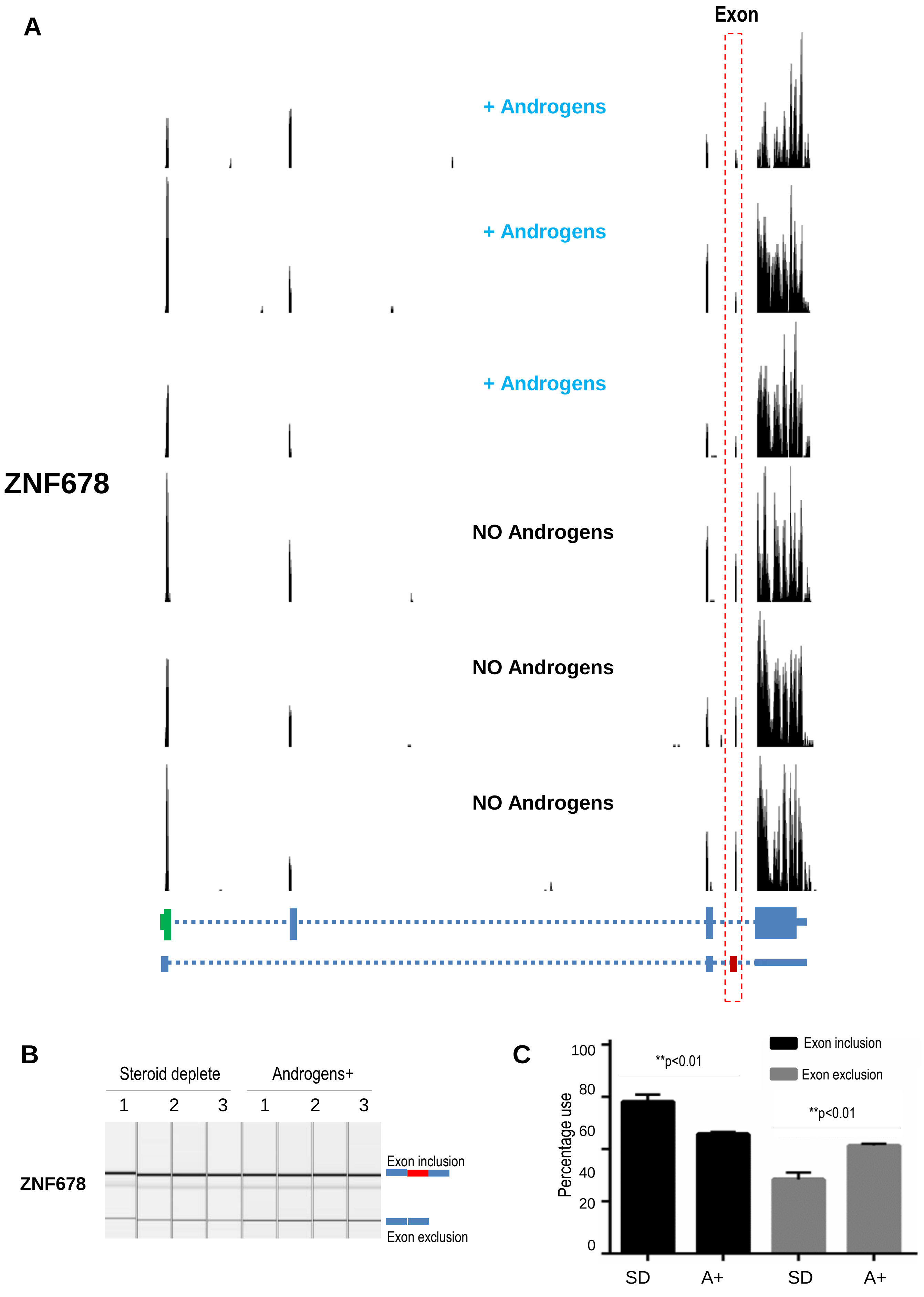
A
Exon
+ Androgens
+ Androgens
+ Androgens
ZNF678
NO Androgens
NO Androgens
NO Androgens
B
Steroid deplete
Androgens+
1 2 3 1 2 3
ZNF678
Exon inclusion
Exon exclusion
C
100
80
60
40
20
0
Percentage use
**p<0.01
**p<0.01
Exon inclusion
Exon exclusion
SD
A+
SD
A+

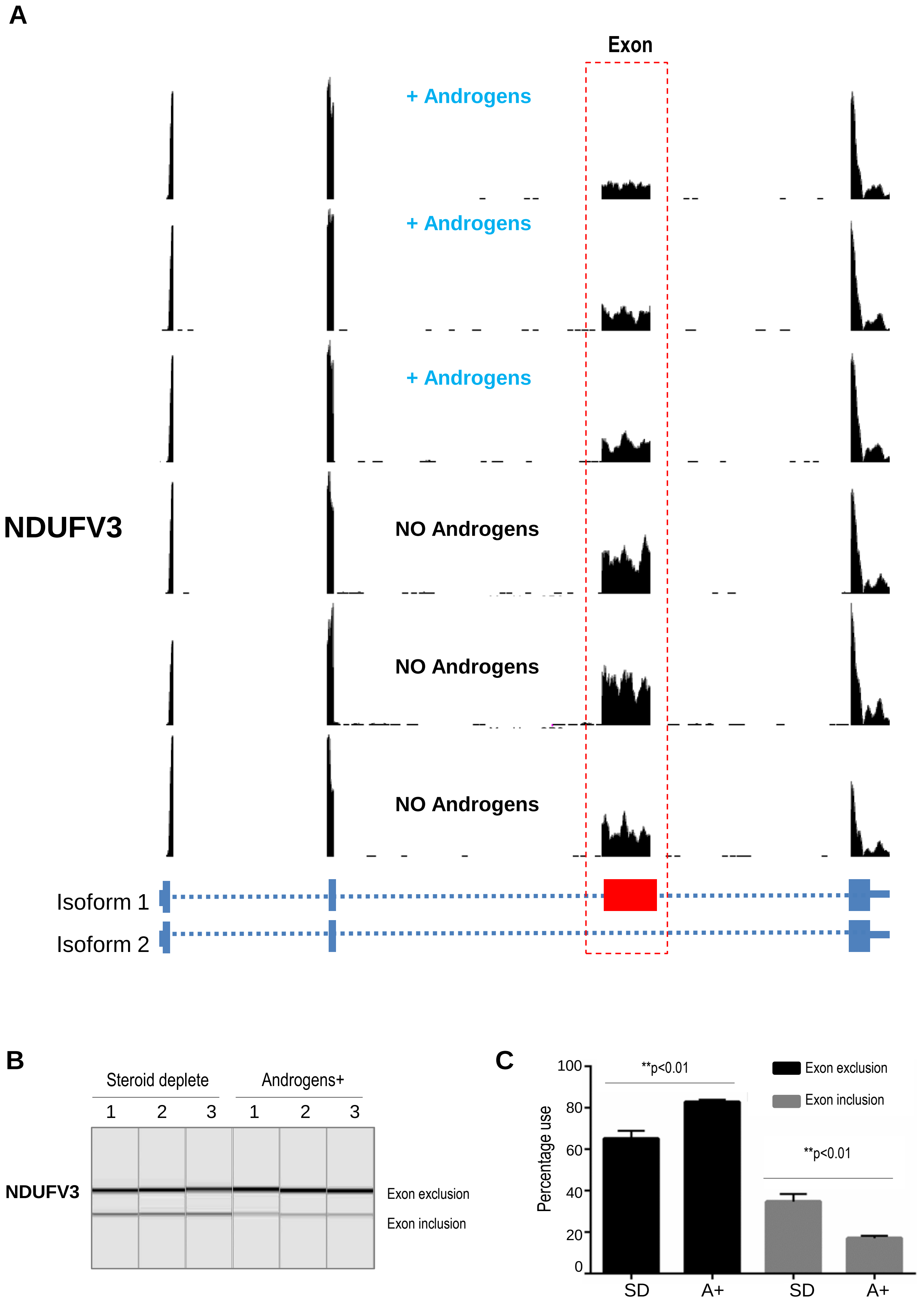
A
Exon
+ Androgens
+ Androgens
+ Androgens
NDUFV3
NO Androgens
NO Androgens
NO Androgens
Isoform 1
Isoform 2
B
Steroid deplete
Androgens+
1 2 3 1 2 3
NDUFV3
Exon exclusion
Exon inclusion
C
Percentage use
100
80
60
40
20
0
**p<0.01
**p<0.01
Exon exclusion
Exon inclusion
SD
A+
SD
A+

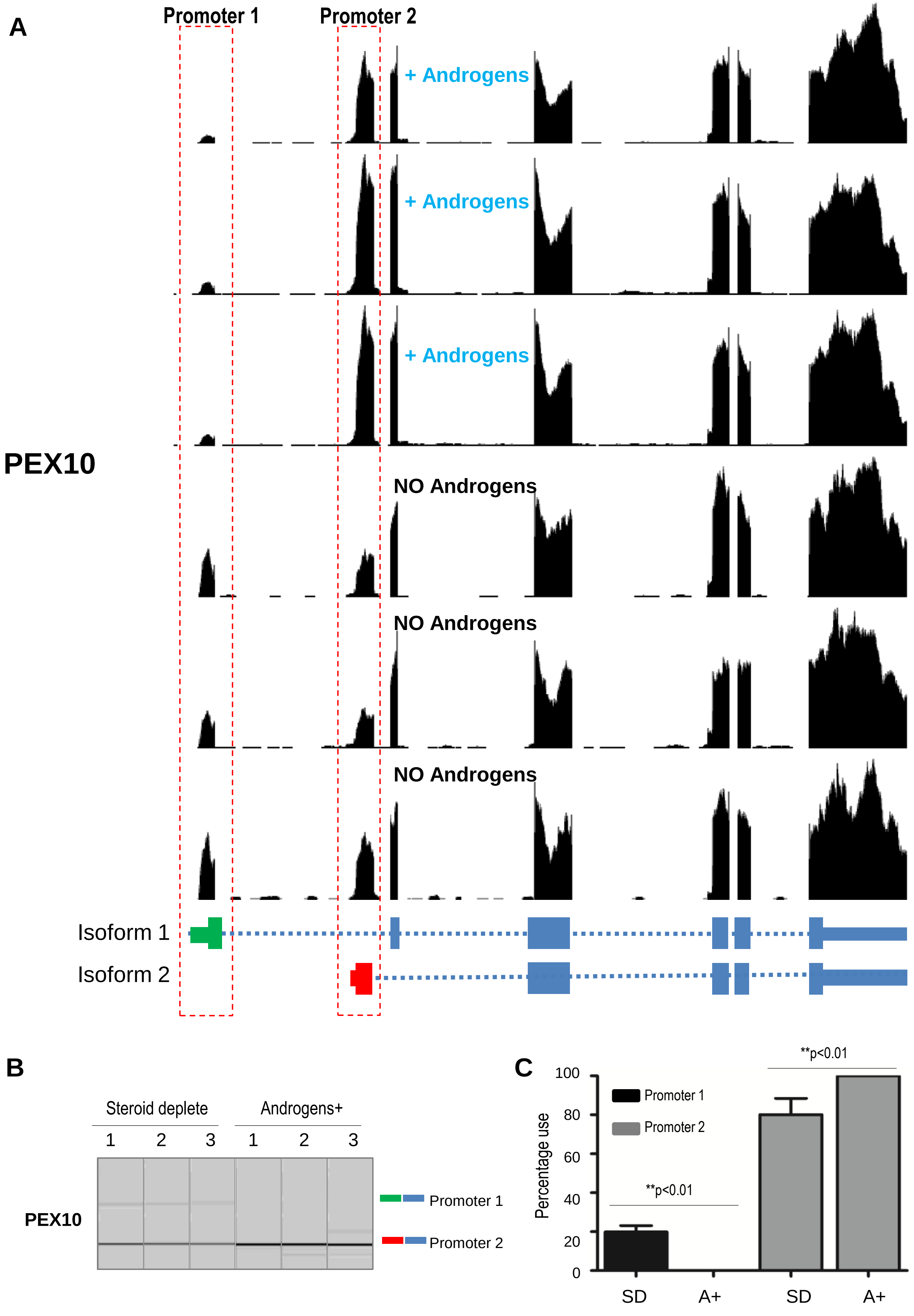
A
Promoter 1
Promoter 2
+ Androgens
+ Androgens
+ Androgens
NO Androgens
NO Androgens
NO Androgens
PEX10
Isoform 1
Isoform 2
B
Steroid deplete
Androgens+
1 2 3 1 2 3
PEX10
Promoter 1
Promoter 2
C
Percentage use
100
80
60
40
20
0
Promoter 1
Promoter 2
**p<0.01
**p<0.01
SD
A+
SD
A+

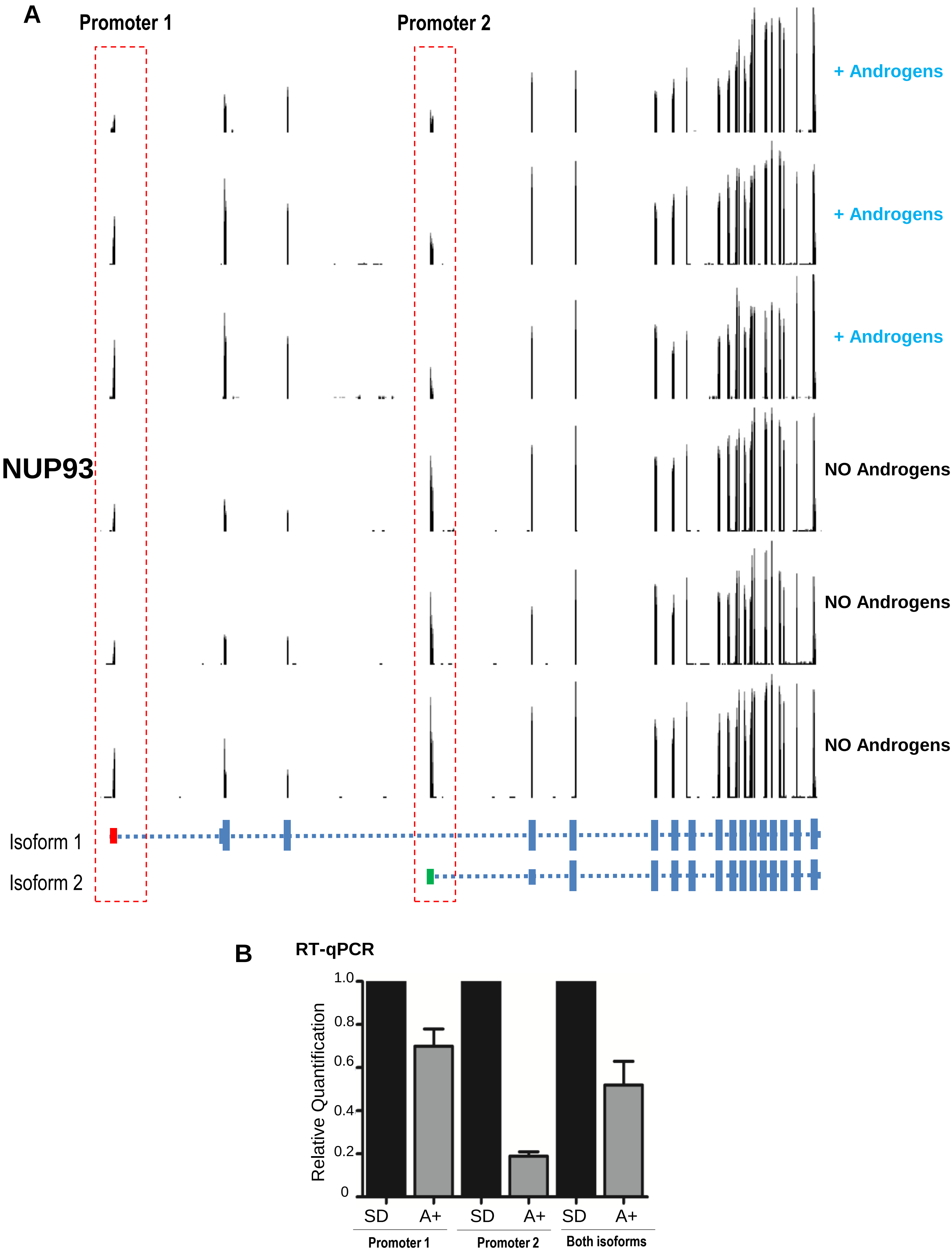
A
Promoter 1
Promoter 2
+ Androgens
+ Androgens
+ Androgens
NUP93
NO Androgens
NO Androgens
NO Androgens
Isoform 1
Isoform 2
B
RT-qPCR
Relative Quantification
1.0
0.8
0.6
0.4
0.2
0
SD
A+
SD
A+
SD
A+
Promoter 1
Promoter 2
Both isoforms

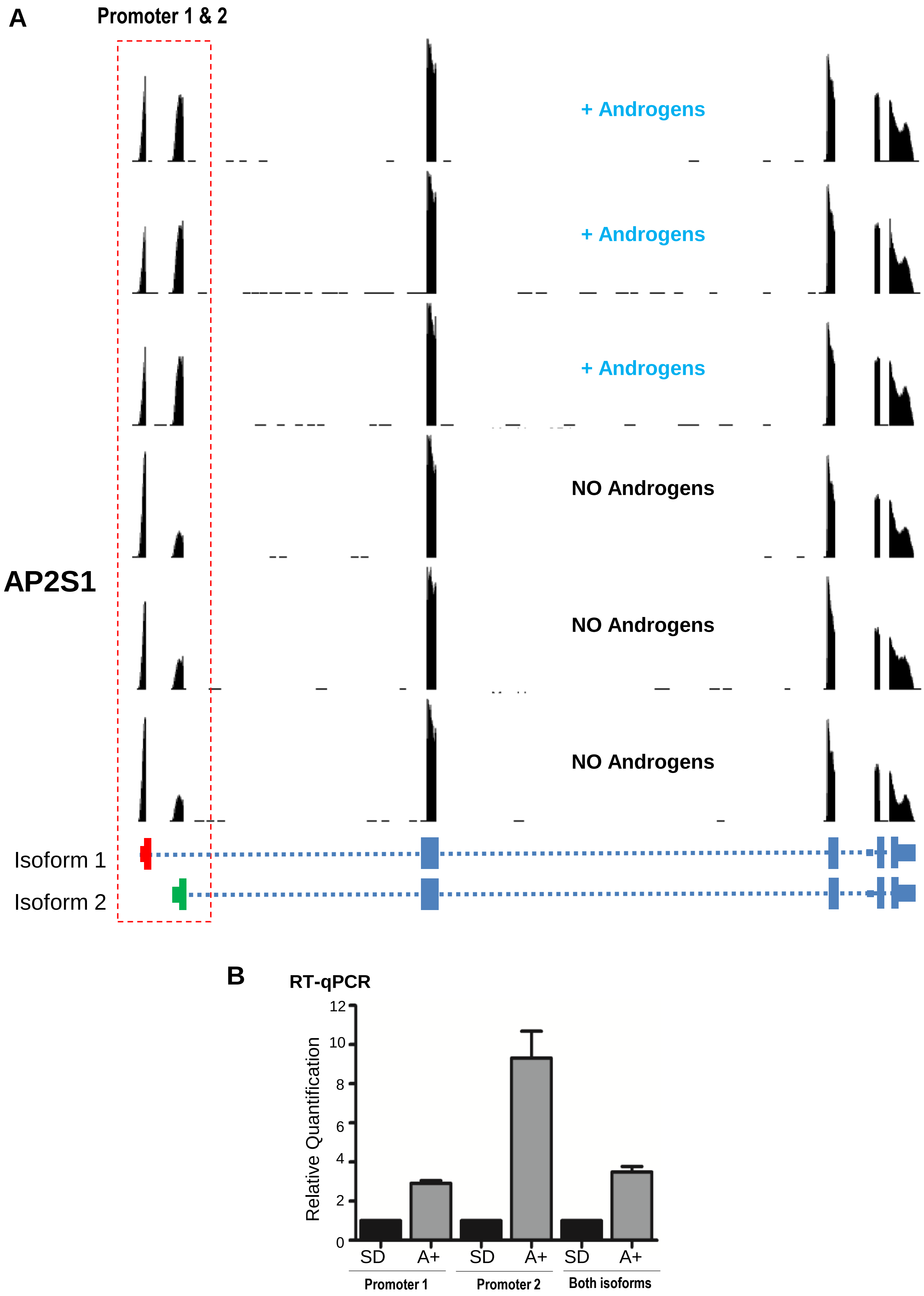

A
Promoter 1 & 2
+ Androgens
+ Androgens
+ Androgens
NO Androgens
NO Androgens
NO Androgens
AP2S1
Isoform 1
Isoform 2
B
RT-qPCR
Relative Quantification
0
2
4
6
8
10
12
SD
A+
SD
A+
SD
A+
Promoter 1
Promoter 2
Both isoforms

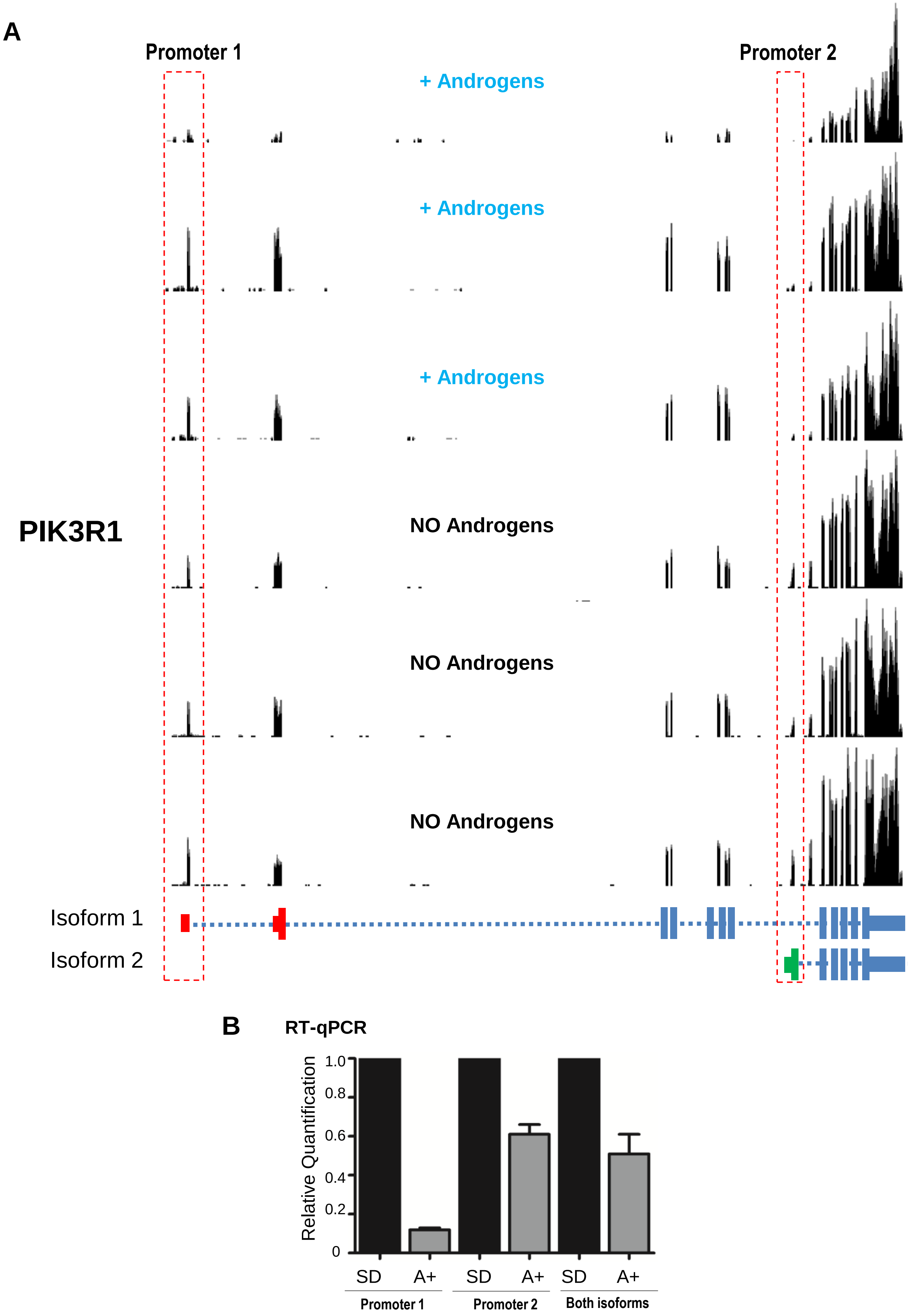
A
Promoter 1
Promoter 2
+ Androgens
+ Androgens
+ Androgens
NO Androgens
NO Androgens
NO Androgens
PIK3R1
Isoform 1
Isoform 2
B
RT-qPCR
Relative Quantification
1.0
0.8
0.6
0.4
0.2
0
SD
A+
SD
A+
SD
A+
Promoter 1
Promoter 2
Both isoforms

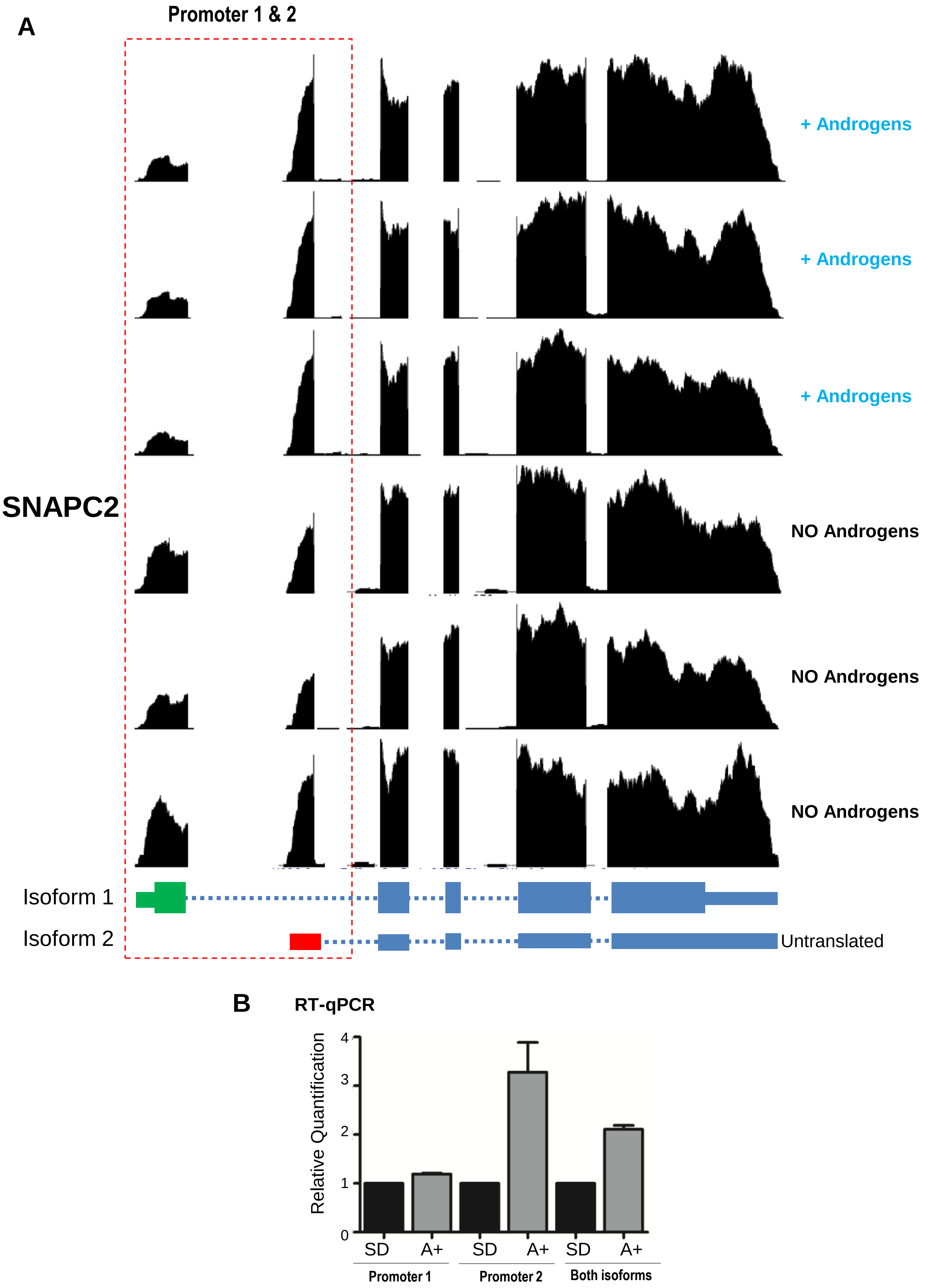
A
Promoter 1 & 2
SNAPC2
+ Androgens
+ Androgens
+ Androgens
NO Androgens
NO Androgens
NO Androgens
Isoform 1
Isoform 2
Untranslated
B
RT-qPCR
Relative Quantification
0
1
2
3
4
SD
A+
SD
A+
SD
A+
Promoter 1
Promoter 2
Both isoforms

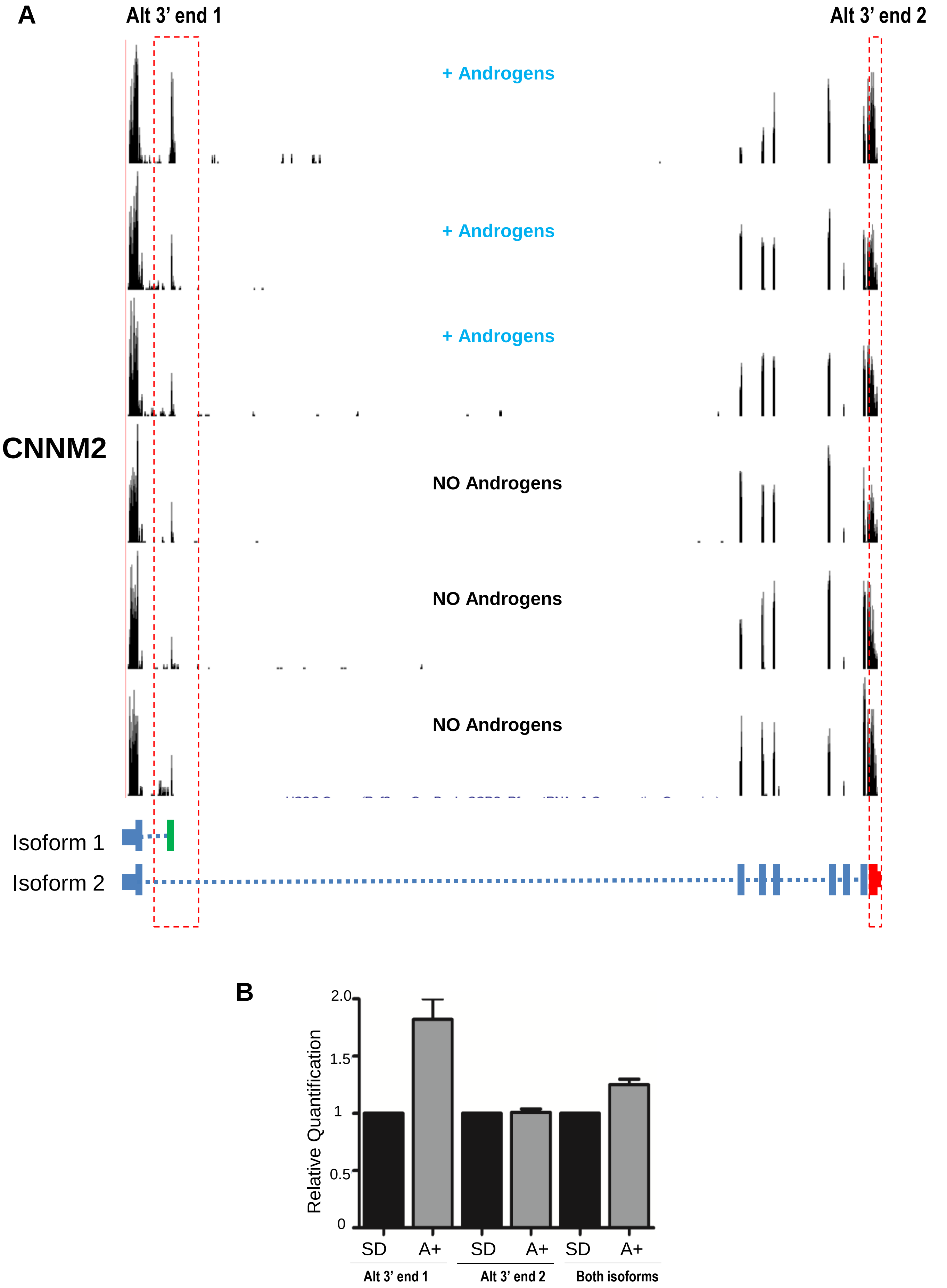
A
Alt 3' end 1
Alt 3' end 2
+ Androgens
+ Androgens
+ Androgens
NO Androgens
NO Androgens
NO Androgens
CNNM2
Isoform 1
Isoform 2
B
Relative Quantification
2.0
1.5
1
0.5
0
SD
A+
SD
A+
SD
A+
Alt 3' end 1
Alt 3' end 2
Both isoforms

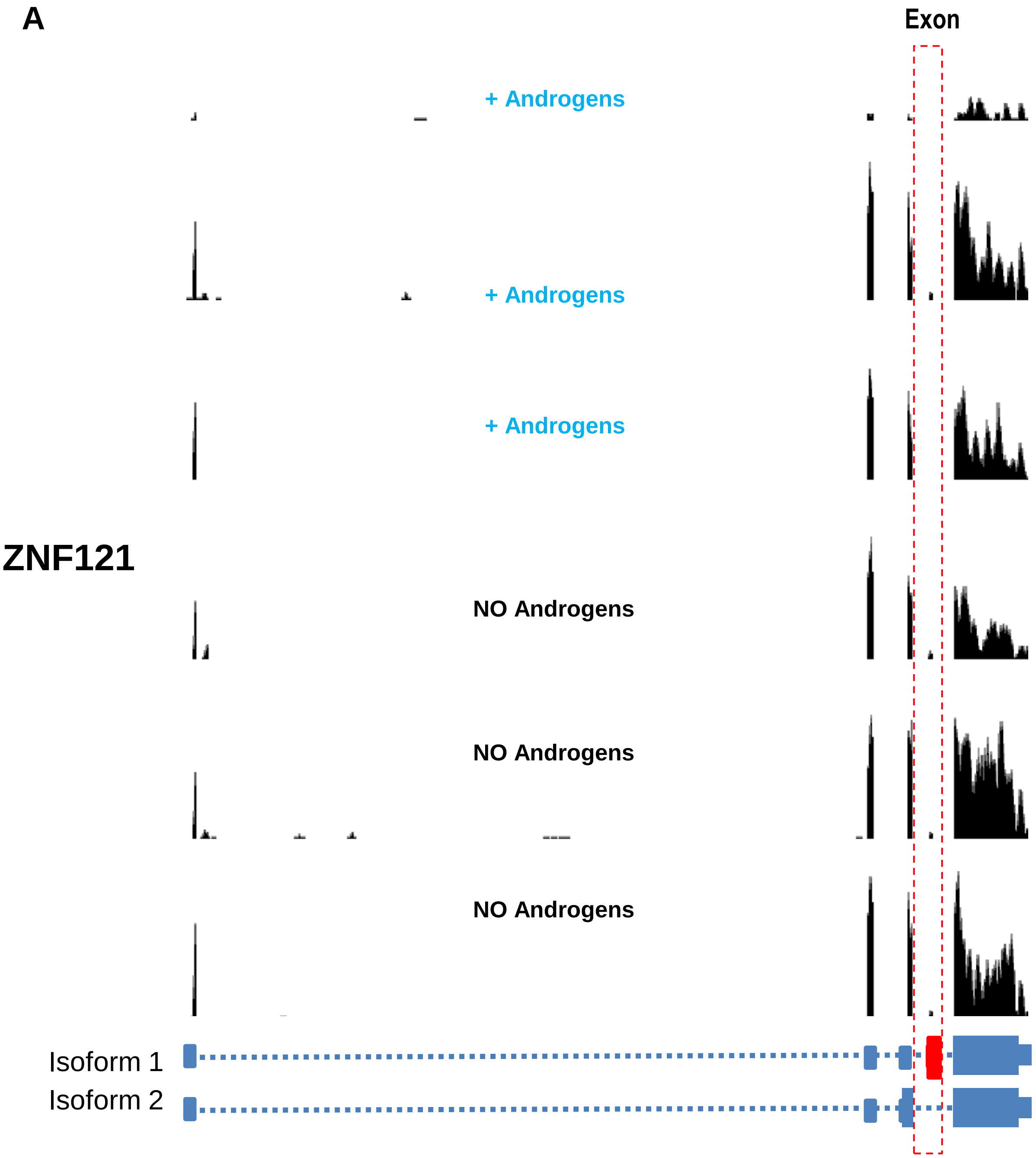

B

Previously validated by Rajan et al. 2011
