# Supplementary material for: Androgen-dependent alternative mRNA isoform expression in prostate cancer cells
Source: F1000Res. 2018 Aug 3;7:1189. [Version 1] doi: 10.12688/f1000research.15604.1 (PMC6143958; doi:10.12688/f1000research.15604.1)
Supplement: Supplementary file 6 [file f1000research-7-17022-s0005.tgz › f522d174-3d4a-462d-bc79-2f789780ba97.pdf]

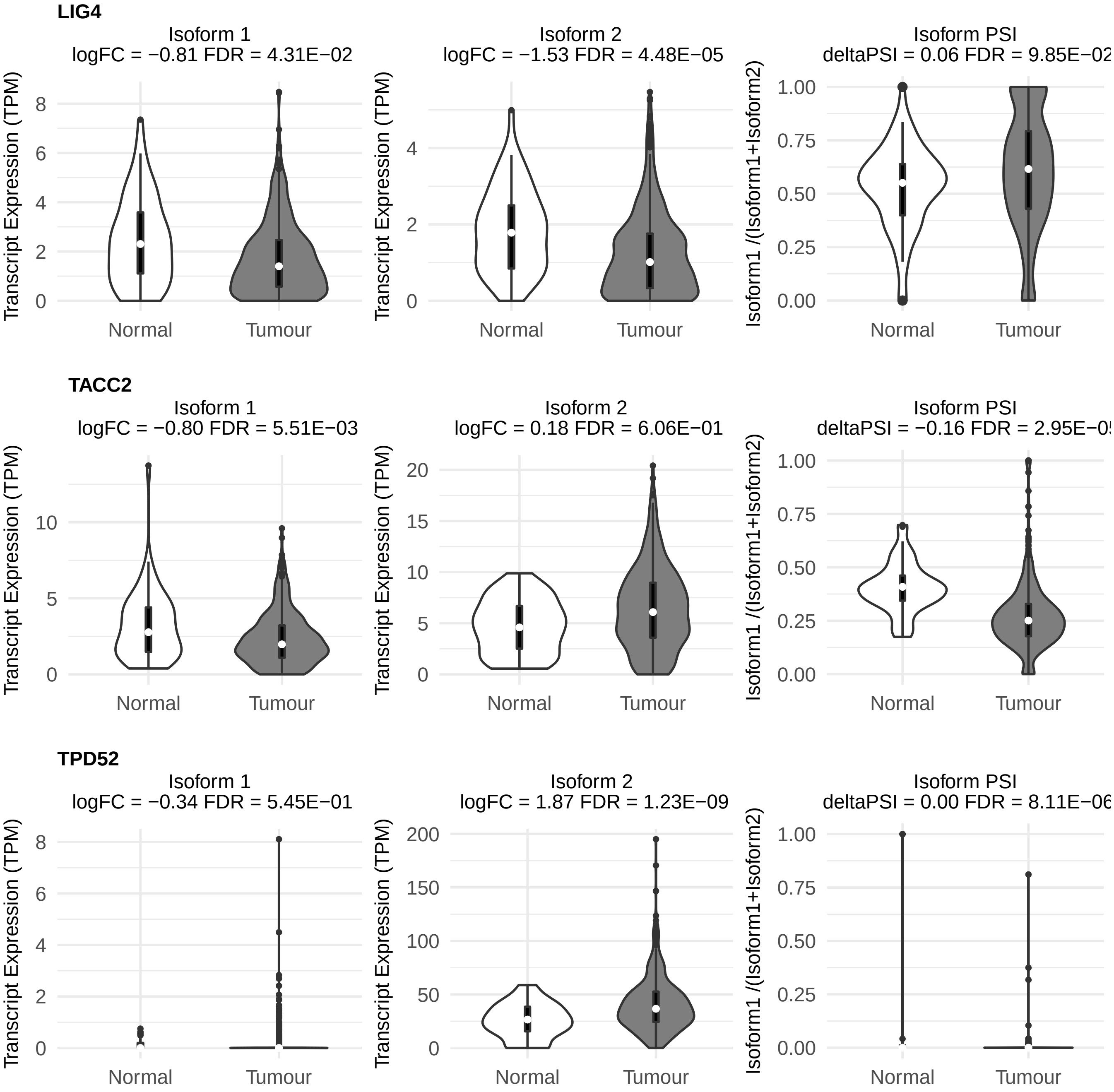

LIG4
Isoform 1
logFC = −0.81 FDR = 4.31E−02
Isoform 2
logFC = −1.53 FDR = 4.48E−05
Isoform PSI
deltaPSI = 0.06 FDR = 9.85E−02
Transcript Expression (TPM)
Isoform1 /(Isoform1+Isoform2)
Normal
Tumour
TACC2
Isoform 1
logFC = −0.80 FDR = 5.51E−03
Isoform 2
logFC = 0.18 FDR = 6.06E−01
Isoform PSI
deltaPSI = −0.16 FDR = 2.95E−05
TPD52
Isoform 1
logFC = −0.34 FDR = 5.45E−01
Isoform 2
logFC = 1.87 FDR = 1.23E−09
Isoform PSI
deltaPSI = 0.00 FDR = 8.11E−06

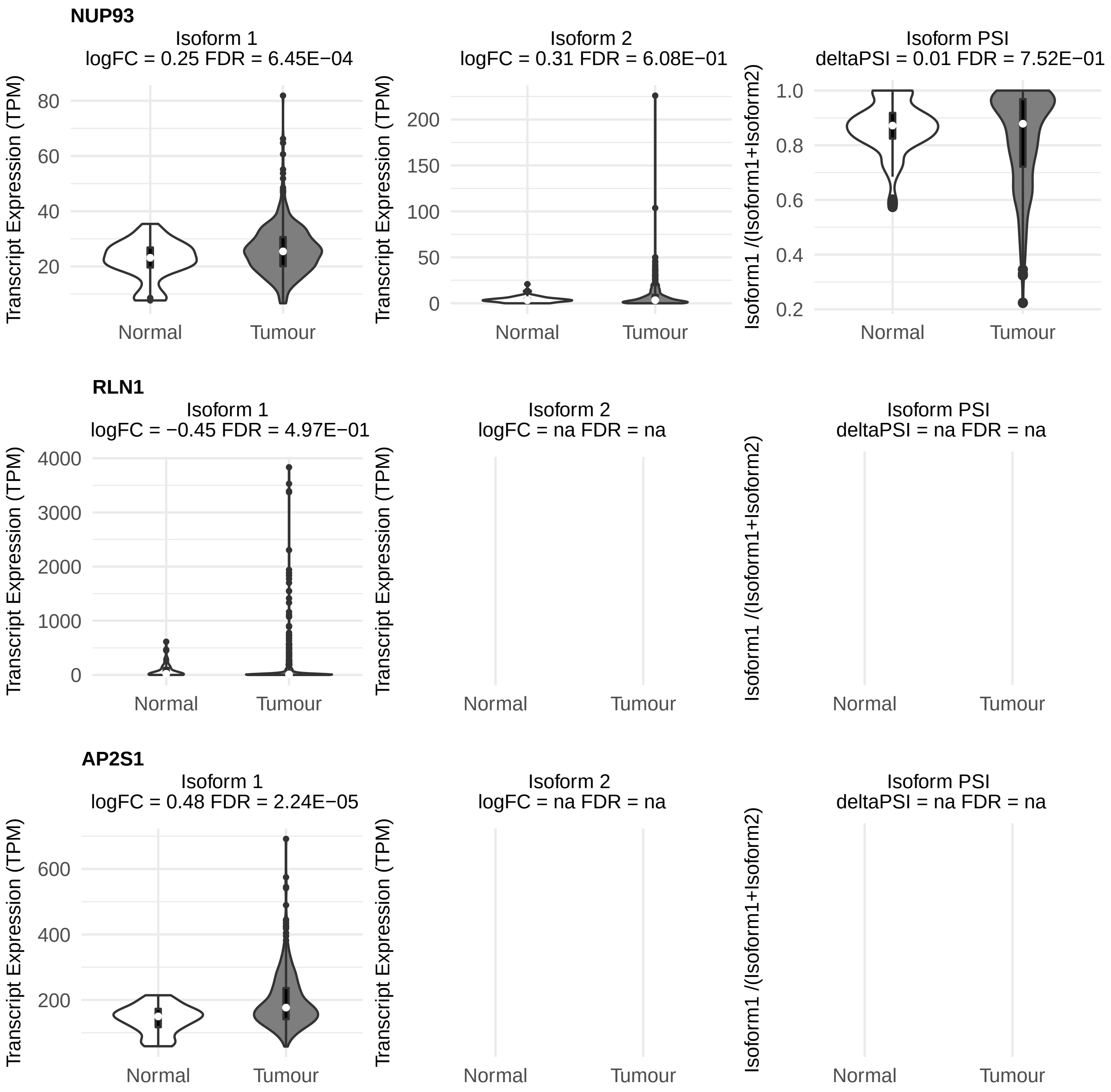
NUP93
Isoform 1
logFC = 0.25 FDR = 6.45E−04
Transcript Expression (TPM)
80
60
40
20
Normal
Tumour
Isoform 2
logFC = 0.31 FDR = 6.08E−01
Transcript Expression (TPM)
200
150
100
50
0
Normal
Tumour
Isoform PSI
deltaPSI = 0.01 FDR = 7.52E−01
Isoform1 /(Isoform1+Isoform2)
1.0
0.8
0.6
0.4
0.2
Normal
Tumour
RLN1
Isoform 1
logFC = −0.45 FDR = 4.97E−01
Transcript Expression (TPM)
4000
3000
2000
1000
0
Normal
Tumour
Isoform 2
logFC = na FDR = na
Transcript Expression (TPM)
Normal
Tumour
Isoform PSI
deltaPSI = na FDR = na
Isoform1 /(Isoform1+Isoform2)
Normal
Tumour
AP2S1
Isoform 1
logFC = 0.48 FDR = 2.24E−05
Transcript Expression (TPM)
600
400
200
Normal
Tumour
Isoform 2
logFC = na FDR = na
Transcript Expression (TPM)
Normal
Tumour
Isoform PSI
deltaPSI = na FDR = na
Isoform1 /(Isoform1+Isoform2)
Normal
Tumour

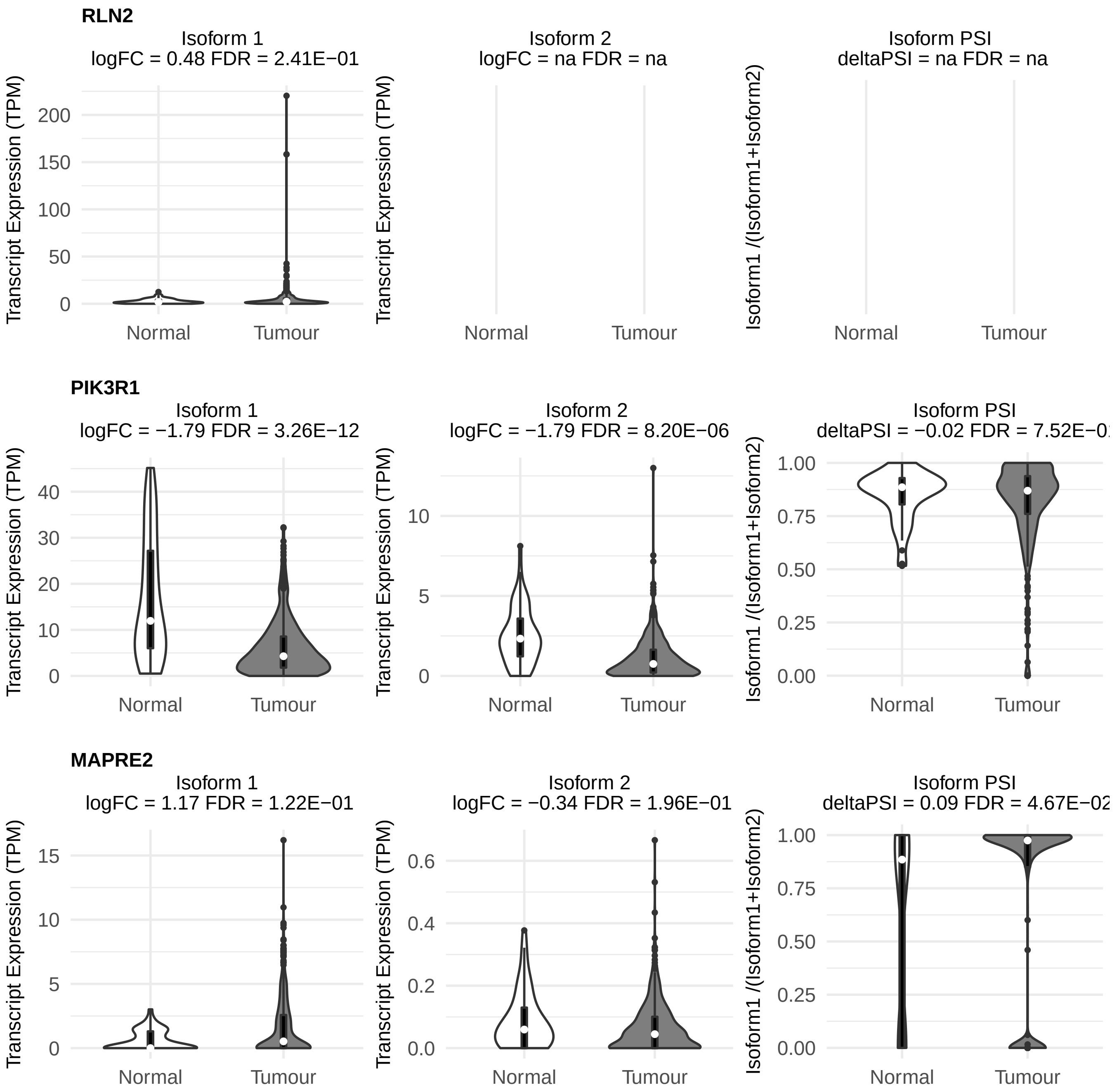
RLN2
Isoform 1
logFC = 0.48 FDR = 2.41E−01
Isoform 2
logFC = na FDR = na
Isoform PSI
deltaPSI = na FDR = na
Transcript Expression (TPM)
Isoform1 /(Isoform1+Isoform2)
Normal
Tumour
PIK3R1
Isoform 1
logFC = −1.79 FDR = 3.26E−12
Isoform 2
logFC = −1.79 FDR = 8.20E−06
Isoform PSI
deltaPSI = −0.02 FDR = 7.52E−0
MAPRE2
Isoform 1
logFC = 1.17 FDR = 1.22E−01
Isoform 2
logFC = −0.34 FDR = 1.96E−01
Isoform PSI
deltaPSI = 0.09 FDR = 4.67E−02

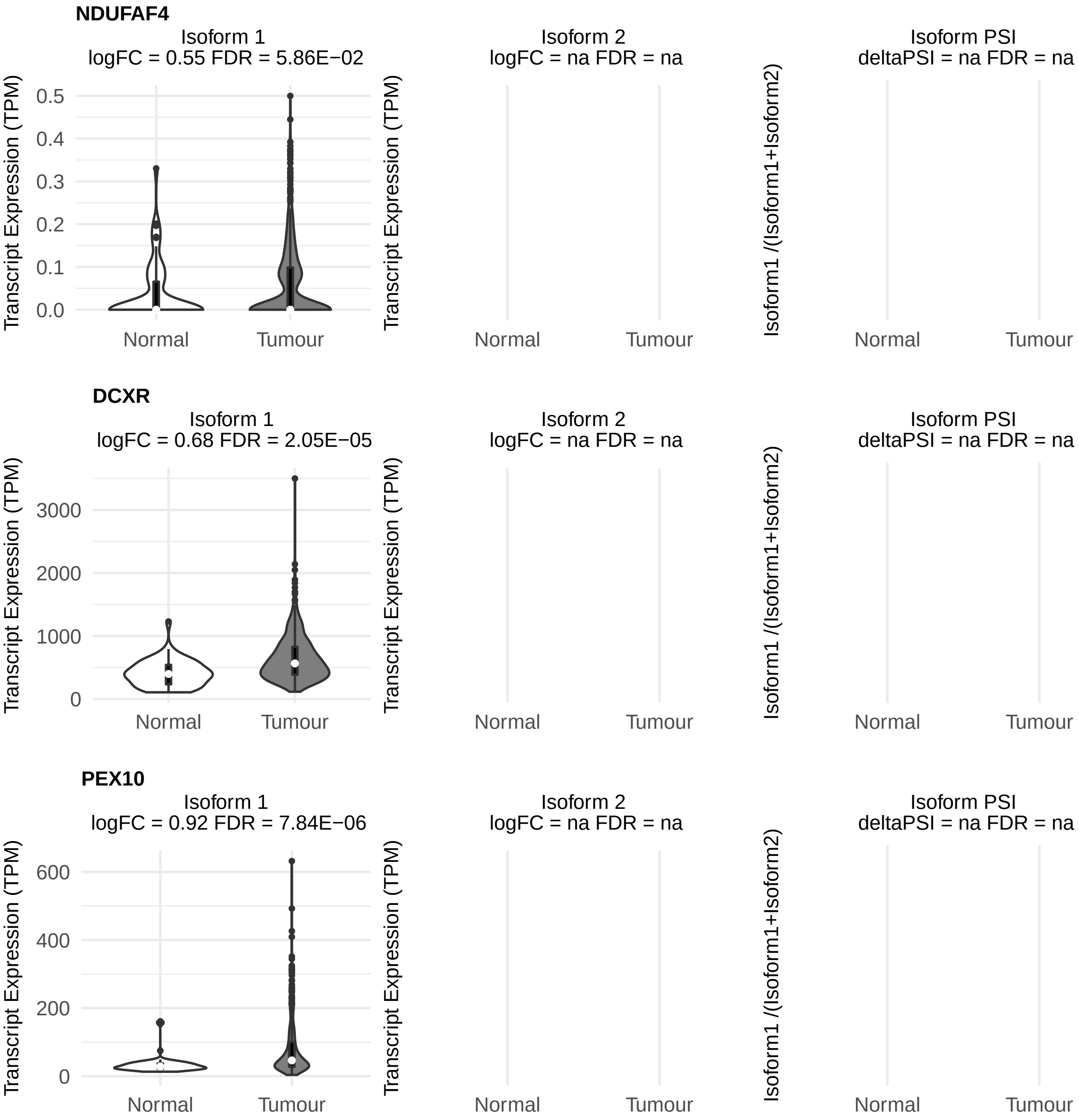
NDUFAF4
Isoform 1
logFC = 0.55 FDR = 5.86E−02
Transcript Expression (TPM)
0.0
0.1
0.2
0.3
0.4
0.5
Normal
Tumour
Isoform 2
logFC = na FDR = na
Transcript Expression (TPM)
Normal
Tumour
Isoform PSI
deltaPSI = na FDR = na
Isoform1 /(Isoform1+Isoform2)
Normal
Tumour
DCXR
Isoform 1
logFC = 0.68 FDR = 2.05E−05
Transcript Expression (TPM)
0
1000
2000
3000
Normal
Tumour
Isoform 2
logFC = na FDR = na
Transcript Expression (TPM)
Normal
Tumour
Isoform PSI
deltaPSI = na FDR = na
Isoform1 /(Isoform1+Isoform2)
Normal
Tumour
PEX10
Isoform 1
logFC = 0.92 FDR = 7.84E−06
Transcript Expression (TPM)
0
200
400
600
Normal
Tumour
Isoform 2
logFC = na FDR = na
Transcript Expression (TPM)
Normal
Tumour
Isoform PSI
deltaPSI = na FDR = na
Isoform1 /(Isoform1+Isoform2)
Normal
Tumour

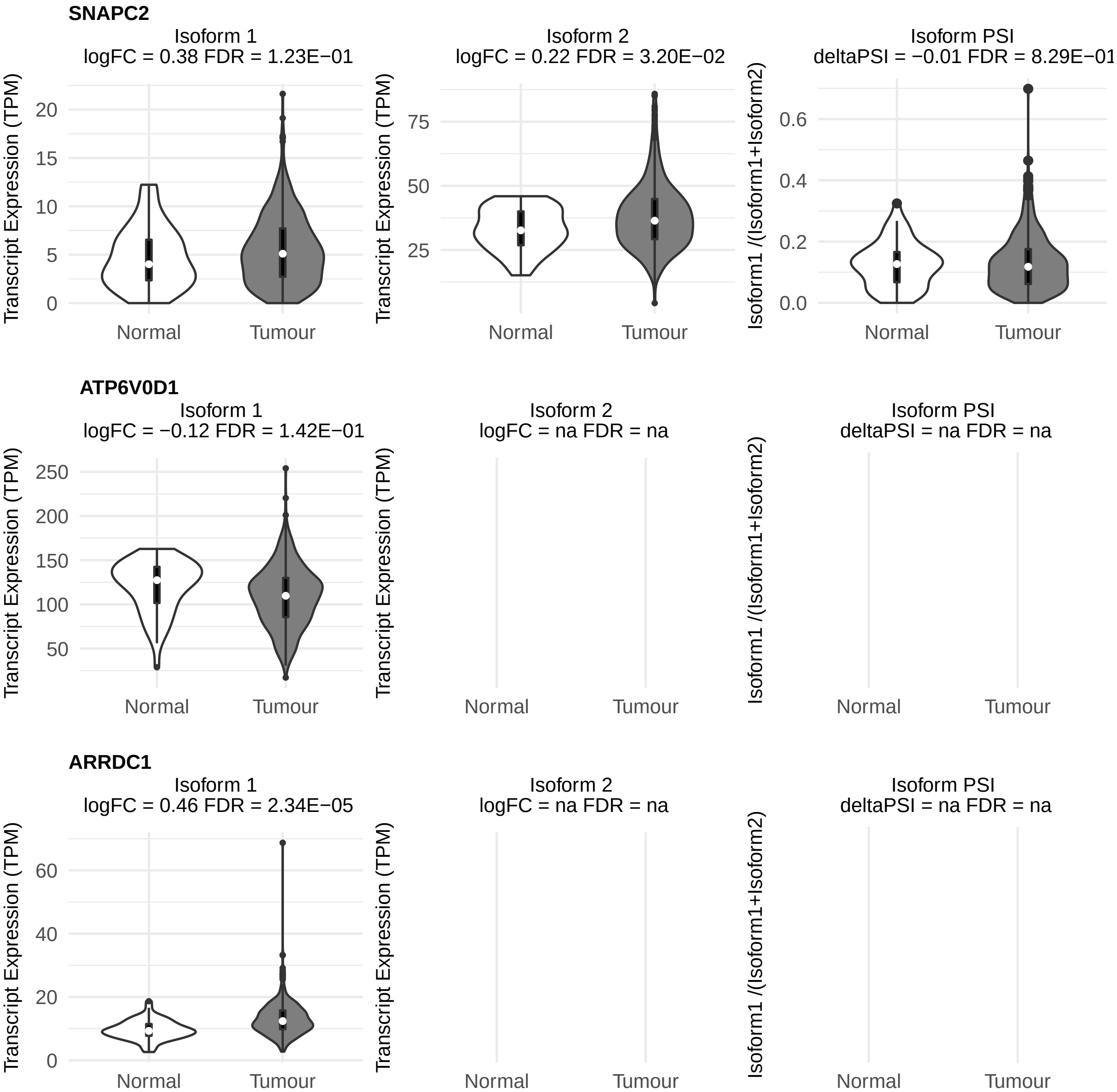
SNAPC2
Isoform 1
logFC = 0.38 FDR = 1.23E−01
Isoform 2
logFC = 0.22 FDR = 3.20E−02
Isoform PSI
deltaPSI = −0.01 FDR = 8.29E−01
Transcript Expression (TPM)
Isoform1 /(Isoform1+Isoform2)
0
5
10
15
20
25
50
75
0.0
0.2
0.4
0.6
Normal
Tumour
ATP6V0D1
Isoform 1
logFC = −0.12 FDR = 1.42E−01
Isoform 2
logFC = na FDR = na
Isoform PSI
deltaPSI = na FDR = na
50
100
150
200
250
ARRDC1
Isoform 1
logFC = 0.46 FDR = 2.34E−05
Isoform 2
logFC = na FDR = na
Isoform PSI
deltaPSI = na FDR = na
0
20
40
60

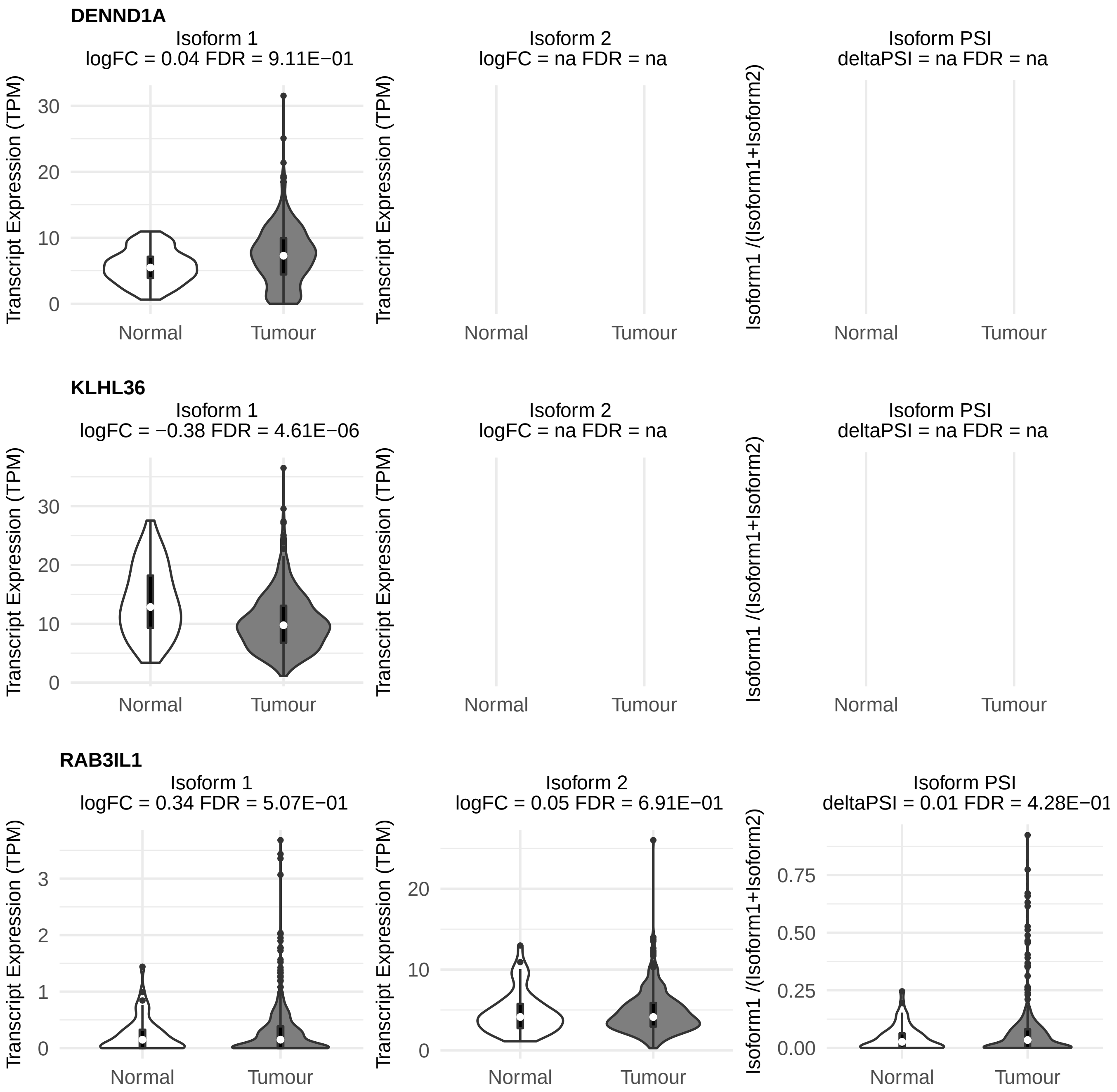

DENND1A
Isoform 1
logFC = 0.04 FDR = 9.11E−01
Transcript Expression (TPM)
0
10
20
30
Normal
Tumour
Isoform 2
logFC = na FDR = na
Transcript Expression (TPM)
Normal
Tumour
Isoform PSI
deltaPSI = na FDR = na
Isoform1 /(Isoform1+Isoform2)
Normal
Tumour
KLHL36
Isoform 1
logFC = −0.38 FDR = 4.61E−06
Transcript Expression (TPM)
0
10
20
30
Normal
Tumour
Isoform 2
logFC = na FDR = na
Transcript Expression (TPM)
Normal
Tumour
Isoform PSI
deltaPSI = na FDR = na
Isoform1 /(Isoform1+Isoform2)
Normal
Tumour
RAB3IL1
Isoform 1
logFC = 0.34 FDR = 5.07E−01
Transcript Expression (TPM)
0
1
2
3
Normal
Tumour
Isoform 2
logFC = 0.05 FDR = 6.91E−01
Transcript Expression (TPM)
0
10
20
Normal
Tumour
Isoform PSI
deltaPSI = 0.01 FDR = 4.28E−01
Isoform1 /(Isoform1+Isoform2)
0.00
0.25
0.50
0.75
Normal
Tumour

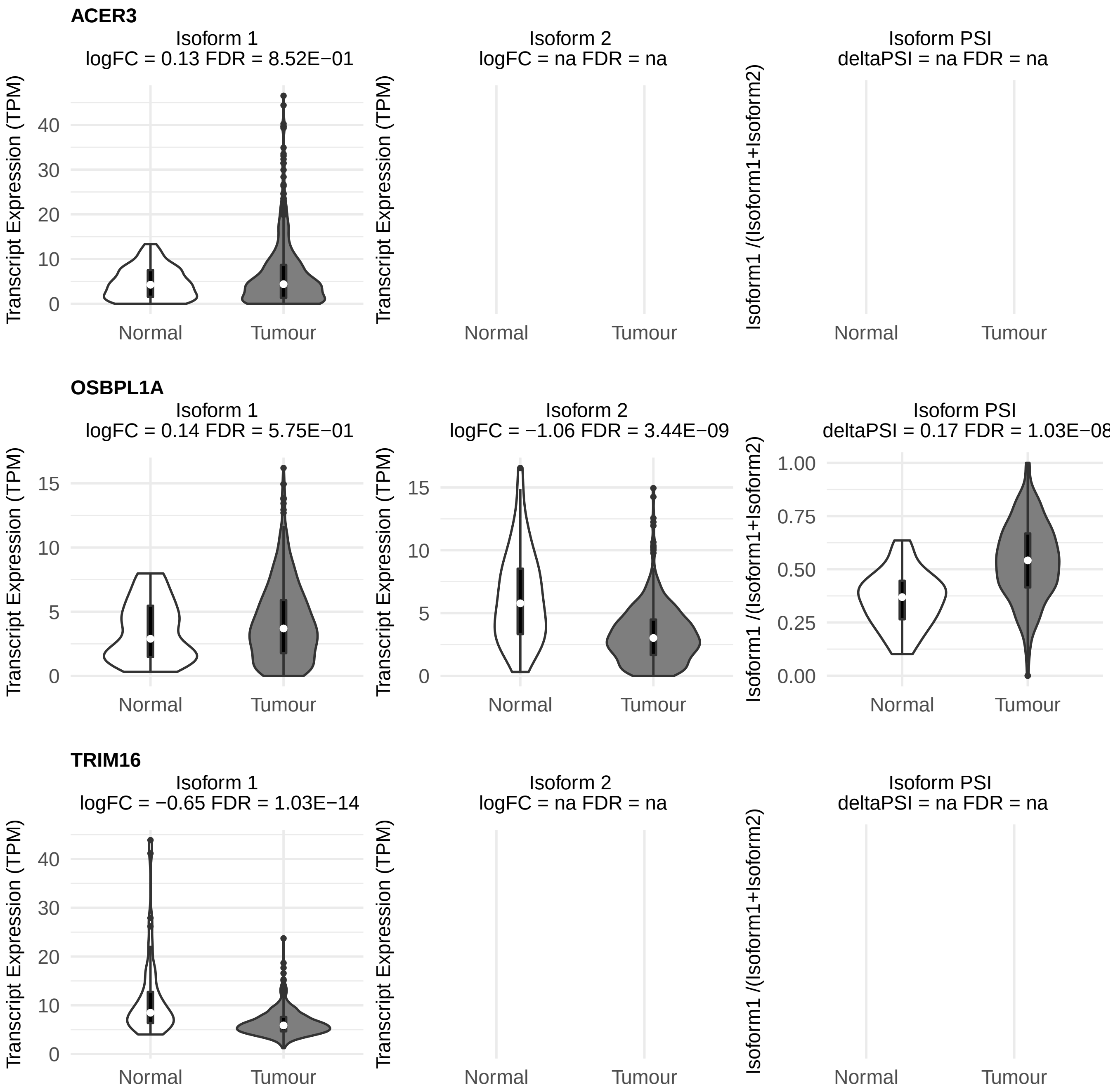

ACER3
Isoform 1
logFC = 0.13 FDR = 8.52E−01
Isoform 2
logFC = na FDR = na
Isoform PSI
deltaPSI = na FDR = na
Transcript Expression (TPM)
Isoform1 /(Isoform1+Isoform2)
Normal
Tumour
OSBPL1A
Isoform 1
logFC = 0.14 FDR = 5.75E−01
Isoform 2
logFC = −1.06 FDR = 3.44E−09
Isoform PSI
deltaPSI = 0.17 FDR = 1.03E−08
TRIM16
Isoform 1
logFC = −0.65 FDR = 1.03E−14
Isoform 2
logFC = na FDR = na
Isoform PSI
deltaPSI = na FDR = na

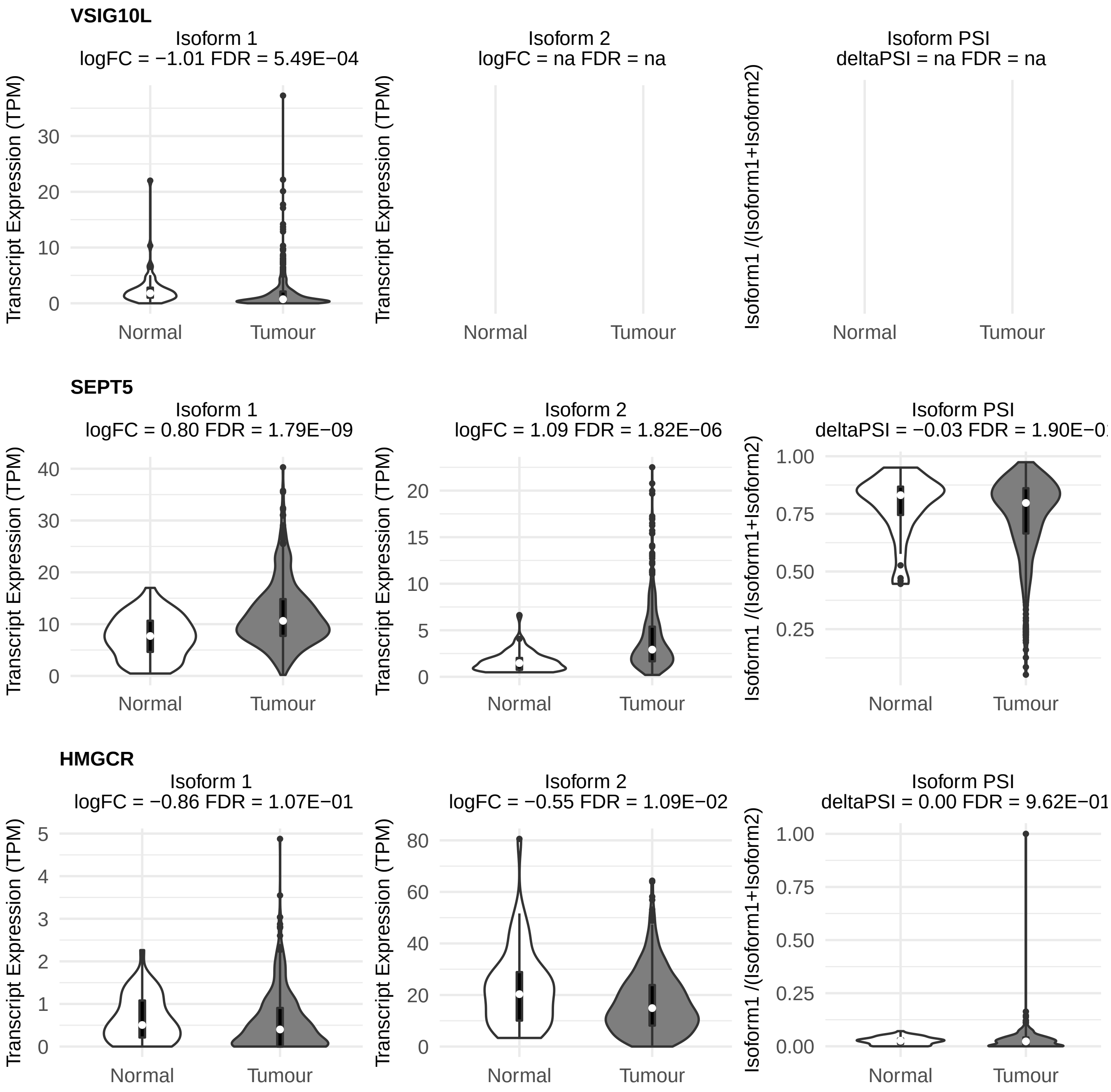

VSIG10L
Isoform 1
logFC = −1.01 FDR = 5.49E−04
Transcript Expression (TPM)
0
10
20
30
Normal
Tumour
Isoform 2
logFC = na FDR = na
Transcript Expression (TPM)
Normal
Tumour
Isoform PSI
deltaPSI = na FDR = na
Isoform1 /(Isoform1+Isoform2)
Normal
Tumour
SEPT5
Isoform 1
logFC = 0.80 FDR = 1.79E−09
Transcript Expression (TPM)
0
10
20
30
40
Normal
Tumour
Isoform 2
logFC = 1.09 FDR = 1.82E−06
Transcript Expression (TPM)
0
5
10
15
20
Normal
Tumour
Isoform PSI
deltaPSI = −0.03 FDR = 1.90E−0
Isoform1 /(Isoform1+Isoform2)
0.25
0.50
0.75
1.00
Normal
Tumour
HMGCR
Isoform 1
logFC = −0.86 FDR = 1.07E−01
Transcript Expression (TPM)
0
1
2
3
4
5
Normal
Tumour
Isoform 2
logFC = −0.55 FDR = 1.09E−02
Transcript Expression (TPM)
0
20
40
60
80
Normal
Tumour
Isoform PSI
deltaPSI = 0.00 FDR = 9.62E−01
Isoform1 /(Isoform1+Isoform2)
0.00
0.25
0.50
0.75
1.00
Normal
Tumour

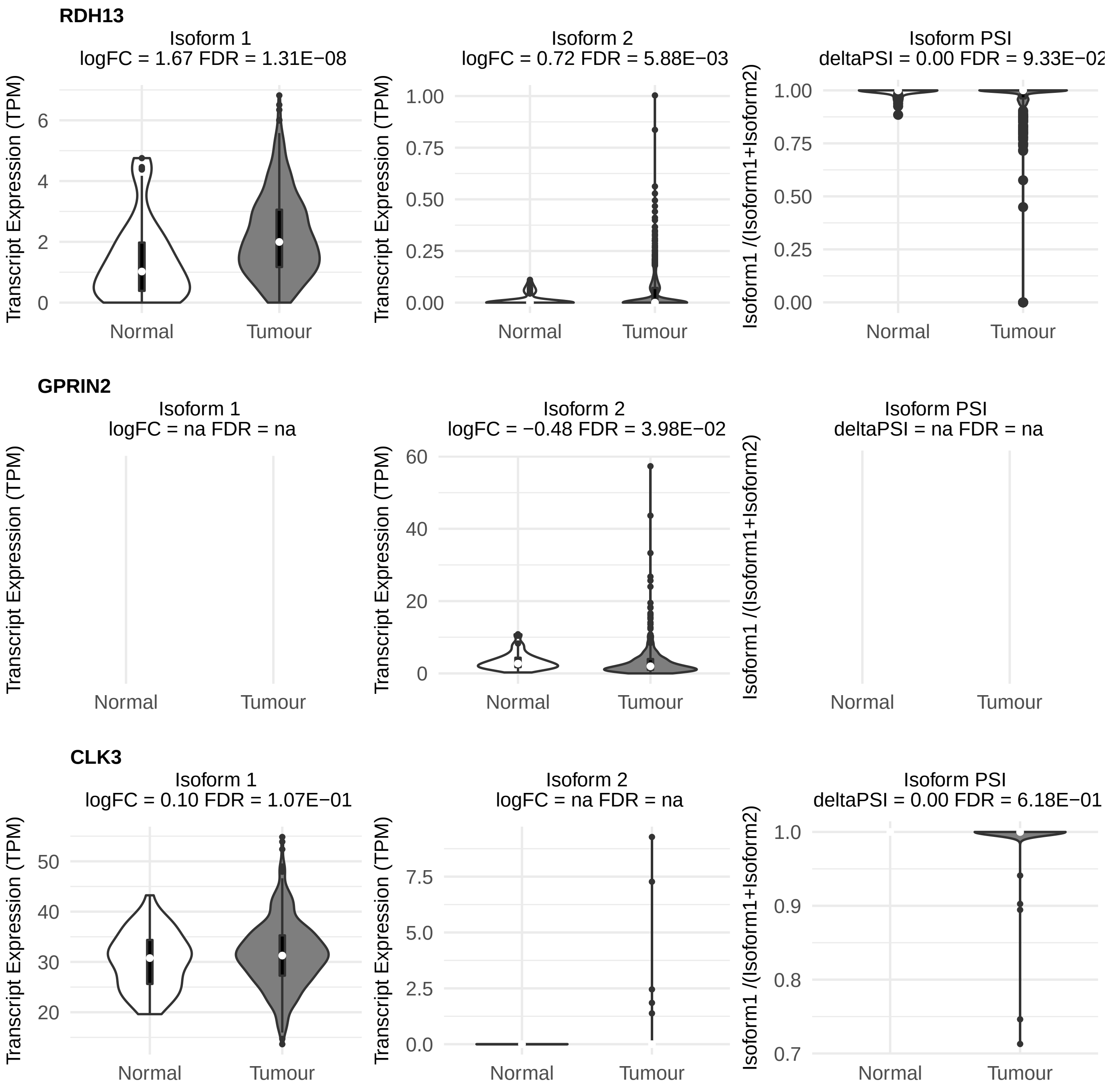
RDH13
Isoform 1
logFC = 1.67 FDR = 1.31E−08
Isoform 2
logFC = 0.72 FDR = 5.88E−03
Isoform PSI
deltaPSI = 0.00 FDR = 9.33E−02
GPRIN2
Isoform 1
logFC = na FDR = na
Isoform 2
logFC = −0.48 FDR = 3.98E−02
Isoform PSI
deltaPSI = na FDR = na
CLK3
Isoform 1
logFC = 0.10 FDR = 1.07E−01
Isoform 2
logFC = na FDR = na
Isoform PSI
deltaPSI = 0.00 FDR = 6.18E−01
Transcript Expression (TPM)
Isoform1 /(Isoform1+Isoform2)
Normal
Tumour

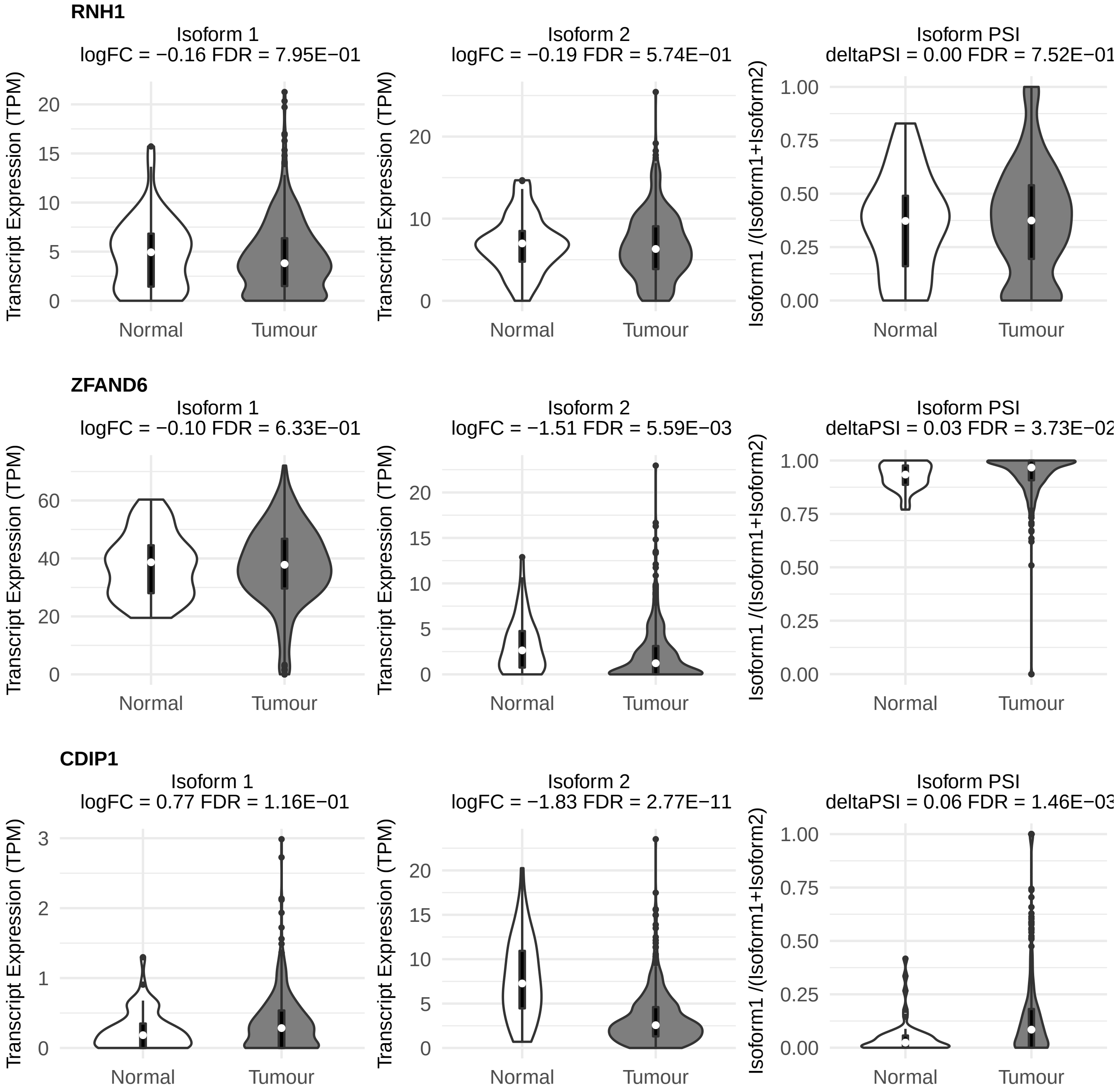
RNH1
Isoform 1
logFC = −0.16 FDR = 7.95E−01
Isoform 2
logFC = −0.19 FDR = 5.74E−01
Isoform PSI
deltaPSI = 0.00 FDR = 7.52E−01
ZFAND6
Isoform 1
logFC = −0.10 FDR = 6.33E−01
Isoform 2
logFC = −1.51 FDR = 5.59E−03
Isoform PSI
deltaPSI = 0.03 FDR = 3.73E−02
CDIP1
Isoform 1
logFC = 0.77 FDR = 1.16E−01
Isoform 2
logFC = −1.83 FDR = 2.77E−11
Isoform PSI
deltaPSI = 0.06 FDR = 1.46E−03
Transcript Expression (TPM)
Isoform1 /(Isoform1+Isoform2)
Normal
Tumour

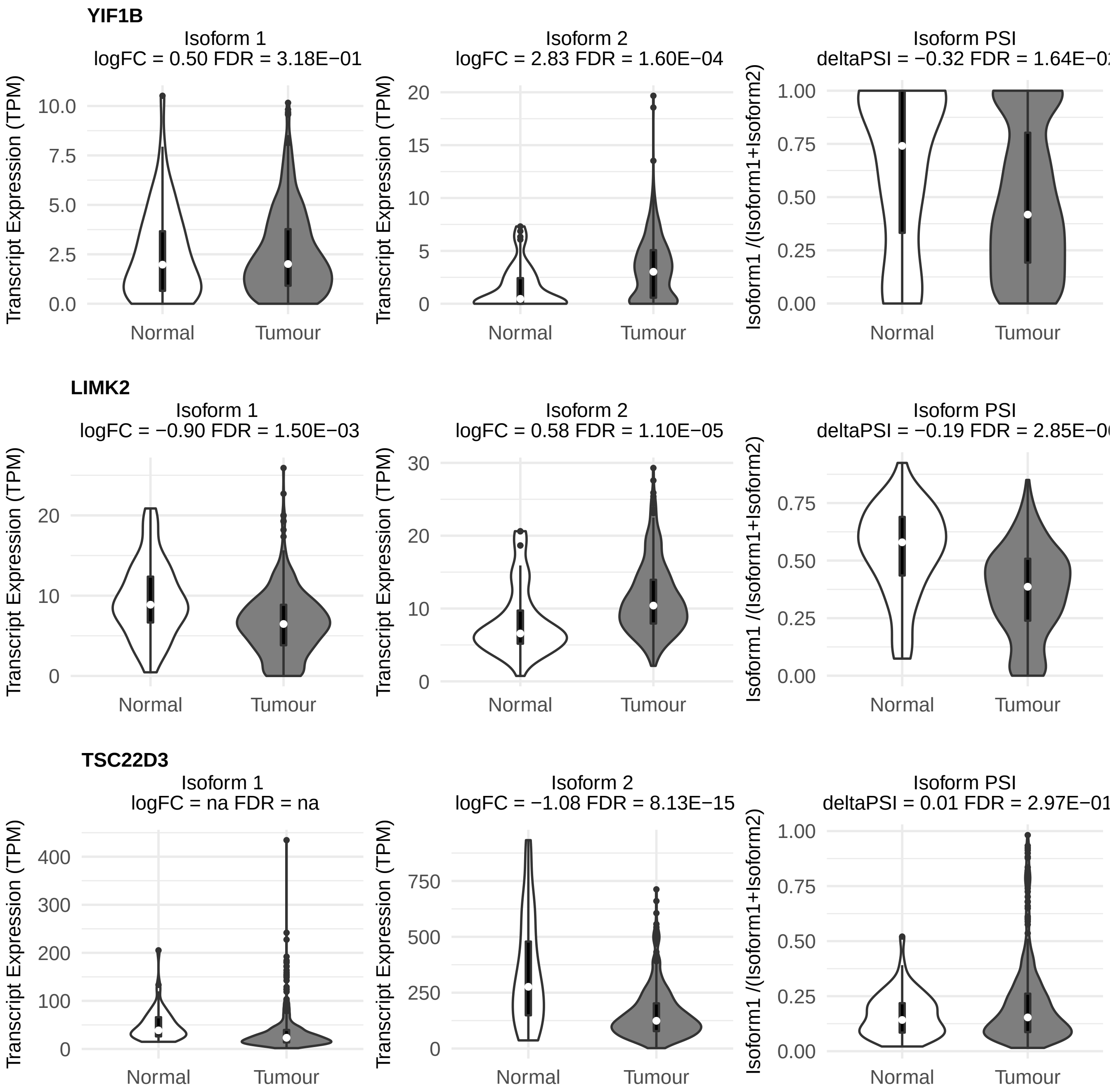

YIF1B
Isoform 1
logFC = 0.50 FDR = 3.18E−01
Isoform 2
logFC = 2.83 FDR = 1.60E−04
Isoform PSI
deltaPSI = −0.32 FDR = 1.64E−0
Transcript Expression (TPM)
Isoform1 /(Isoform1+Isoform2)
Normal
Tumour
LIMK2
Isoform 1
logFC = −0.90 FDR = 1.50E−03
Isoform 2
logFC = 0.58 FDR = 1.10E−05
Isoform PSI
deltaPSI = −0.19 FDR = 2.85E−0
TSC22D3
Isoform 1
logFC = na FDR = na
Isoform 2
logFC = −1.08 FDR = 8.13E−15
Isoform PSI
deltaPSI = 0.01 FDR = 2.97E−01

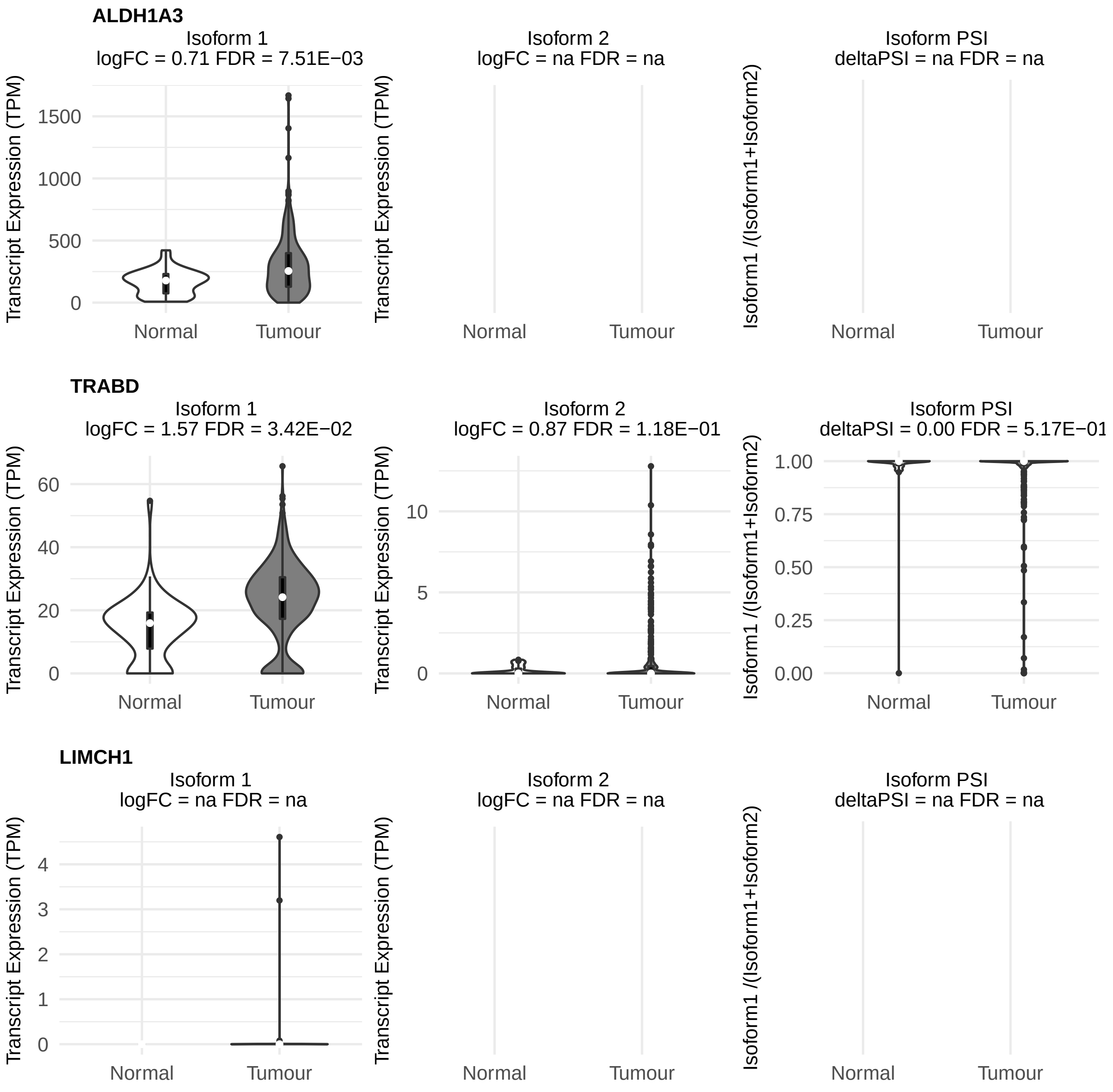

ALDH1A3
Isoform 1
logFC = 0.71 FDR = 7.51E−03
Transcript Expression (TPM)
1500
1000
500
0
Normal
Tumour
Isoform 2
logFC = na FDR = na
Transcript Expression (TPM)
Normal
Tumour
Isoform PSI
deltaPSI = na FDR = na
Isoform1 /(Isoform1+Isoform2)
Normal
Tumour
TRABD
Isoform 1
logFC = 1.57 FDR = 3.42E−02
Transcript Expression (TPM)
60
40
20
0
Normal
Tumour
Isoform 2
logFC = 0.87 FDR = 1.18E−01
Transcript Expression (TPM)
10
5
0
Normal
Tumour
Isoform PSI
deltaPSI = 0.00 FDR = 5.17E−01
Isoform1 /(Isoform1+Isoform2)
1.00
0.75
0.50
0.25
0.00
Normal
Tumour
LIMCH1
Isoform 1
logFC = na FDR = na
Transcript Expression (TPM)
4
3
2
1
0
Normal
Tumour
Isoform 2
logFC = na FDR = na
Transcript Expression (TPM)
Normal
Tumour
Isoform PSI
deltaPSI = na FDR = na
Isoform1 /(Isoform1+Isoform2)
Normal
Tumour

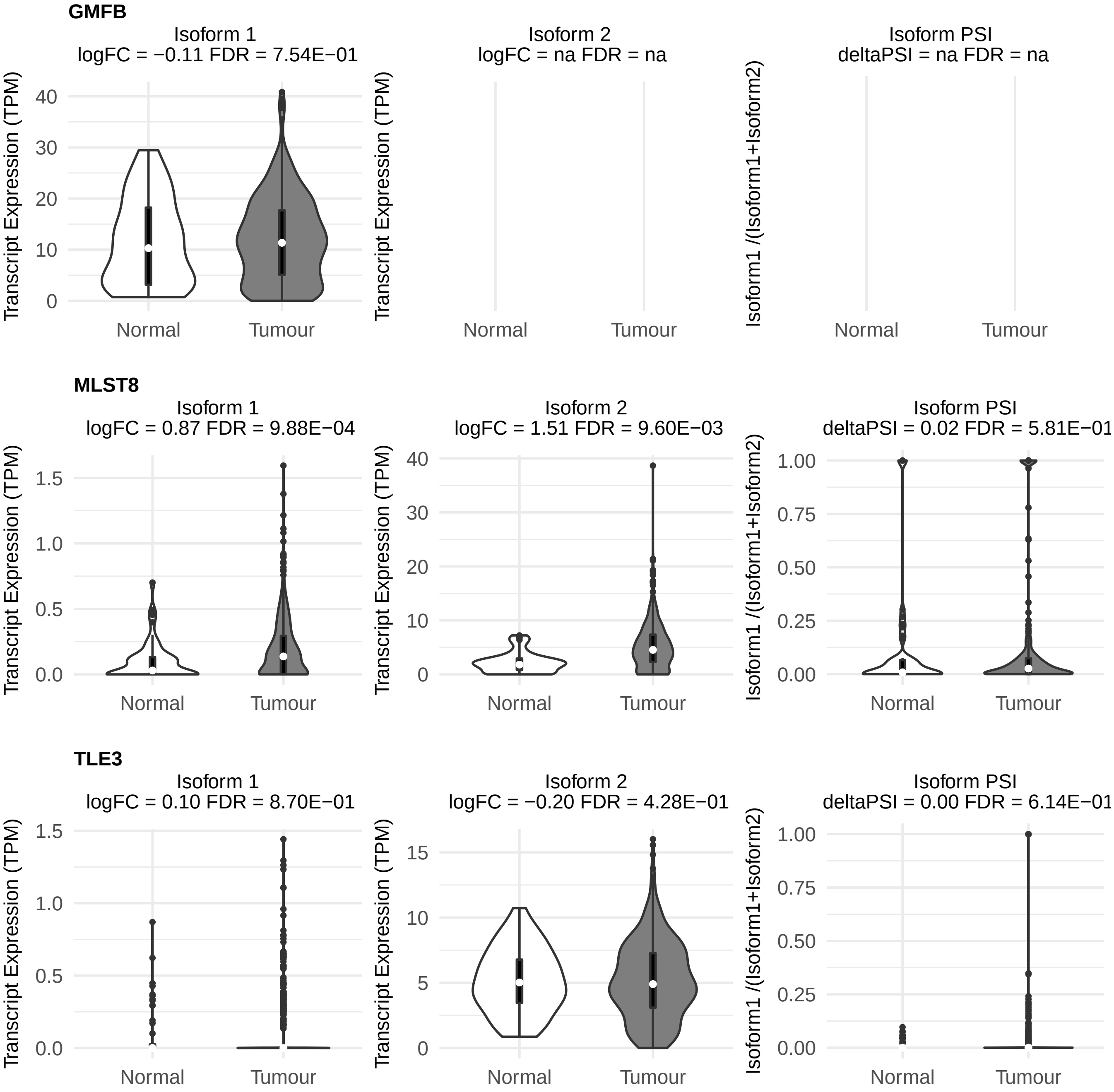
GMFB
Isoform 1
logFC = −0.11 FDR = 7.54E−01
Isoform 2
logFC = na FDR = na
Isoform PSI
deltaPSI = na FDR = na
Transcript Expression (TPM)
Isoform1 /(Isoform1+Isoform2)
Normal
Tumour
MLST8
Isoform 1
logFC = 0.87 FDR = 9.88E−04
Isoform 2
logFC = 1.51 FDR = 9.60E−03
Isoform PSI
deltaPSI = 0.02 FDR = 5.81E−01
TLE3
Isoform 1
logFC = 0.10 FDR = 8.70E−01
Isoform 2
logFC = −0.20 FDR = 4.28E−01
Isoform PSI
deltaPSI = 0.00 FDR = 6.14E−01

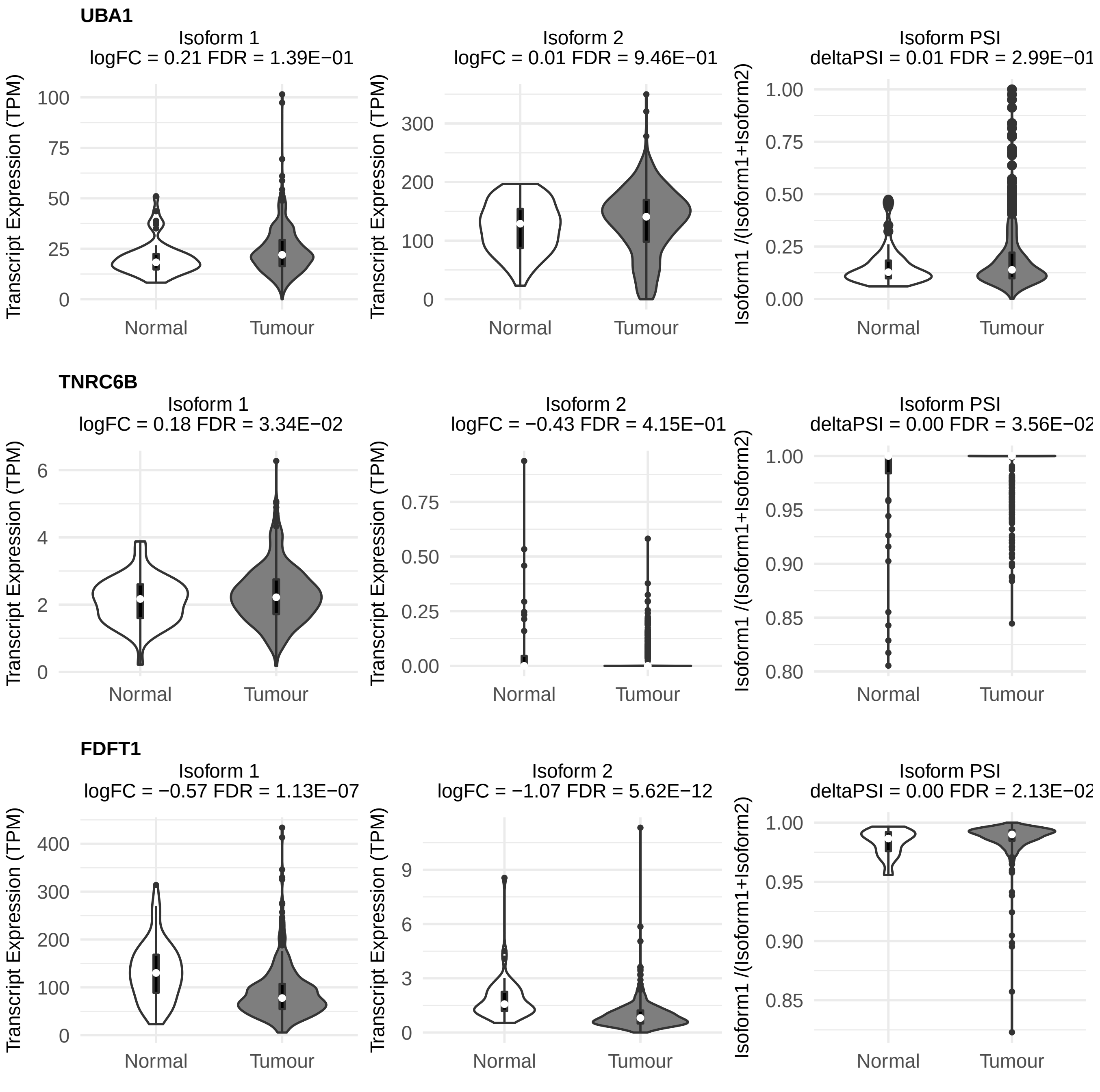
UBA1
Isoform 1
logFC = 0.21 FDR = 1.39E−01
Isoform 2
logFC = 0.01 FDR = 9.46E−01
Isoform PSI
deltaPSI = 0.01 FDR = 2.99E−01
TNRC6B
Isoform 1
logFC = 0.18 FDR = 3.34E−02
Isoform 2
logFC = −0.43 FDR = 4.15E−01
Isoform PSI
deltaPSI = 0.00 FDR = 3.56E−02
FDFT1
Isoform 1
logFC = −0.57 FDR = 1.13E−07
Isoform 2
logFC = −1.07 FDR = 5.62E−12
Isoform PSI
deltaPSI = 0.00 FDR = 2.13E−02
Transcript Expression (TPM)
Isoform1 /(Isoform1+Isoform2)
Normal
Tumour

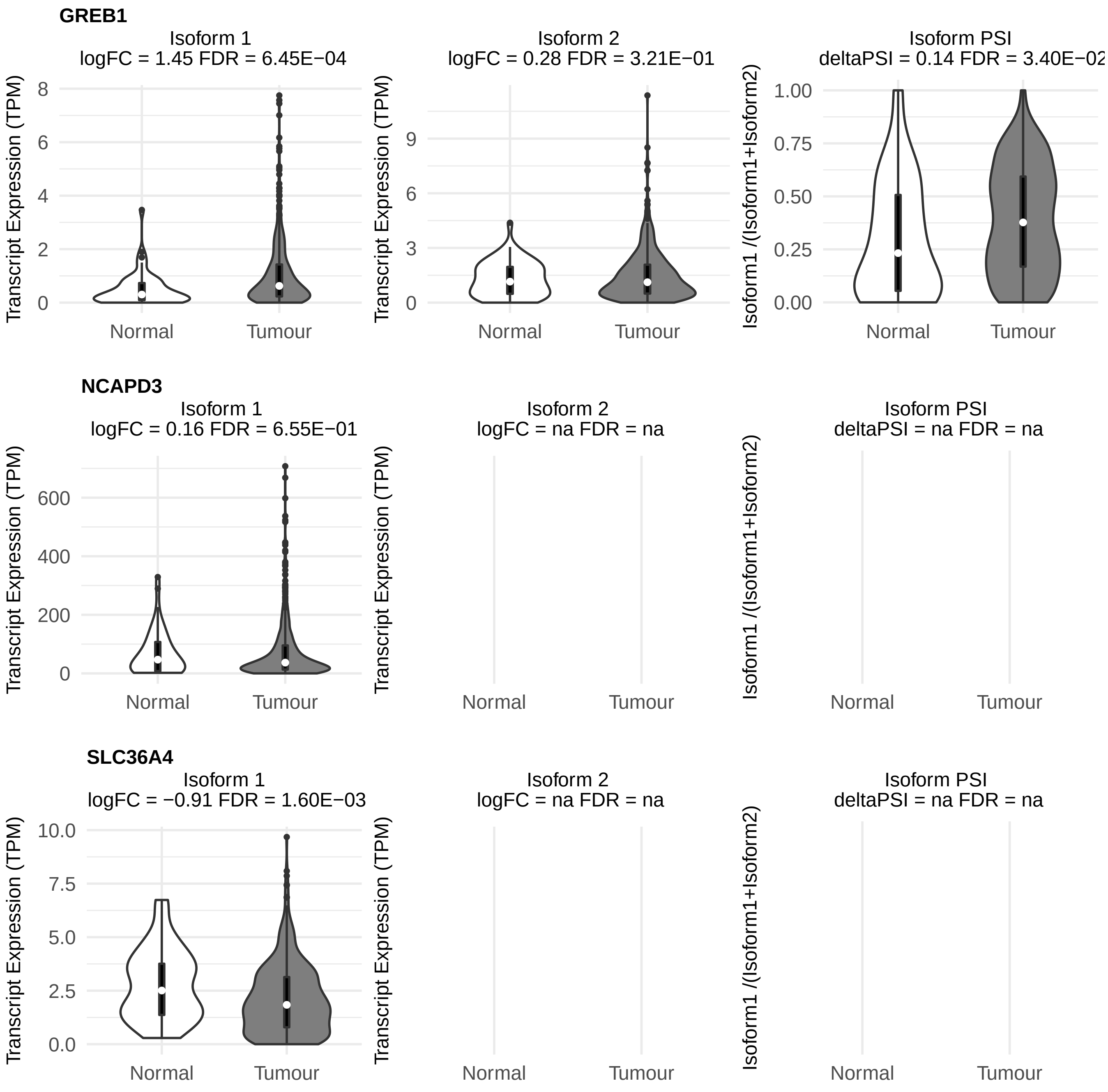

GREB1
Isoform 1
logFC = 1.45 FDR = 6.45E−04
Isoform 2
logFC = 0.28 FDR = 3.21E−01
Isoform PSI
deltaPSI = 0.14 FDR = 3.40E−02
Transcript Expression (TPM)
Isoform1 /(Isoform1+Isoform2)
Normal
Tumour
NCAPD3
Isoform 1
logFC = 0.16 FDR = 6.55E−01
Isoform 2
logFC = na FDR = na
Isoform PSI
deltaPSI = na FDR = na
SLC36A4
Isoform 1
logFC = −0.91 FDR = 1.60E−03
Isoform 2
logFC = na FDR = na
Isoform PSI
deltaPSI = na FDR = na

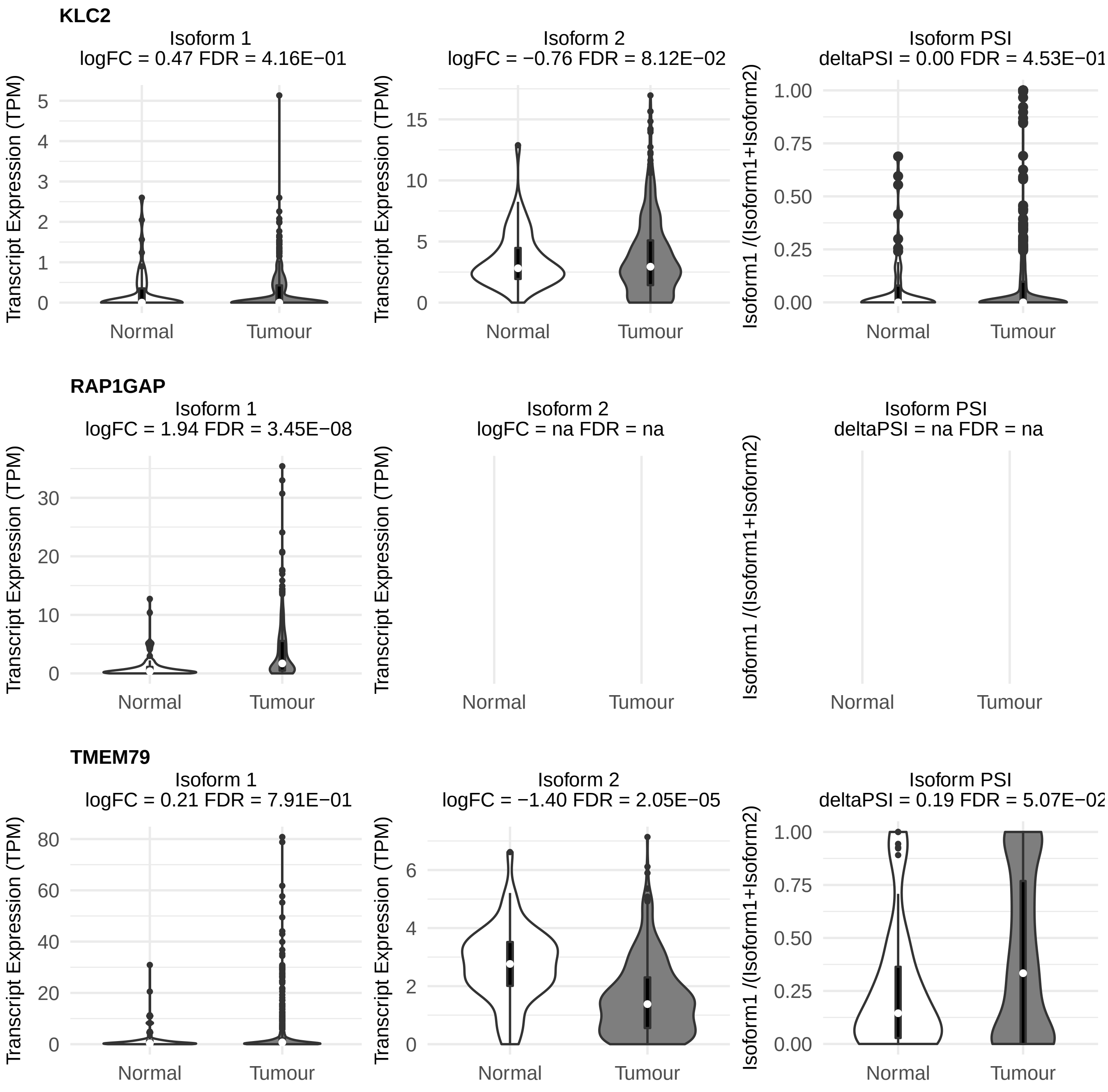

KLC2
Isoform 1
logFC = 0.47 FDR = 4.16E−01
Isoform 2
logFC = −0.76 FDR = 8.12E−02
Isoform PSI
deltaPSI = 0.00 FDR = 4.53E−01
Transcript Expression (TPM)
Isoform1 /(Isoform1+Isoform2)
Normal
Tumour
RAP1GAP
Isoform 1
logFC = 1.94 FDR = 3.45E−08
Isoform 2
logFC = na FDR = na
Isoform PSI
deltaPSI = na FDR = na
TMEM79
Isoform 1
logFC = 0.21 FDR = 7.91E−01
Isoform 2
logFC = −1.40 FDR = 2.05E−05
Isoform PSI
deltaPSI = 0.19 FDR = 5.07E−02

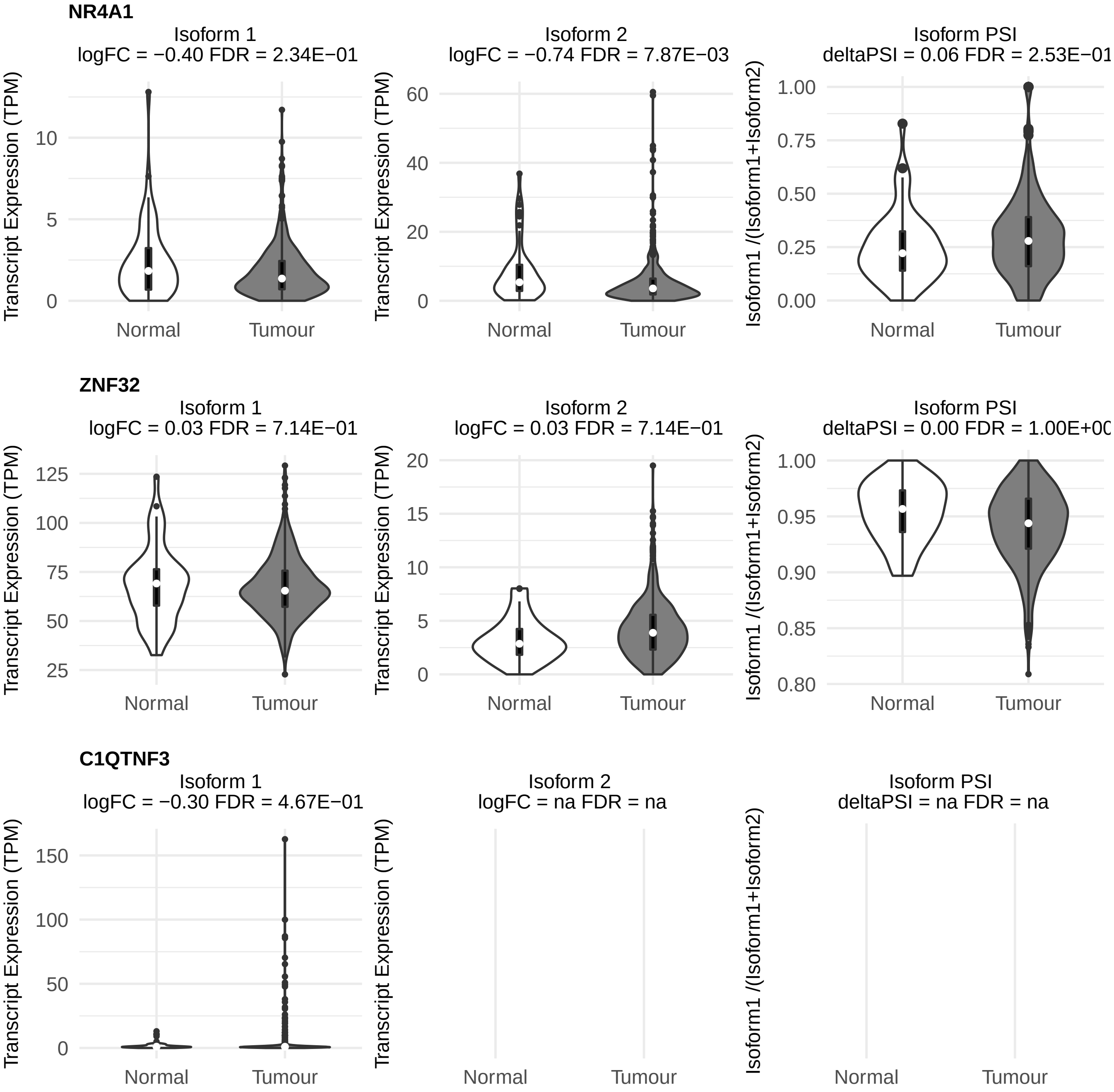

NR4A1
Isoform 1
logFC = −0.40 FDR = 2.34E−01
Isoform 2
logFC = −0.74 FDR = 7.87E−03
Isoform PSI
deltaPSI = 0.06 FDR = 2.53E−01
ZNF32
Isoform 1
logFC = 0.03 FDR = 7.14E−01
Isoform 2
logFC = 0.03 FDR = 7.14E−01
Isoform PSI
deltaPSI = 0.00 FDR = 1.00E+00
C1QTNF3
Isoform 1
logFC = −0.30 FDR = 4.67E−01
Isoform 2
logFC = na FDR = na
Isoform PSI
deltaPSI = na FDR = na
Transcript Expression (TPM)
Isoform1 /(Isoform1+Isoform2)
Normal
Tumour

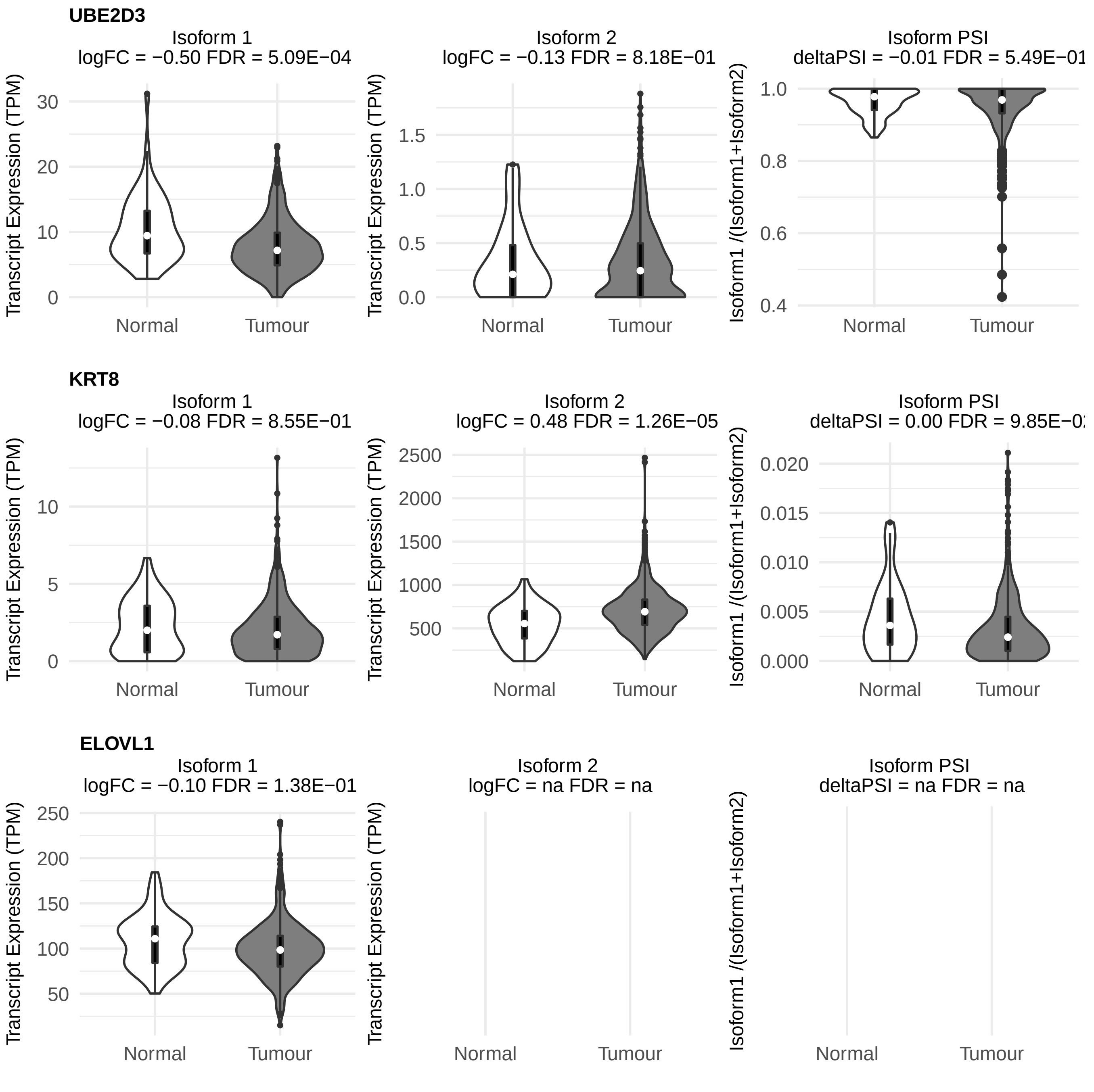

UBE2D3
Isoform 1
logFC = −0.50 FDR = 5.09E−04
Isoform 2
logFC = −0.13 FDR = 8.18E−01
Isoform PSI
deltaPSI = −0.01 FDR = 5.49E−01
KRT8
Isoform 1
logFC = −0.08 FDR = 8.55E−01
Isoform 2
logFC = 0.48 FDR = 1.26E−05
Isoform PSI
deltaPSI = 0.00 FDR = 9.85E−0
ELOVL1
Isoform 1
logFC = −0.10 FDR = 1.38E−01
Isoform 2
logFC = na FDR = na
Isoform PSI
deltaPSI = na FDR = na
Transcript Expression (TPM)
Isoform1 /(Isoform1+Isoform2)
Normal
Tumour

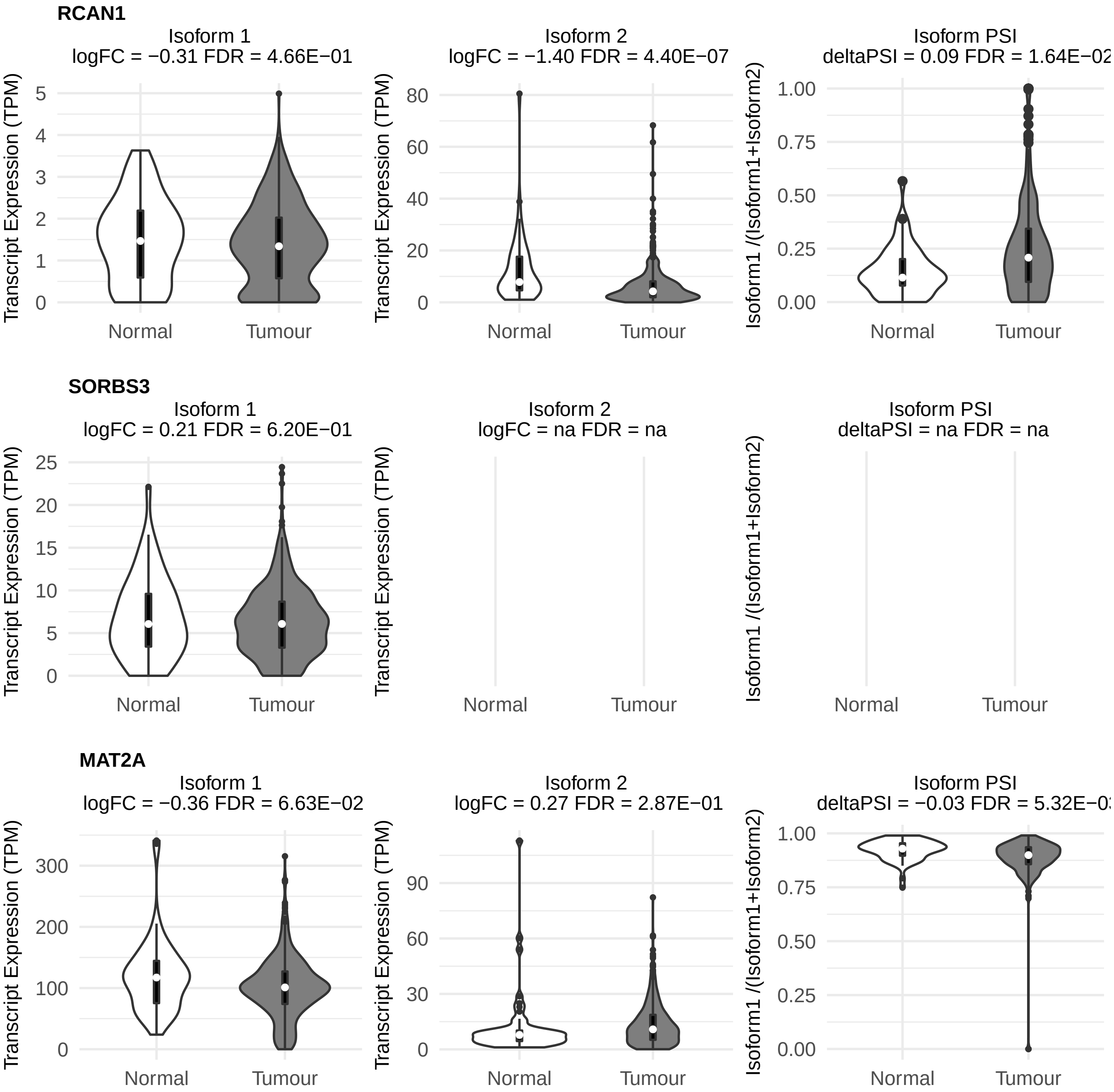

RCAN1
Isoform 1
logFC = −0.31 FDR = 4.66E−01
Isoform 2
logFC = −1.40 FDR = 4.40E−07
Isoform PSI
deltaPSI = 0.09 FDR = 1.64E−02
Transcript Expression (TPM)
Isoform1 /(Isoform1+Isoform2)
Normal
Tumour
SORBS3
Isoform 1
logFC = 0.21 FDR = 6.20E−01
Isoform 2
logFC = na FDR = na
Isoform PSI
deltaPSI = na FDR = na
MAT2A
Isoform 1
logFC = −0.36 FDR = 6.63E−02
Isoform 2
logFC = 0.27 FDR = 2.87E−01
Isoform PSI
deltaPSI = −0.03 FDR = 5.32E−0

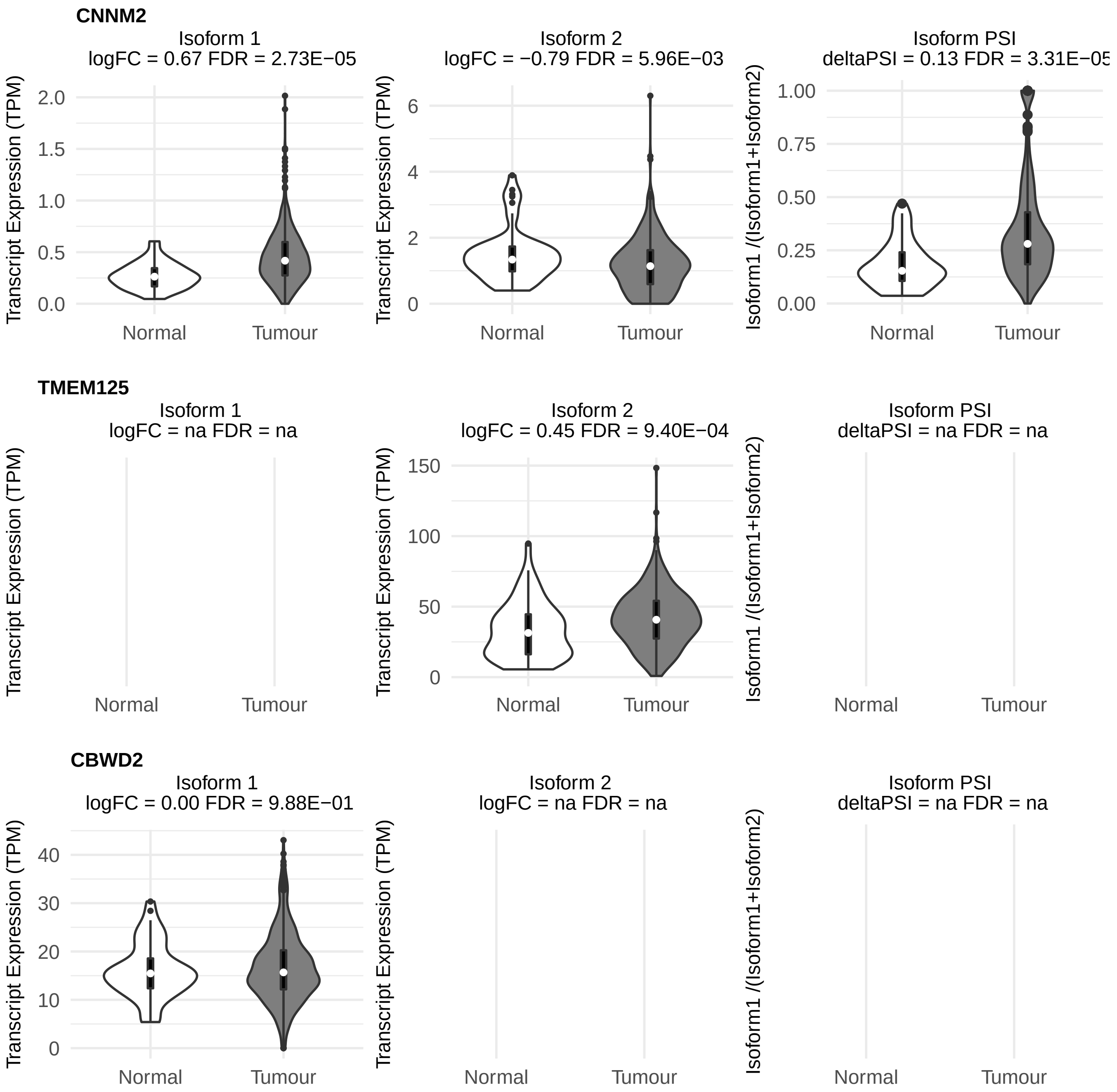

CNNM2
Isoform 1
logFC = 0.67 FDR = 2.73E−05
Isoform 2
logFC = −0.79 FDR = 5.96E−03
Isoform PSI
deltaPSI = 0.13 FDR = 3.31E−05
Transcript Expression (TPM)
Isoform1 /(Isoform1+Isoform2)
Normal
Tumour
TMEM125
Isoform 1
logFC = na FDR = na
Isoform 2
logFC = 0.45 FDR = 9.40E−04
Isoform PSI
deltaPSI = na FDR = na
CBWD2
Isoform 1
logFC = 0.00 FDR = 9.88E−01
Isoform 2
logFC = na FDR = na
Isoform PSI
deltaPSI = na FDR = na

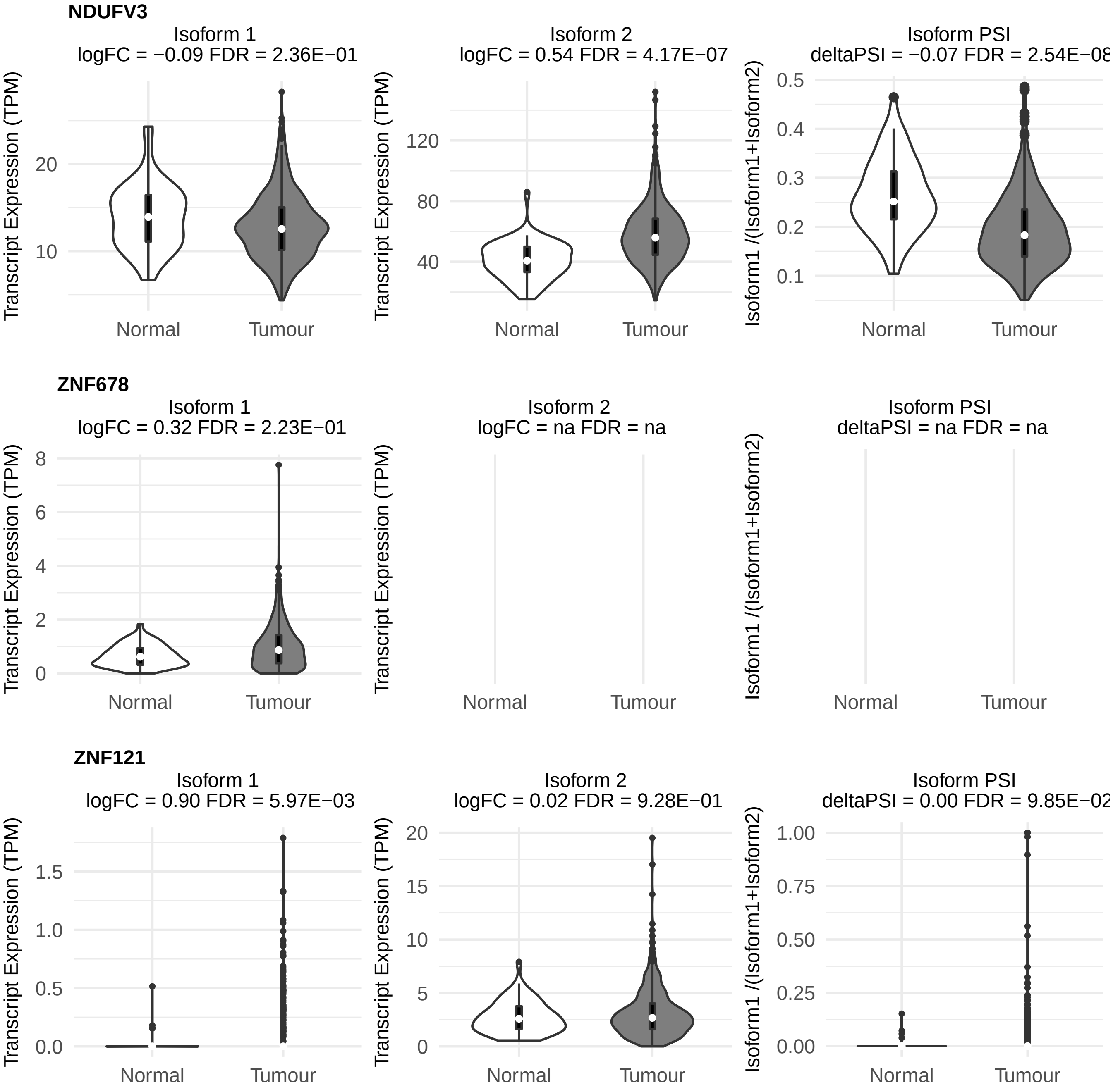
NDUFV3
Isoform 1
logFC = −0.09 FDR = 2.36E−01
Isoform 2
logFC = 0.54 FDR = 4.17E−07
Isoform PSI
deltaPSI = −0.07 FDR = 2.54E−08
Transcript Expression (TPM)
Isoform1 /(Isoform1+Isoform2)
Normal
Tumour
ZNF678
Isoform 1
logFC = 0.32 FDR = 2.23E−01
Isoform 2
logFC = na FDR = na
Isoform PSI
deltaPSI = na FDR = na
ZNF121
Isoform 1
logFC = 0.90 FDR = 5.97E−03
Isoform 2
logFC = 0.02 FDR = 9.28E−01
Isoform PSI
deltaPSI = 0.00 FDR = 9.85E−02

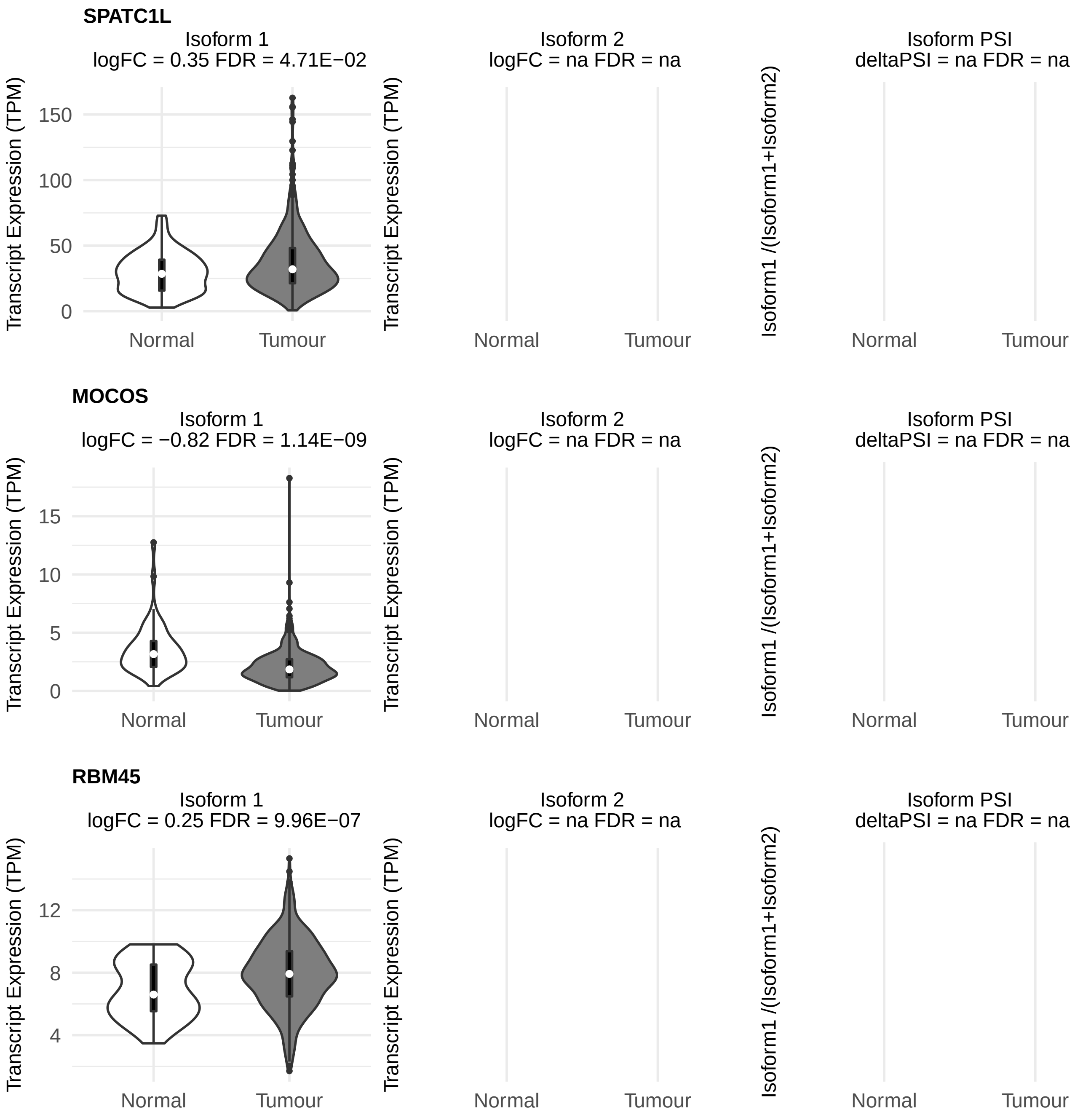
SPATC1L
Isoform 1
logFC = 0.35 FDR = 4.71E−02
Isoform 2
logFC = na FDR = na
Isoform PSI
deltaPSI = na FDR = na
Transcript Expression (TPM)
Isoform1 /(Isoform1+Isoform2)
Normal
Tumour
MOCOS
Isoform 1
logFC = −0.82 FDR = 1.14E−09
Isoform 2
logFC = na FDR = na
Isoform PSI
deltaPSI = na FDR = na
RBM45
Isoform 1
logFC = 0.25 FDR = 9.96E−07
Isoform 2
logFC = na FDR = na
Isoform PSI
deltaPSI = na FDR = na

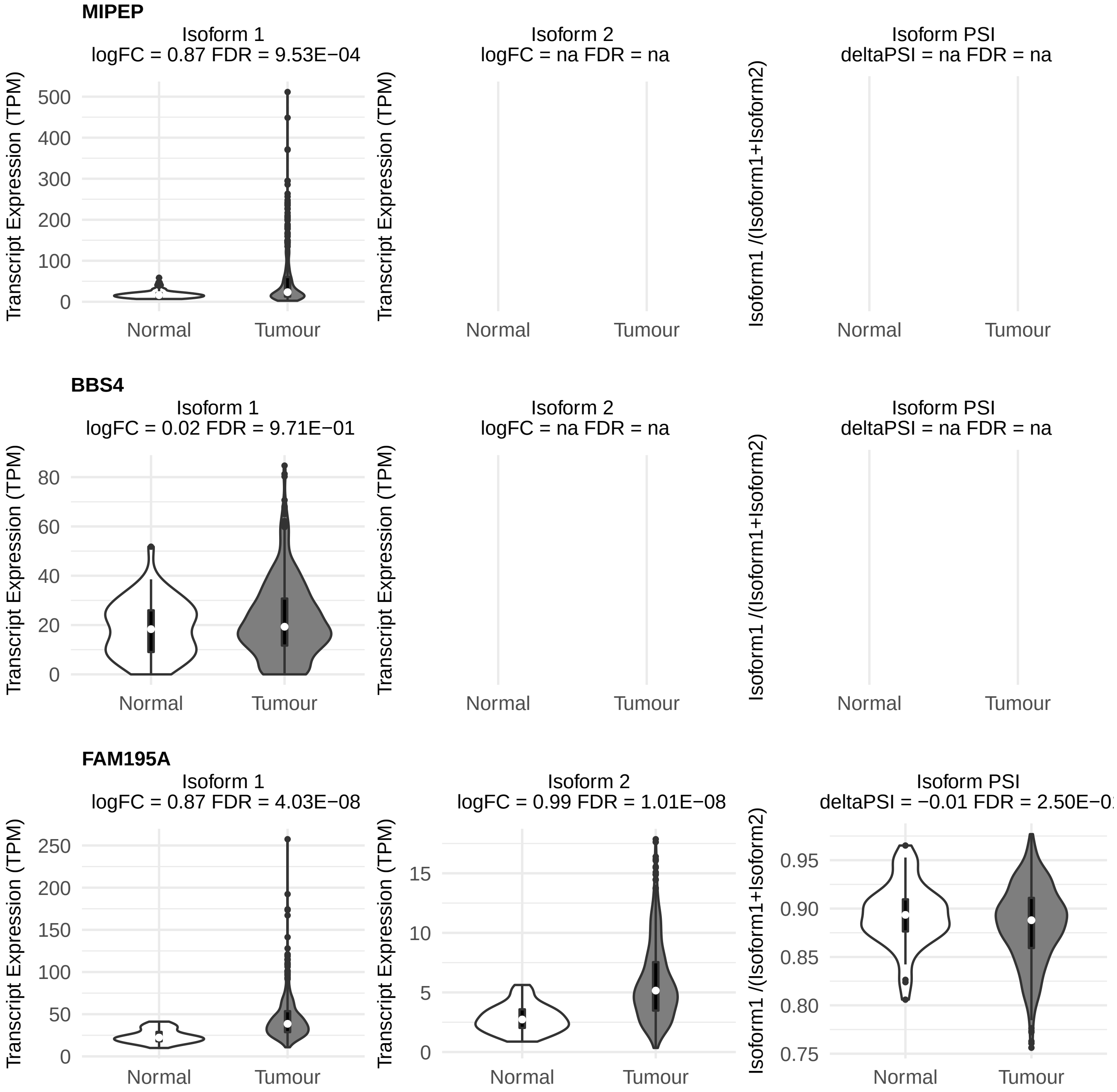
MIPEP
Isoform 1
logFC = 0.87 FDR = 9.53E−04
Isoform 2
logFC = na FDR = na
Isoform PSI
deltaPSI = na FDR = na
Transcript Expression (TPM)
Isoform1 /(Isoform1+Isoform2)
500
400
300
200
100
0
Normal
Tumour
BBS4
Isoform 1
logFC = 0.02 FDR = 9.71E−01
Isoform 2
logFC = na FDR = na
Isoform PSI
deltaPSI = na FDR = na
80
60
40
20
0
FAM195A
Isoform 1
logFC = 0.87 FDR = 4.03E−08
Isoform 2
logFC = 0.99 FDR = 1.01E−08
Isoform PSI
250
200
150
100
50
0
15
10
5
0.95
0.90
0.85
0.80
0.75

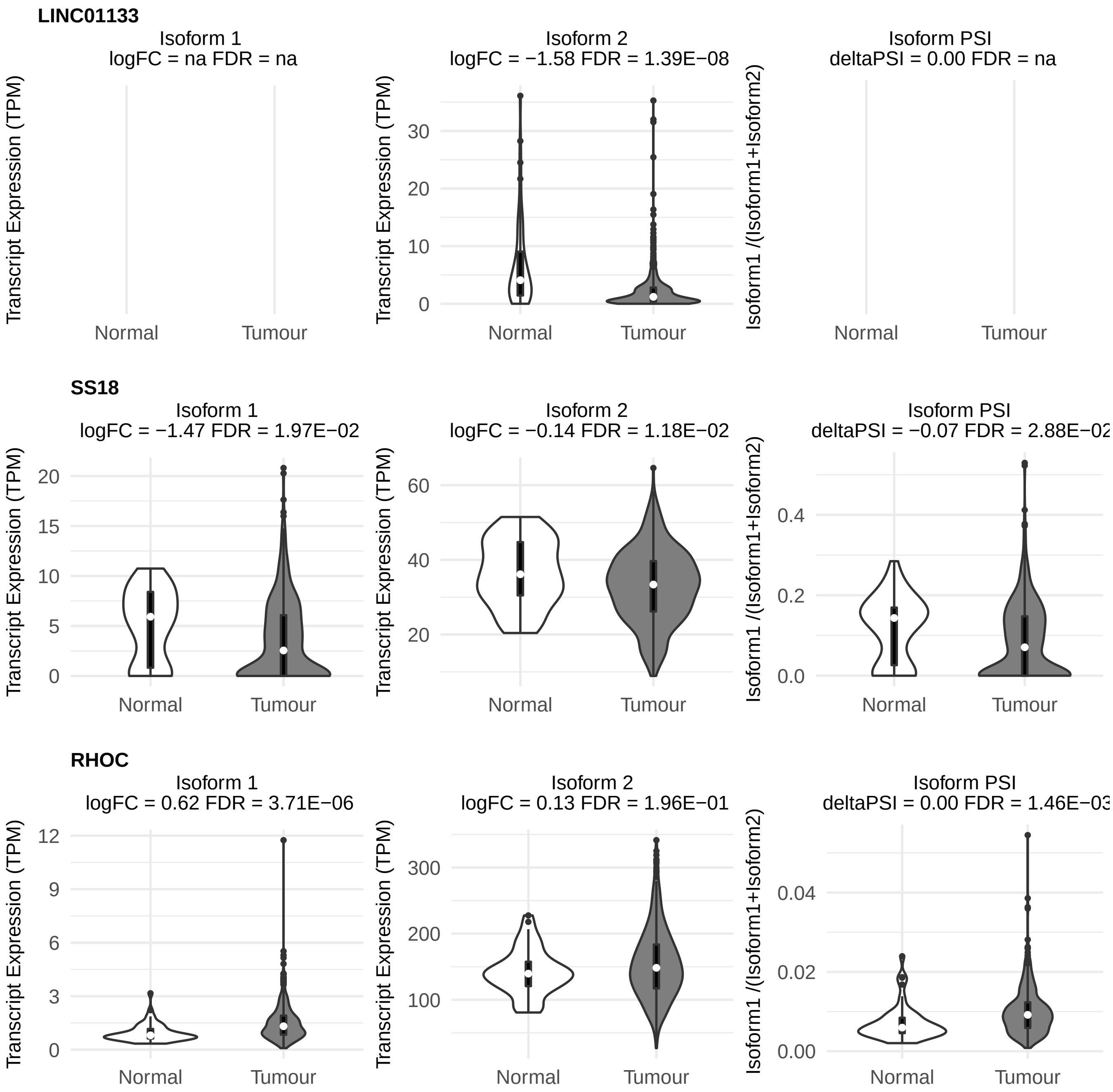

LINC01133
Isoform 1
logFC = na FDR = na
Isoform 2
logFC = −1.58 FDR = 1.39E−08
Isoform PSI
deltaPSI = 0.00 FDR = na
SS18
Isoform 1
logFC = −1.47 FDR = 1.97E−02
Isoform 2
logFC = −0.14 FDR = 1.18E−02
Isoform PSI
deltaPSI = −0.07 FDR = 2.88E−02
RHOC
Isoform 1
logFC = 0.62 FDR = 3.71E−06
Isoform 2
logFC = 0.13 FDR = 1.96E−01
Isoform PSI
deltaPSI = 0.00 FDR = 1.46E−03
Transcript Expression (TPM)
Isoform1 /(Isoform1+Isoform2)
Normal
Tumour

ZNF226

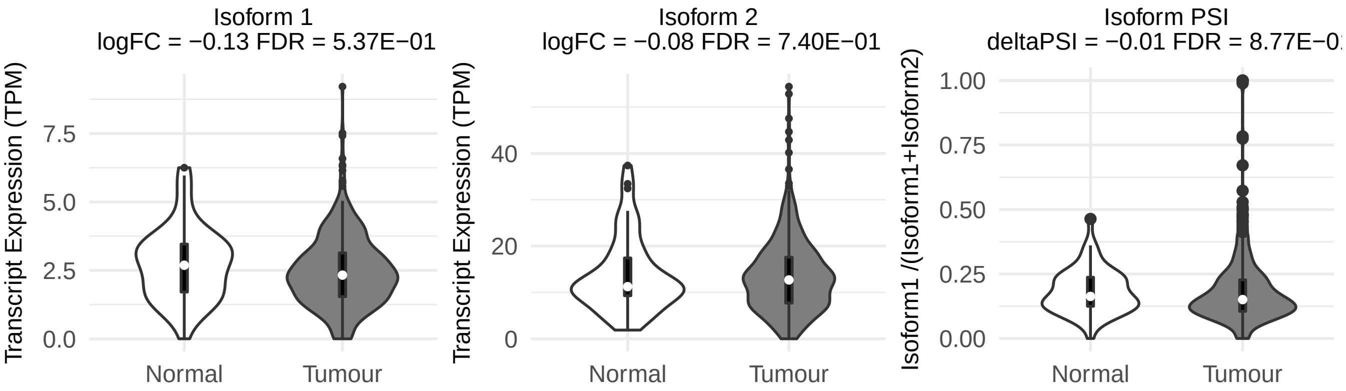
Isoform 1
logFC = −0.13 FDR = 5.37E−01
Transcript Expression (TPM)
0.0
2.5
5.0
7.5
Normal
Tumour
Isoform 2
logFC = −0.08 FDR = 7.40E−01
Transcript Expression (TPM)
0
20
40
Normal
Tumour
Isoform PSI
deltaPSI = −0.01 FDR = 8.77E−0
Isoform1 /(Isoform1+Isoform2)
0.00
0.25
0.50
0.75
1.00
Normal
Tumour
