# Supplementary material for: Androgen-dependent alternative mRNA isoform expression in prostate cancer cells
Source: F1000Res. 2018 Aug 3;7:1189. [Version 1] doi: 10.12688/f1000research.15604.1 (PMC6143958; doi:10.12688/f1000research.15604.1)
Supplement: Supplementary file 7 [file f1000research-7-17022-s0006.tgz › d4596cf7-6b7e-4f38-8be3-019b7570dc1f.pdf]

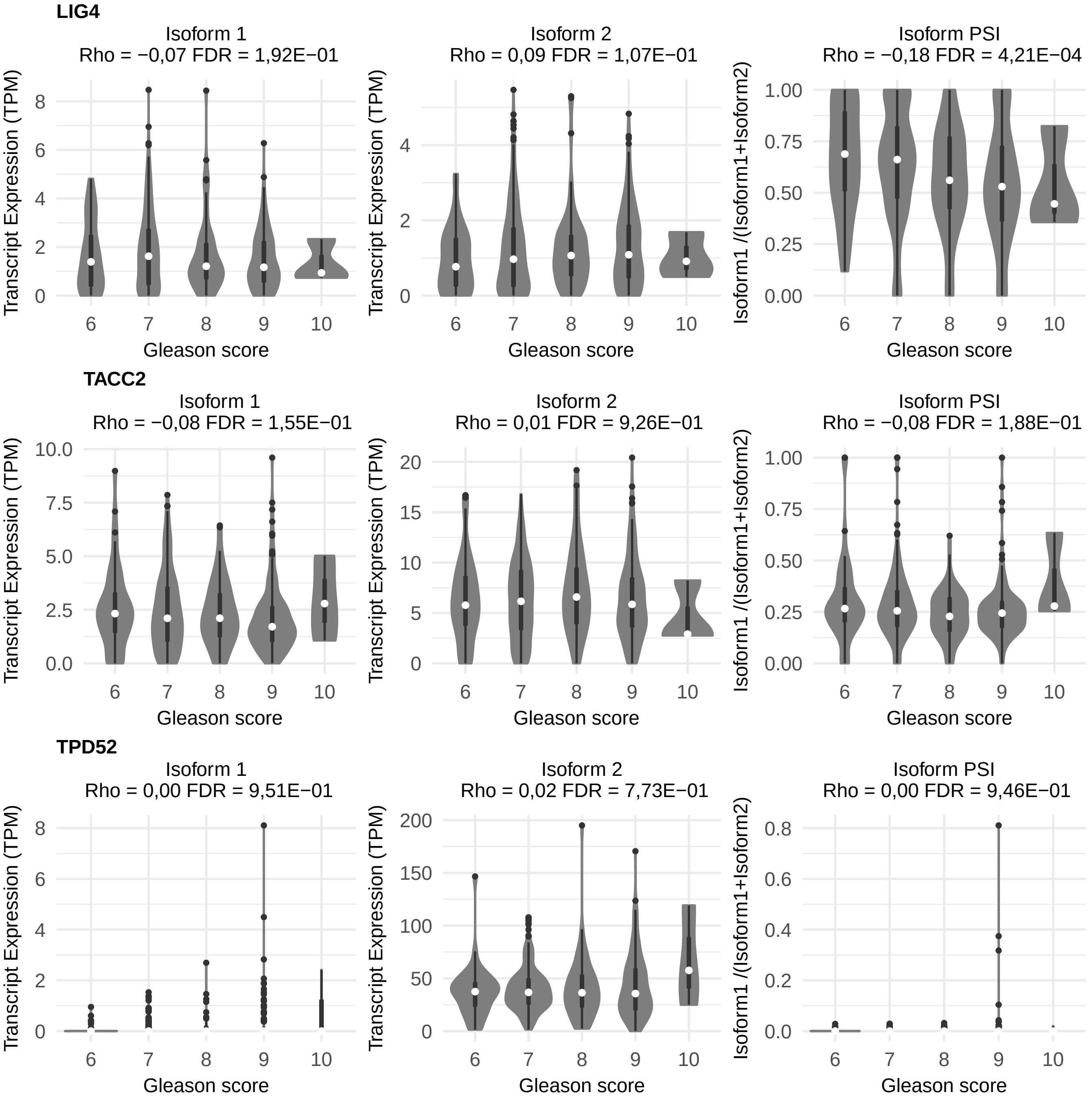
LIG4
Isoform 1
Rho = −0,07 FDR = 1,92E−01
Isoform 2
Rho = 0,09 FDR = 1,07E−01
Isoform PSI
Rho = −0,18 FDR = 4,21E−04
TACC2
Isoform 1
Rho = −0,08 FDR = 1,55E−01
Isoform 2
Rho = 0,01 FDR = 9,26E−01
Isoform PSI
Rho = −0,08 FDR = 1,88E−01
TPD52
Isoform 1
Rho = 0,00 FDR = 9,51E−01
Isoform 2
Rho = 0,02 FDR = 7,73E−01
Isoform PSI
Rho = 0,00 FDR = 9,46E−01
Transcript Expression (TPM)
Isoform1 /(Isoform1+Isoform2)
Gleason score
6
7
8
9
10

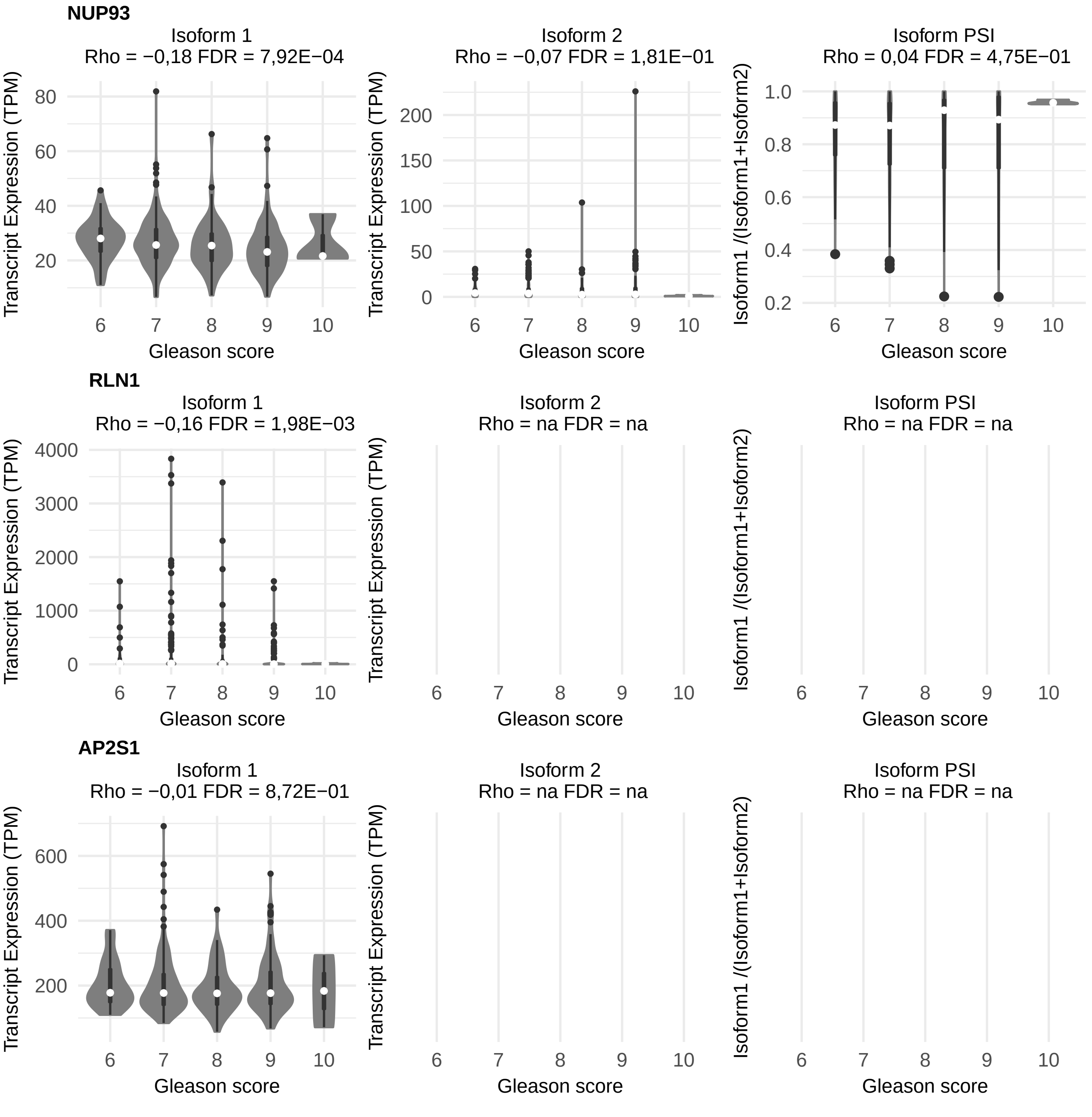
NUP93
Isoform 1
Rho = −0,18 FDR = 7,92E−04
Isoform 2
Rho = −0,07 FDR = 1,81E−01
Isoform PSI
Rho = 0,04 FDR = 4,75E−01
RLN1
Isoform 1
Rho = −0,16 FDR = 1,98E−03
Isoform 2
Rho = na FDR = na
Isoform PSI
Rho = na FDR = na
AP2S1
Isoform 1
Rho = −0,01 FDR = 8,72E−01
Isoform 2
Rho = na FDR = na
Isoform PSI
Rho = na FDR = na
Transcript Expression (TPM)
Isoform1 /(Isoform1+Isoform2)
Gleason score

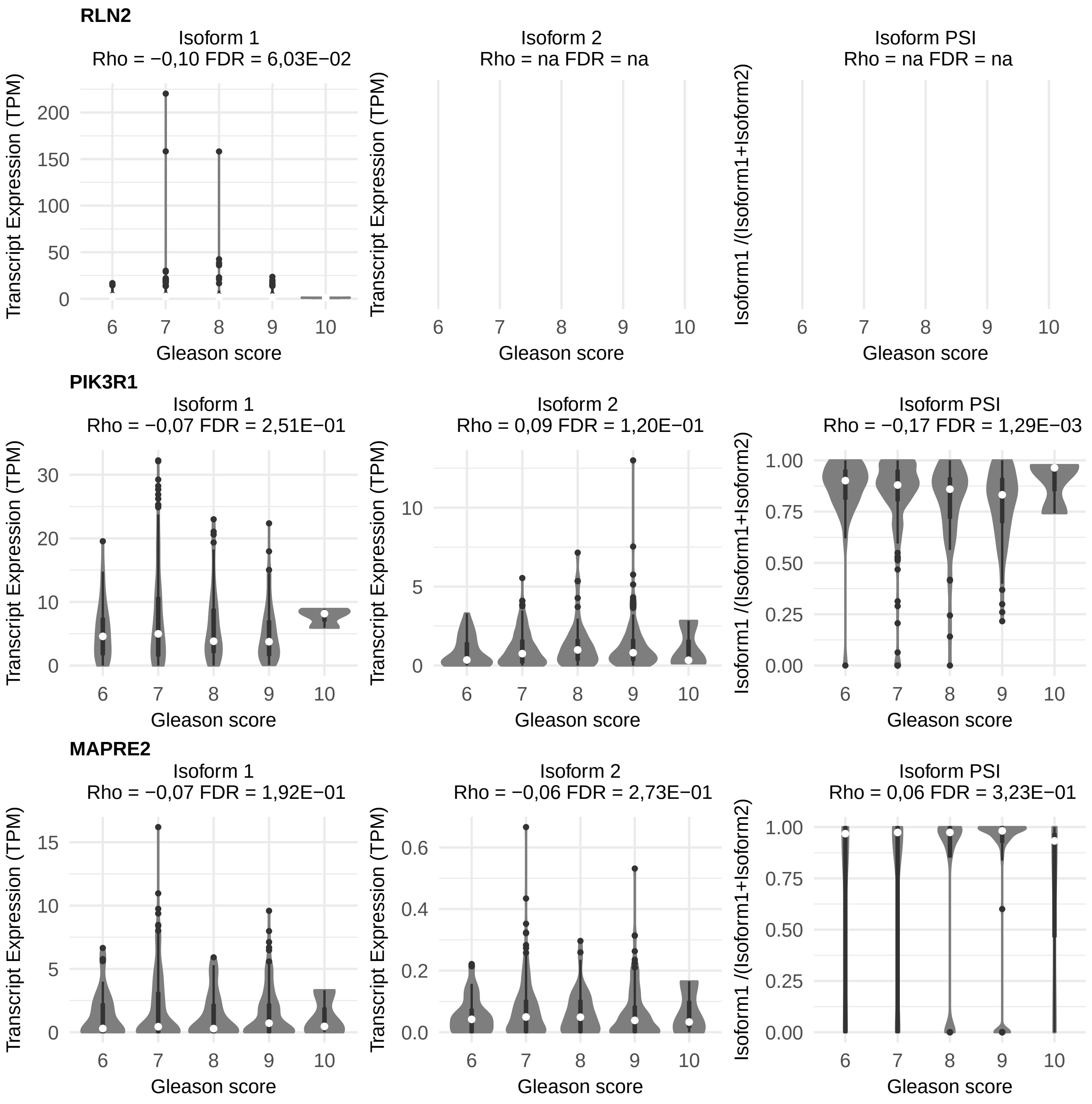
RLN2
Isoform 1
Rho = −0,10 FDR = 6,03E−02
Isoform 2
Rho = na FDR = na
Isoform PSI
Rho = na FDR = na
PIK3R1
Isoform 1
Rho = −0,07 FDR = 2,51E−01
Isoform 2
Rho = 0,09 FDR = 1,20E−01
Isoform PSI
Rho = −0,17 FDR = 1,29E−03
MAPRE2
Isoform 1
Rho = −0,07 FDR = 1,92E−01
Isoform 2
Rho = −0,06 FDR = 2,73E−01
Isoform PSI
Rho = 0,06 FDR = 3,23E−01
Transcript Expression (TPM)
Isoform1 /(Isoform1+Isoform2)
Gleason score
6
7
8
9
10
0
50
100
150
200
10
20
30
5
15
0.0
0.2
0.4
0.6
0.00
0.25
0.50
0.75
1.00

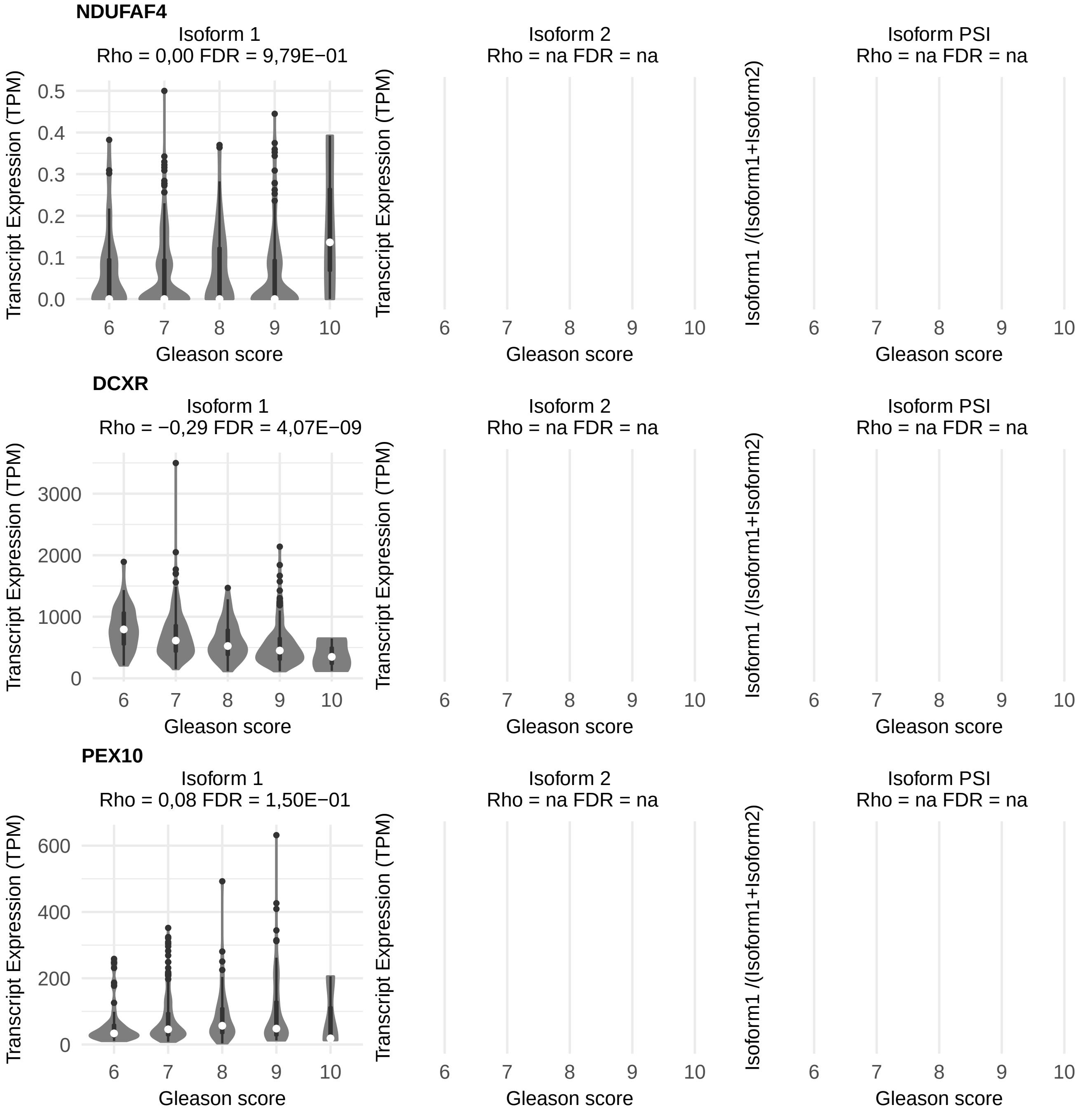

NDUFAF4
Isoform 1
Rho = 0,00 FDR = 9,79E−01
Isoform 2
Rho = na FDR = na
Isoform PSI
Rho = na FDR = na
DCXR
Isoform 1
Rho = −0,29 FDR = 4,07E−09
Isoform 2
Rho = na FDR = na
Isoform PSI
Rho = na FDR = na
PEX10
Isoform 1
Rho = 0,08 FDR = 1,50E−01
Isoform 2
Rho = na FDR = na
Isoform PSI
Rho = na FDR = na
Transcript Expression (TPM)
Isoform1 /(Isoform1+Isoform2)
Gleason score

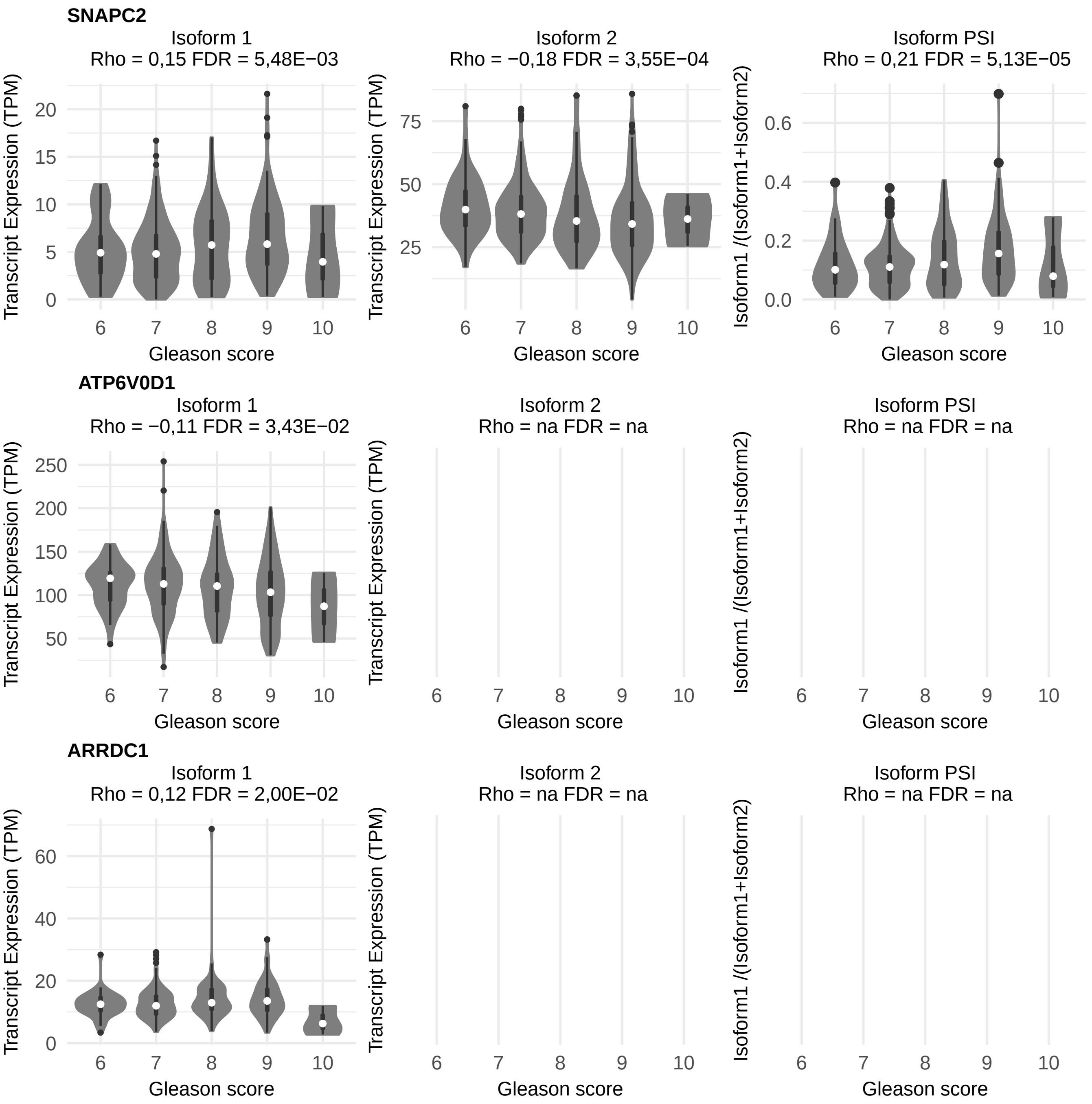
SNAPC2
Isoform 1
Rho = 0,15 FDR = 5,48E−03
Isoform 2
Rho = −0,18 FDR = 3,55E−04
Isoform PSI
Rho = 0,21 FDR = 5,13E−05
ATP6V0D1
Isoform 1
Rho = −0,11 FDR = 3,43E−02
Isoform 2
Rho = na FDR = na
Isoform PSI
Rho = na FDR = na
ARRDC1
Isoform 1
Rho = 0,12 FDR = 2,00E−02
Isoform 2
Rho = na FDR = na
Isoform PSI
Rho = na FDR = na
Transcript Expression (TPM)
Isoform1 /(Isoform1+Isoform2)
Gleason score
6
7
8
9
10

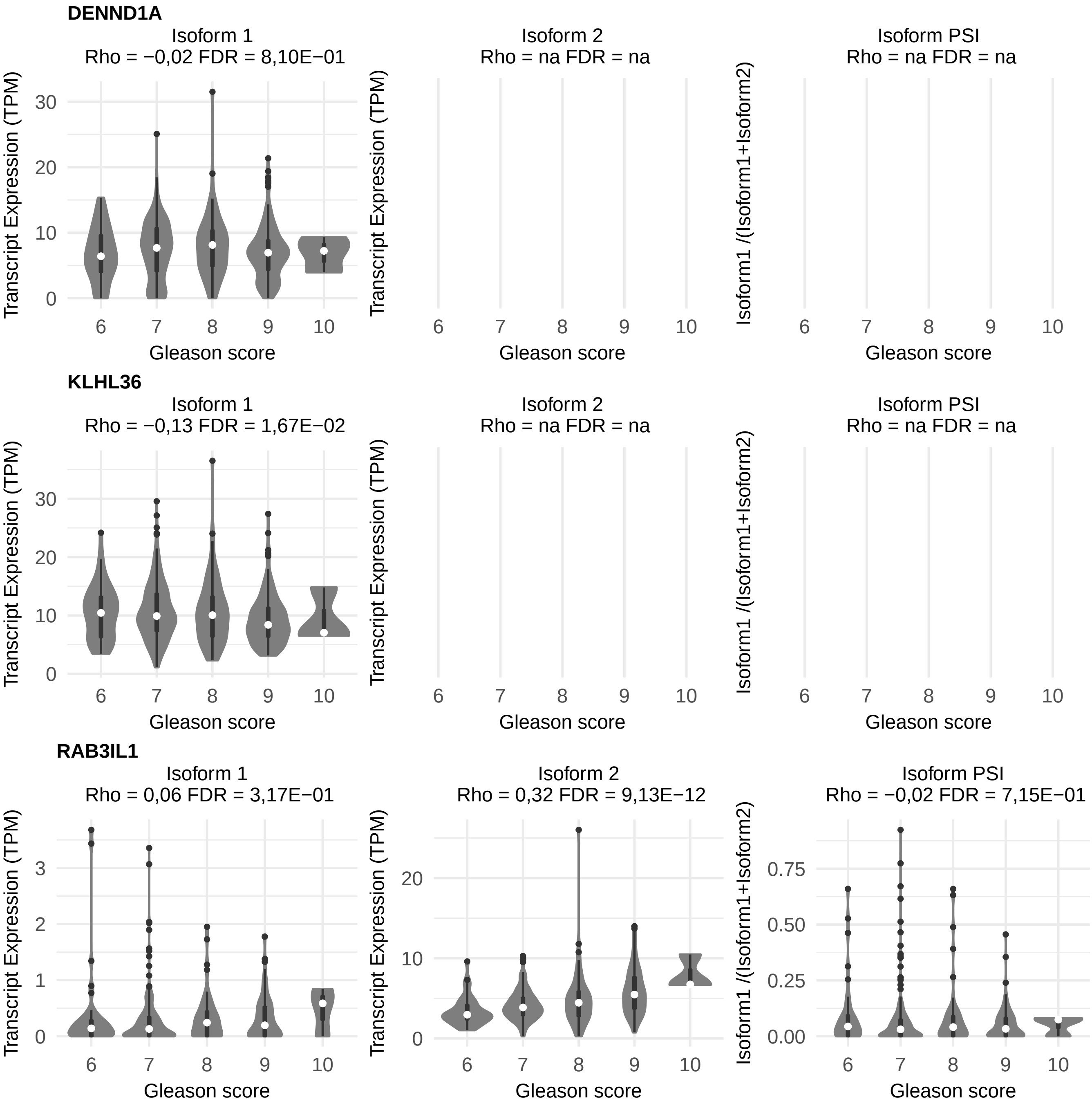

DENND1A
Isoform 1
Rho = −0,02 FDR = 8,10E−01
Isoform 2
Rho = na FDR = na
Isoform PSI
Rho = na FDR = na
KLHL36
Isoform 1
Rho = −0,13 FDR = 1,67E−02
Isoform 2
Rho = na FDR = na
Isoform PSI
Rho = na FDR = na
RAB3IL1
Isoform 1
Rho = 0,06 FDR = 3,17E−01
Isoform 2
Rho = 0,32 FDR = 9,13E−12
Isoform PSI
Rho = −0,02 FDR = 7,15E−01
Transcript Expression (TPM)
Isoform1 /(Isoform1+Isoform2)
Gleason score
6
7
8
9
10

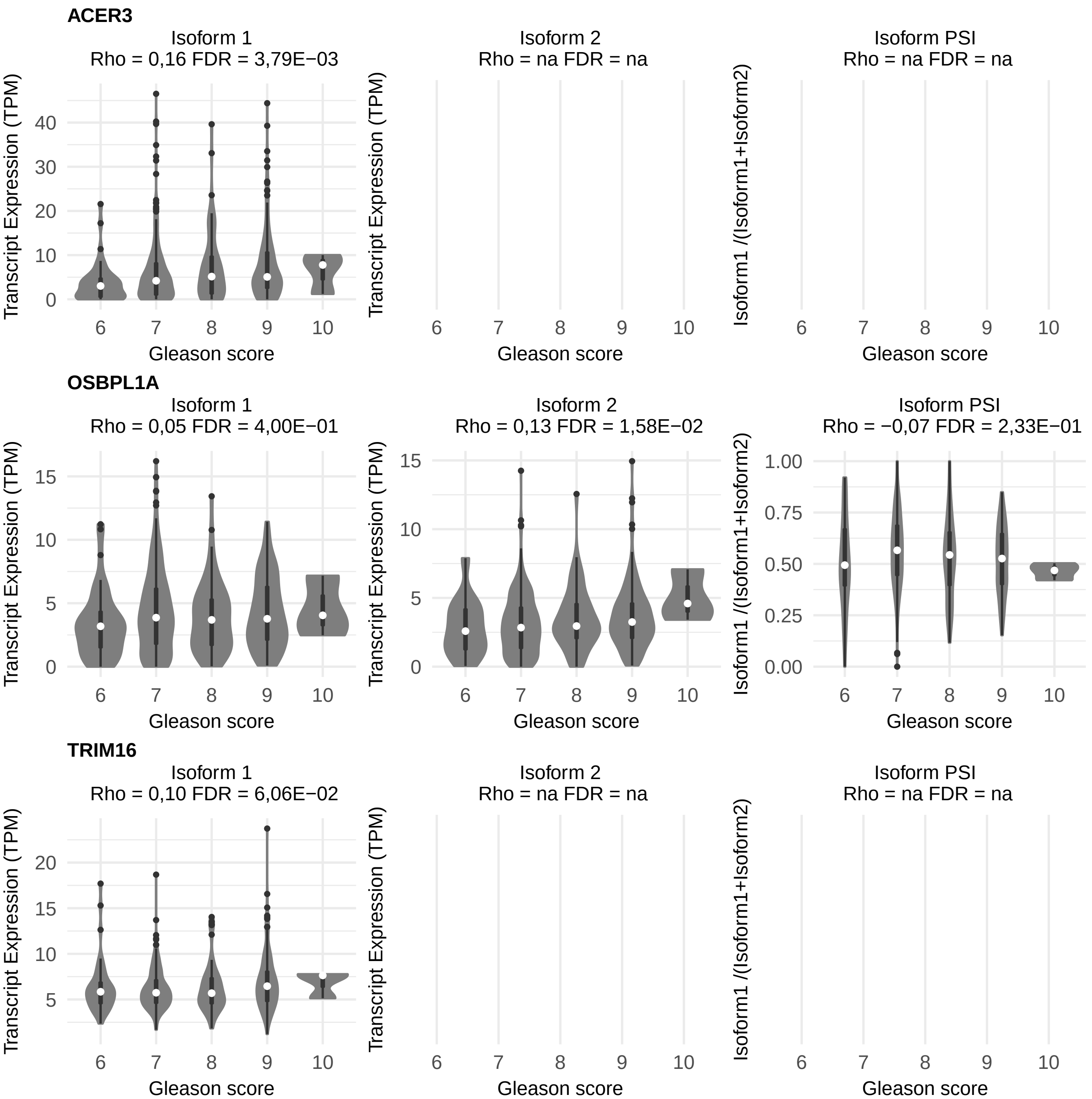

ACER3
Isoform 1
Rho = 0,16 FDR = 3,79E−03
Isoform 2
Rho = na FDR = na
Isoform PSI
Rho = na FDR = na
OSBPL1A
Isoform 1
Rho = 0,05 FDR = 4,00E−01
Isoform 2
Rho = 0,13 FDR = 1,58E−02
Isoform PSI
Rho = −0,07 FDR = 2,33E−01
TRIM16
Isoform 1
Rho = 0,10 FDR = 6,06E−02
Isoform 2
Rho = na FDR = na
Isoform PSI
Rho = na FDR = na
Transcript Expression (TPM)
Isoform1 /(Isoform1+Isoform2)
Gleason score

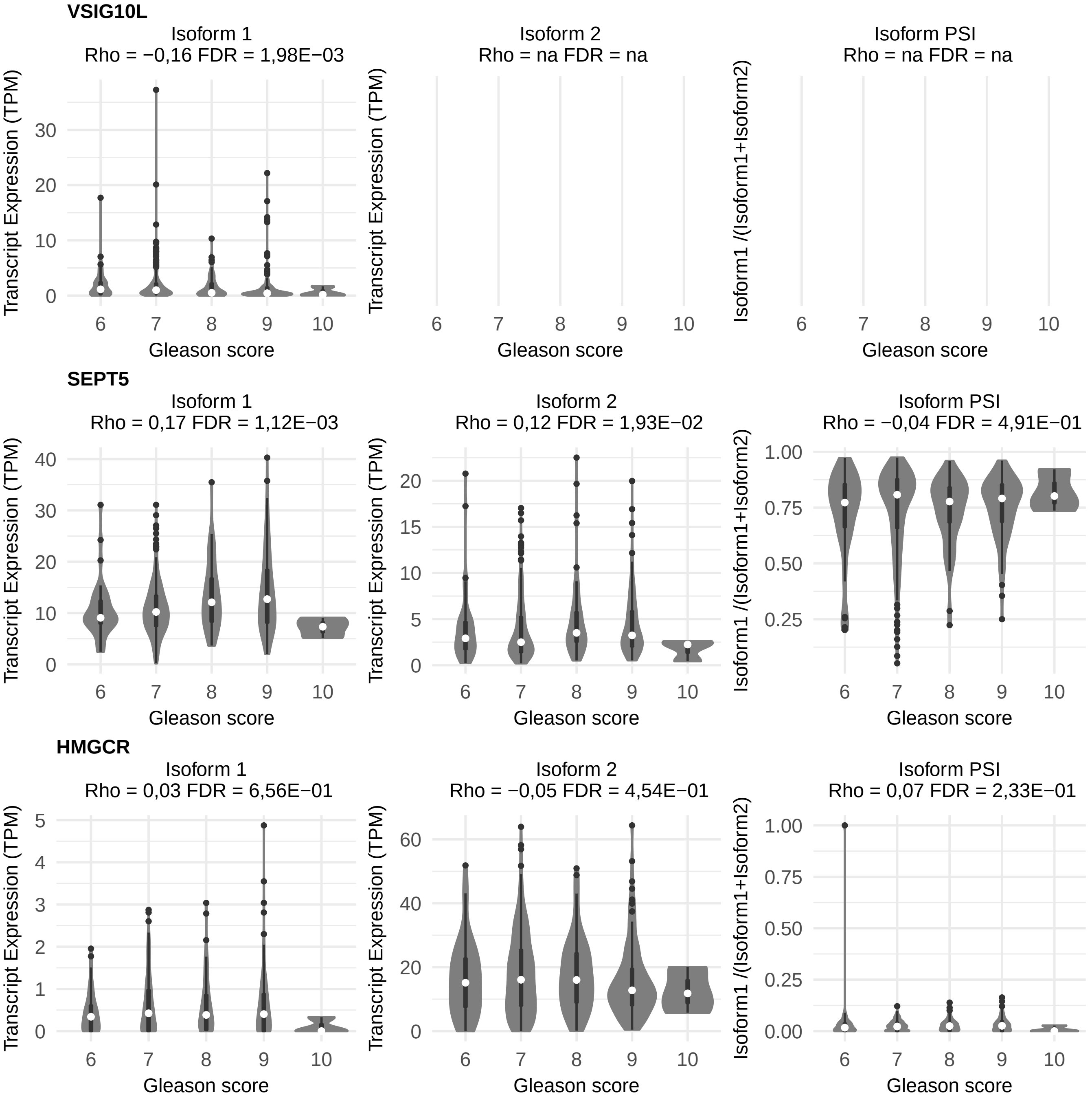
VSIG10L
Isoform 1
Rho = −0,16 FDR = 1,98E−03
Isoform 2
Rho = na FDR = na
Isoform PSI
Rho = na FDR = na
SEPT5
Isoform 1
Rho = 0,17 FDR = 1,12E−03
Isoform 2
Rho = 0,12 FDR = 1,93E−02
Isoform PSI
Rho = −0,04 FDR = 4,91E−01
HMGCR
Isoform 1
Rho = 0,03 FDR = 6,56E−01
Isoform 2
Rho = −0,05 FDR = 4,54E−01
Isoform PSI
Rho = 0,07 FDR = 2,33E−01
Transcript Expression (TPM)
Isoform1 /(Isoform1+Isoform2)
Gleason score
6
7
8
9
10

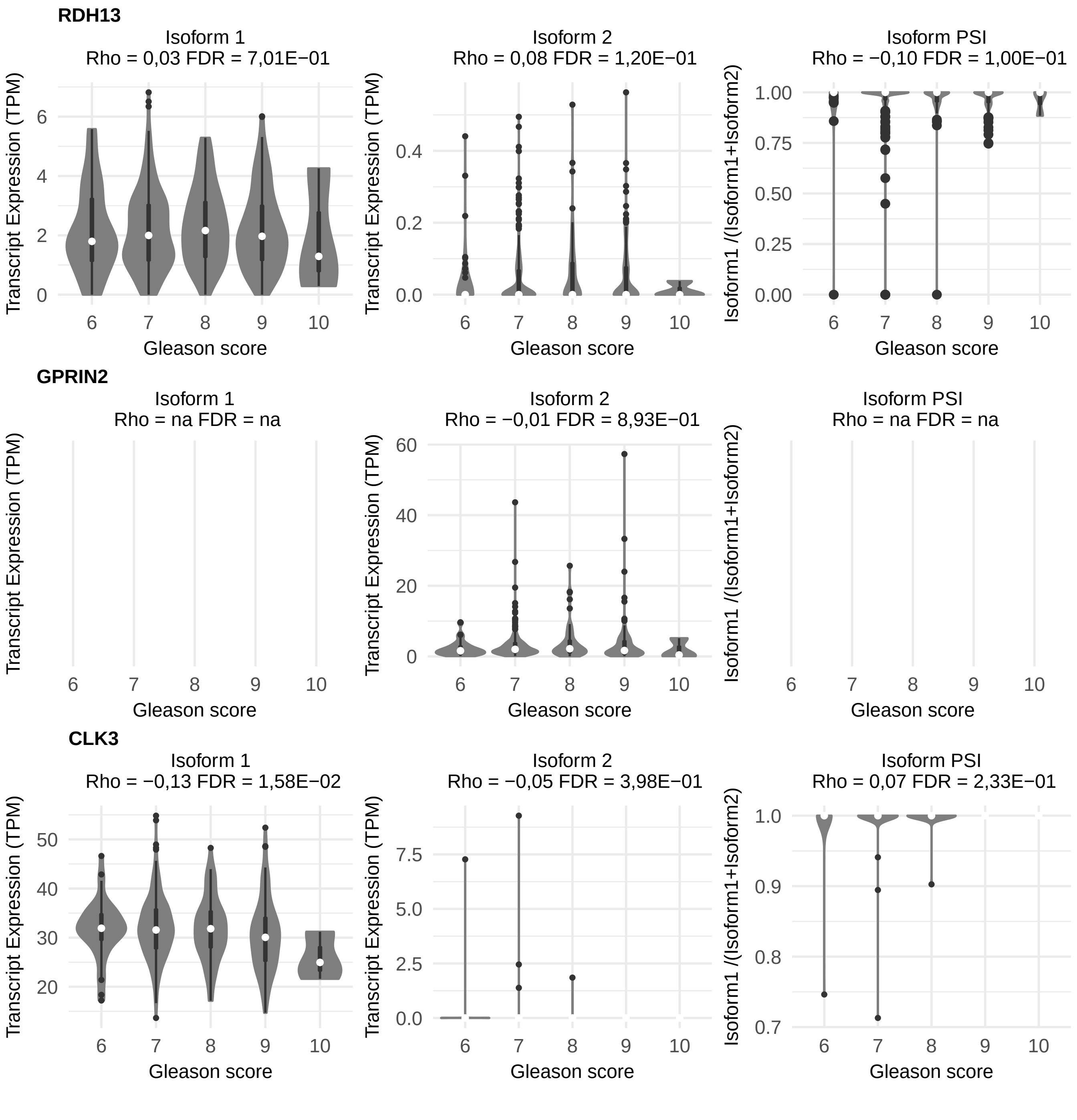
RDH13
Isoform 1
Rho = 0,03 FDR = 7,01E−01
Isoform 2
Rho = 0,08 FDR = 1,20E−01
Isoform PSI
Rho = −0,10 FDR = 1,00E−01
GPRIN2
Isoform 1
Rho = na FDR = na
Isoform 2
Rho = −0,01 FDR = 8,93E−01
Isoform PSI
Rho = na FDR = na
CLK3
Isoform 1
Rho = −0,13 FDR = 1,58E−02
Isoform 2
Rho = −0,05 FDR = 3,98E−01
Isoform PSI
Rho = 0,07 FDR = 2,33E−01
Transcript Expression (TPM)
Isoform1 /(Isoform1+Isoform2)
Gleason score

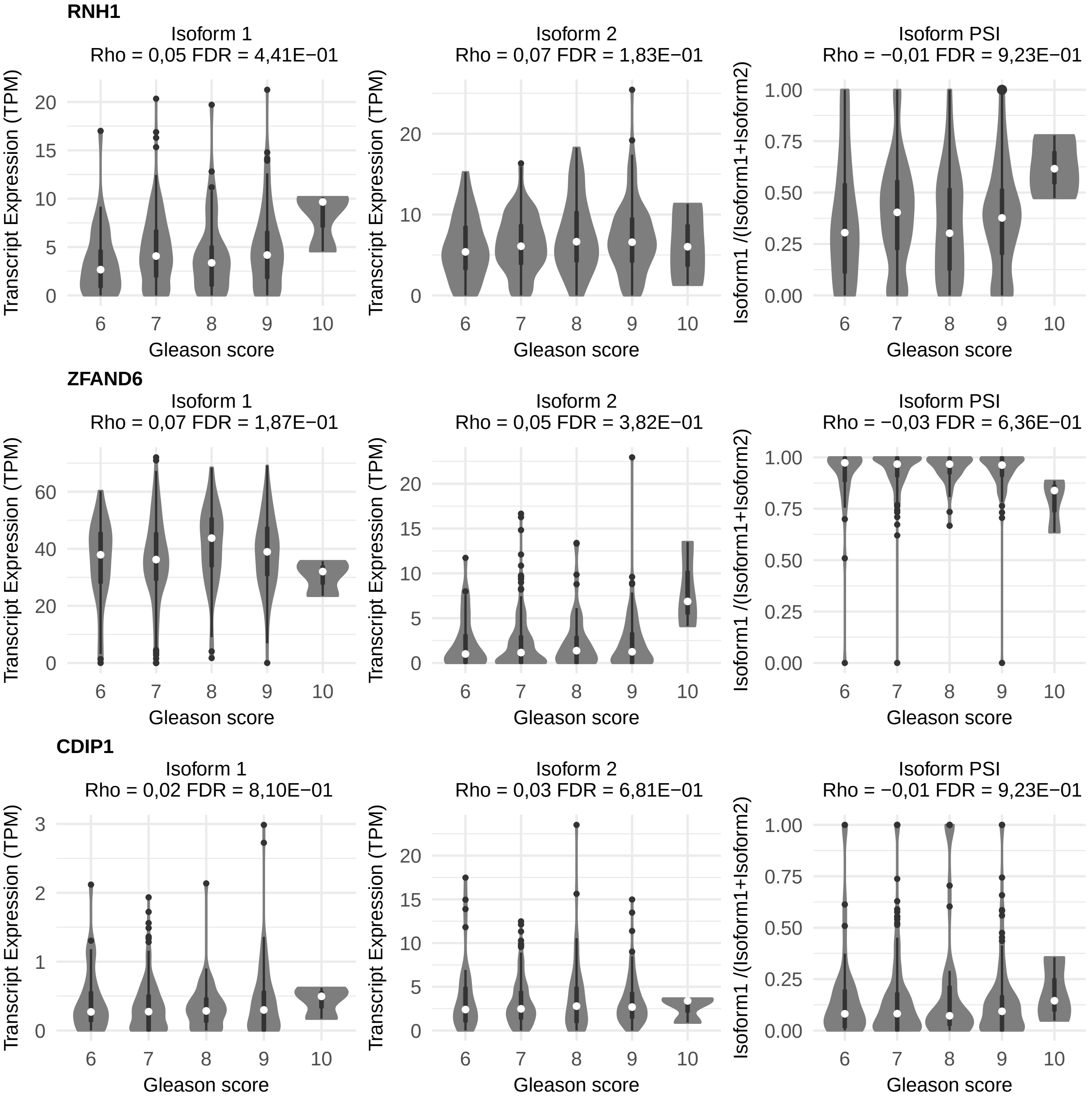

RNH1
Isoform 1
Rho = 0,05 FDR = 4,41E−01
Isoform 2
Rho = 0,07 FDR = 1,83E−01
Isoform PSI
Rho = −0,01 FDR = 9,23E−01
ZFAND6
Isoform 1
Rho = 0,07 FDR = 1,87E−01
Isoform 2
Rho = 0,05 FDR = 3,82E−01
Isoform PSI
Rho = −0,03 FDR = 6,36E−01
CDIP1
Isoform 1
Rho = 0,02 FDR = 8,10E−01
Isoform 2
Rho = 0,03 FDR = 6,81E−01
Isoform PSI
Rho = −0,01 FDR = 9,23E−01
Transcript Expression (TPM)
Isoform1 /(Isoform1+Isoform2)
Gleason score
6
7
8
9
10

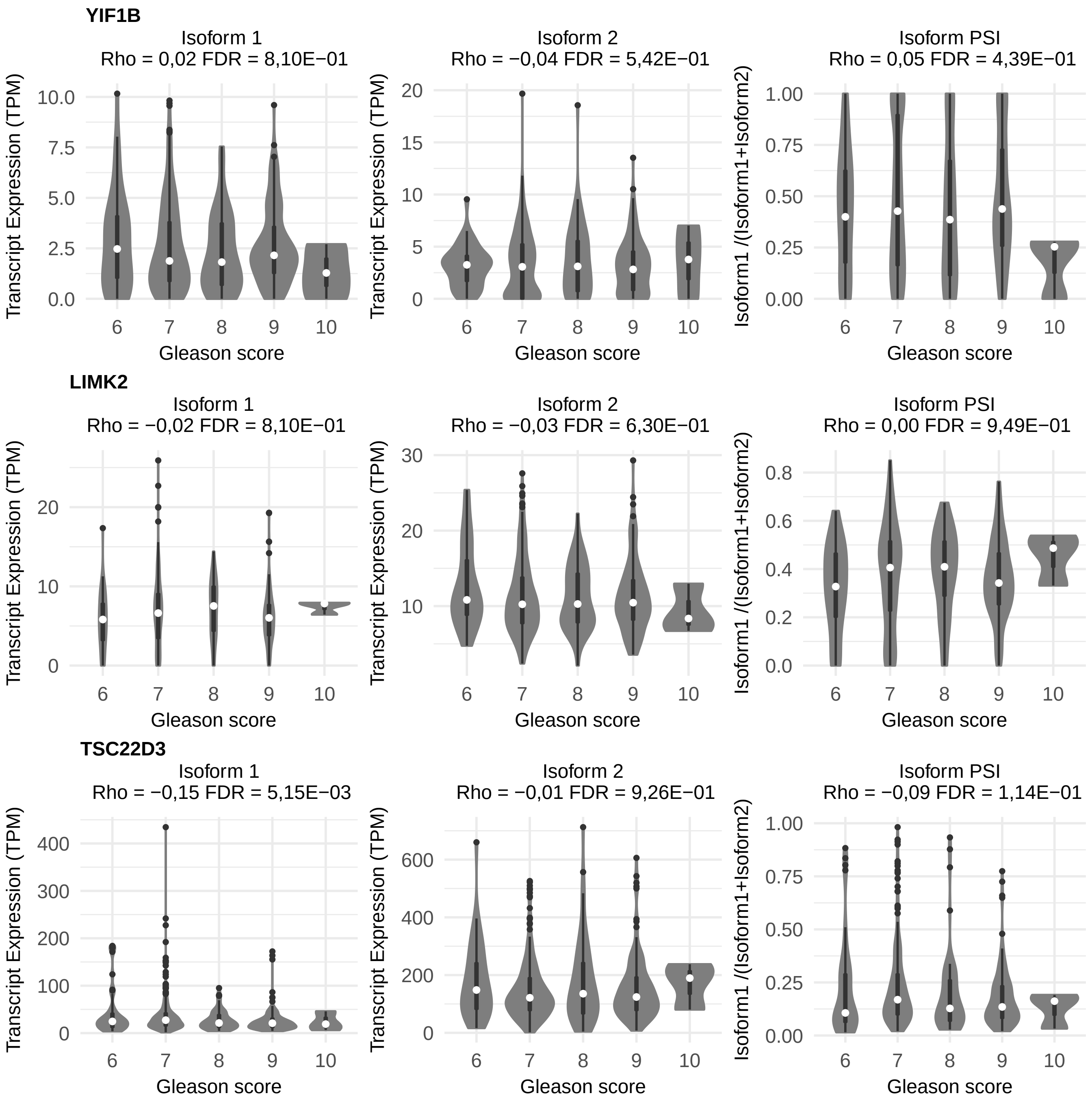
YIF1B
Isoform 1
Rho = 0,02 FDR = 8,10E−01
Isoform 2
Rho = −0,04 FDR = 5,42E−01
Isoform PSI
Rho = 0,05 FDR = 4,39E−01
LIMK2
Isoform 1
Rho = −0,02 FDR = 8,10E−01
Isoform 2
Rho = −0,03 FDR = 6,30E−01
Isoform PSI
Rho = 0,00 FDR = 9,49E−01
TSC22D3
Isoform 1
Rho = −0,15 FDR = 5,15E−03
Isoform 2
Rho = −0,01 FDR = 9,26E−01
Isoform PSI
Rho = −0,09 FDR = 1,14E−01
Transcript Expression (TPM)
Isoform1 /(Isoform1+Isoform2)
Gleason score
6
7
8
9
10

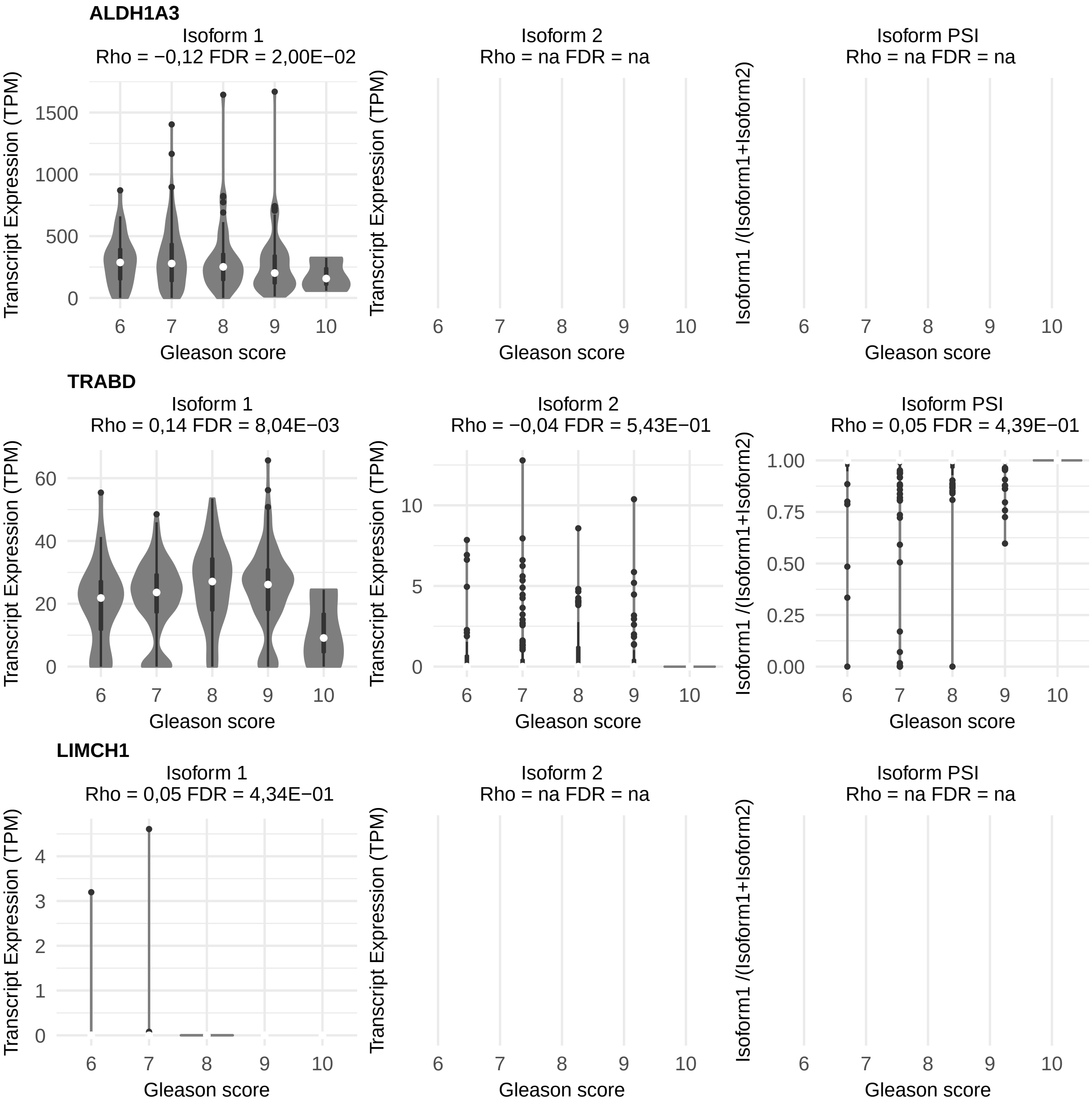
ALDH1A3
Isoform 1
Rho = −0,12 FDR = 2,00E−02
Isoform 2
Rho = na FDR = na
Isoform PSI
Rho = na FDR = na
TRABD
Isoform 1
Rho = 0,14 FDR = 8,04E−03
Isoform 2
Rho = −0,04 FDR = 5,43E−01
Isoform PSI
Rho = 0,05 FDR = 4,39E−01
LIMCH1
Isoform 1
Rho = 0,05 FDR = 4,34E−01
Isoform 2
Rho = na FDR = na
Isoform PSI
Rho = na FDR = na
Transcript Expression (TPM)
Isoform1 /(Isoform1+Isoform2)
Gleason score

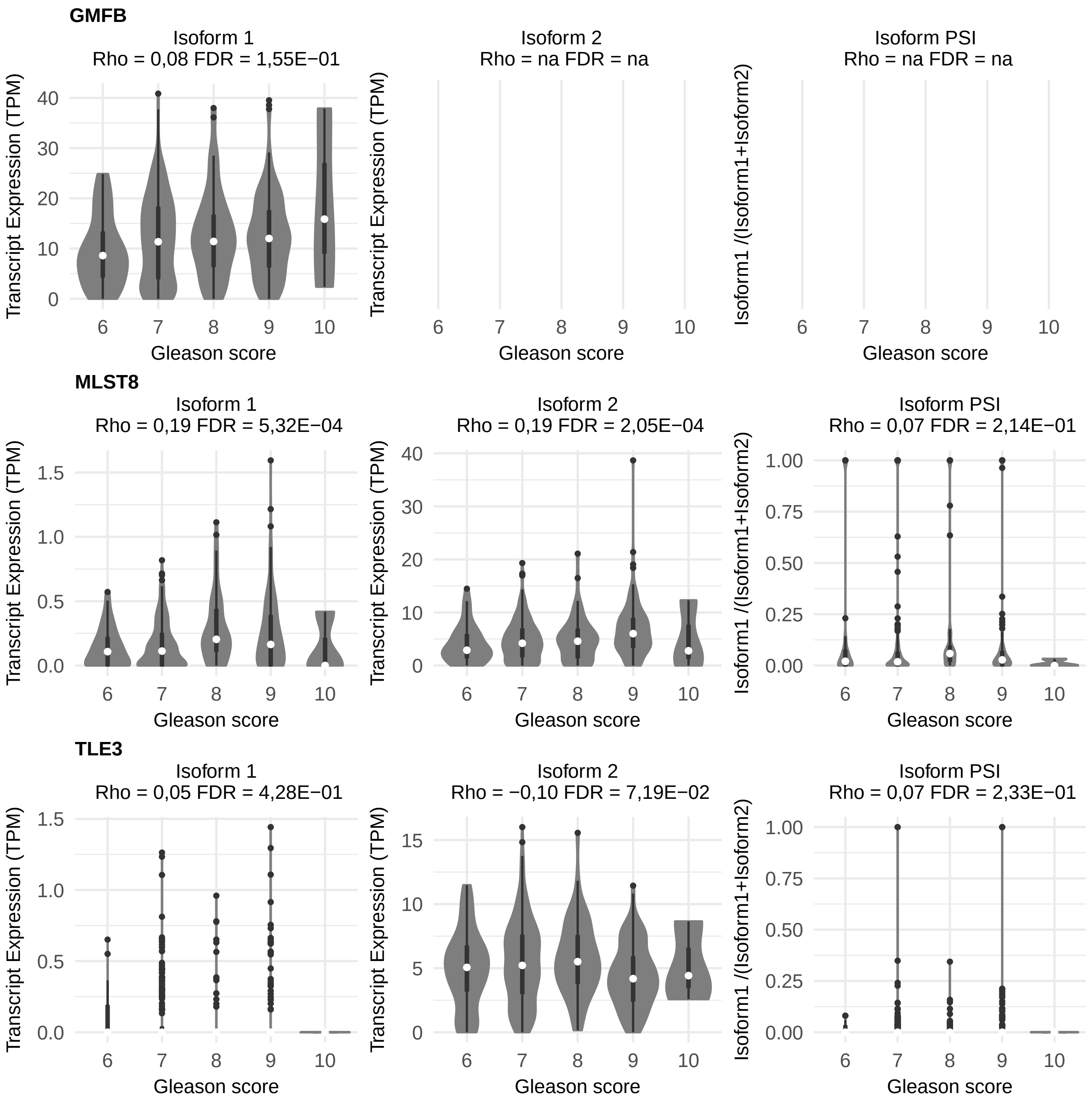
GMFB
Isoform 1
Rho = 0,08 FDR = 1,55E−01
Isoform 2
Rho = na FDR = na
Isoform PSI
Rho = na FDR = na
MLST8
Isoform 1
Rho = 0,19 FDR = 5,32E−04
Isoform 2
Rho = 0,19 FDR = 2,05E−04
Isoform PSI
Rho = 0,07 FDR = 2,14E−01
TLE3
Isoform 1
Rho = 0,05 FDR = 4,28E−01
Isoform 2
Rho = −0,10 FDR = 7,19E−02
Isoform PSI
Rho = 0,07 FDR = 2,33E−01
Transcript Expression (TPM)
Isoform1 /(Isoform1+Isoform2)
Gleason score
6
7
8
9
10

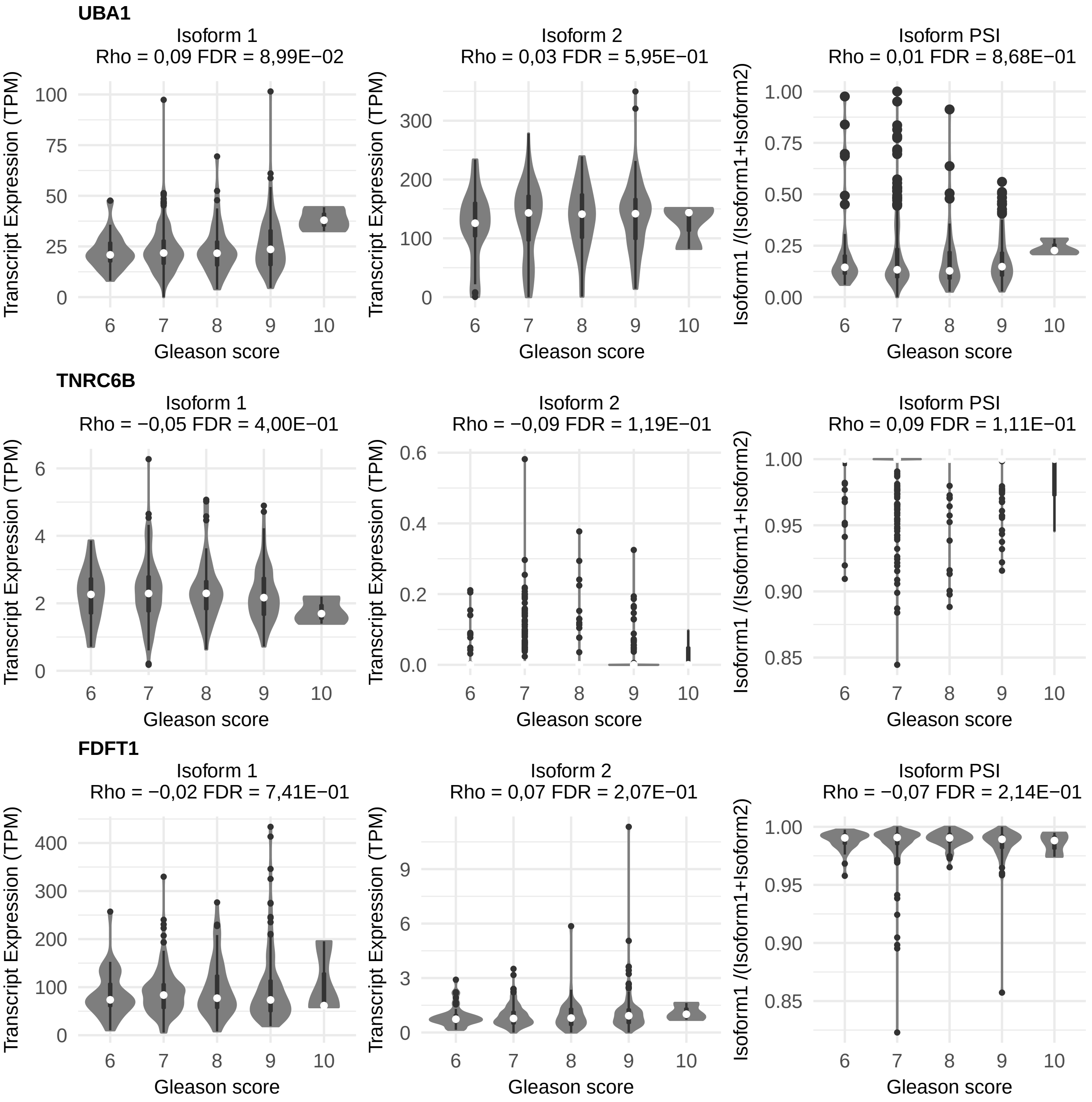
UBA1
Isoform 1
Rho = 0,09 FDR = 8,99E−02
Isoform 2
Rho = 0,03 FDR = 5,95E−01
Isoform PSI
Rho = 0,01 FDR = 8,68E−01
TNRC6B
Isoform 1
Rho = −0,05 FDR = 4,00E−01
Isoform 2
Rho = −0,09 FDR = 1,19E−01
Isoform PSI
Rho = 0,09 FDR = 1,11E−01
FDFT1
Isoform 1
Rho = −0,02 FDR = 7,41E−01
Isoform 2
Rho = 0,07 FDR = 2,07E−01
Isoform PSI
Rho = −0,07 FDR = 2,14E−01
Transcript Expression (TPM)
Isoform1 /(Isoform1+Isoform2)
Gleason score
6
7
8
9
10

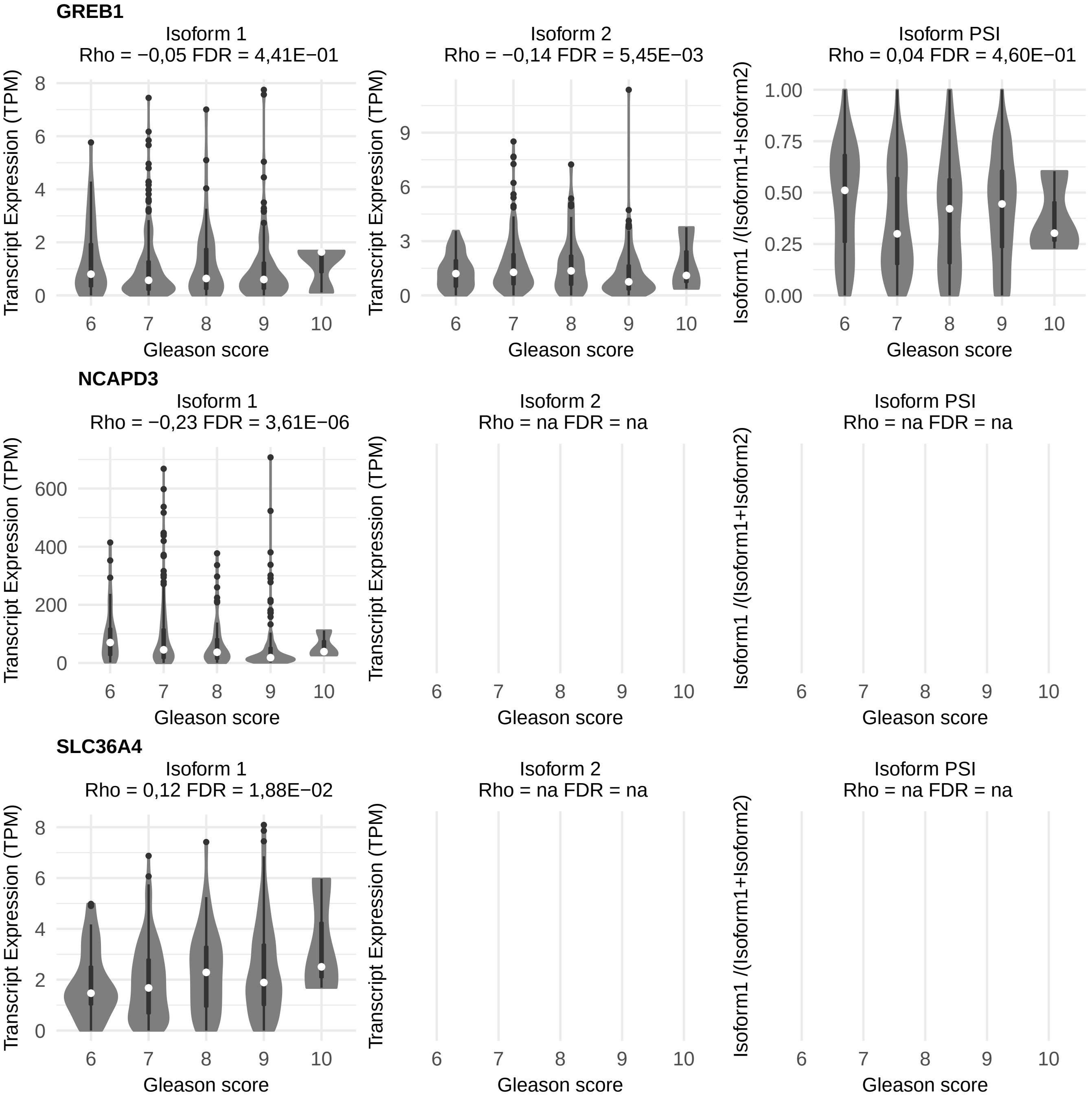
GREB1
Isoform 1
Rho = −0,05 FDR = 4,41E−01
Isoform 2
Rho = −0,14 FDR = 5,45E−03
Isoform PSI
Rho = 0,04 FDR = 4,60E−01
NCAPD3
Isoform 1
Rho = −0,23 FDR = 3,61E−06
Isoform 2
Rho = na FDR = na
Isoform PSI
Rho = na FDR = na
SLC36A4
Isoform 1
Rho = 0,12 FDR = 1,88E−02
Isoform 2
Rho = na FDR = na
Isoform PSI
Rho = na FDR = na
Transcript Expression (TPM)
Isoform1 /(Isoform1+Isoform2)
Gleason score
6
7
8
9
10

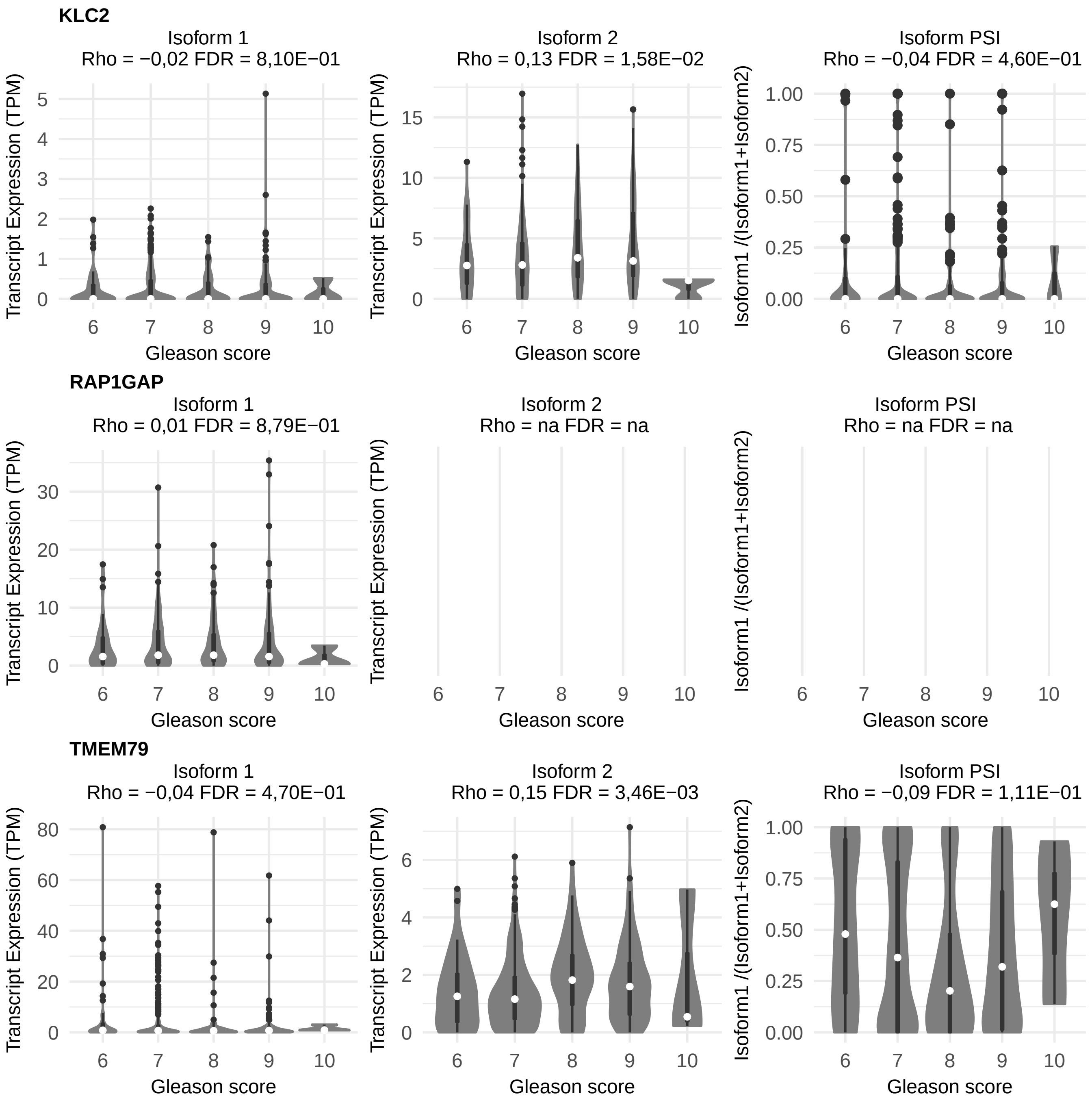
KLC2
Isoform 1
Rho = −0,02 FDR = 8,10E−01
Isoform 2
Rho = 0,13 FDR = 1,58E−02
Isoform PSI
Rho = −0,04 FDR = 4,60E−01
RAP1GAP
Isoform 1
Rho = 0,01 FDR = 8,79E−01
Isoform 2
Rho = na FDR = na
Isoform PSI
Rho = na FDR = na
TMEM79
Isoform 1
Rho = −0,04 FDR = 4,70E−01
Isoform 2
Rho = 0,15 FDR = 3,46E−03
Isoform PSI
Rho = −0,09 FDR = 1,11E−01
Transcript Expression (TPM)
Isoform1 /(Isoform1+Isoform2)
Gleason score
6
7
8
9
10

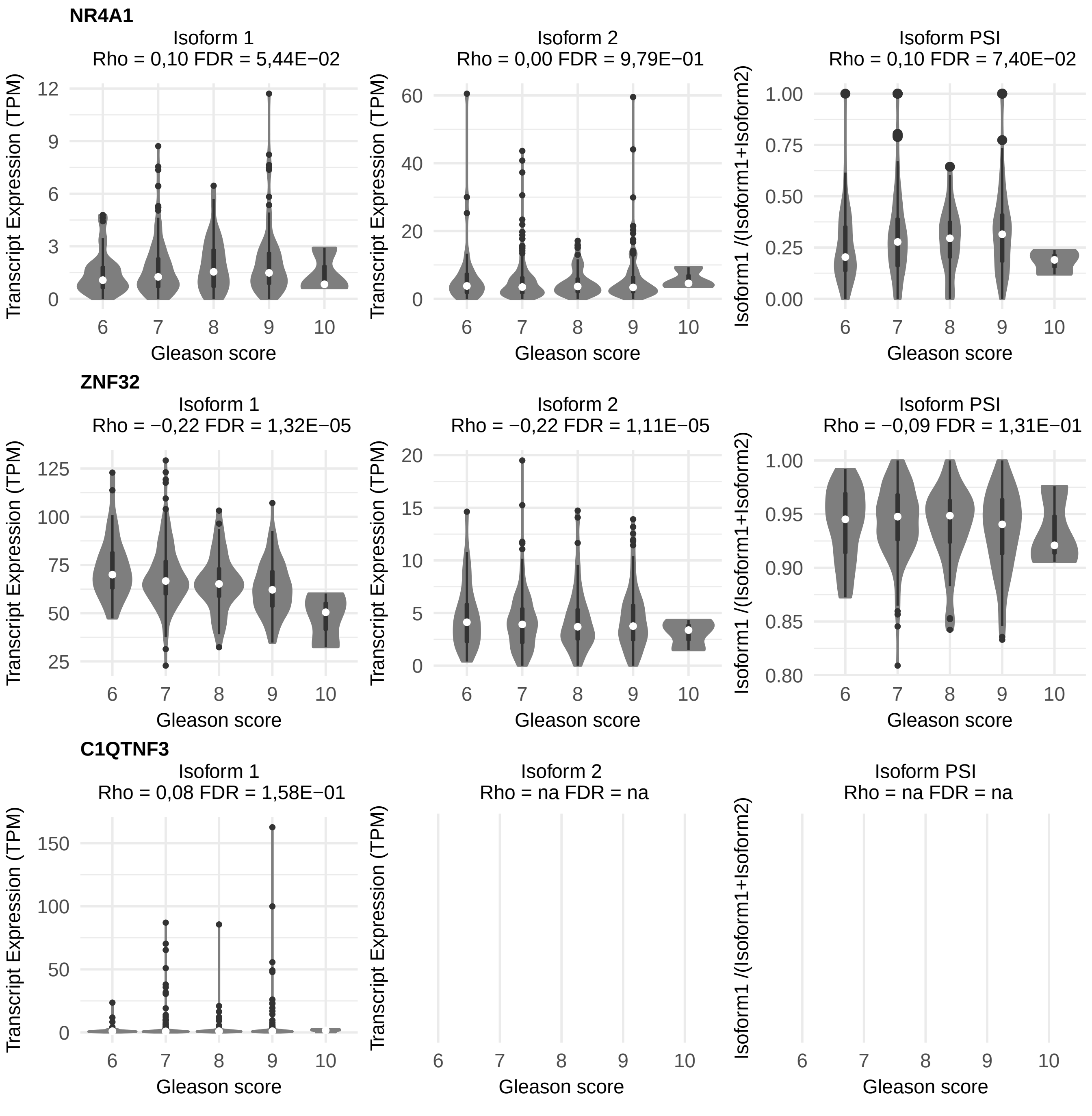
NR4A1
Isoform 1
Rho = 0,10 FDR = 5,44E−02
Isoform 2
Rho = 0,00 FDR = 9,79E−01
Isoform PSI
Rho = 0,10 FDR = 7,40E−02
ZNF32
Isoform 1
Rho = −0,22 FDR = 1,32E−05
Isoform 2
Rho = −0,22 FDR = 1,11E−05
Isoform PSI
Rho = −0,09 FDR = 1,31E−01
C1QTNF3
Isoform 1
Rho = 0,08 FDR = 1,58E−01
Isoform 2
Rho = na FDR = na
Isoform PSI
Rho = na FDR = na
Transcript Expression (TPM)
Isoform1 /(Isoform1+Isoform2)
Gleason score
6
7
8
9
10

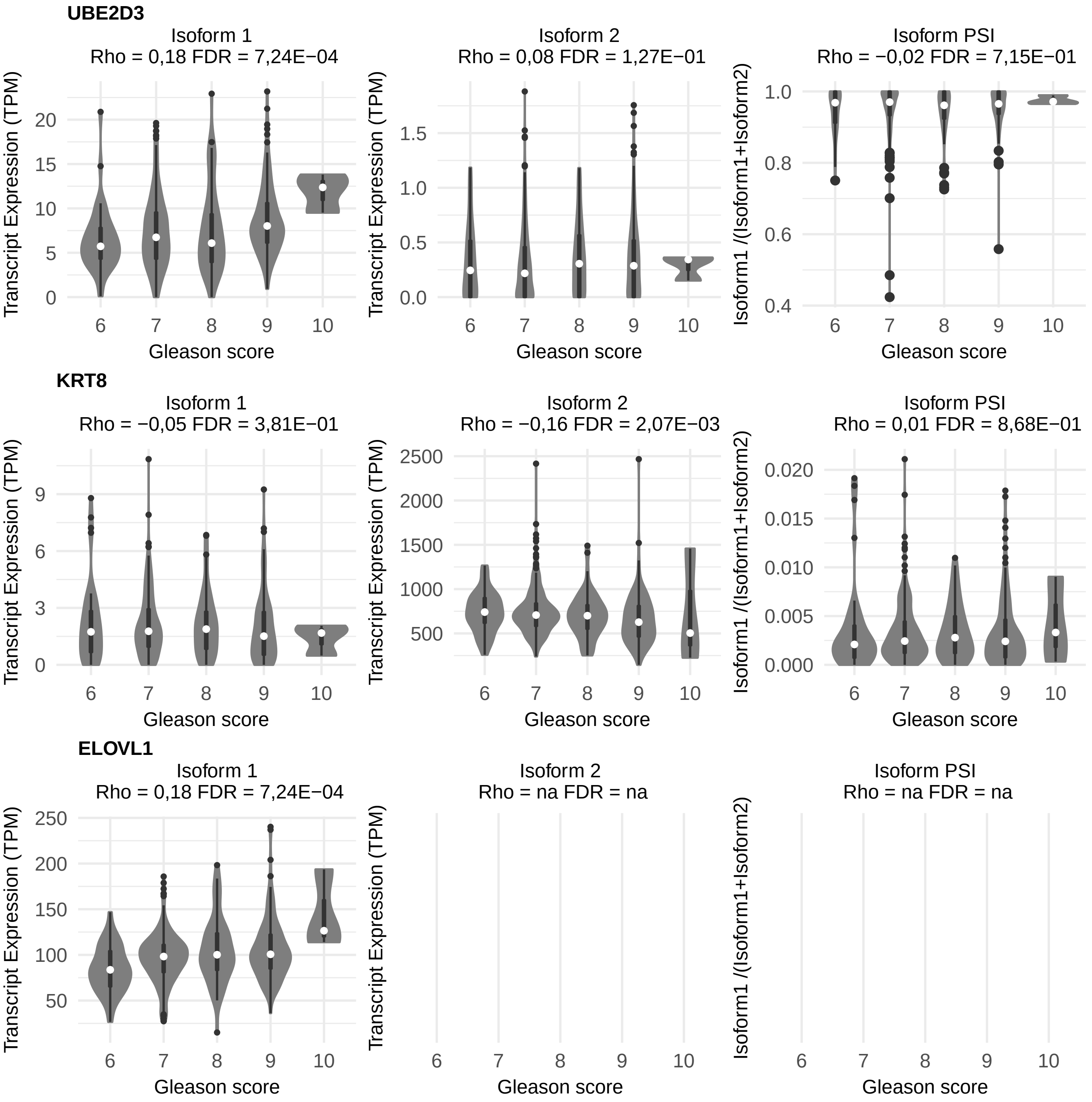
UBE2D3
Isoform 1
Rho = 0,18 FDR = 7,24E−04
Isoform 2
Rho = 0,08 FDR = 1,27E−01
Isoform PSI
Rho = −0,02 FDR = 7,15E−01
KRT8
Isoform 1
Rho = −0,05 FDR = 3,81E−01
Isoform 2
Rho = −0,16 FDR = 2,07E−03
Isoform PSI
Rho = 0,01 FDR = 8,68E−01
ELOVL1
Isoform 1
Rho = 0,18 FDR = 7,24E−04
Isoform 2
Rho = na FDR = na
Isoform PSI
Rho = na FDR = na
Transcript Expression (TPM)
Isoform1 /(Isoform1+Isoform2)
Gleason score
6
7
8
9
10

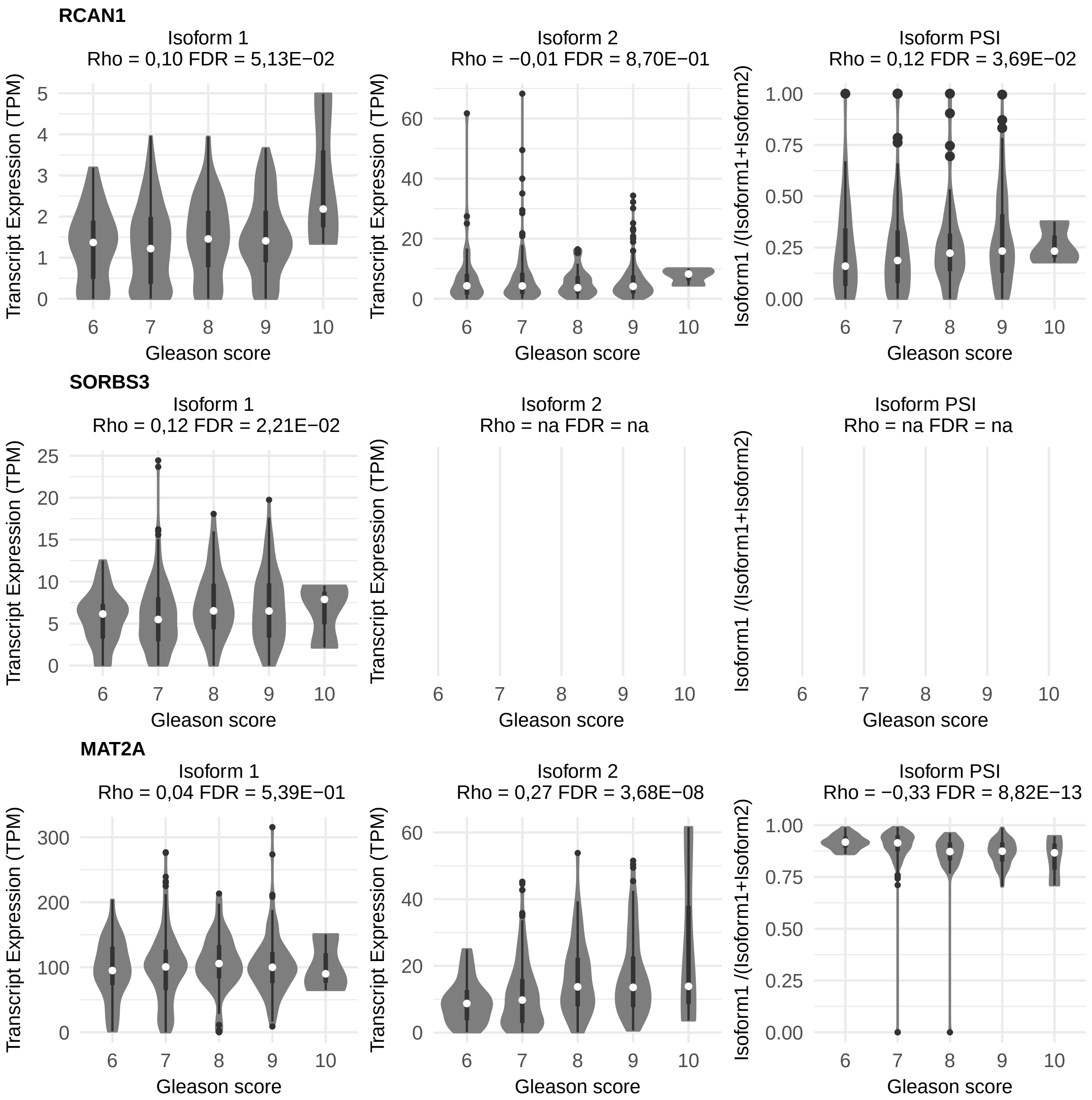
RCAN1
Isoform 1
Rho = 0,10 FDR = 5,13E−02
Isoform 2
Rho = −0,01 FDR = 8,70E−01
Isoform PSI
Rho = 0,12 FDR = 3,69E−02
SORBS3
Isoform 1
Rho = 0,12 FDR = 2,21E−02
Isoform 2
Rho = na FDR = na
Isoform PSI
Rho = na FDR = na
MAT2A
Isoform 1
Rho = 0,04 FDR = 5,39E−01
Isoform 2
Rho = 0,27 FDR = 3,68E−08
Isoform PSI
Rho = −0,33 FDR = 8,82E−13
Transcript Expression (TPM)
Isoform1 /(Isoform1+Isoform2)
Gleason score

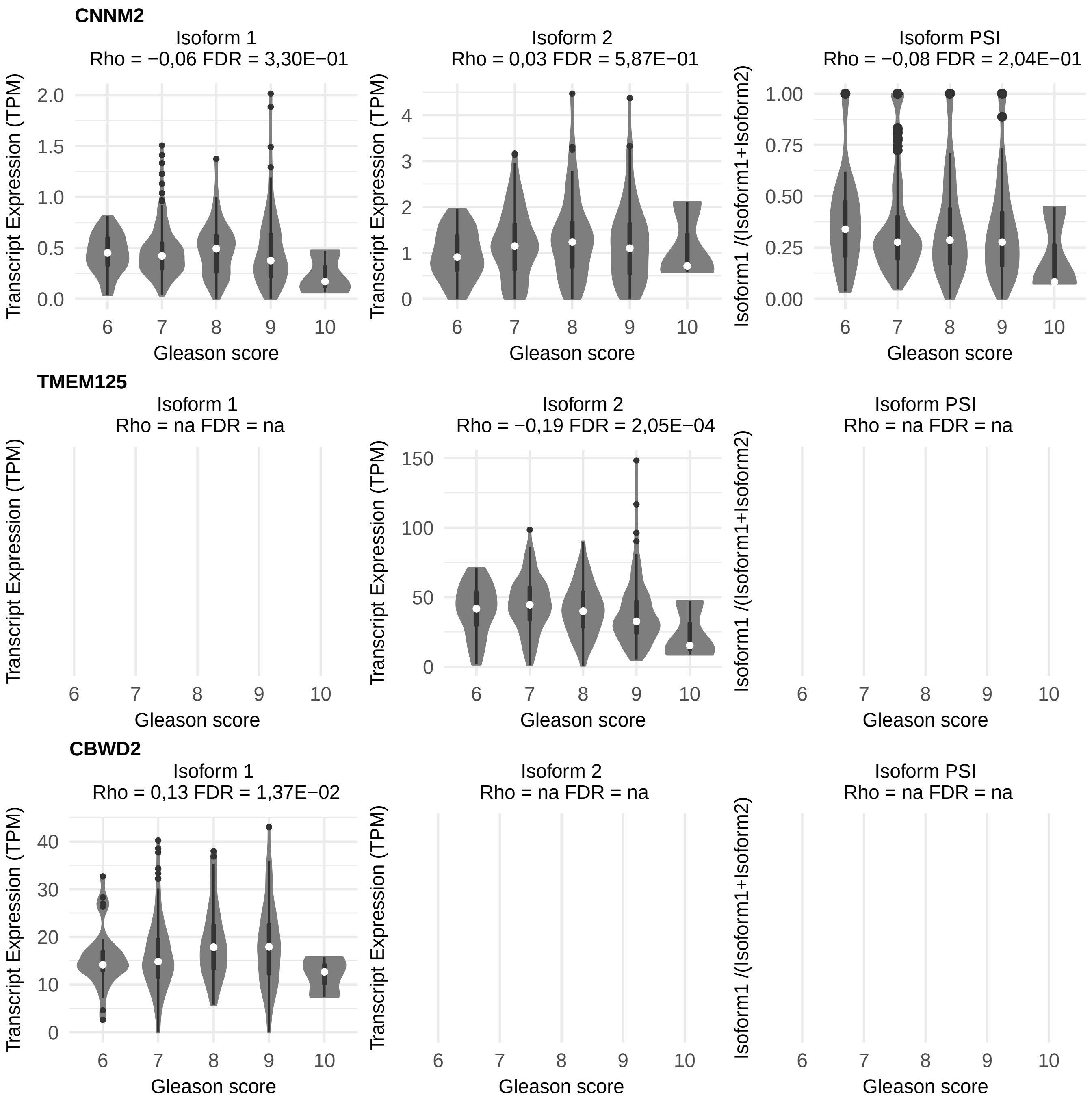
CNNM2
Isoform 1
Rho = −0,06 FDR = 3,30E−01
Isoform 2
Rho = 0,03 FDR = 5,87E−01
Isoform PSI
Rho = −0,08 FDR = 2,04E−01
TMEM125
Isoform 1
Rho = na FDR = na
Isoform 2
Rho = −0,19 FDR = 2,05E−04
Isoform PSI
Rho = na FDR = na
CBWD2
Isoform 1
Rho = 0,13 FDR = 1,37E−02
Isoform 2
Rho = na FDR = na
Isoform PSI
Rho = na FDR = na
Transcript Expression (TPM)
Isoform1 /(Isoform1+Isoform2)
Gleason score

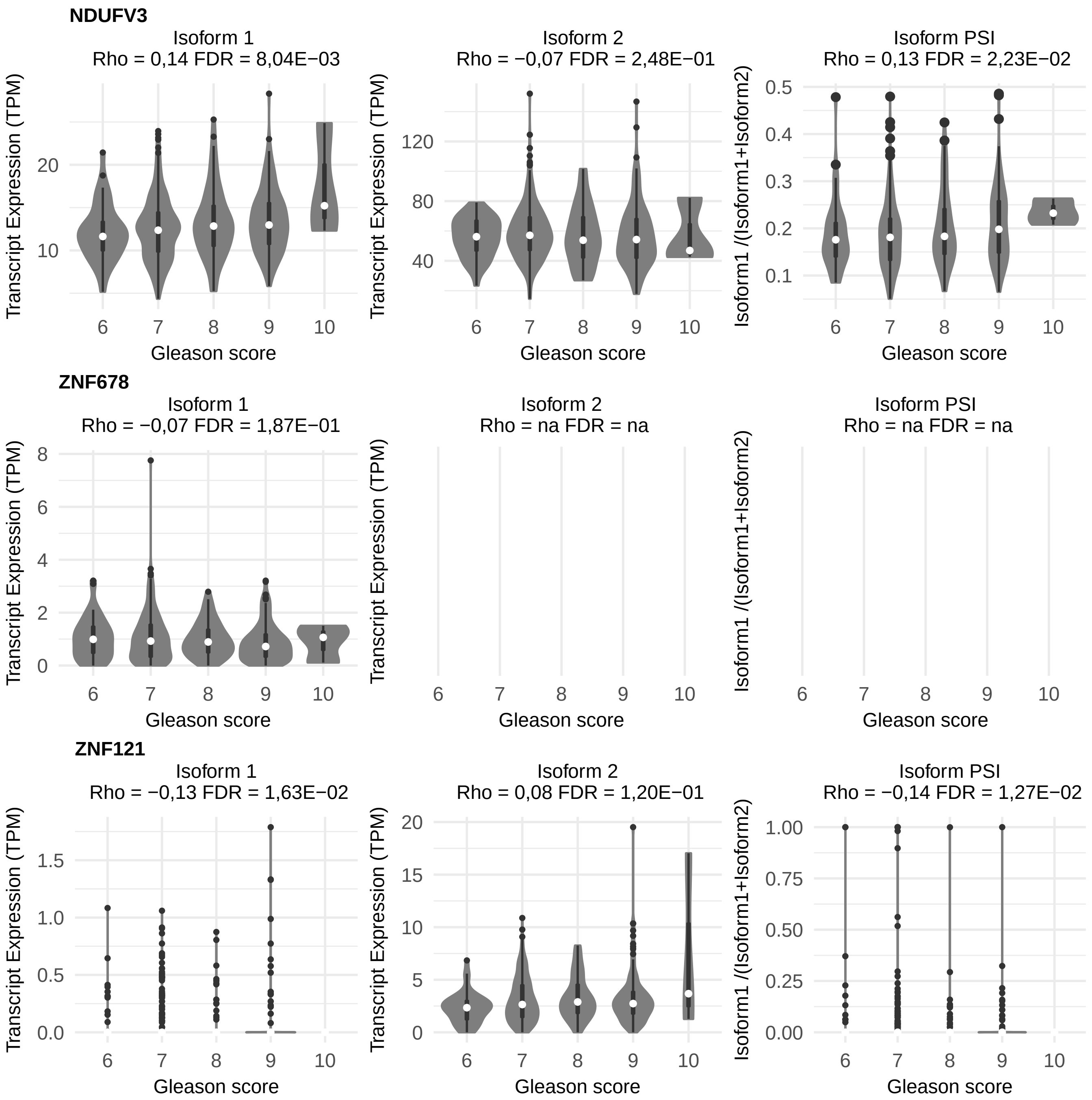
NDUFV3
Isoform 1
Rho = 0,14 FDR = 8,04E−03
Isoform 2
Rho = −0,07 FDR = 2,48E−01
Isoform PSI
Rho = 0,13 FDR = 2,23E−02
ZNF678
Isoform 1
Rho = −0,07 FDR = 1,87E−01
Isoform 2
Rho = na FDR = na
Isoform PSI
Rho = na FDR = na
ZNF121
Isoform 1
Rho = −0,13 FDR = 1,63E−02
Isoform 2
Rho = 0,08 FDR = 1,20E−01
Isoform PSI
Rho = −0,14 FDR = 1,27E−02
Transcript Expression (TPM)
Isoform1 /(Isoform1+Isoform2)
Gleason score

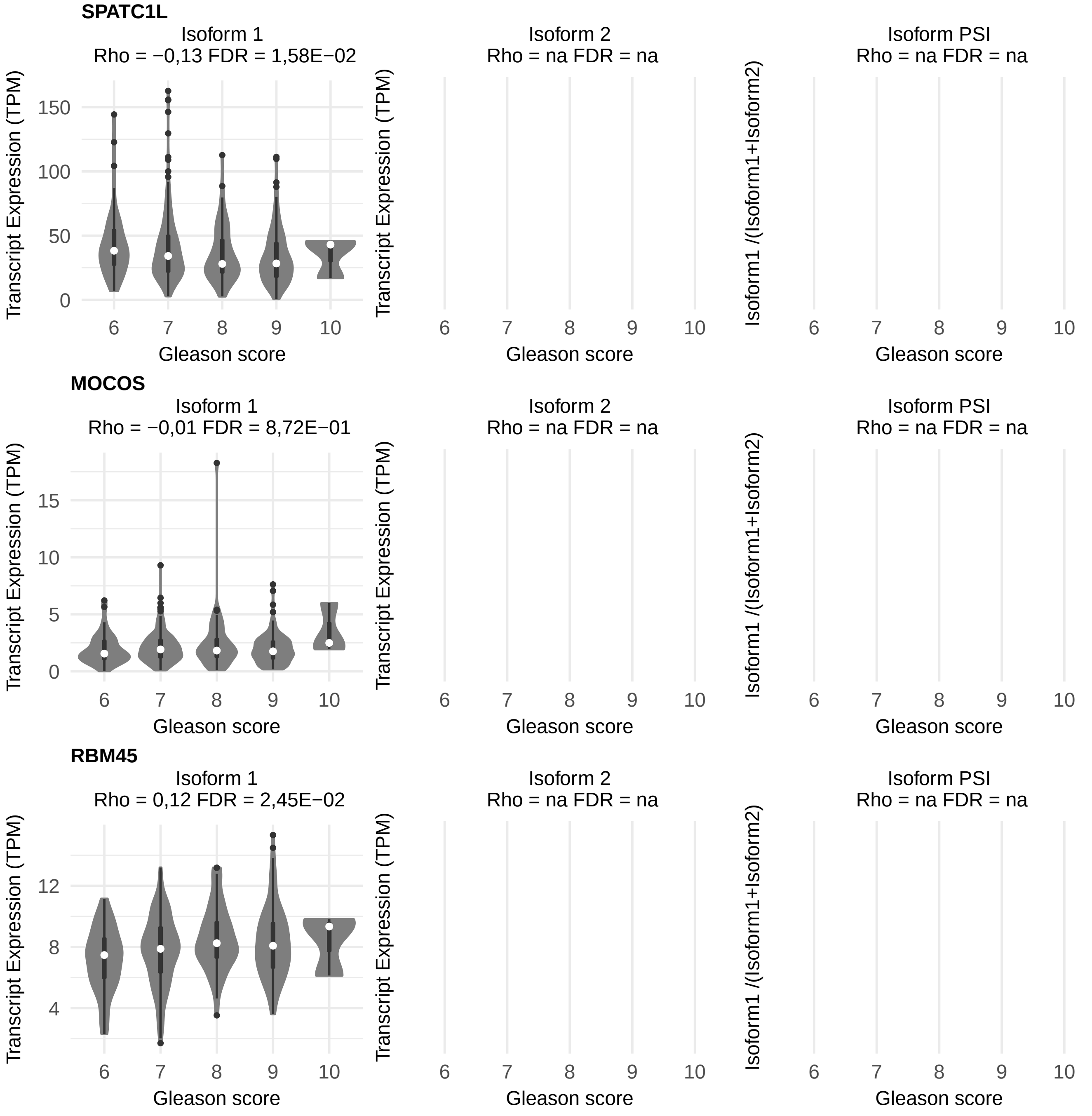

SPATC1L
Isoform 1
Rho = −0,13 FDR = 1,58E−02
Isoform 2
Rho = na FDR = na
Isoform PSI
Rho = na FDR = na
MOCOS
Isoform 1
Rho = −0,01 FDR = 8,72E−01
Isoform 2
Rho = na FDR = na
Isoform PSI
Rho = na FDR = na
RBM45
Isoform 1
Rho = 0,12 FDR = 2,45E−02
Isoform 2
Rho = na FDR = na
Isoform PSI
Rho = na FDR = na
Transcript Expression (TPM)
Isoform1 /(Isoform1+Isoform2)
Gleason score
6
7
8
9
10

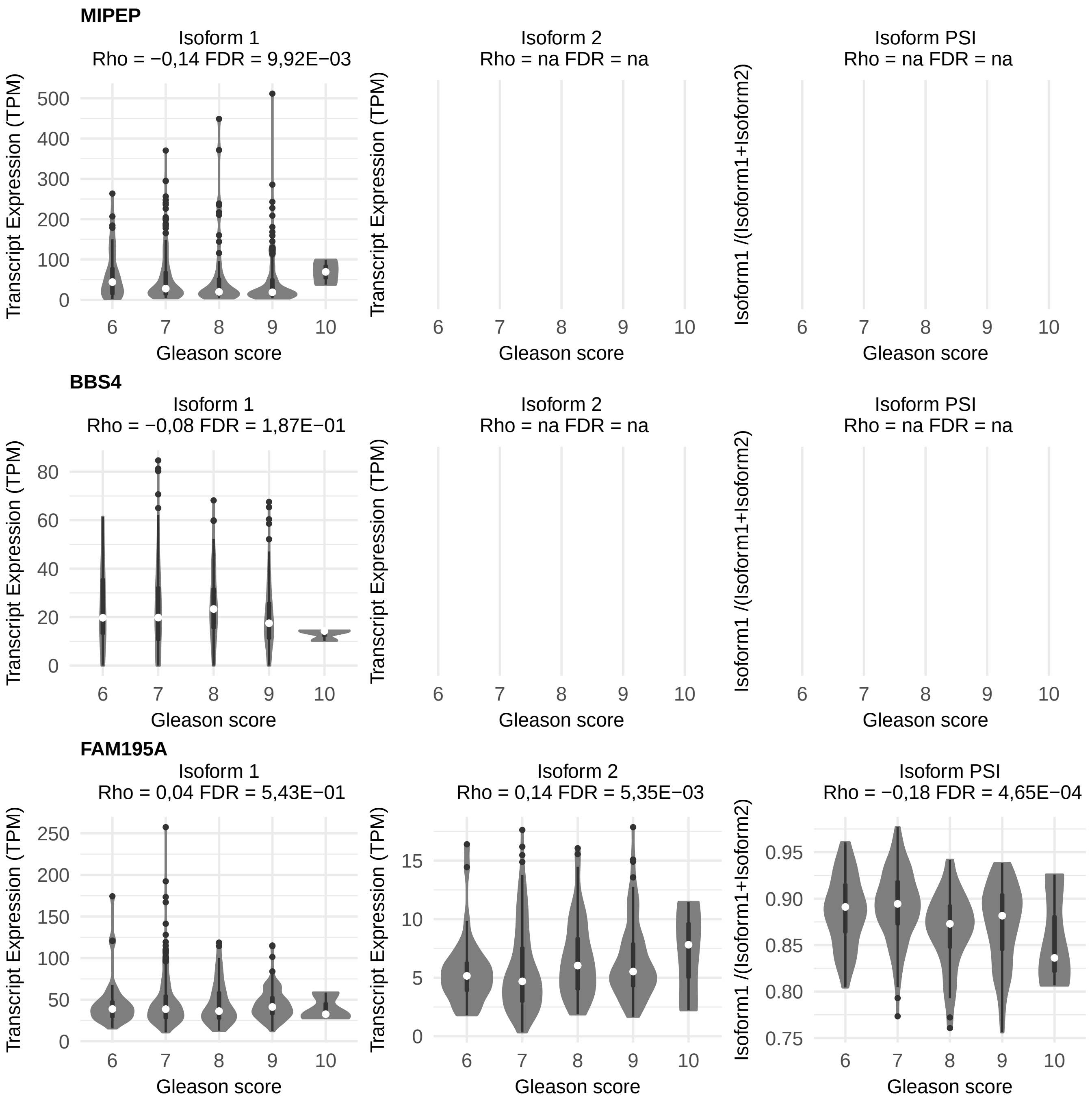
MIPEP
Isoform 1
Rho = −0,14 FDR = 9,92E−03
Isoform 2
Rho = na FDR = na
Isoform PSI
Rho = na FDR = na
BBS4
Isoform 1
Rho = −0,08 FDR = 1,87E−01
Isoform 2
Rho = na FDR = na
Isoform PSI
Rho = na FDR = na
FAM195A
Isoform 1
Rho = 0,04 FDR = 5,43E−01
Isoform 2
Rho = 0,14 FDR = 5,35E−03
Isoform PSI
Rho = −0,18 FDR = 4,65E−04
Transcript Expression (TPM)
Isoform1 /(Isoform1+Isoform2)
Gleason score
6
7
8
9
10

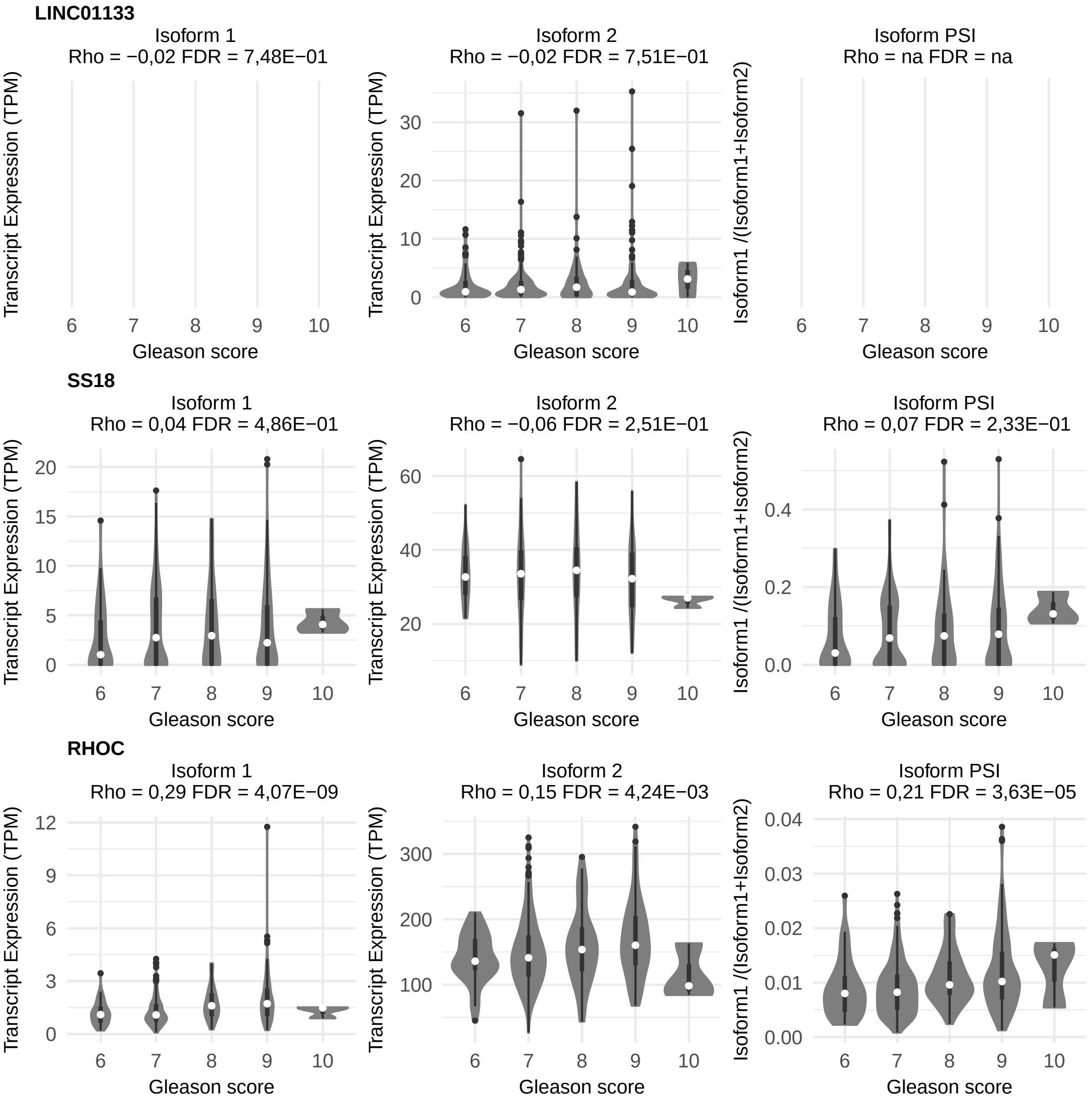
LINC01133
Isoform 1
Rho = −0,02 FDR = 7,48E−01
Isoform 2
Rho = −0,02 FDR = 7,51E−01
Isoform PSI
Rho = na FDR = na
SS18
Isoform 1
Rho = 0,04 FDR = 4,86E−01
Isoform 2
Rho = −0,06 FDR = 2,51E−01
Isoform PSI
Rho = 0,07 FDR = 2,33E−01
RHOC
Isoform 1
Rho = 0,29 FDR = 4,07E−09
Isoform 2
Rho = 0,15 FDR = 4,24E−03
Isoform PSI
Rho = 0,21 FDR = 3,63E−05
Transcript Expression (TPM)
Isoform1 /(Isoform1+Isoform2)
Gleason score
6
7
8
9
10

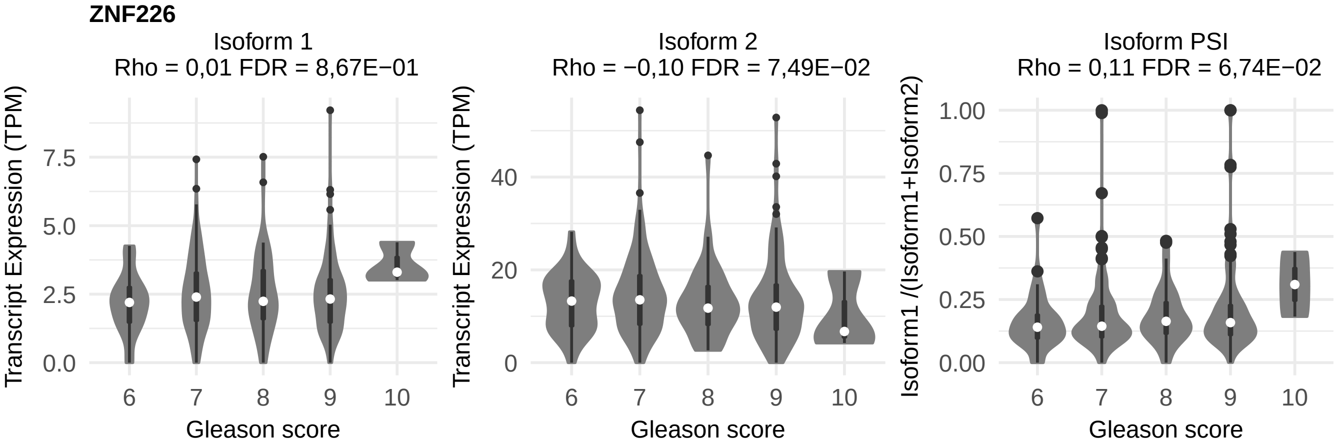
ZNF226
Isoform 1
Rho = 0,01 FDR = 8,67E−01
Transcript Expression (TPM)
0.0
2.5
5.0
7.5
6
7
8
9
10
Gleason score
Isoform 2
Rho = −0,10 FDR = 7,49E−02
Transcript Expression (TPM)
0
20
40
6
7
8
9
10
Gleason score
Isoform PSI
Rho = 0,11 FDR = 6,74E−02
Isoform1 /(Isoform1+Isoform2)
0.00
0.25
0.50
0.75
1.00
6
7
8
9
10
Gleason score
