# Supplementary material for: Androgen-dependent alternative mRNA isoform expression in prostate cancer cells
Source: F1000Res. 2018 Aug 3;7:1189. [Version 1] doi: 10.12688/f1000research.15604.1 (PMC6143958; doi:10.12688/f1000research.15604.1)
Supplement: Supplementary file 8 [file f1000research-7-17022-s0007.tgz › 2fc3b6ff-1195-413c-a50b-1249bce4455f.pdf]

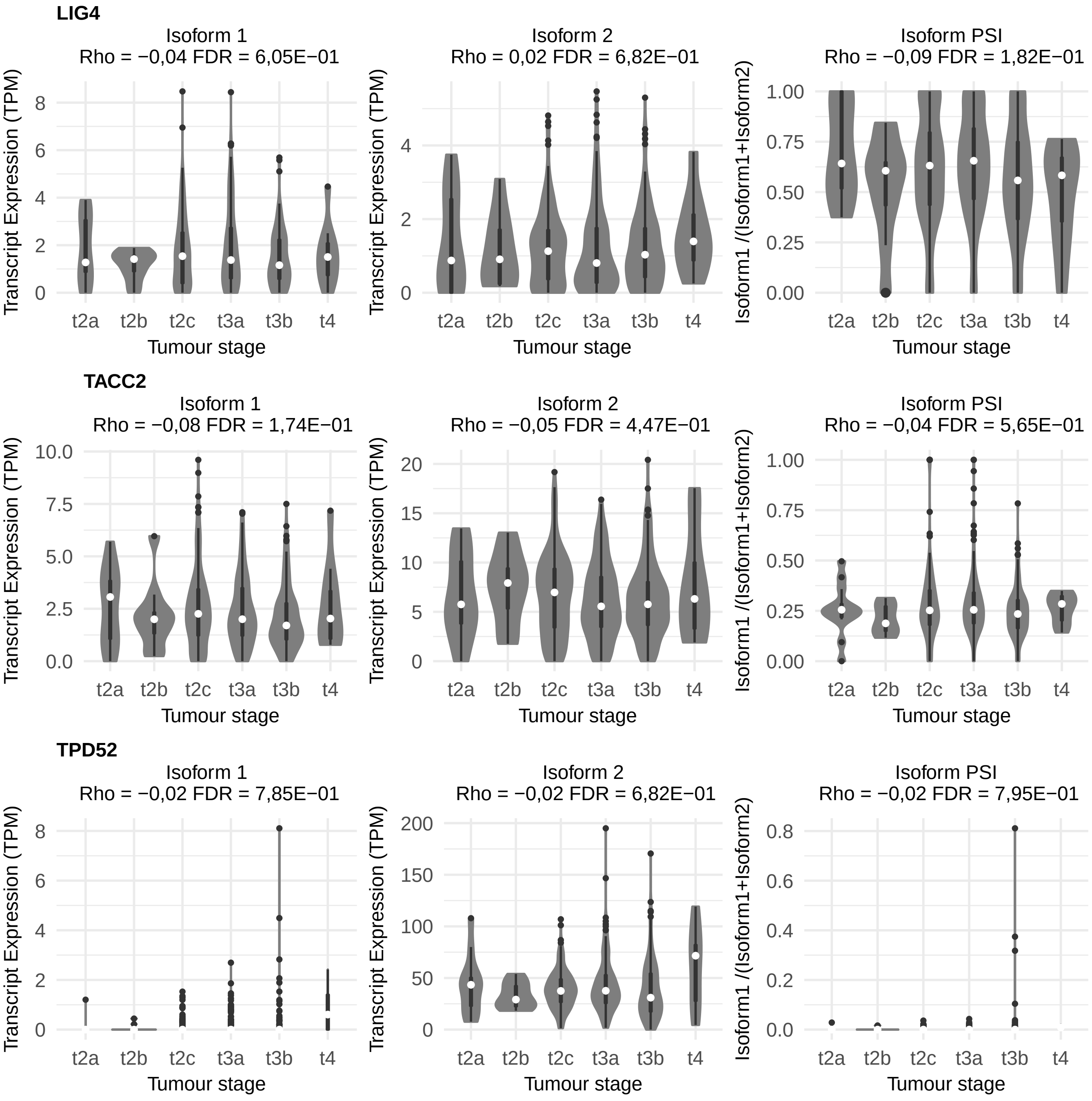

LIG4
Isoform 1
Rho = −0,04 FDR = 6,05E−01
Isoform 2
Rho = 0,02 FDR = 6,82E−01
Isoform PSI
Rho = −0,09 FDR = 1,82E−01
TACC2
Isoform 1
Rho = −0,08 FDR = 1,74E−01
Isoform 2
Rho = −0,05 FDR = 4,47E−01
Isoform PSI
Rho = −0,04 FDR = 5,65E−01
TPD52
Isoform 1
Rho = −0,02 FDR = 7,85E−01
Isoform 2
Rho = −0,02 FDR = 6,82E−01
Isoform PSI
Rho = −0,02 FDR = 7,95E−01
Transcript Expression (TPM)
Isoform1 /(Isoform1+Isoform2)
t2a
t2b
t2c
t3a
t3b
t4
Tumour stage

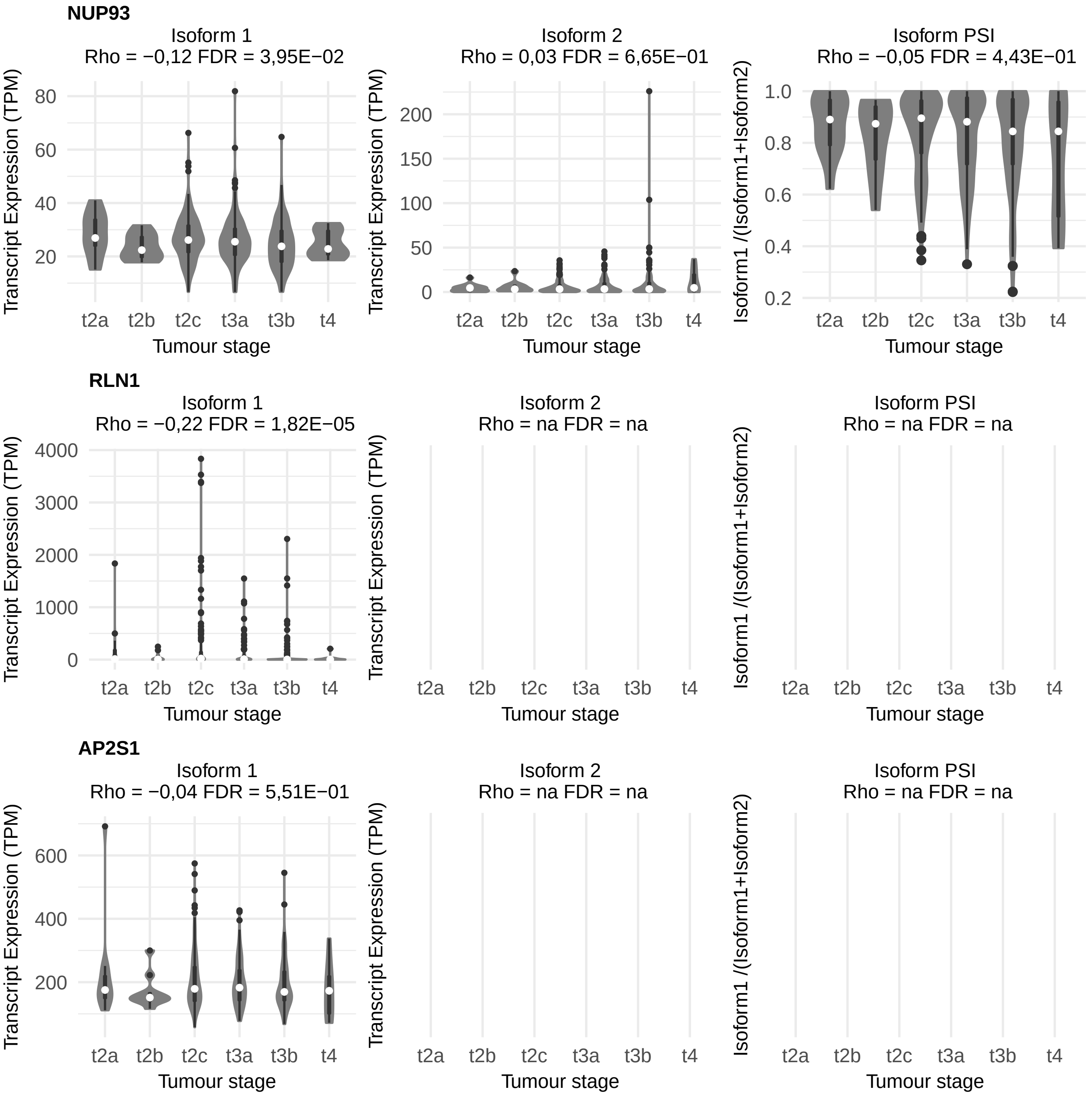
NUP93
Isoform 1
Rho = −0,12 FDR = 3,95E−02
Transcript Expression (TPM)
80
60
40
20
t2a t2b t2c t3a t3b t4
Tumour stage
Isoform 2
Rho = 0,03 FDR = 6,65E−01
Transcript Expression (TPM)
200
150
100
50
0
t2a t2b t2c t3a t3b t4
Tumour stage
Isoform PSI
Rho = −0,05 FDR = 4,43E−01
Isoform1 /(Isoform1+Isoform2)
1.0
0.8
0.6
0.4
0.2
t2a t2b t2c t3a t3b t4
Tumour stage
RLN1
Isoform 1
Rho = −0,22 FDR = 1,82E−05
Transcript Expression (TPM)
4000
3000
2000
1000
0
t2a t2b t2c t3a t3b t4
Tumour stage
Isoform 2
Rho = na FDR = na
Transcript Expression (TPM)
t2a t2b t2c t3a t3b t4
Tumour stage
Isoform PSI
Rho = na FDR = na
Isoform1 /(Isoform1+Isoform2)
t2a t2b t2c t3a t3b t4
Tumour stage
AP2S1
Isoform 1
Rho = −0,04 FDR = 5,51E−01
Transcript Expression (TPM)
600
400
200
t2a t2b t2c t3a t3b t4
Tumour stage
Isoform 2
Rho = na FDR = na
Transcript Expression (TPM)
t2a t2b t2c t3a t3b t4
Tumour stage
Isoform PSI
Rho = na FDR = na
Isoform1 /(Isoform1+Isoform2)
t2a t2b t2c t3a t3b t4
Tumour stage

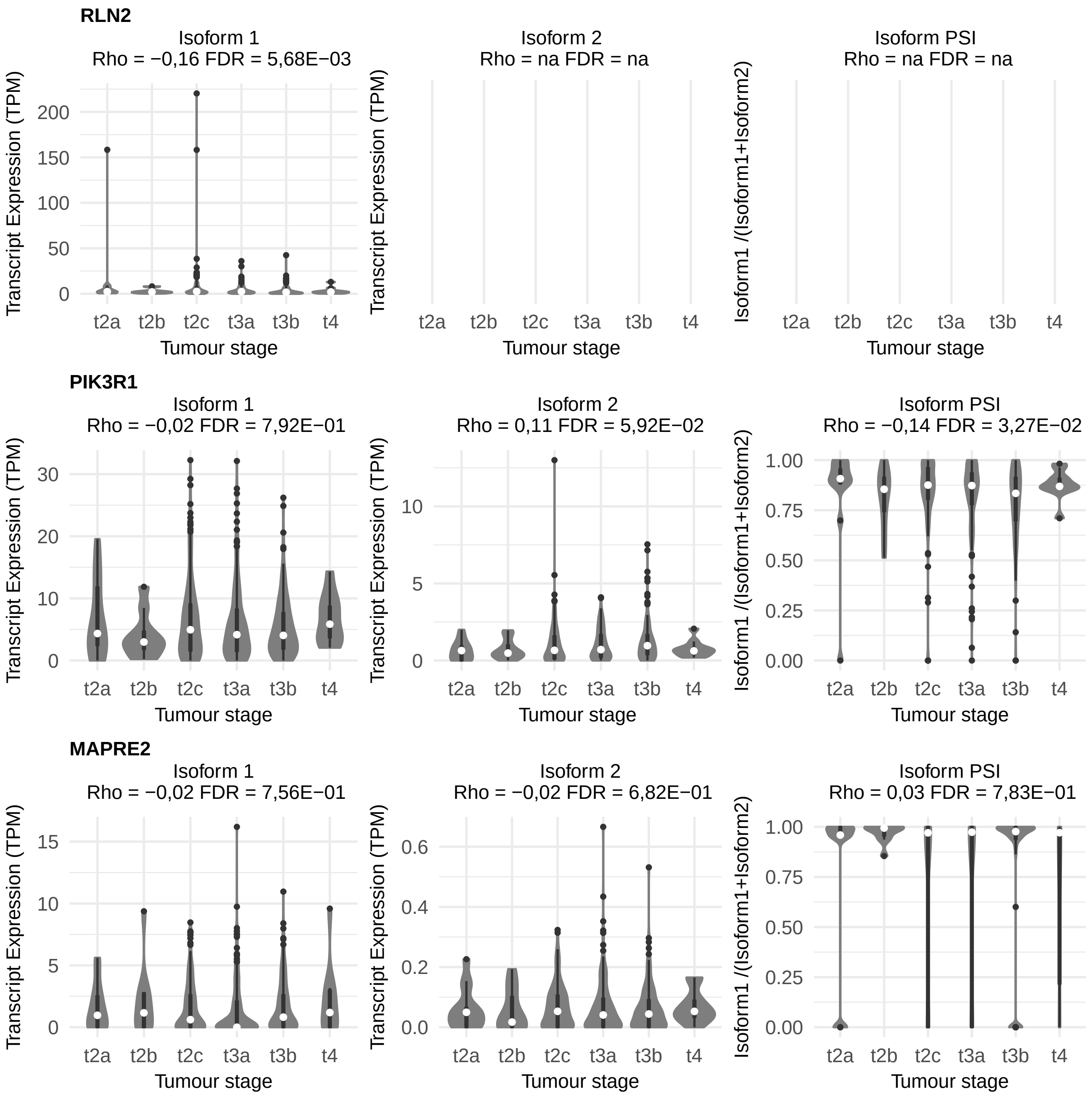

RLN2
Isoform 1
Rho = −0,16 FDR = 5,68E−03
Isoform 2
Rho = na FDR = na
Isoform PSI
Rho = na FDR = na
PIK3R1
Isoform 1
Rho = −0,02 FDR = 7,92E−01
Isoform 2
Rho = 0,11 FDR = 5,92E−02
Isoform PSI
Rho = −0,14 FDR = 3,27E−02
MAPRE2
Isoform 1
Rho = −0,02 FDR = 7,56E−01
Isoform 2
Rho = −0,02 FDR = 6,82E−01
Isoform PSI
Rho = 0,03 FDR = 7,83E−01
Transcript Expression (TPM)
Isoform1 /(Isoform1+Isoform2)
Tumour stage
t2a
t2b
t2c
t3a
t3b
t4

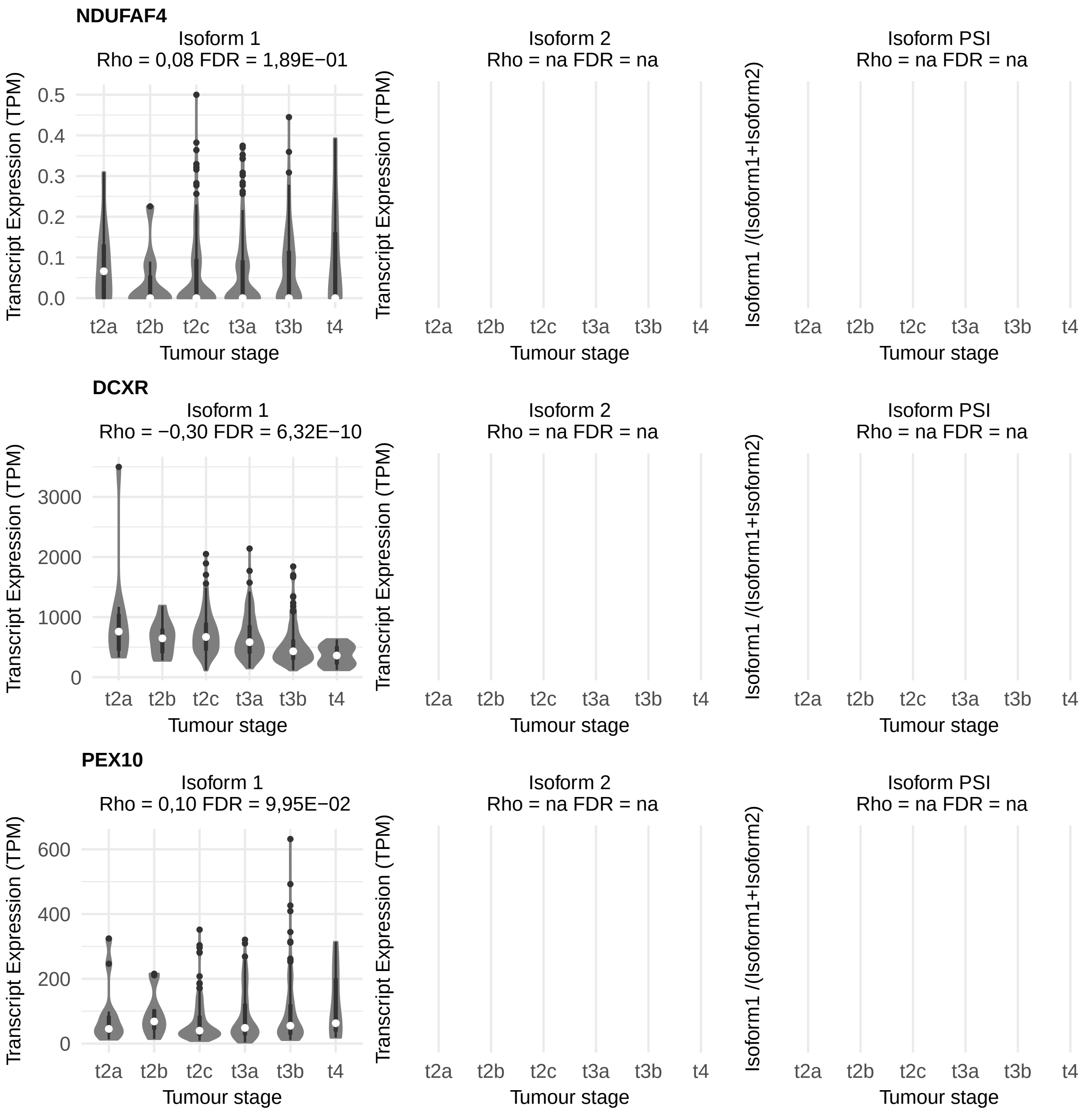

NDUFAF4
Isoform 1
Rho = 0,08 FDR = 1,89E−01
Isoform 2
Rho = na FDR = na
Isoform PSI
Rho = na FDR = na
DCXR
Isoform 1
Rho = −0,30 FDR = 6,32E−10
Isoform 2
Rho = na FDR = na
Isoform PSI
Rho = na FDR = na
PEX10
Isoform 1
Rho = 0,10 FDR = 9,95E−02
Isoform 2
Rho = na FDR = na
Isoform PSI
Rho = na FDR = na
Transcript Expression (TPM)
Isoform1 /(Isoform1+Isoform2)
t2a
t2b
t2c
t3a
t3b
t4
Tumour stage

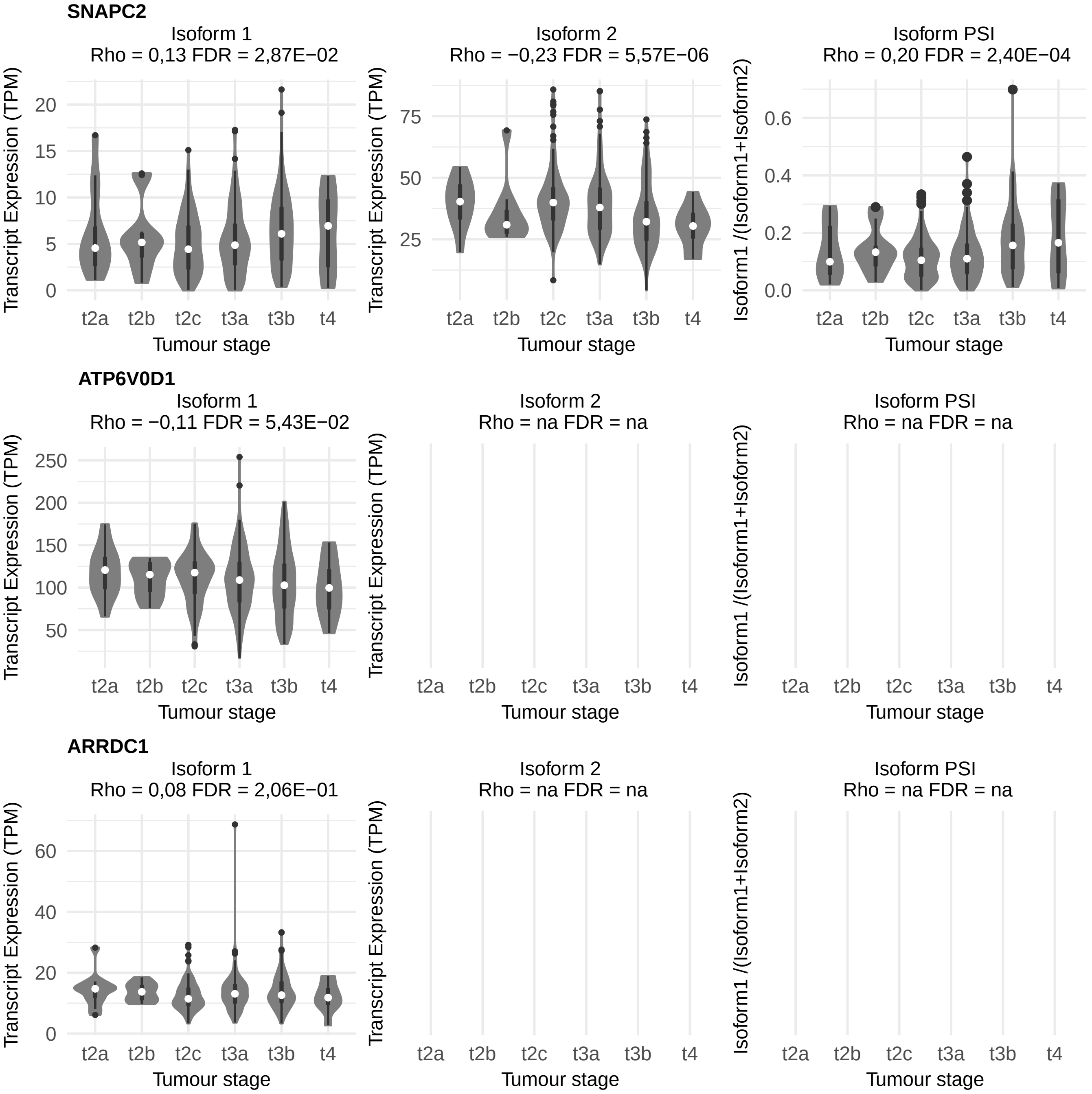
SNAPC2
Isoform 1
Rho = 0,13 FDR = 2,87E−02
Isoform 2
Rho = −0,23 FDR = 5,57E−06
Isoform PSI
Rho = 0,20 FDR = 2,40E−04
ATP6V0D1
Isoform 1
Rho = −0,11 FDR = 5,43E−02
Isoform 2
Rho = na FDR = na
Isoform PSI
Rho = na FDR = na
ARRDC1
Isoform 1
Rho = 0,08 FDR = 2,06E−01
Isoform 2
Rho = na FDR = na
Isoform PSI
Rho = na FDR = na
Transcript Expression (TPM)
Isoform1 /(Isoform1+Isoform2)
Tumour stage
t2a
t2b
t2c
t3a
t3b
t4

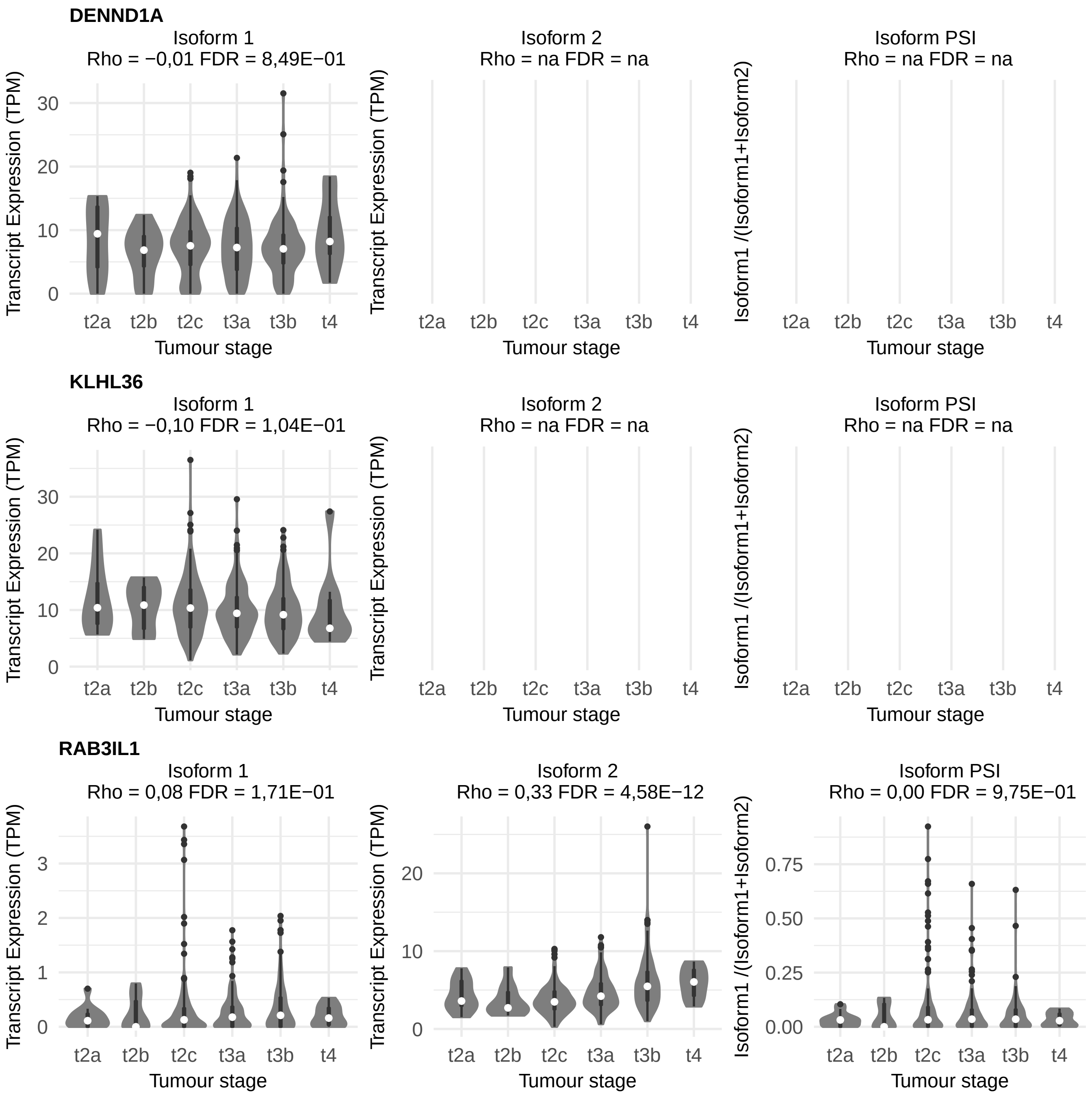
DENND1A
Isoform 1
Rho = −0,01 FDR = 8,49E−01
Isoform 2
Rho = na FDR = na
Isoform PSI
Rho = na FDR = na
KLHL36
Isoform 1
Rho = −0,10 FDR = 1,04E−01
Isoform 2
Rho = na FDR = na
Isoform PSI
Rho = na FDR = na
RAB3IL1
Isoform 1
Rho = 0,08 FDR = 1,71E−01
Isoform 2
Rho = 0,33 FDR = 4,58E−12
Isoform PSI
Rho = 0,00 FDR = 9,75E−01
Transcript Expression (TPM)
Isoform1 /(Isoform1+Isoform2)
t2a
t2b
t2c
t3a
t3b
t4
Tumour stage
0
10
20
30
0
1
2
3
0.00
0.25
0.50
0.75

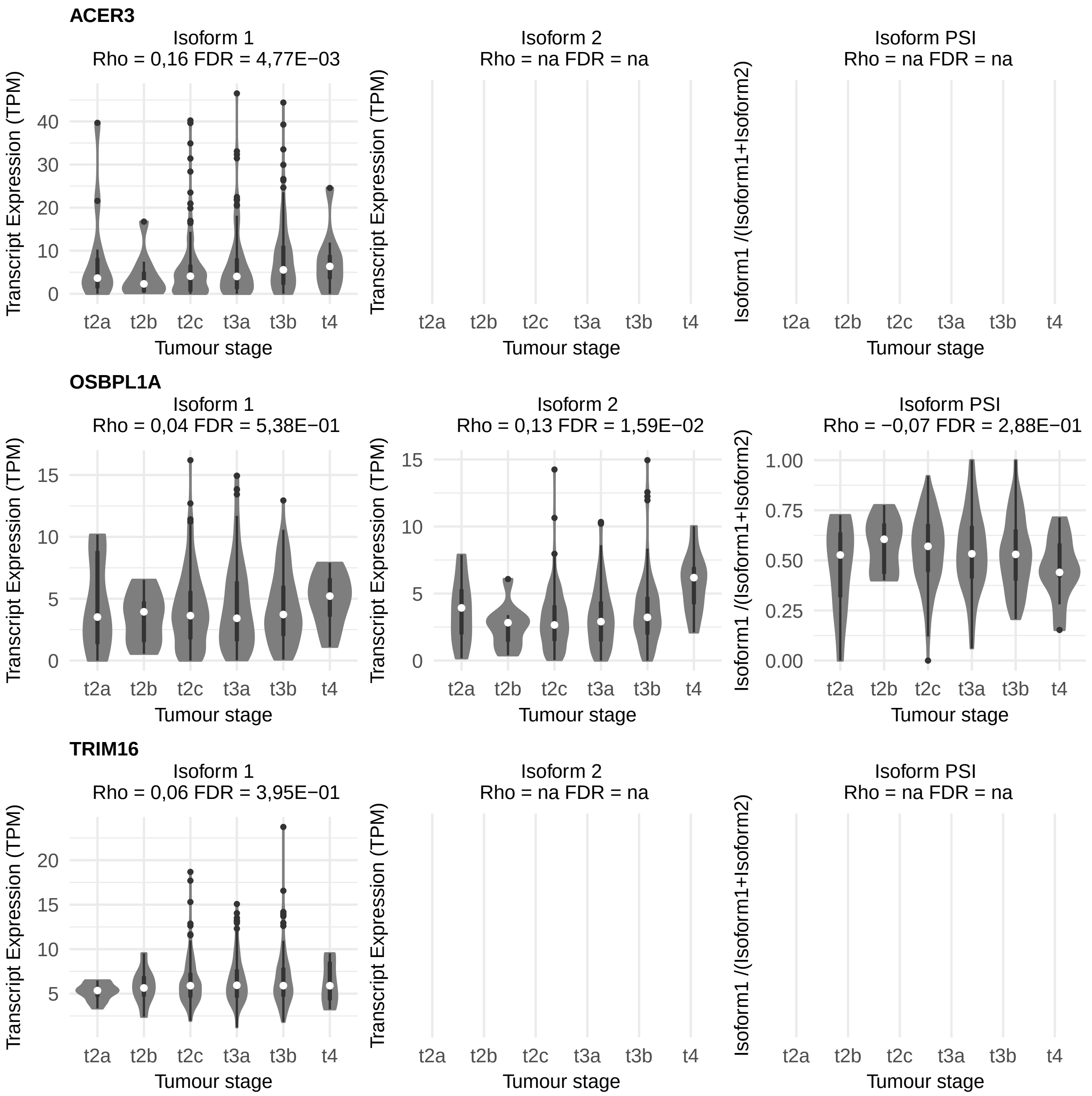
ACER3
Isoform 1
Rho = 0,16 FDR = 4,77E−03
Isoform 2
Rho = na FDR = na
Isoform PSI
Rho = na FDR = na
OSBPL1A
Isoform 1
Rho = 0,04 FDR = 5,38E−01
Isoform 2
Rho = 0,13 FDR = 1,59E−02
Isoform PSI
Rho = −0,07 FDR = 2,88E−01
TRIM16
Isoform 1
Rho = 0,06 FDR = 3,95E−01
Isoform 2
Rho = na FDR = na
Isoform PSI
Rho = na FDR = na
Transcript Expression (TPM)
Isoform1 /(Isoform1+Isoform2)
t2a
t2b
t2c
t3a
t3b
t4
Tumour stage

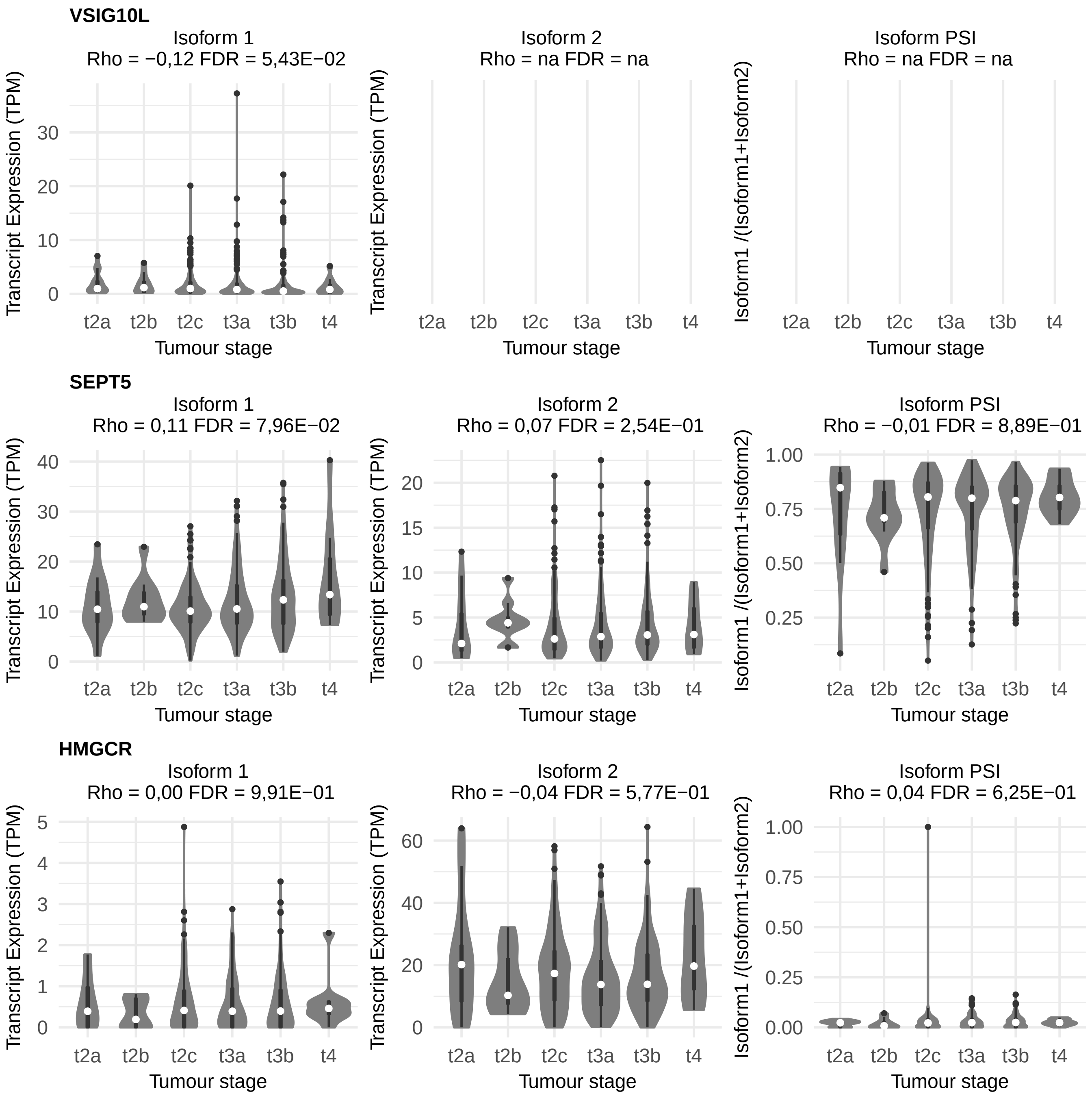

VSIG10L
Isoform 1
Rho = −0,12 FDR = 5,43E−02
Isoform 2
Rho = na FDR = na
Isoform PSI
Rho = na FDR = na
SEPT5
Isoform 1
Rho = 0,11 FDR = 7,96E−02
Isoform 2
Rho = 0,07 FDR = 2,54E−01
Isoform PSI
Rho = −0,01 FDR = 8,89E−01
HMGCR
Isoform 1
Rho = 0,00 FDR = 9,91E−01
Isoform 2
Rho = −0,04 FDR = 5,77E−01
Isoform PSI
Rho = 0,04 FDR = 6,25E−01
Transcript Expression (TPM)
Isoform1 /(Isoform1+Isoform2)
Tumour stage
t2a
t2b
t2c
t3a
t3b
t4

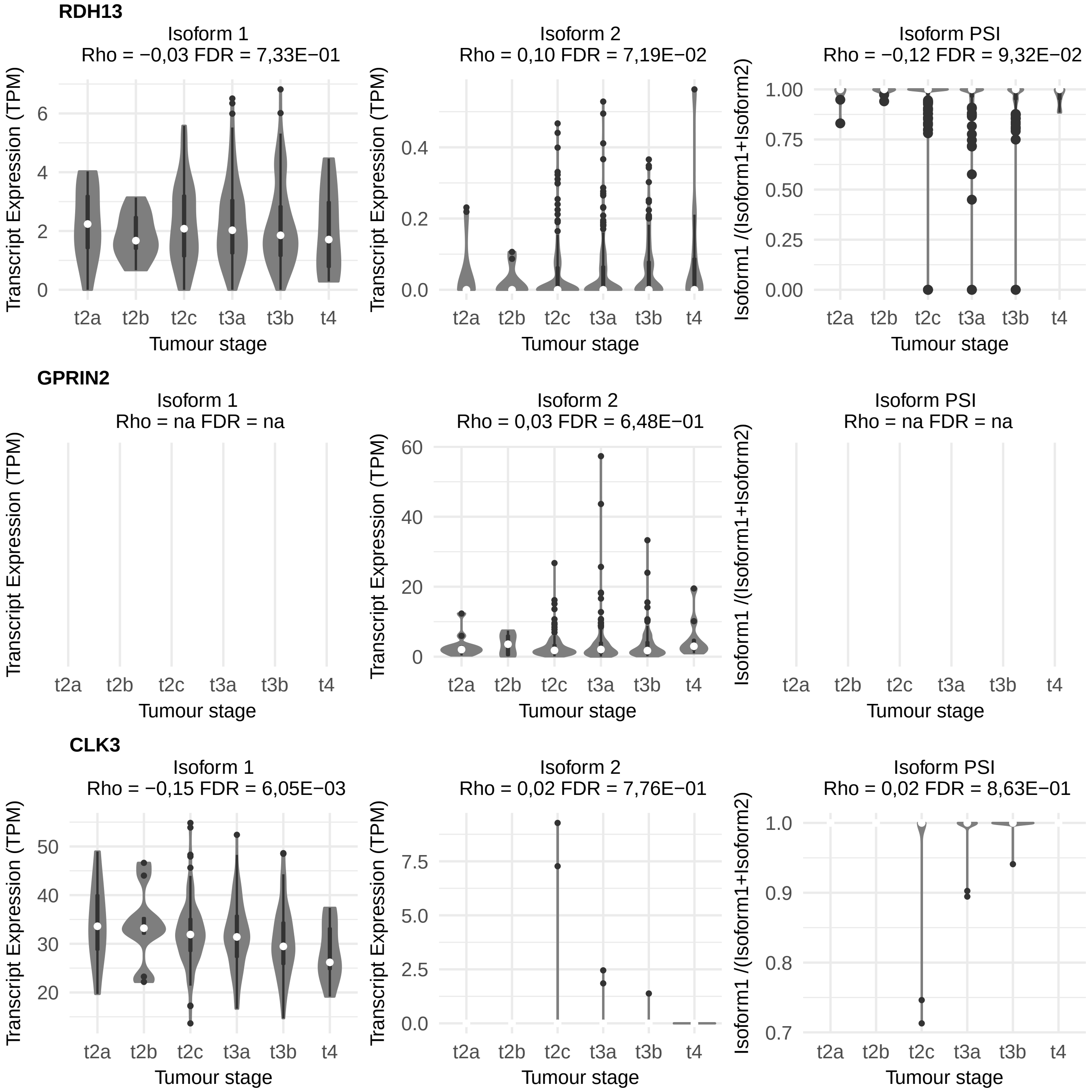
RDH13
Isoform 1
Rho = −0,03 FDR = 7,33E−01
Isoform 2
Rho = 0,10 FDR = 7,19E−02
Isoform PSI
Rho = −0,12 FDR = 9,32E−02
GPRIN2
Isoform 1
Rho = na FDR = na
Isoform 2
Rho = 0,03 FDR = 6,48E−01
Isoform PSI
Rho = na FDR = na
CLK3
Isoform 1
Rho = −0,15 FDR = 6,05E−03
Isoform 2
Rho = 0,02 FDR = 7,76E−01
Isoform PSI
Rho = 0,02 FDR = 8,63E−01
Transcript Expression (TPM)
Isoform1 /(Isoform1+Isoform2)
Tumour stage
t2a
t2b
t2c
t3a
t3b
t4

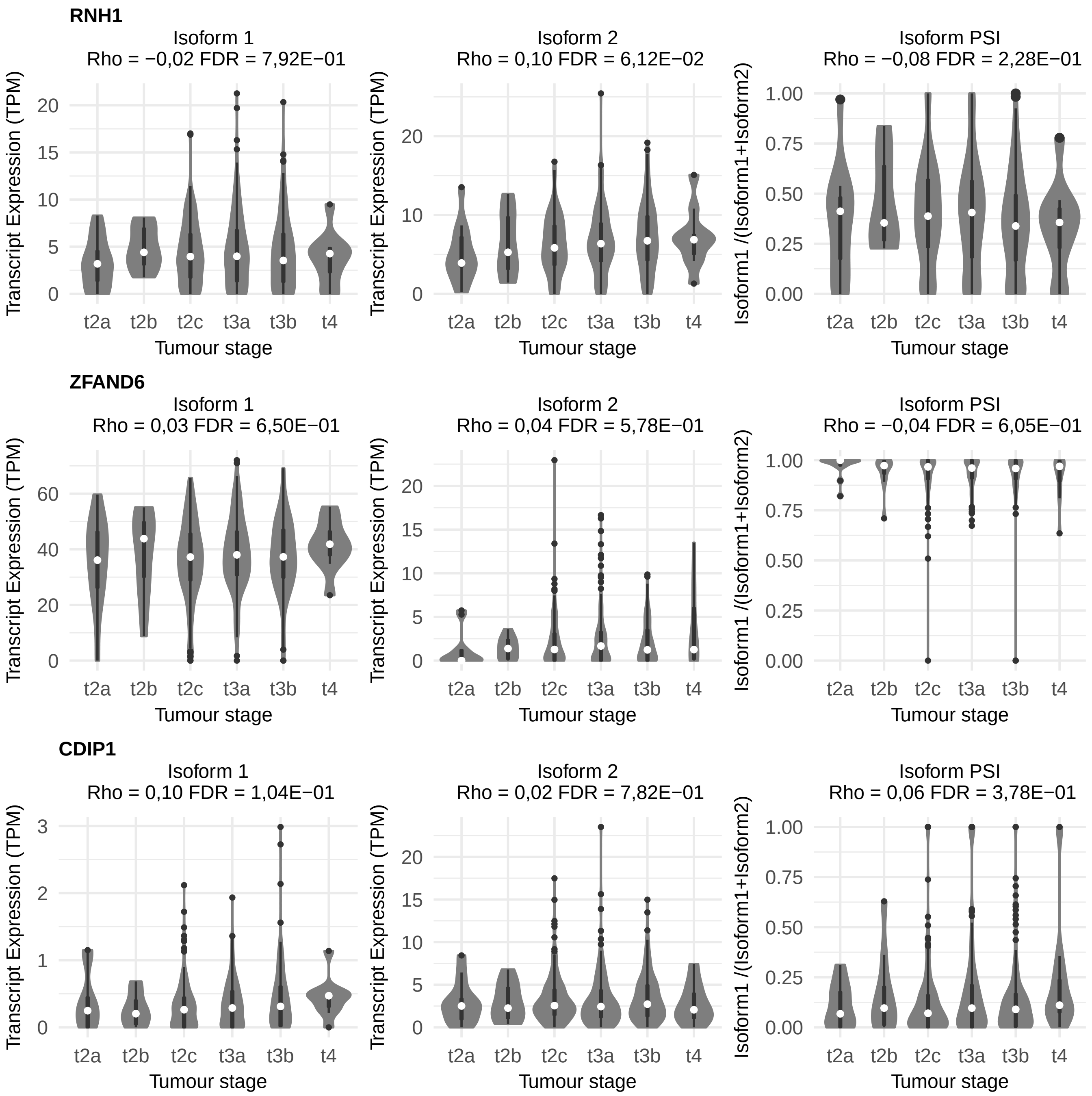

RNH1
Isoform 1
Rho = −0,02 FDR = 7,92E−01
Isoform 2
Rho = 0,10 FDR = 6,12E−02
Isoform PSI
Rho = −0,08 FDR = 2,28E−01
ZFAND6
Isoform 1
Rho = 0,03 FDR = 6,50E−01
Isoform 2
Rho = 0,04 FDR = 5,78E−01
Isoform PSI
Rho = −0,04 FDR = 6,05E−01
CDIP1
Isoform 1
Rho = 0,10 FDR = 1,04E−01
Isoform 2
Rho = 0,02 FDR = 7,82E−01
Isoform PSI
Rho = 0,06 FDR = 3,78E−01
Transcript Expression (TPM)
Isoform1 /(Isoform1+Isoform2)
Tumour stage
t2a
t2b
t2c
t3a
t3b
t4

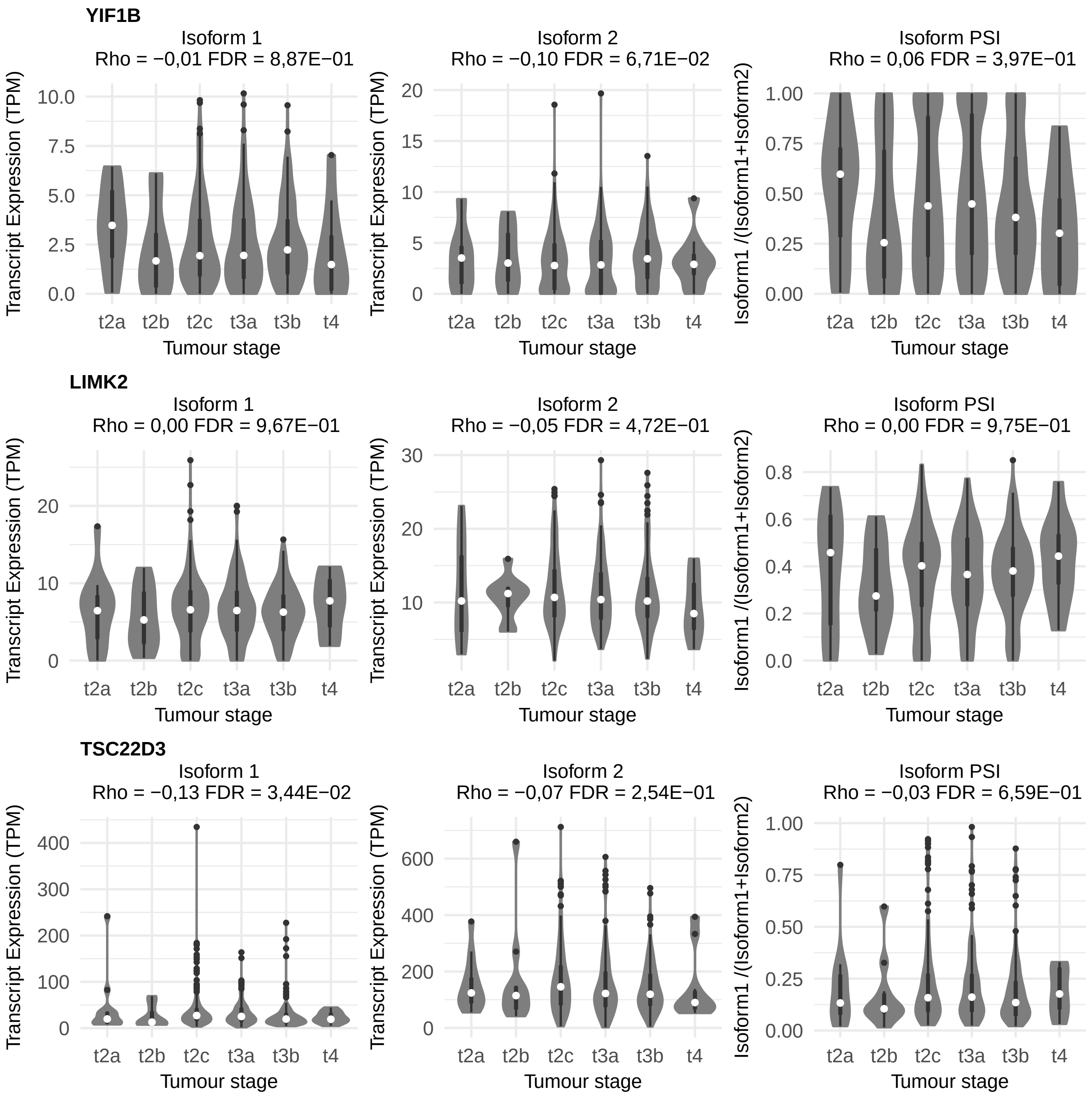

YIF1B
Isoform 1
Rho = −0,01 FDR = 8,87E−01
Isoform 2
Rho = −0,10 FDR = 6,71E−02
Isoform PSI
Rho = 0,06 FDR = 3,97E−01
LIMK2
Isoform 1
Rho = 0,00 FDR = 9,67E−01
Isoform 2
Rho = −0,05 FDR = 4,72E−01
Isoform PSI
Rho = 0,00 FDR = 9,75E−01
TSC22D3
Isoform 1
Rho = −0,13 FDR = 3,44E−02
Isoform 2
Rho = −0,07 FDR = 2,54E−01
Isoform PSI
Rho = −0,03 FDR = 6,59E−01
Transcript Expression (TPM)
Isoform1 /(Isoform1+Isoform2)
t2a
t2b
t2c
t3a
t3b
t4
Tumour stage

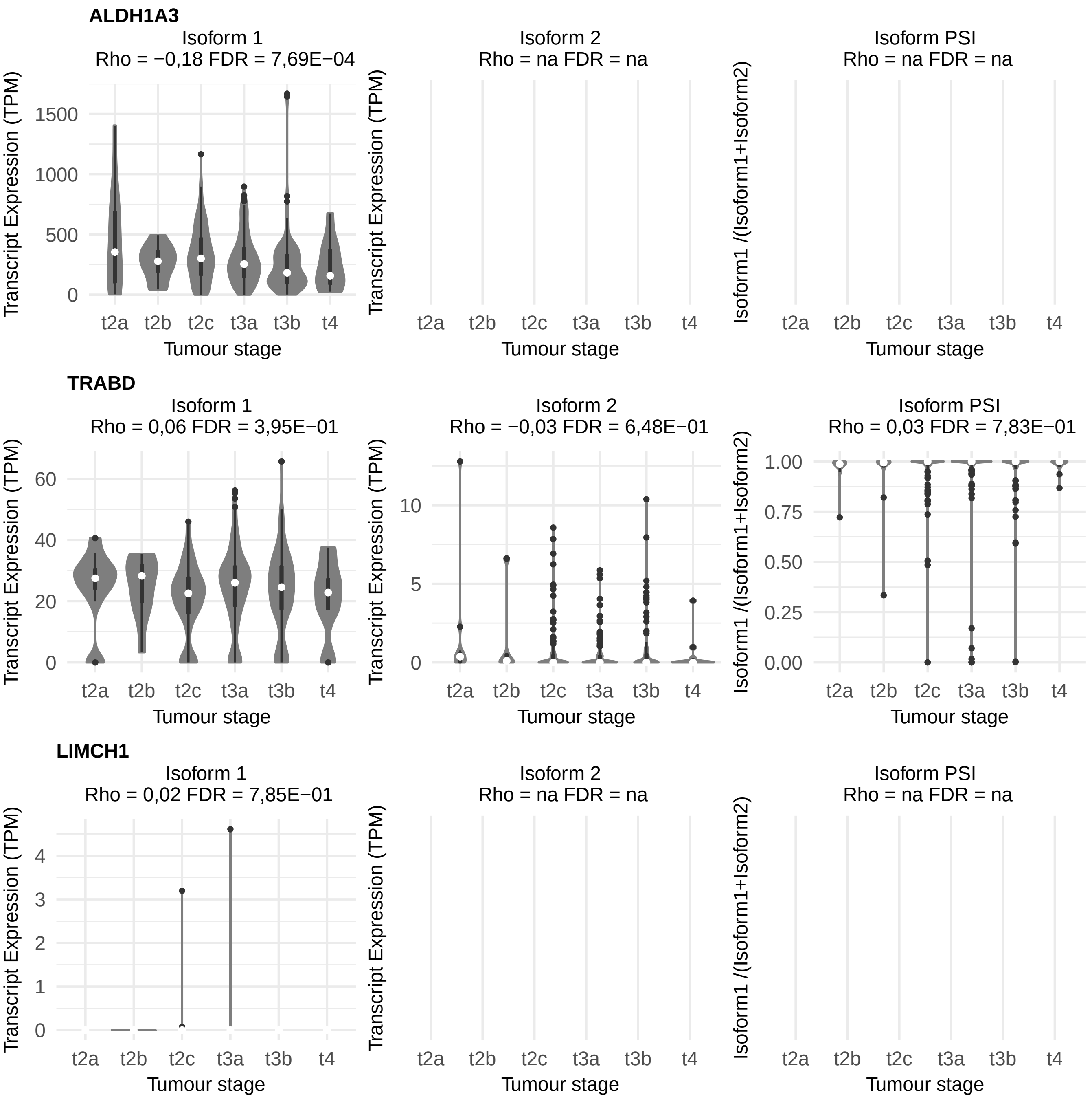
ALDH1A3
Isoform 1
Rho = −0,18 FDR = 7,69E−04
Isoform 2
Rho = na FDR = na
Isoform PSI
Rho = na FDR = na
TRABD
Isoform 1
Rho = 0,06 FDR = 3,95E−01
Isoform 2
Rho = −0,03 FDR = 6,48E−01
Isoform PSI
Rho = 0,03 FDR = 7,83E−01
LIMCH1
Isoform 1
Rho = 0,02 FDR = 7,85E−01
Isoform 2
Rho = na FDR = na
Isoform PSI
Rho = na FDR = na
Transcript Expression (TPM)
Isoform1 /(Isoform1+Isoform2)
Tumour stage
t2a
t2b
t2c
t3a
t3b
t4
1500
1000
500
0
60
40
20
10
5
1.00
0.75
0.50
0.25
0.00
4
3
2
1

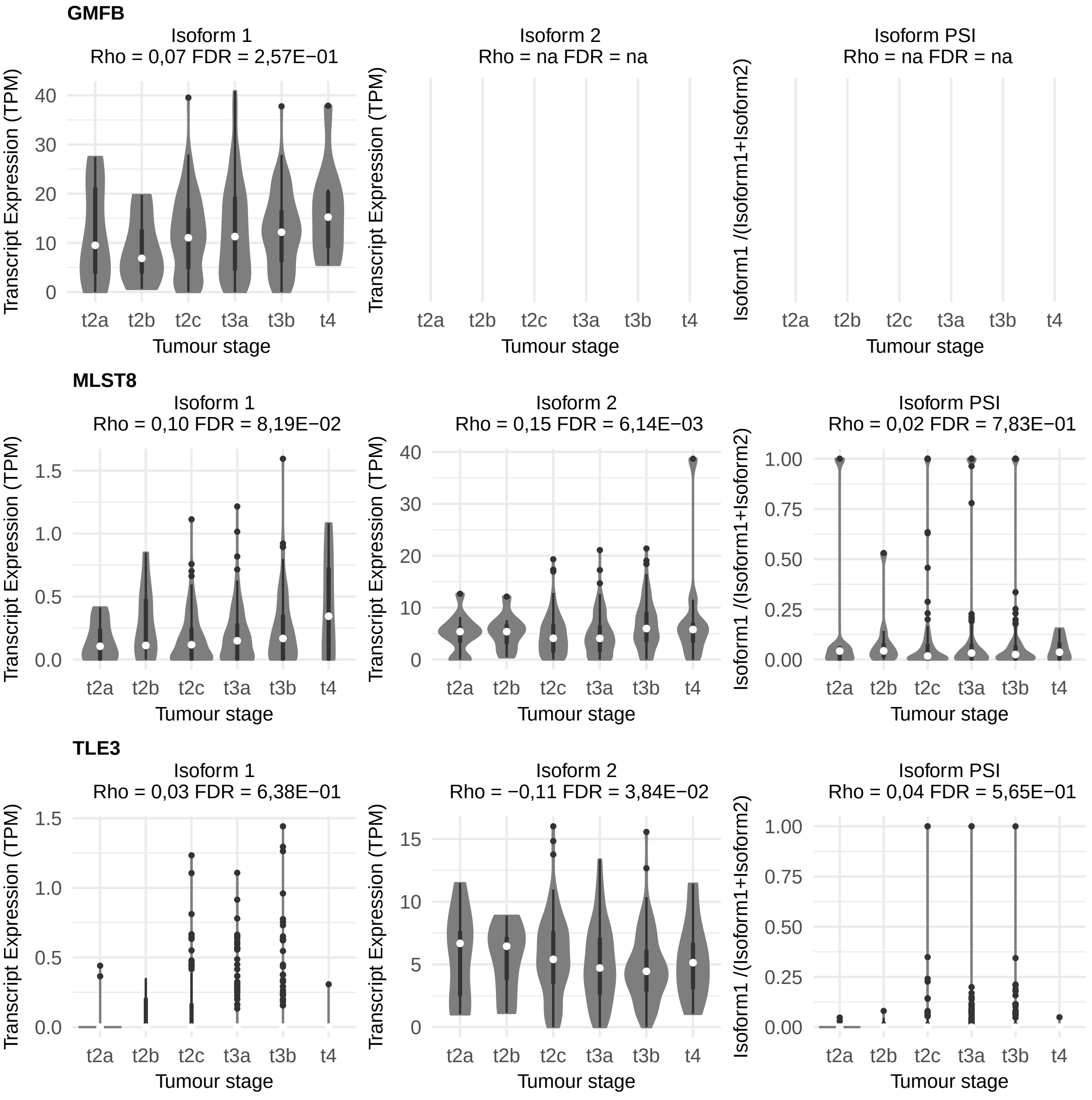

GMFB
Isoform 1
Rho = 0,07 FDR = 2,57E−01
Isoform 2
Rho = na FDR = na
Isoform PSI
Rho = na FDR = na
MLST8
Isoform 1
Rho = 0,10 FDR = 8,19E−02
Isoform 2
Rho = 0,15 FDR = 6,14E−03
Isoform PSI
Rho = 0,02 FDR = 7,83E−01
TLE3
Isoform 1
Rho = 0,03 FDR = 6,38E−01
Isoform 2
Rho = −0,11 FDR = 3,84E−02
Isoform PSI
Rho = 0,04 FDR = 5,65E−01
Transcript Expression (TPM)
Isoform1 /(Isoform1+Isoform2)
Tumour stage
t2a
t2b
t2c
t3a
t3b
t4

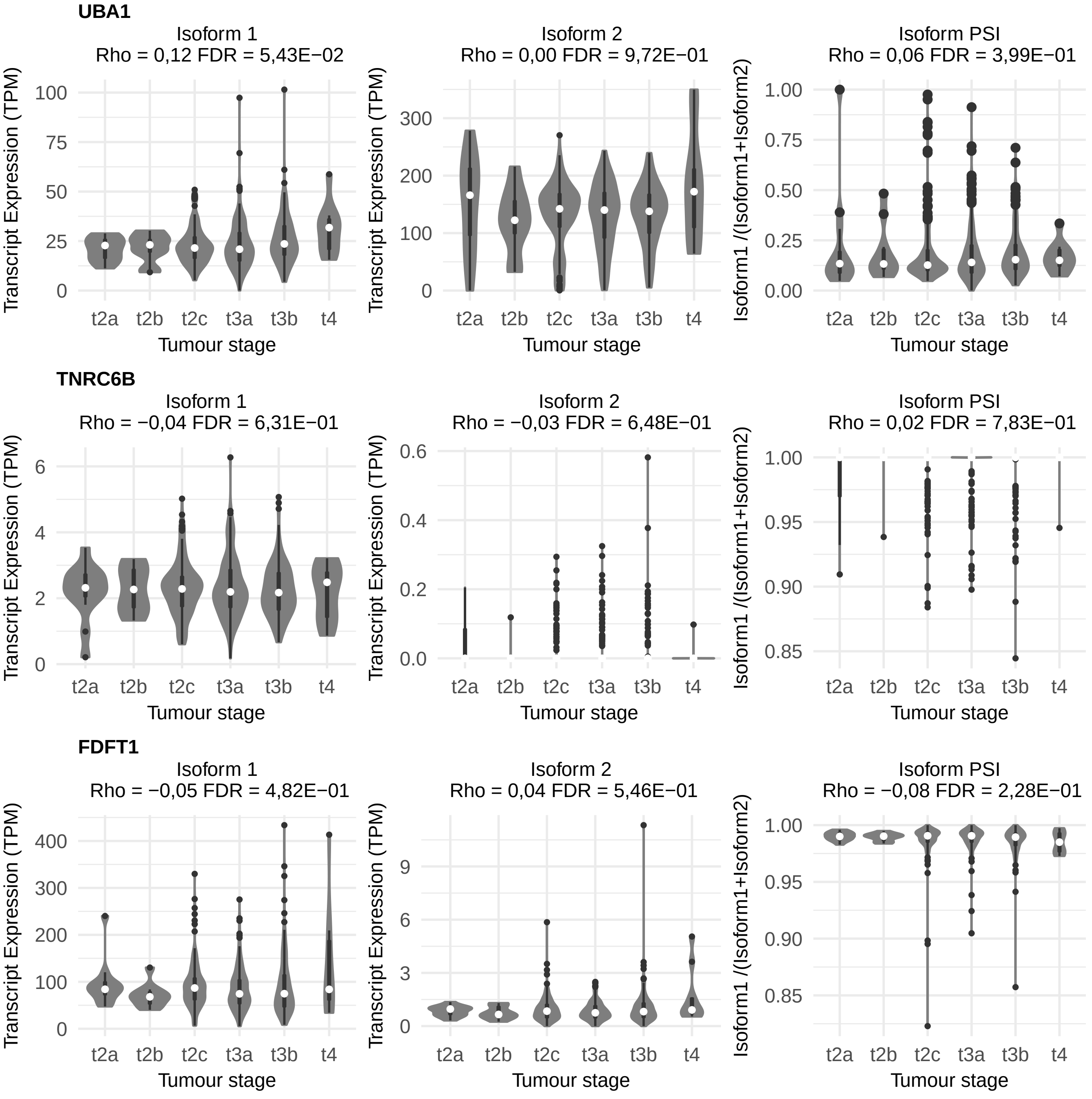
UBA1
Isoform 1
Rho = 0,12 FDR = 5,43E−02
Isoform 2
Rho = 0,00 FDR = 9,72E−01
Isoform PSI
Rho = 0,06 FDR = 3,99E−01
TNRC6B
Isoform 1
Rho = −0,04 FDR = 6,31E−01
Isoform 2
Rho = −0,03 FDR = 6,48E−01
Isoform PSI
Rho = 0,02 FDR = 7,83E−01
FDFT1
Isoform 1
Rho = −0,05 FDR = 4,82E−01
Isoform 2
Rho = 0,04 FDR = 5,46E−01
Isoform PSI
Rho = −0,08 FDR = 2,28E−01
Transcript Expression (TPM)
Isoform1 /(Isoform1+Isoform2)
t2a t2b t2c t3a t3b t4
Tumour stage

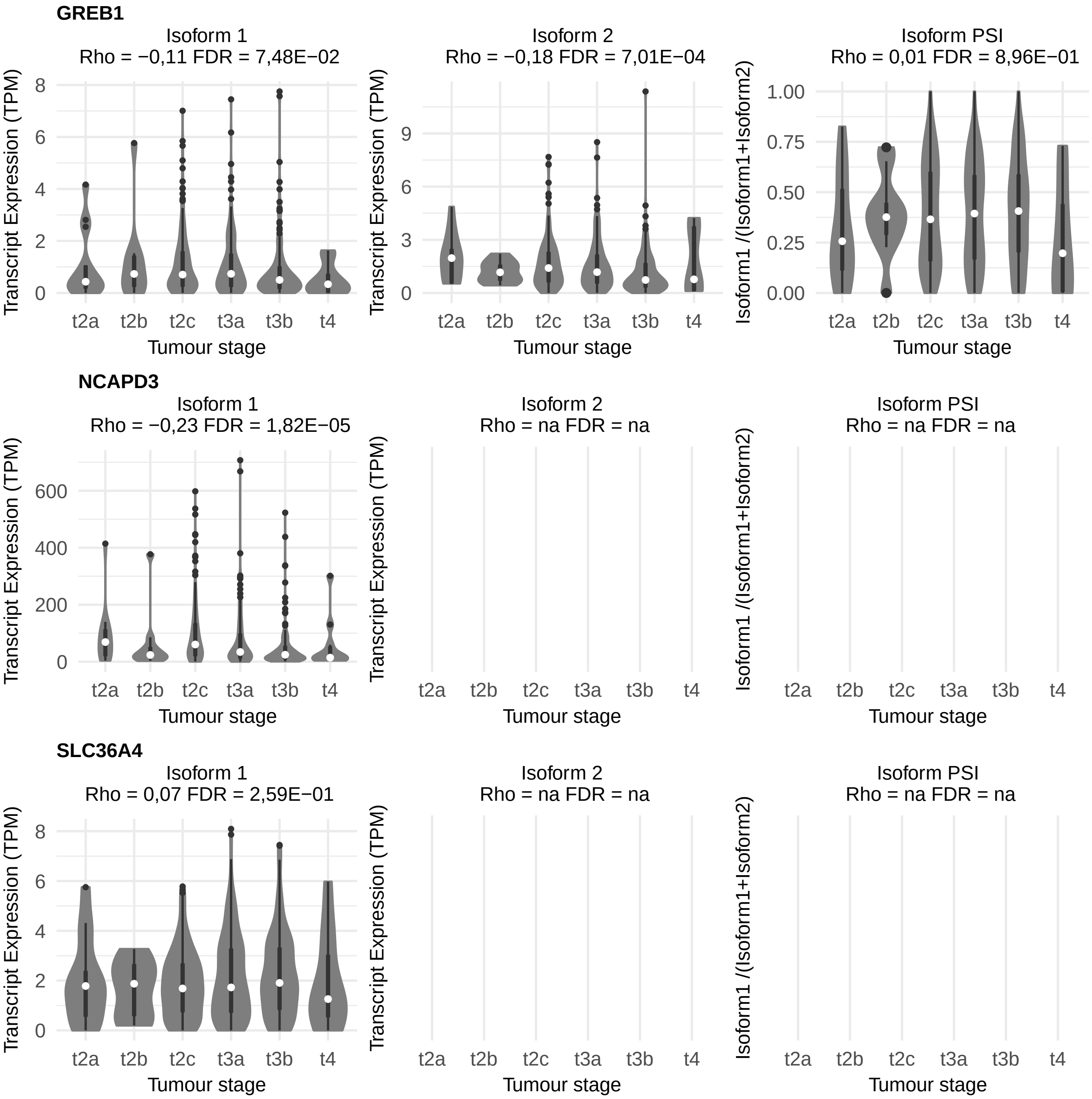
GREB1
Isoform 1
Rho = −0,11 FDR = 7,48E−02
Isoform 2
Rho = −0,18 FDR = 7,01E−04
Isoform PSI
Rho = 0,01 FDR = 8,96E−01
NCAPD3
Isoform 1
Rho = −0,23 FDR = 1,82E−05
Isoform 2
Rho = na FDR = na
Isoform PSI
Rho = na FDR = na
SLC36A4
Isoform 1
Rho = 0,07 FDR = 2,59E−01
Isoform 2
Rho = na FDR = na
Isoform PSI
Rho = na FDR = na
Transcript Expression (TPM)
Isoform1 /(Isoform1+Isoform2)
Tumour stage
t2a
t2b
t2c
t3a
t3b
t4

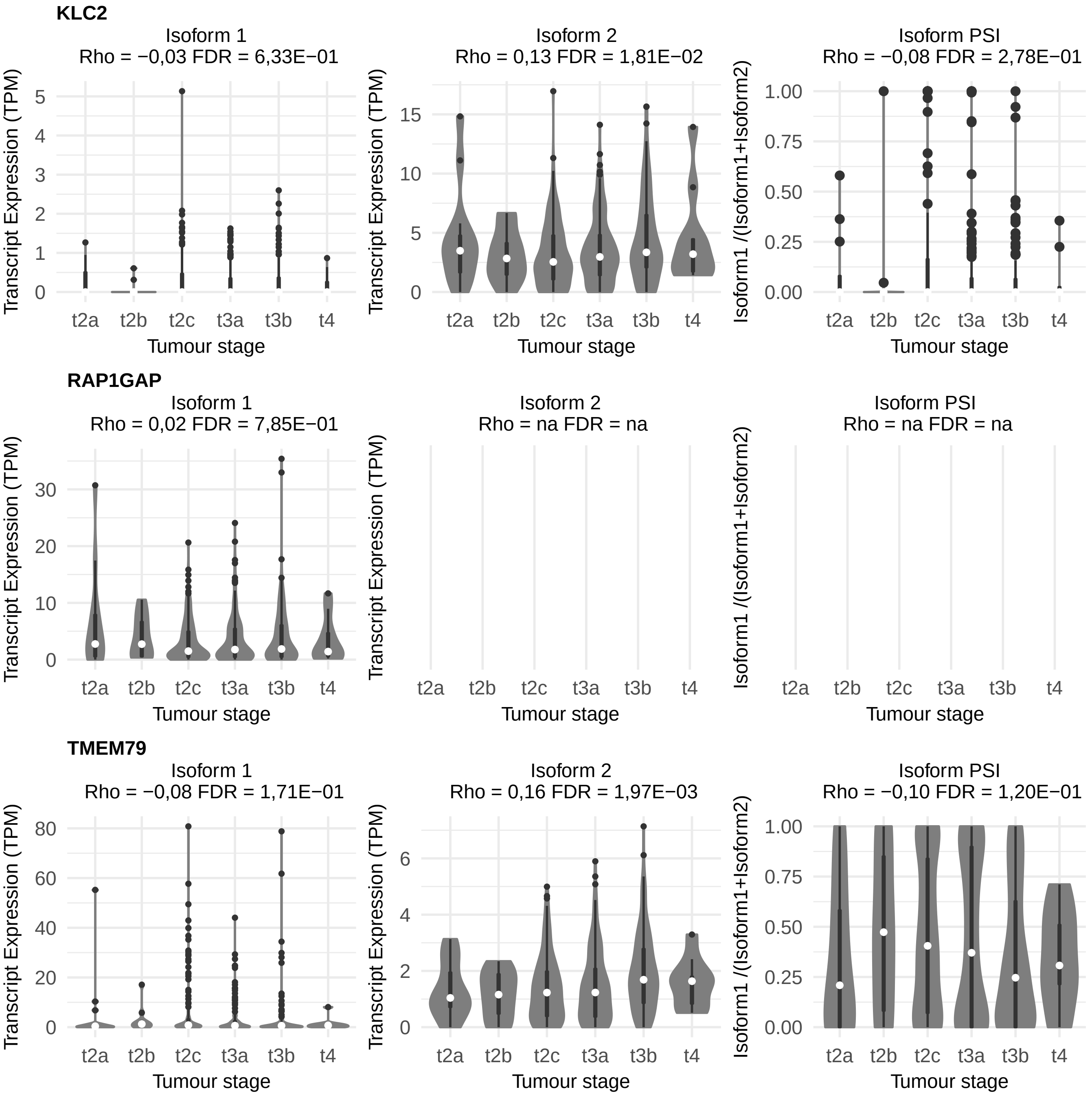
KLC2
Isoform 1
Rho = −0,03 FDR = 6,33E−01
Isoform 2
Rho = 0,13 FDR = 1,81E−02
Isoform PSI
Rho = −0,08 FDR = 2,78E−01
RAP1GAP
Isoform 1
Rho = 0,02 FDR = 7,85E−01
Isoform 2
Rho = na FDR = na
Isoform PSI
Rho = na FDR = na
TMEM79
Isoform 1
Rho = −0,08 FDR = 1,71E−01
Isoform 2
Rho = 0,16 FDR = 1,97E−03
Isoform PSI
Rho = −0,10 FDR = 1,20E−01
Transcript Expression (TPM)
Isoform1 /(Isoform1+Isoform2)
Tumour stage
t2a
t2b
t2c
t3a
t3b
t4

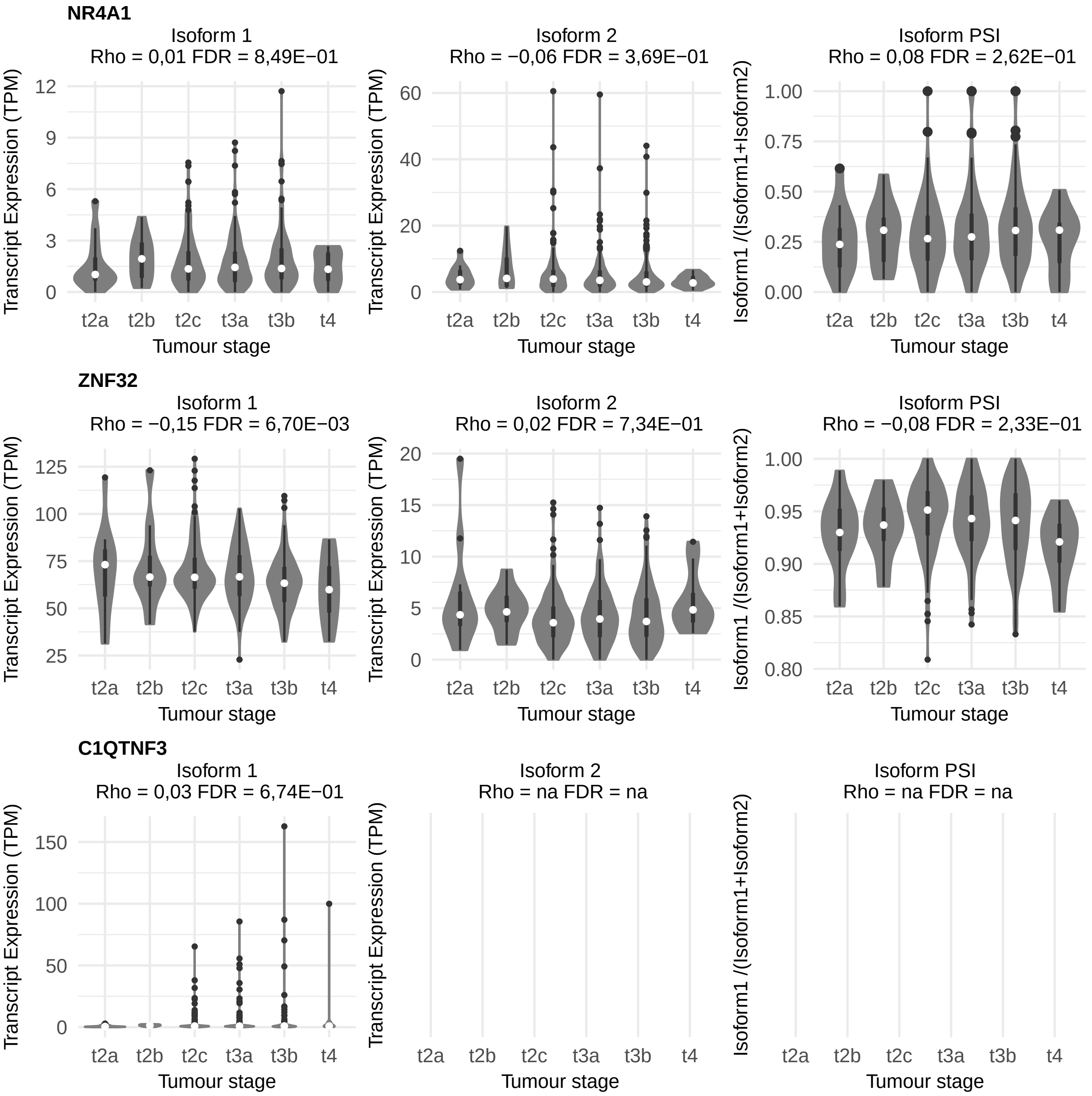
NR4A1
Isoform 1
Rho = 0,01 FDR = 8,49E−01
Isoform 2
Rho = −0,06 FDR = 3,69E−01
Isoform PSI
Rho = 0,08 FDR = 2,62E−01
ZNF32
Isoform 1
Rho = −0,15 FDR = 6,70E−03
Isoform 2
Rho = 0,02 FDR = 7,34E−01
Isoform PSI
Rho = −0,08 FDR = 2,33E−01
C1QTNF3
Isoform 1
Rho = 0,03 FDR = 6,74E−01
Isoform 2
Rho = na FDR = na
Isoform PSI
Rho = na FDR = na
Transcript Expression (TPM)
Isoform1 /(Isoform1+Isoform2)
Tumour stage
t2a
t2b
t2c
t3a
t3b
t4

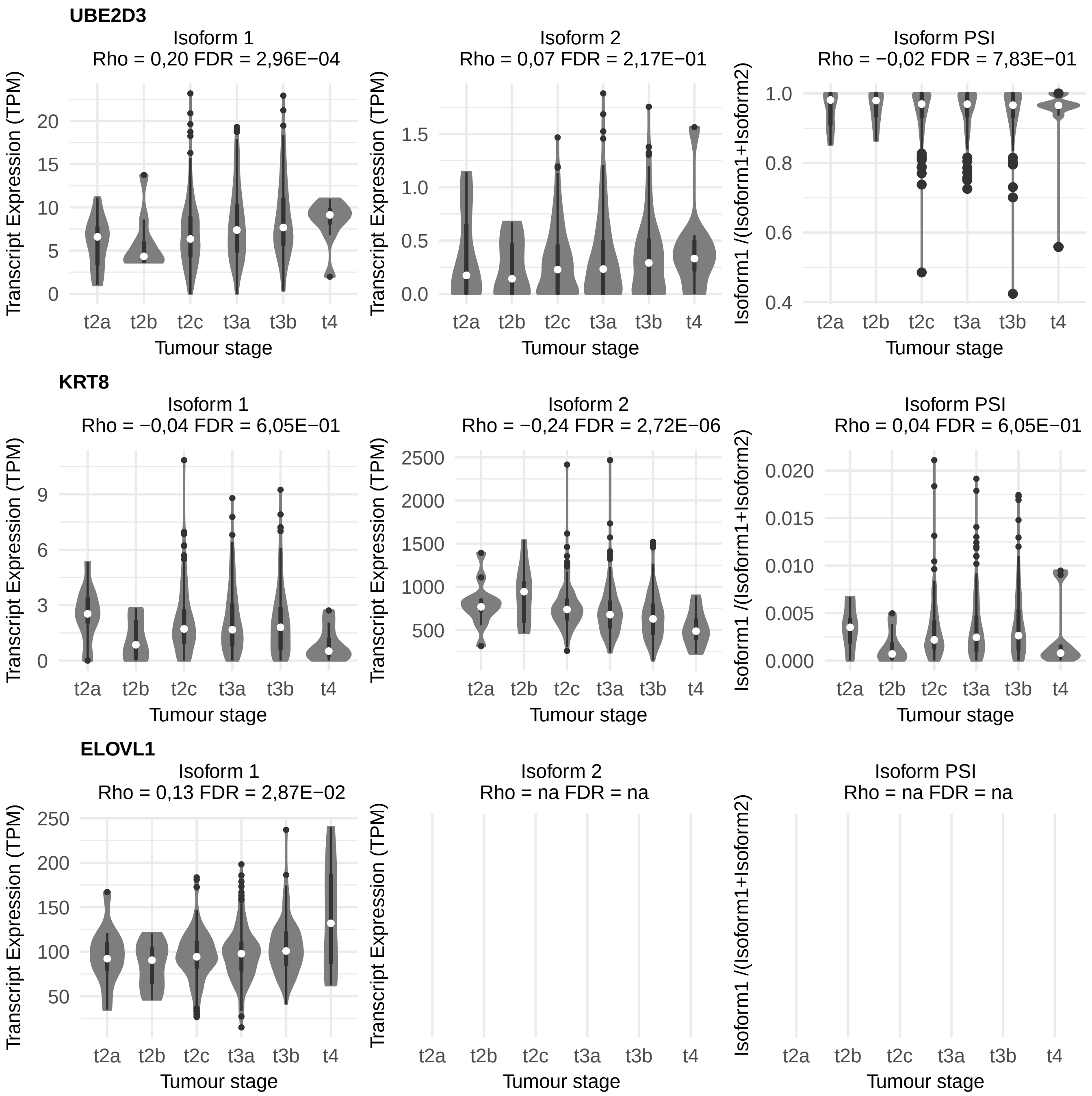

UBE2D3
Isoform 1
Rho = 0,20 FDR = 2,96E−04
Isoform 2
Rho = 0,07 FDR = 2,17E−01
Isoform PSI
Rho = −0,02 FDR = 7,83E−01
KRT8
Isoform 1
Rho = −0,04 FDR = 6,05E−01
Isoform 2
Rho = −0,24 FDR = 2,72E−06
Isoform PSI
Rho = 0,04 FDR = 6,05E−01
ELOVL1
Isoform 1
Rho = 0,13 FDR = 2,87E−02
Isoform 2
Rho = na FDR = na
Isoform PSI
Rho = na FDR = na
Transcript Expression (TPM)
Isoform1 /(Isoform1+Isoform2)
t2a
t2b
t2c
t3a
t3b
t4
Tumour stage

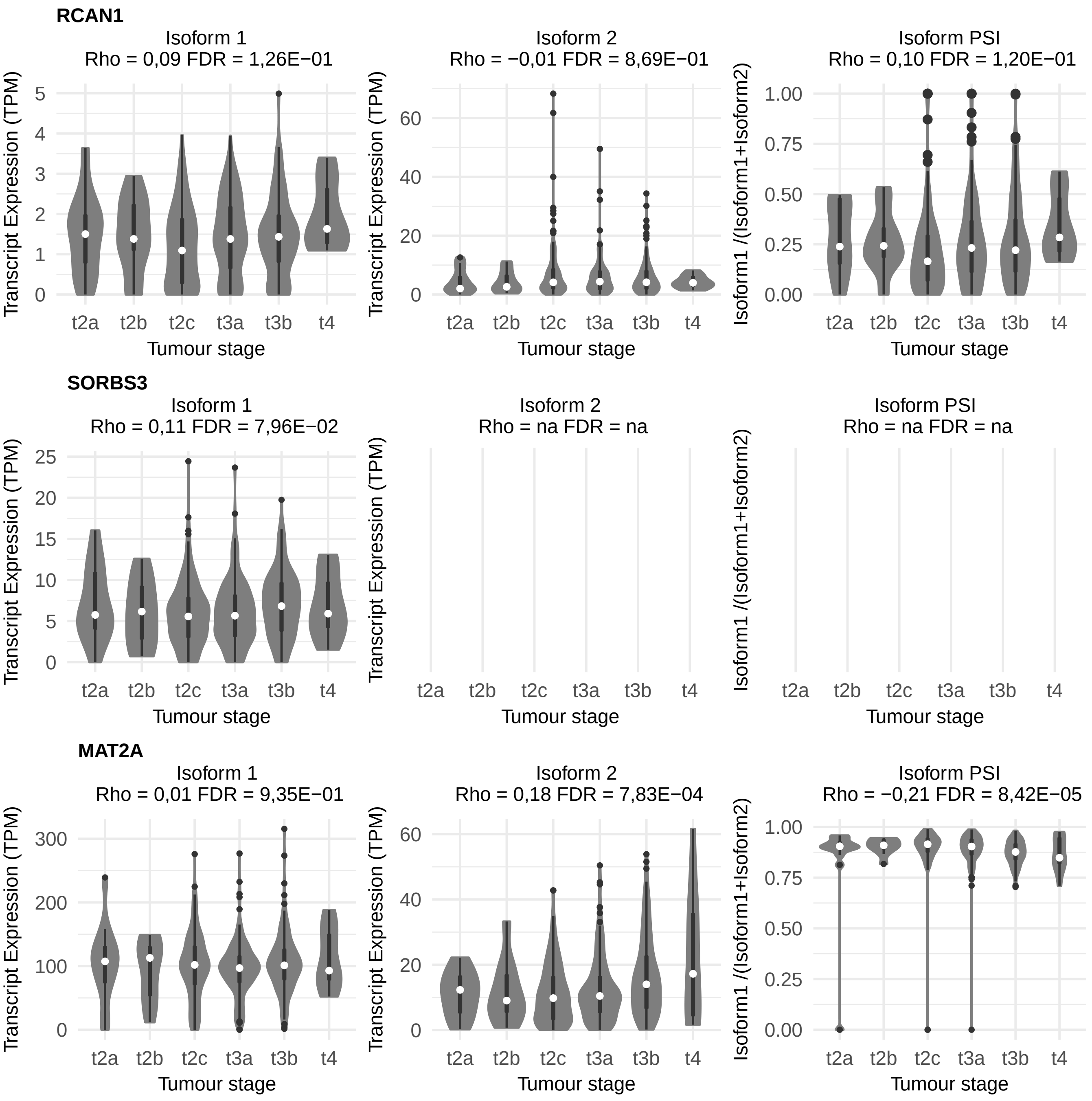

RCAN1
Isoform 1
Rho = 0,09 FDR = 1,26E−01
Isoform 2
Rho = −0,01 FDR = 8,69E−01
Isoform PSI
Rho = 0,10 FDR = 1,20E−01
SORBS3
Isoform 1
Rho = 0,11 FDR = 7,96E−02
Isoform 2
Rho = na FDR = na
Isoform PSI
Rho = na FDR = na
MAT2A
Isoform 1
Rho = 0,01 FDR = 9,35E−01
Isoform 2
Rho = 0,18 FDR = 7,83E−04
Isoform PSI
Rho = −0,21 FDR = 8,42E−05
Transcript Expression (TPM)
Isoform1 /(Isoform1+Isoform2)
Tumour stage
t2a
t2b
t2c
t3a
t3b
t4

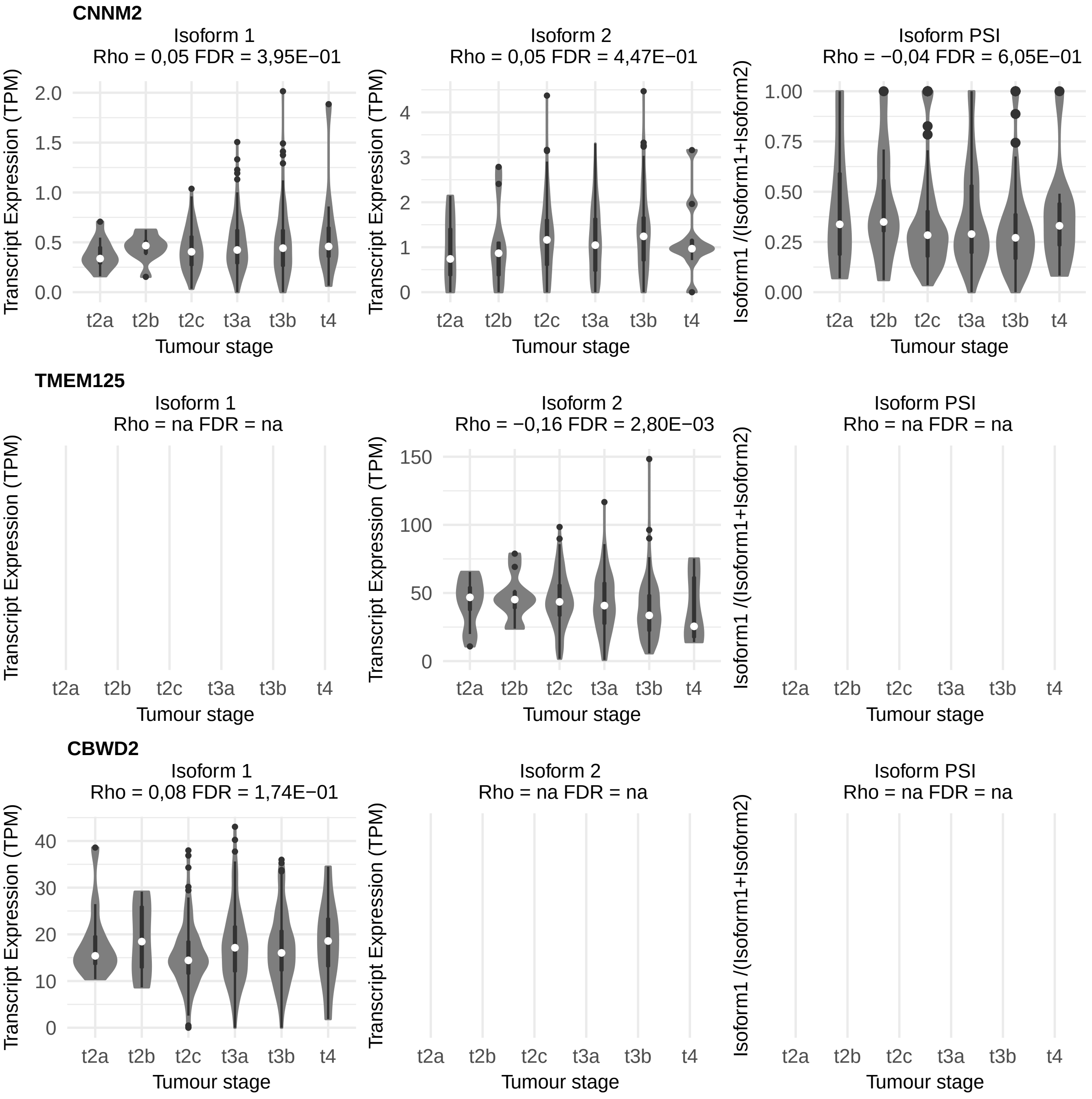

CNNM2
Isoform 1
Rho = 0,05 FDR = 3,95E−01
Isoform 2
Rho = 0,05 FDR = 4,47E−01
Isoform PSI
Rho = −0,04 FDR = 6,05E−01
TMEM125
Isoform 1
Rho = na FDR = na
Isoform 2
Rho = −0,16 FDR = 2,80E−03
Isoform PSI
Rho = na FDR = na
CBWD2
Isoform 1
Rho = 0,08 FDR = 1,74E−01
Isoform 2
Rho = na FDR = na
Isoform PSI
Rho = na FDR = na
Transcript Expression (TPM)
Isoform1 /(Isoform1+Isoform2)
Tumour stage
t2a
t2b
t2c
t3a
t3b
t4

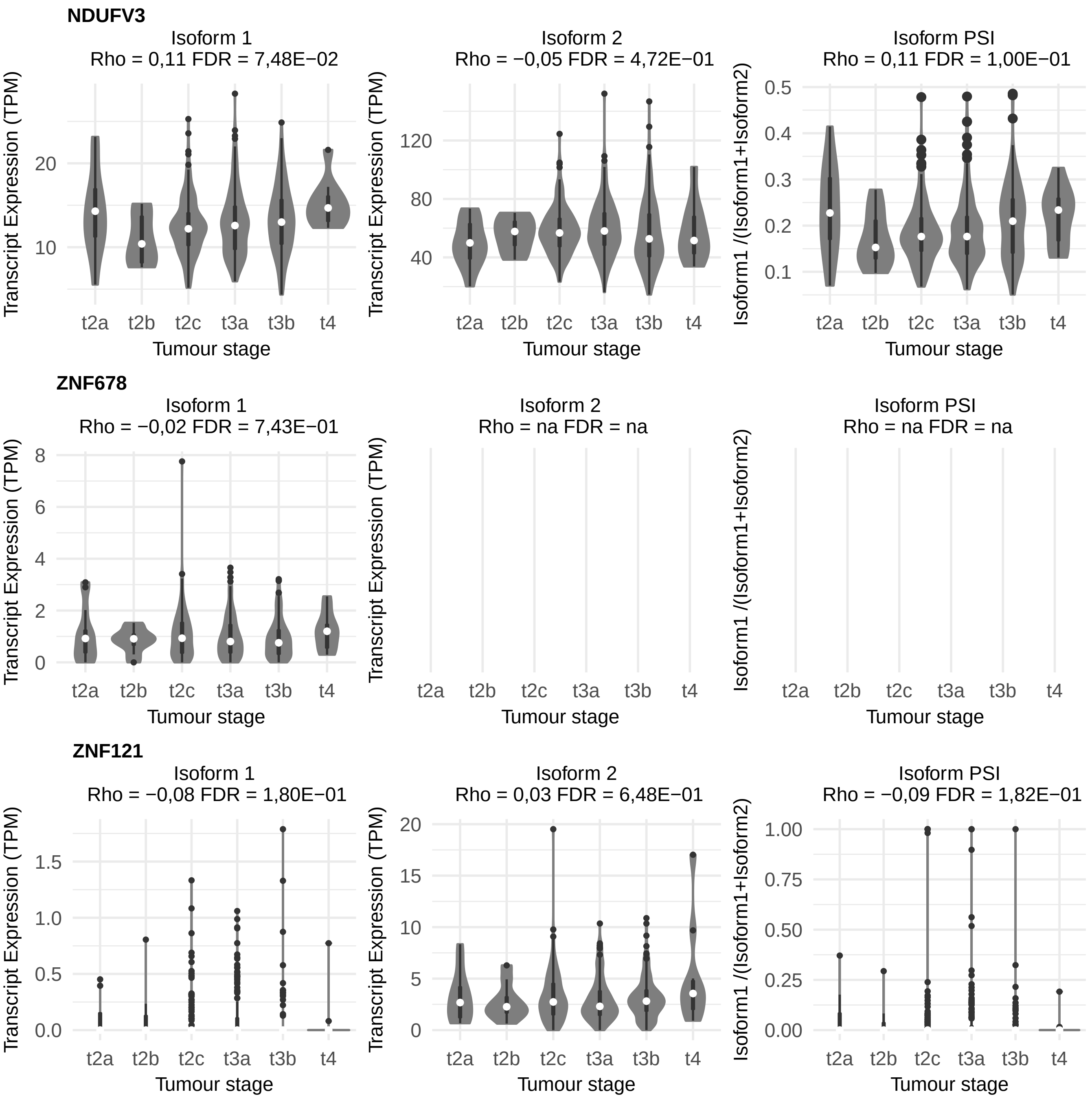
NDUFV3
Isoform 1
Rho = 0,11 FDR = 7,48E−02
Isoform 2
Rho = −0,05 FDR = 4,72E−01
Isoform PSI
Rho = 0,11 FDR = 1,00E−01
ZNF678
Isoform 1
Rho = −0,02 FDR = 7,43E−01
Isoform 2
Rho = na FDR = na
Isoform PSI
Rho = na FDR = na
ZNF121
Isoform 1
Rho = −0,08 FDR = 1,80E−01
Isoform 2
Rho = 0,03 FDR = 6,48E−01
Isoform PSI
Rho = −0,09 FDR = 1,82E−01
Transcript Expression (TPM)
Isoform1 /(Isoform1+Isoform2)
Tumour stage
t2a
t2b
t2c
t3a
t3b
t4

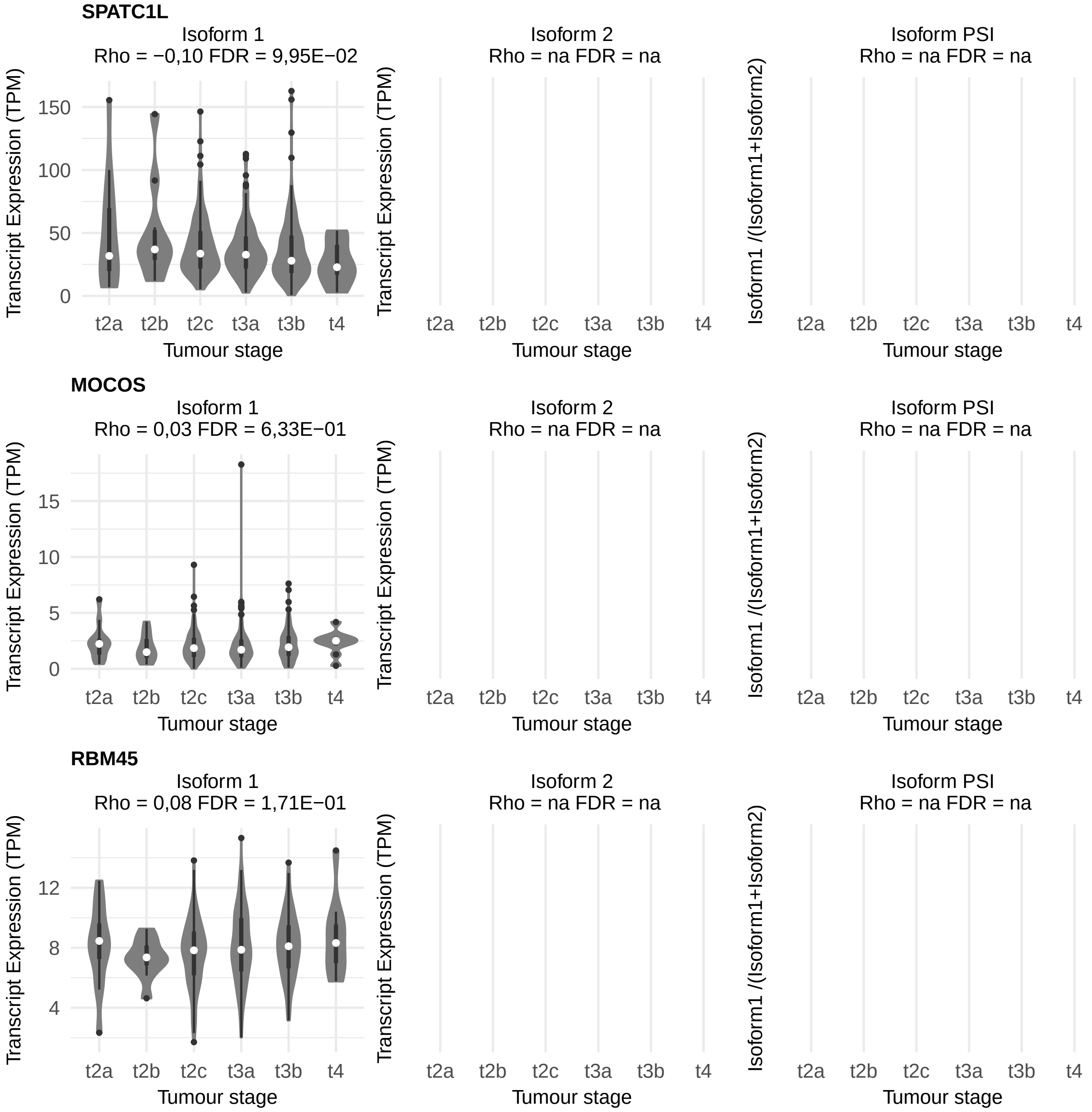
SPATC1L
Isoform 1
Rho = −0,10 FDR = 9,95E−02
Isoform 2
Rho = na FDR = na
Isoform PSI
Rho = na FDR = na
Transcript Expression (TPM)
Isoform1 /(Isoform1+Isoform2)
Tumour stage
t2a
t2b
t2c
t3a
t3b
t4
MOCOS
Isoform 1
Rho = 0,03 FDR = 6,33E−01
Isoform 2
Rho = na FDR = na
Isoform PSI
Rho = na FDR = na
RBM45
Isoform 1
Rho = 0,08 FDR = 1,71E−01
Isoform 2
Rho = na FDR = na
Isoform PSI
Rho = na FDR = na

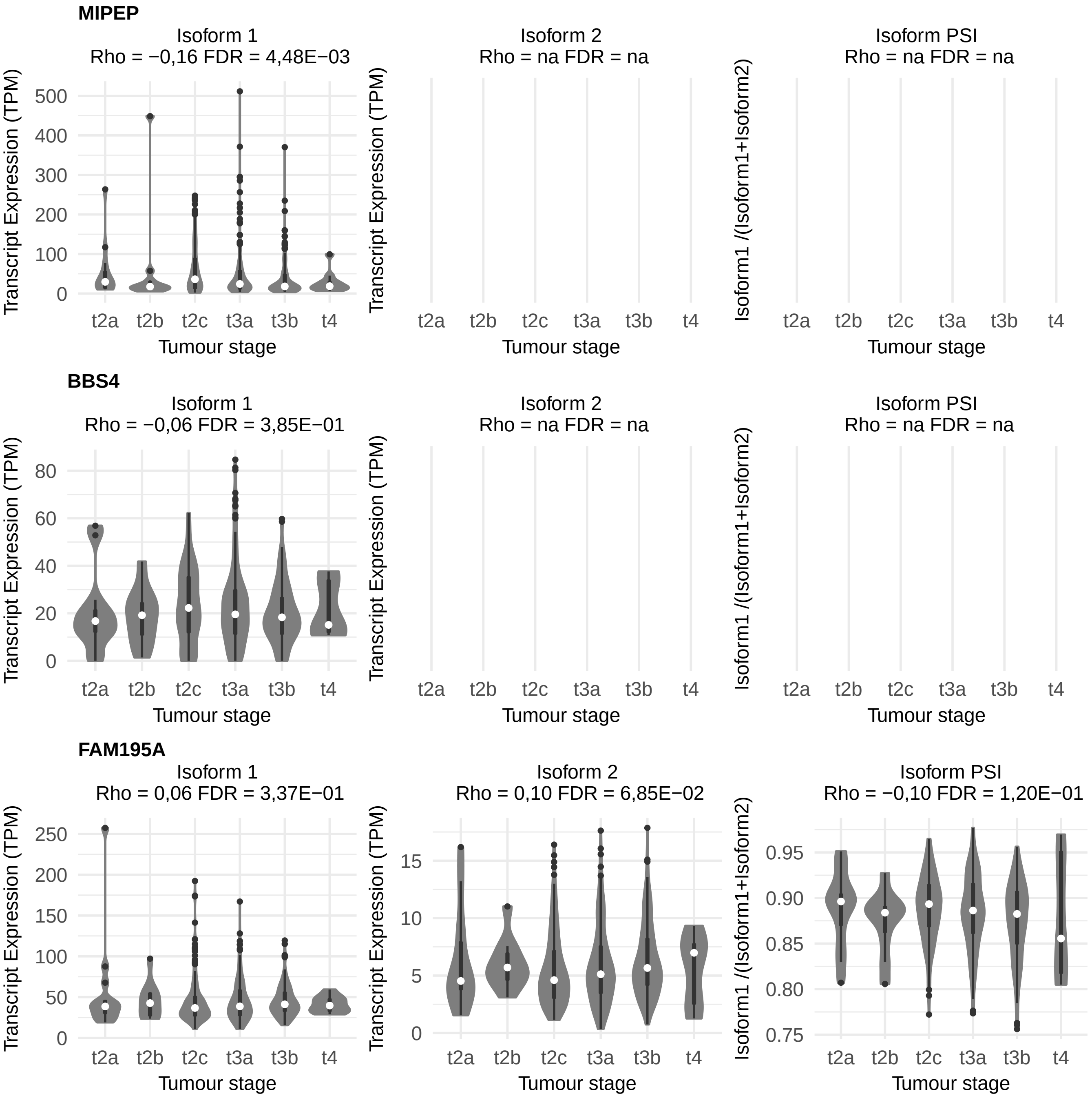

MIPEP
Isoform 1
Rho = −0,16 FDR = 4,48E−03
Isoform 2
Rho = na FDR = na
Isoform PSI
Rho = na FDR = na
BBS4
Isoform 1
Rho = −0,06 FDR = 3,85E−01
Isoform 2
Rho = na FDR = na
Isoform PSI
Rho = na FDR = na
FAM195A
Isoform 1
Rho = 0,06 FDR = 3,37E−01
Isoform 2
Rho = 0,10 FDR = 6,85E−02
Isoform PSI
Rho = −0,10 FDR = 1,20E−01
Transcript Expression (TPM)
Isoform1 /(Isoform1+Isoform2)
t2a
t2b
t2c
t3a
t3b
t4
Tumour stage

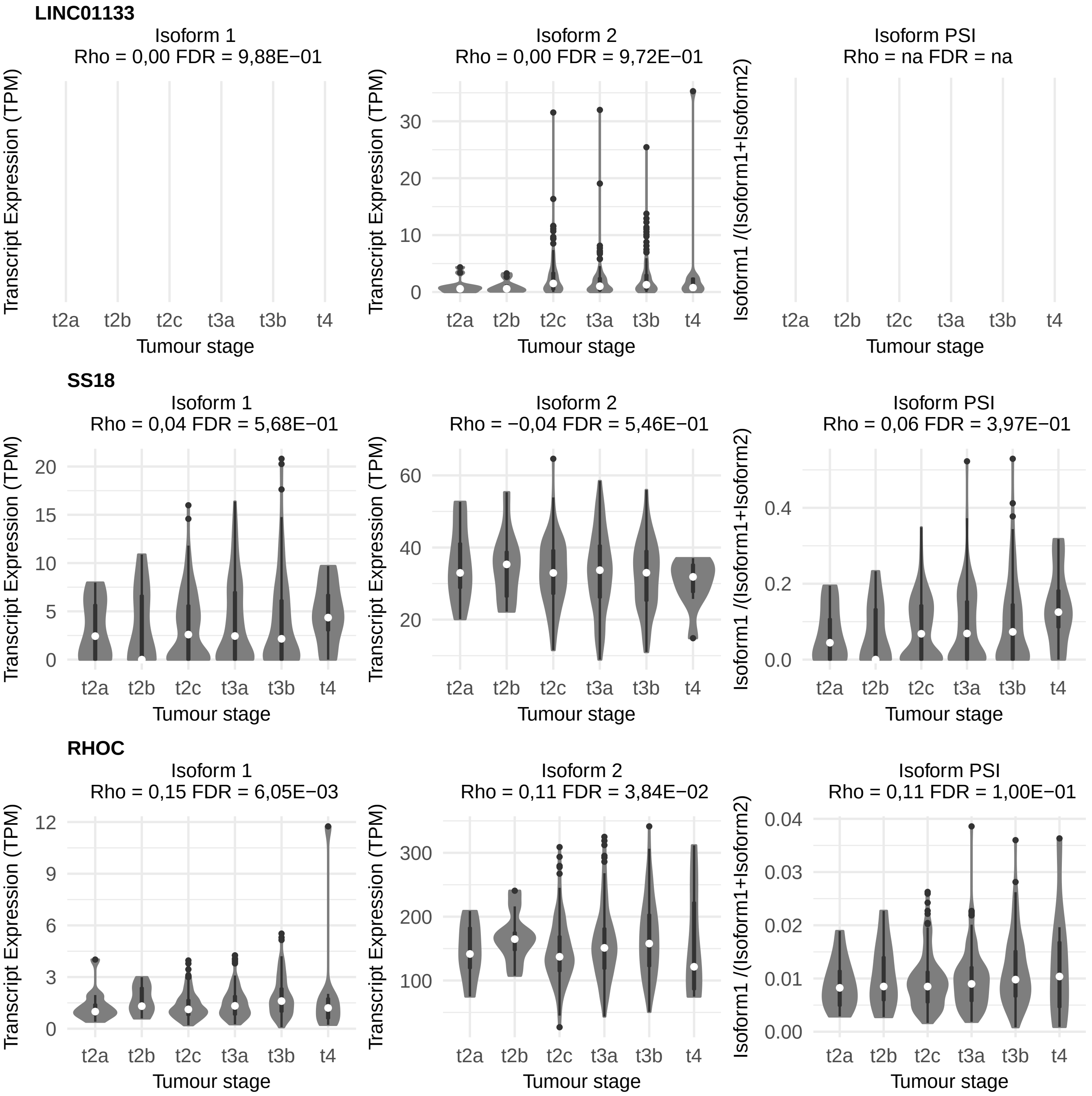
LINC01133
Isoform 1
Rho = 0,00 FDR = 9,88E−01
Isoform 2
Rho = 0,00 FDR = 9,72E−01
Isoform PSI
Rho = na FDR = na
SS18
Isoform 1
Rho = 0,04 FDR = 5,68E−01
Isoform 2
Rho = −0,04 FDR = 5,46E−01
Isoform PSI
Rho = 0,06 FDR = 3,97E−01
RHOC
Isoform 1
Rho = 0,15 FDR = 6,05E−03
Isoform 2
Rho = 0,11 FDR = 3,84E−02
Isoform PSI
Rho = 0,11 FDR = 1,00E−01
Transcript Expression (TPM)
Isoform1 /(Isoform1+Isoform2)
Tumour stage
t2a
t2b
t2c
t3a
t3b
t4

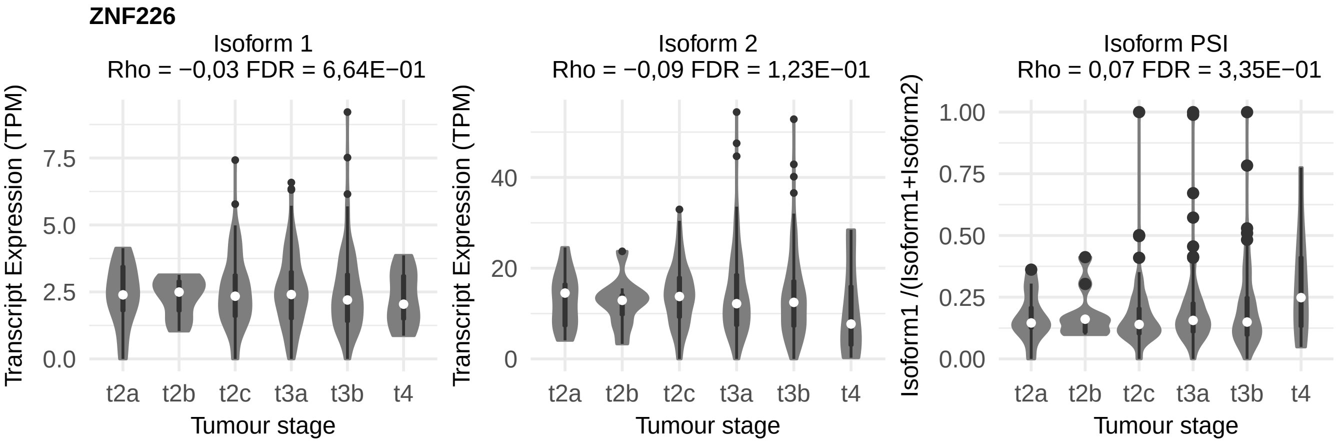
ZNF226
Isoform 1
Rho = −0,03 FDR = 6,64E−01
Transcript Expression (TPM)
0.0
2.5
5.0
7.5
t2a t2b t2c t3a t3b t4
Tumour stage
Isoform 2
Rho = −0,09 FDR = 1,23E−01
Transcript Expression (TPM)
0
20
40
t2a t2b t2c t3a t3b t4
Tumour stage
Isoform PSI
Rho = 0,07 FDR = 3,35E−01
Isoform1 /(Isoform1+Isoform2)
0.00
0.25
0.50
0.75
1.00
t2a t2b t2c t3a t3b t4
Tumour stage
