# Supplementary material for: Androgen-dependent alternative mRNA isoform expression in prostate cancer cells
Source: F1000Res. 2018 Aug 3;7:1189. [Version 1] doi: 10.12688/f1000research.15604.1 (PMC6143958; doi:10.12688/f1000research.15604.1)
Supplement: Peer review history

# Peer review: Referee response for version 1

**DOI:** 10.5256/f1000research.17022.r37702

**Published:** 2018-10-04

**Authors:** Jennifer Byrne

**Affiliations:** 1 Children's Cancer Research Unit, Kids Research Institute, The Children's Hospital at Westmead, Westmead, NSW, Australia

Prostate cancer is a common cancer in men that is driven in part through deregulated androgen signalling. The importance of androgen inhibitors in prostate cancer therapy and the clinical challenges posed by the development of androgen-resistant disease both justify the detailed description of the effects of androgen treatment on gene transcription and alternative splicing in prostate cancer cells. In this sense, the analyses reported by Munkley and colleagues represent valuable additions to the literature. However, further explanation of the results presented would increase the reader’s ability to understand these results and their significance, and to identify which results should be prioritised for further research. I have therefore provided some specific suggestions to increase the accessibility of the data as they are currently presented.

 Specific comments

1. The genes shown in Tables 1, 2, 3 and 5 are not shown in alphabetical order. It is unclear how these genes are ranked and why they are shown in the orders displayed. It would be helpful for any groupings of genes to be more clearly displayed in these tables where this is relevant.
2. It would be helpful to more clearly indicate which findings are novel and which are supported by the literature in Tables and/or Figures.
3. In Table 1, a number of genes are shown in bold, but this is not explained.
4. In Table 1, it would be helpful to annotate the isoform ID’s shown (columns towards the right side of Table). What does “comparable” mean here?
5. It is challenging to show data for a large number of genes, most of which the authors will not be familiar with. However, in Figure 2, incorrect information is shown for the TPD52 gene (panel A). The PrLZ isoform is actually longer than the TPD52 isoform (through an extended N-terminal sequence specific to PrLZ), yet the sizes of these isoforms indicated in the diagram at the right have been switched (TPD52 is incorrectly shown to be the longer isoform). The authors should check whether this is an isolated error or whether other data for the TPD52 and PrLZ isoforms have been switched (for example in Figure 3).
6. It would be helpful for Table 4 to include percentages as well as sample numbers so that readers can compare the composition of the TCGA PRAD cohort with other published cohorts.
7. Analyses compared differential isoform expression in prostate cancer and normal tissue. The cohort included 497 prostate cancer patients, for which 52 had matched normal tissue (Table 5, Figure 3). I’ve assumed that these analyses compared transcript levels in the 497 prostate cancer cases with those in the 52 normal tissue cases. However, given that the 52 normal tissue cases had matched tumour samples available, were analyses conducted to compare the 52 matched cases? These analyses could be argued to be more robust through comparing matched samples, albeit in a smaller cohort.
8. Table 5 should indicate the numbers of tumour and normal tissue samples compared.
9. Some data in Tables 5, 6, and 6 are shown in bold, but this is not explained.
10. I could not open Dataset 2. Could this be made available as a pdf file?
11. All violin plots (Figures 3-5, also supplementary data) should specify the sample numbers compared, either below the X axis or in the figure legend if the same sample numbers apply to every plot shown.

I have read this submission. I believe that I have an appropriate level of expertise to confirm that it is of an acceptable scientific standard.

# Peer review: Referee response for version 1

**DOI:** 10.5256/f1000research.17022.r38286

**Published:** 2018-09-18

**Authors:** Cyril F. Bourgeois

**Affiliations:** 1 INSERM U1210, CNRS UMR 5239, Laboratory of Biology and Modelling of the Cell, Ecole Normale Supérieure de Lyon (ENS Lyon), University of Lyon, Lyon, France

This paper by Munkley and colleagues identifies in a comprehensive manner novel alternative mRNA isoforms regulated by androgens. Interestingly most isoforms result from a choice between alternative promoters, suggesting that regulation takes place mostly at the transcriptional level, but they identified also a few alternative cassette exons and 3' ends. They show experimental validation for 17 isoforms. Beside increasing the number of identified genes in the context of androgen-treated prostate cancer LNCaP cells, the authors analysed the expression of those new isoforms in a large cohort of prostate tumours. They found the expression of some of the mRNA isoforms is positively correlated in the androgen-treated cell and in cancer versus normal samples, and find further correlation with the tumour grade and stage for 3 alternative isoforms.

 Overall this is an interesting work that clearly deserves to be published, as it reveals new potentially interesting target genes for prostate cancer. I have only a couple of comments/questions that may help to improve the strength of the manuscript.

 Did the authors try to experimentally validate the regulation of alternative isoforms for the 3 most interesting genes, i.e. *OSBPL1A*, *CLK3* and *TSC22D3*, which is correlated to tumour stage ? As these new isoforms are predicted to alter the protein sequence, is it possible to discuss or predict what could be the impact of these modifications for these proteins, with regards to what is known about their function and/or in the context of prostate cancer?

 Looking at the RNA-seq profiles for the validated examples, it seems to me that in some cases, especially for RLN1 and RLN2, regulation of promoter choice correspond also to changes in the 3 end of the transcript (the peak seems to be shifted to the 3' end). Such examples may have escaped the in silico prediction, but can you make any comment on this ?

I have read this submission. I believe that I have an appropriate level of expertise to confirm that it is of an acceptable scientific standard.

# Peer review: Referee response for version 1

**DOI:** 10.5256/f1000research.17022.r36819

**Published:** 2018-09-05

**Authors:** Sebastian Oltean

**Affiliations:** 1 Institute of Biomedical and Clinical Sciences, Medical School, University of Exeter, Exeter, UK

Prostate cancer (PCa) is still a significant health problem in UK and across the world. Though a small minority of patients progress to aggressive forms, the absolute number is quite significant due to the high incidence of PCa among men. Therefore, investigation of molecular mechanisms of PCa progression is very important and will hopefully unravel novel therapeutic targets.

 Alternative splicing (AS) has been shown to occur in over 94% of genes in humans. It is therefore a crucial level of gene regulation and not surprisingly involved in virtually every physiological and pathological process. AS de-regulation has been implicated in many diseases, including cancer and in particular PCa, and interestingly, many times it has been shown to drive cancer pathology independently of transcription.

 Since androgens are main players in PCa, the idea of analysing global changes in AS in response to androgens is very welcome to the field. The authors found 10 times more AS isoforms regulated by androgens than previously reported in data from cell culture, most of them occurring through alternative promoter mechanism. They have confirmed and validated part of these changes. They have also analysed the isoforms changes between adenocarcinoma and normal tissues as well as during progression through the Gleason stages of PCa.

 This is a very well thought and executed study, with may informative results. I have a suggestion for the discussion part:

-  one issue in global analysis of splice isoforms is which ones are causal (ie maintain and aggravate the phenotype) and which ones are just associated with the pathological progression; while a full answer to this would need experimental evidence on each individual splicing event, could the authors discuss 1-2 examples, if possible, where the changes at protein level (either sequence or expression level of a particular isoform) would hypothetically have a causal role

I have read this submission. I believe that I have an appropriate level of expertise to confirm that it is of an acceptable scientific standard.
